# Immunosuppressive Sesquiterpenoids from the Edible Mushroom *Craterellus odoratus*

**DOI:** 10.3390/jof7121052

**Published:** 2021-12-08

**Authors:** Quan Dai, Fa-Lei Zhang, Zheng-Hui Li, Juan He, Tao Feng

**Affiliations:** 1 School of Pharmaceutical Sciences, South-Central University for Nationalities, Wuhan 430074, China; quandai@mail.scuec.edu.cn (Q.D.); flzhang@mail.scuec.edu.cn (F.-L.Z.); 2015051@mail.scuec.edu.cn (Z.-H.L.); 2 National Demonstration Center for Experimental Ethnopharmacology Education, South-Central University for Nationalities, Wuhan 430074, China

**Keywords:** *Craterellus ordoratus*, sesquiterpenoids, isolation and structural elucidation, immunosuppressive activity

## Abstract

The aim of this work was to comprehensively understand the chemical constituents of the edible mushroom *Craterellus ordoratus* and their bioactivity. A chemical investigation on this mushroom led to the isolation of 23 sesquiterpenoids including eighteen previously undescribed bergamotane sesquiterpenes, craterodoratins A–R (**1**–**18**), and one new victoxinine derivative, craterodoratin S (**19**). The new structures were elucidated by detailed interpretation of spectrometric data, theoretical nuclear magnetic resonance (NMR) and electronic circular dichroism (ECD) calculations, and single-crystal X-ray crystallographic analysis. Compounds **1** and **2** possess a ring-rearranged carbon skeleton. Compounds **3**, **10**, **12**–**15**, **19**, **20** and **23** exhibit potent inhibitory activity against the lipopolysaccharide (LPS)-induced proliferation of B lymphocyte cells with the IC_50_ values ranging from 0.67 to 22.68 μM. Compounds **17** and **20** inhibit the concanavalin A (ConA)-induced proliferation of T lymphocyte cell with IC_50_ values of 31.50 and 0.98 μM, respectively. It is suggested that *C. ordoratus* is a good source for bergamotane sesquiterpenoids, and their immunosuppressive activity was reported for the first time. This research is conducive to the further development and utilization of *C. ordoratus*.

## 1. Introduction

Edible mushrooms are a large and fascinating group of fungi. Many species of wild edible mushrooms are valued ingredients due to their unique taste and short growth cycle. Yunnan Province is located in southwest China. Its unique climate and geological diversity make this area very favorable for the growth of wild mushrooms. It is estimated that more than 40% of the world’s and 90% of Chinese edible mushrooms (about 900 species) grow in Yunnan [1,2]. Many wild edible mushrooms are regarded as local delicacies such as *Tricholoma matsutake*, *Collybia albuminosa*, *Cantharellus cibarius*, and several species of the genus *Boletus*. Studying the chemical constituents of these edible mushrooms, therefore, has become our long-term research project [2,3,4]. The systematic mining of chemical components and evaluation of their biological activity will be beneficial to the scientific development and utilization of these edible fungi.

**Figure 1:**
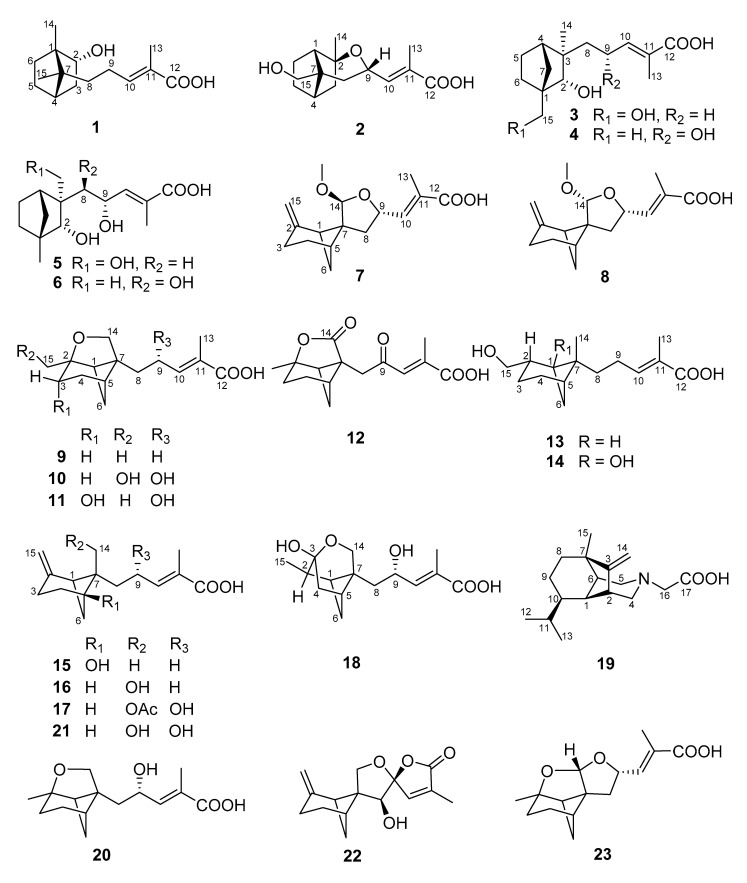
Chemical structures of compounds **1**–**23**.

Species of the genus *Craterellus* (Cantharellaceae) include well-known edible mushrooms. At present, 142 records of *Craterellus* have been found and about 74 species are currently recognized as members of the genus. Ten species are originally described from Asia and four of these species have been reported in China [5]. Of them, *C. odoratus* is an edible mushroom in the family widespread in mainland China and characterized by possessing a bright orange or yellow cap [5,6,7,8]. In China, this wild edible mushroom is especially popular in Yunnan Province, where it is a delicacy on the dinner table from June to August each year. In northern Thailand, *C. odoratus* is also a wild edible mushroom. Its nutritive value including the content of protein, fat, crude fiber, carbohydrate, and mineral contents was assessed. From the perspective of secondary metabolites, this mushroom has not been systematically studied, only a few polyketides and terpenoids have been reported [9,10,11,12,13]. Therefore, it is necessary to conduct systematic chemical component mining and biological activity evaluation on this mushroom. In this study, a total of 23 sesquiterpenoids (**1**–**23**, Figure 1) including eighteen new bergamotane-type ones, namely craterodoratins A–R (**1**–**18**), and one new victoxinine derivative, namely craterodoratin S (**19**), were isolated from *C. odoratus*. Their structures with absolute configurations were elucidated by extensive spectroscopic methods (including 1D and 2D NMR, MS, UV, and IR technologies), single-crystal X-ray diffraction analysis, as well as NMR and ECD calculations.

Previous pharmacological studies on bergamotane metabolites have demonstrated *α*-glucosidase inhibitory activity [14], antibacterial activity [9,15,16], inhibition of pancreatic lipase [17], and phytotoxic effects against Johnson grass and Sorghum [18,19,20,21]. In this study, all compounds were evaluated for their cytotoxic activities against five human cancer cell lines, and for their immunosuppressive activity on T-cell and B-cell proliferation. Herein the isolation, structural elucidation, and bioactivities of these isolates are reported.

## 2. Materials and Methods

### 2.1. General Experimental Procedures

IR spectra were obtained on a Shimadzu Fourier transform infrared spectrometer using KBr pellets. UV spectra were obtained by using a double beam spectrophotometer UH5300 (Hitachi High-Technologies, Tokyo, Japan). Optical rotations were measured on a Rudolph Autopol IV polarimeter (Hackettstown, NJ, USA). High resolution electrospray ionization mass spectra (HRESIMS) were recorded on an Agilent 6200 Q-TOF MS system or a Thermo Scientific Q Exactive Orbitrap MS system. Circular dichroism (CD) spectra were measured with an Applied Photophysics spectrometer (Chirascan, New Haven, CT, USA). NMR spectra were recorded with a Bruker Avance III 600 MHz spectrometer (Bruker, Karlsruhe, Germany). Sephadex LH-20 (GE Healthcare, Pittsburgh, PA, USA), Silica gel (200–300 mesh), and RP-18 gel (20–45 μM, FuJi) were used for column chromatography (CC). HPLC was performed on an Agilent 1260 liquid chromatography system equipped with Zorbax SB-C18 columns (5 μM, 9.4 mm × 150 mm, or 21.2 mm × 150 mm).

### 2.2. Fungal Material

The fungus *Craterellus odoratus* was collected from the Southern part of the Gaoligong Mountains in Yunnan Province, China, in July 2007. The fungus was identified by Prof. Mu Zang at the Kunming Institute of Botany. A voucher specimen (HFC2007-20180714-DQ1) has been deposited in the School of Pharmaceutical Sciences, South-Central University for Nationalities.

### 2.3. Fermentation, Extraction and Isolation

The rice culture medium was composed of glucose 5%, yeast 5%, pork peptone 0.15%, KH_2_PO_4_ 0.05%, and MgSO_4_ 0.05%. The initial pH was adjusted to 6.0. Cultures were grown in an Erlenmeyer flask (220 rpm, 24 °C) for 6 days until the mycelium biomass reached a maximum. This was then transferred to a rice medium and incubated at 24 °C in the dark for 40 days. The rice medium contained 50 g of rice and 50 mL of water, in a 250 mL Erlenmeyer flask, sterilized at 121 °C for 15 min. A total of 400 flasks were used in this work.

The solid rice culture broth of *C. odoratus* (20 kg) was extracted six time with MeOH to give a crude extract. The extract was partitioned between water and ethyl acetate (EtOAc). The EtOAc layer was concentrated under reduced pressure to give an organic extract (167 g). It was subjected to CC over silica gel (200–300 mesh) eluted with a solvent system of CHCl_3_/MeOH (from 1:0 to 0:1, *v*/*v*) to obtain nine fractions A–I. Fraction D (20 g) was fractionated by MPLC over RP-18 silica gel eluted with MeOH/H_2_O (from 5:95 to 100:0, *v*/*v*) to give 13 subfractions (D_1_–D_13_). Fraction D_6_ (5 g) was repeatedly fractionated by CC over silica gel eluted with CHCl_3_/MeOH (10:1) to give compounds **9** (7 mg), **10** (4 mg), and **11** (18 mg). Fraction D_4_ (3 g) was fractionated by CC over Sephadex LH-20 (MeOH) and then purified by prep-HPLC (CH_3_CN/H_2_O 35:65 in 30 min) to give compounds **1** (3.0 mg, retention time (*t*_R_) = 16.3 min), **8** (3.2 mg, *t*_R_ = 20.1 min), and **4** (3.4 mg, *t*_R_ = 24.7 min). Fraction D_3_ (5 g) was fractionated by CC over Sephadex LH-20 (MeOH) and then purified by prep-HPLC (CH_3_CN/H_2_O 32:68 in 25 min) to give compounds **17** (2.3 mg, *t*_R_ = 13.2 min), **20** (3.0 mg, *t*_R_ = 15.4 min), and **18** (2.6 mg, *t*_R_ = 22.0 min). Fraction E (9 g) was fractionated by MPLC over RP-18 eluted with MeOH/H_2_O (from 5:95 to 100:0, *v*/*v*) to give seven subfractions (E_1_–E_7_). Fraction E_3_ (800 mg) was subjected to CC over silica gel (80–100 mesh), Sephadex LH-20 (MeOH), and then purified by prep-HPLC (CH_3_CN/H_2_O from 25:75 to 61:39 in 25 min) to give compounds **3** (2.1 mg, *t*_R_ = 11.2 min), **6** (3 mg, *t*_R_ = 15.3 min), **7** (2.5 mg, *t*_R_ = 17.9 min), and **16** (3.8 mg, *t*_R_ = 23.1 min). Fraction E_5_ (36 mg) was prepared by prep-HPLC (CH_3_CN/H_2_O 30:70 in 25 min) to give compounds **14** (2.1 mg, *t*_R_ = 14.8 min), **13** (2.4 mg, *t*_R_ = 18.6 min), and **15** (2.6 mg, *t*_R_ = 23.0 min). Fraction F (7 g) was separated by MPLC over RP-18 to give five subfractions F_1_–F_5_. Fraction F_3_ (23 mg) was prepared by prep-HPLC (CH_3_CN/H_2_O 38:62 in 25 min) to give compounds **2** (2.3 mg, *t*_R_ = 15.1 min), **12** (2.0 mg, *t*_R_ = 17.6 min), and **5** (3.1 mg, *t*_R_ = 20.3 min). Fraction F_4_ (30 mg) was prepared by prep-HPLC (CH_3_CN/H_2_O 40:60 in 25 min) to give compounds **21** (2.3 mg, *t*_R_ = 13.0 min), **22** (1.8 mg, *t*_R_ = 17.0 min), and **19** (3.1 mg, *t*_R_ = 21.3 min). Fraction G (1.2 g) was fractionated by CC over silica gel eluted with CHCl_3_/CH_3_OH (10:1) to give subfractions G_1_−G_6_. Compound **23** (3.2 mg) precipitated as acicular crystals from fraction G_3_.

*Craterodoratin A* (**1**). Colorless oil; ${\left[\alpha \right]}_{D}^{21}$ −1.9 (*c* 0.11, MeOH); UV (MeOH) *λ*_max_ (log *ε*) 210 (1.04) nm; IR (KBr) *ν*_max_ 3365, 2945, 2833, 1660, 1454, 1114, 1031 cm^−1^; ^1^H and ^13^C NMR spectroscopic data, see Table 1; HRESIMS *m*/*z* 253.17986 [M + H]^+^ (calcd for C_15_H_25_O_3_^+^, 253.17982).

*Craterodoratin B* (**2**). Colorless crystals (MeOH), mp 138.2–140.3 °C; ${\left[\alpha \right]}_{D}^{21}$ − 11.4 (*c* 0.09, MeOH); UV (MeOH) *λ*_max_ (log *ε*) 215 (1.88) nm; IR (KBr) *ν*_max_ 3442, 2951, 2841, 1647, 1018 cm^−1^; ^1^H and ^13^C NMR spectroscopic data, see Table 1; HRESIMS *m*/*z* 267.15903 [M + H]^+^ (calcd for C_15_H_23_O_4_^+^, 267.15909).

*Craterodoratin C* (**3**). Colorless crystals (MeOH), mp 180.7–182.4 °C; ${\left[\alpha \right]}_{D}^{21}$ − 14.5 (*c* 0.11, MeOH); UV (MeOH) *λ*_max_ (log *ε*) 230 (3.12) nm; IR (KBr) *ν*_max_ 3464, 2924, 1647, 1280, 1265 cm^−1^; ^1^H and ^13^C NMR spectroscopic data, see Table 1; HRESIMS *m*/*z* 291.15650 [M + Na]^+^ (calcd for C_15_H_24_O_4_Na^+^, 291.15668).

*Craterodoratin D* (**4**). Colorless crystals (MeOH), mp 159.1–162.5 °C; ${\left[\alpha \right]}_{D}^{21}$ + 32 (*c* 0.10, MeOH); UV (MeOH) *λ*_max_ (log *ε*) 225 (3.02) nm; IR (KBr) *ν*_max_ 3468, 2967, 2541, 1647, 1016 cm^−1^; ^1^H and ^13^C NMR spectroscopic data, see Table 1; HRESIMS *m*/*z* 267.14712 [M − H]^−^ (calcd for C_15_H_23_O_4_^−^, 267.16018).

*Craterodoratin E* (**5**). Colorless crystals (MeOH), mp 140.9–142.4 °C; ${\left[\alpha \right]}_{D}^{21}$ + 6.9 (*c* 0.10, MeOH); UV (MeOH) *λ*_max_ (log *ε*) 210 (1.27) nm; IR (KBr) *ν*_max_ 3408, 2951, 2841, 1651, 1018 cm^−1^; ^1^H and ^13^C NMR spectroscopic data, see Table 2; HRESIMS *m*/*z* 307.15146 [M + Na]^+^ (calcd for C_15_H_24_O_5_Na^+^, 307.15159).

*Craterodoratin F* (**6**). Colorless oil; ${\left[\alpha \right]}_{D}^{21}$ + 18 (*c* 0.10, MeOH); UV (MeOH) *λ*_max_ (log *ε*) 210 (0.84) nm; IR (KBr) *ν*_max_ 3400, 2949, 2837, 1653, 1456, 1411, 1112, 1024 cm^−1^; ^1^H and ^13^C NMR spectroscopic data, see Table 2; HRESIMS *m*/*z* 307.15161 [M + Na]^+^ (calcd for C_15_H_24_O_5_Na^+^, 307.15159).

*Craterodoratin G* (**7**). Colorless oil; ${\left[\alpha \right]}_{D}^{21}$ + 1.10 (*c* 0.10, MeOH); UV (MeOH) *λ*_max_ (log *ε*) 210 (1.74) nm; IR (KBr) *ν*_max_ 3444, 2933, 1647, 1091, 1016 cm^−1^; ^1^H and ^13^C NMR spectroscopic data, see Table 2; HRESIMS *m*/*z* 279.15903 [M + H]^+^ (calcd for C_15_H_23_O_4_^+^, 279.15909).

*Craterodoratin H* (**8**). Colorless oil; ${\left[\alpha \right]}_{D}^{21}$ − 30.6 (*c* 0.16, MeOH); UV (MeOH) *λ*_max_ (log *ε*) 210 (0.68) nm; IR (KBr) *ν*_max_ 3398, 2949, 2835, 1653, 1452, 1112, 1031 cm^−1^; ^1^H and ^13^C NMR spectroscopic data, see Table 2; HRESIMS *m*/*z* 279.15920 [M + H]^+^ (calcd for C_15_H_23_O_4_^+^, 279.15909).

*Craterodoratin I* (**9**). Colorless oil; ${\left[\alpha \right]}_{D}^{21}$ + 31.6 (*c* 0.10, MeOH); UV (MeOH) *λ*_max_ (log *ε*) 230 (3.01) nm; IR (KBr) *ν*_max_ 3379, 2947, 2835, 1653, 1456, 1114, 1031 cm^−1^; ^1^H and ^13^C NMR spectroscopic data, see Table 3; HRESIMS *m*/*z* 251.16409 [M + H]^+^ (calcd for C_15_H_23_O_3_^+^, 251.16417).

*Craterodoratin J* (**10**). Colorless oil; ${\left[\alpha \right]}_{D}^{21}$ + 52 (*c* 0.10, MeOH); UV (MeOH) *λ*_max_ (log *ε*) 210 (1.48) nm; IR (KBr) *ν*_max_ 3379, 2947, 2835, 1653, 1456, 1114, 1031 cm^−1^; ^1^H and ^13^C NMR spectroscopic data, see Table 3; HRESIMS *m*/*z* 305.13602 [M + Na]^+^ (calcd for C_15_H_22_O_5_Na^+^, 305.13594).

*Craterodoratin K* (**11**). Colorless oil; ${\left[\alpha \right]}_{D}^{21}$ + 37 (*c* 0.10, MeOH); UV (MeOH) *λ*_max_ (log *ε*) 210 (1.34) nm; IR (KBr) *ν*_max_ 3456, 2924, 2815, 1647, 1396, 1018 cm^−1^; ^1^H and ^13^C NMR spectroscopic data, see Table 3; HRESIMS *m*/*z* 283.15136 [M + H]^+^ (calcd for C_15_H_23_O_5_^+^, 283.15400).

*Craterodoratin L* (**12**). Colorless oil; ${\left[\alpha \right]}_{D}^{21}$ + 53 (*c* 0.11, MeOH); UV (MeOH) *λ*_max_ (log *ε*) 235 (1.12) nm; IR (KBr) *ν*_max_ 3390, 2947, 2833, 1653, 1456, 1112, 1031 cm^−1^; ^1^H and ^13^C NMR spectroscopic data, see Table 3; HR-ESIMS *m*/*z* 279.12250 [M + H]^+^ (calcd for C_15_H_19_O_5_^+^, 279.12270).

*Craterodoratin M* (**13**). Colorless oil; ${\left[\alpha \right]}_{D}^{21}$ − 23 (*c* 0.10, MeOH); UV (MeOH) *λ*_max_ (log *ε*) 215 (1.90) nm; IR (KBr) *ν*_max_ 3487, 1645 cm^−1^; ^1^H and ^13^C NMR spectroscopic data, see Table 4; HRESIMS *m*/*z* 253.17992 [M + H]^+^ (calcd for C_15_H_25_O_3_^+^, 253.17982).

*Craterodoratin N* (**14**). Colorless oil; ${\left[\alpha \right]}_{D}^{21}$ − 11 (*c* 0.10, MeOH); UV (MeOH) *λ*_max_ (log *ε*) 210 (1.04) nm; IR (KBr) *ν*_max_ 3487, 1645 cm^−1^; ^1^H and ^13^C NMR spectroscopic data, see Table 4; HRESIMS *m*/*z* 291.15658 [M + Na]^+^ (calcd for C_15_H_24_O_4_Na^+^, 291.15668).

*Craterodoratin O* (**15**). Colorless oil; ${\left[\alpha \right]}_{D}^{21}$ − 28.6 (*c* 0.10, MeOH); UV (MeOH) *λ*_max_ (log *ε*) 210 (1.48) nm; IR (KBr) *ν*_max_ 3460, 2951, 2843, 1645, 1016 cm^−1^; ^1^H and ^13^C NMR spectroscopic data, see Table 4; HRESIMS *m*/*z* 251.16431 [M + H]^+^ (calcd for C_15_H_23_O_3_^+^, 251.16417).

*Craterodoratin P* (**16**). Colorless oil; ${\left[\alpha \right]}_{D}^{21}$ − 27.9 (*c* 0.10, MeOH); UV (MeOH) *λ*_max_ (log *ε*) 210 (1.77) nm; IR (KBr) *ν*_max_ 3367, 2943, 2833, 1654, 1456, 1112, 1031 cm^−1^; ^1^H and ^13^C NMR spectroscopic data, see Table 4; HRESIMS *m*/*z* 251.16417 [M + H]^+^ (calcd for C_15_H_23_O_3_H^+^, 251.16417).

*Craterodoratin Q* (**17**). Colorless oil; ${\left[\alpha \right]}_{D}^{21}$ + 22 (*c* 0.07, MeOH), UV (MeOH) *λ*_max_ (log *ε*) 210 (1.37) nm; IR (KBr) *ν*_max_ 3468, 1645, 1016 cm^−1^; ^1^H and ^13^C NMR spectroscopic data, see Table 5; HRESIMS *m*/*z* 331.15140 [M + Na]^+^ (calcd for C_17_H_24_O_5_Na^+^, 331.15159).

*Craterodoratin R* (**18**). Colorless oil; ${\left[\alpha \right]}_{D}^{21}$ − 42 (*c* 0.10, MeOH); UV (MeOH) *λ*_max_ (log *ε*) 215 (1.82) nm; IR (KBr) *ν*_max_ 3412, 2924, 1637, 1574, 1435, 1065 cm^−1^; ^1^H and ^13^C NMR spectroscopic data, see Table 5; HRESIMS *m*/*z* 305.13596 [M +Na]^+^ (calcd for C_15_H_22_O_5_Na^+^, 305.13594).

*Craterodoratin S* (**19**). Colorless oil; ${\left[\alpha \right]}_{D}^{21}$ − 125 (*c* 0.10, MeOH); UV (MeOH) *λ*_max_ (log *ε*) 210 (1.04) nm; IR (KBr) *ν*_max_ 3412, 2924, 1637, 1574, 1435, 1065 cm^−1^; ^1^H and ^13^C NMR spectroscopic data, see Table 5; HRESIMS *m*/*z* 278.21140 [M +H]^+^ (calcd for C_17_H_28_NO_2_^+^, 278.21146).

*X-ray Crystallographic Data for Craterodoratin B* (**2**). C_15_H_22_O_4_·H_2_O, *M* = 284.34, *a* = 8.6658(3) Å, *b* = 10.0982(4) Å, *c* = 17.1595(6) Å, *α* = 90°, *β* = 90°, *γ* = 90°, *V* = 1501.61(9) Å^3^, *T* = 100(2) K, space group *P*212121, *Z* = 4, *μ*(Cu K*α*) = 0.768 mm^−1^, 12029 reflections measured, 2967 independent reflections (*R*_int_ = 0.0391). The final *R*_1_ values were 0.0314 (*I* > 2*σ*(*I*)). The final *wR*(*F*^2^) values were 0.0802 (*I* > 2*σ*(*I*)). The final *R*_1_ values were 0.0325 (all data). The final *wR*(*F*^2^) values were 0.0813 (all data). The goodness of fit on *F*^2^ was 1.049. Flack parameter = 0.06(7). CCDC: 2059695 (https://www.ccdc.cam.ac.uk (accessed on 13 November 2021)).

*X-ray Crystallographic Data for Craterodoratin C* (**3**). C_15_H_24_O_4_, *M* = 268.34, *a* = 9.9718(3) Å, *b* = 7.2863(2) Å, *c* = 10.4844(3) Å, *α* = 90°, *β* = 111.5850(10)°, *γ* = 90°, *V* = 708.35(4) Å^3^, *T* = 100(2) K, space group *P*1211, *Z* = 2, *μ*(Cu K*α*) = 0.729 mm^−1^, 13370 reflections measured, 2751 independent reflections (*R*_int_ = 0.0334). The final *R*_1_ values were 0.0290 (*I* > 2*σ*(*I*)). The final *wR*(*F*^2^) values were 0.0749 (*I* > 2*σ*(*I*)). The final *R*_1_ values were 0.0291 (all data). The final *wR*(*F*^2^) values were 0.0750 (all data). The goodness of fit on *F*^2^ was 1.039. Flack parameter = 0.06(6). CCDC: 2059696 (https://www.ccdc.cam.ac.uk (accessed on 13 November 2021)).

*X-ray Crystallographic Data for Craterodoratin D* (**4**). C_15_H_24_O_4_, *M* = 268.34, *a* = 6.4802(4) Å, *b* = 10.8539(7) Å, *c* = 10.8186(7) Å, *α* = 90.00°, *β* = 101.555(2)°, *γ* = 90.00°, *V* = 745.51(8) Å^3^, *T* = 297(2) K, space group *P*1211, *Z* = 2, *μ*(Cu K*α*) = 1.54178, 15119 reflections measured, 3109 independent reflections (*R*_int_ = 0.0263). The final *R*_1_ values were 0.0293 (all data). The final *wR*(*F*^2^) values were 0.0798 (all data). The goodness of fit on *F*^2^ was 1.051. Flack parameter = 0.09(4). CCDC: 2059697 (https://www.ccdc.cam.ac.uk (accessed on 13 November 2021)).

*X-ray Crystallographic Data for Craterodoratin E* (**5**). C_15_H_24_O_5_·H_2_O, *M* = 302.36, *a* = 25.5045(11) Å, *b* = 6.4589(3) Å, *c* = 19.4274(8) Å, *α* = 90°, *β* = 97.923(2)°, *γ* = 90°, *V* = 3169.7(2) Å^3^, *T* = 100(2) K, space group C121, *Z* = 8, *μ*(Cu K*α*) = 0.805 mm^−1^, 50,328 reflections measured, 6168 independent reflections (*R*^int^ = 0.0956). The final *R*_1_ values were 0.0680 (*I* > 2*σ*(*I*)). The final *wR*(*F*^2^) values were 0.1739 (*I* > 2*σ*(*I*)). The final *R*_1_ values were 0.0741 (all data). The final *wR*(*F*^2^) values were 0.1821 (all data). The goodness of fit on *F*^2^ was 1.085. Flack parameter = 0.26(11). CCDC: 2059698 (https://www.ccdc.cam.ac.uk (accessed on 13 November 2021)).

### 2.4. NMR and ECD Calculations

Details of NMR and ECD calculations for compounds **1**, **6**‒**8**, **10**, **11** and **18** were given in the Supplementary Materials.

### 2.5. Cytotoxicity Assay

There are five human cancer cell lines using in this assay including human myeloid leukemia HL-60, human breast cancer MCF-7, human colon cancer SW480, human hepatocellular carcinoma SMMC-7721, and human lung cancer A-549 cells. All selected cells were stored in DMEM medium or RPMI-1640, supplemented with 10% fetal bovine serum (Hyclone, Logan, UT, USA) at 37 °C. The MTT (3-(4,5-dimethylthiazol-2-yl)-2,5-diphenyl tetrazolium bromide) method in 96-well microplates was used for cytotoxicity assay [22]. In brief, 100 µL adherent cells were seeded into each well for 12 h before drug addition, while suspended cells were seeded just before adding the test compound with initial density of 1 × 10^5^ cells/mL. The tumor cell line was exposed to the test compound at concentrations of 0.0625, 0.32, 1.6, 8 and 40 μM in triplicates for 48 h. Taxol (Sigma, St. Louis, MO, USA) was used as a positive control. The cell viability was detected, and the cell growth curve was graphed after compound treatment. The IC_50_ values were calculated by the Reed and Muench’s method [23].

### 2.6. Immunosuppressive Activities Assay

#### 2.6.1. Preparation of Spleen Cells from Mice

Female BALB/c mice were sacrificed by cervical dislocation, and their spleens were aseptically removed. After cell debris, mononuclear cell suspensions were prepared, and clumps were removed. Erythrocytes were depleted with ammonium chloride buffer solution. Lymphocytes were washed and resuspended in RPMI 1640 medium supplemented with 10% FBS, penicillin (100 U/mL), and streptomycin (100 mg/mL).

#### 2.6.2. Cytotoxicity Assay

We used the Cell Counting Kit-8 (CCK-8) assay to test cytotoxicity. In brief, fresh spleen cells were obtained from female BALB/c mice (18–20 g). Spleen cells (1 × 10^6^ cells) were seeded in triplicate in 96-well flat plates and cultured at 37 °C for 48 h, with or without compounds of various concentrations, in a humidified and 5% CO_2_-containing incubator. A certain amount of CCK-8 was added to each well during the last 8–10 h of culture. At the end of the culture, we use a microplate reader (Bio-Rad 650) to measure the OD value at 450 nm. Cyclosporin A (CsA) is an immunosuppressant agent which is used as a positive control with definite activity. Only the OD value of culture medium is used as background. The cytotoxicity of compounds was expressed as the concentration of the compounds that reduces cell viability to 50% (CC_50_).

#### 2.6.3. T and B Cell Function Assay

As mentioned above, fresh spleen cells were obtained from female BALB/c mice (18–20 g). Similarly, the 5 × 10^5^ spleen cells were cultured at the same conditions. The cultures, with or without various concentrations of compounds, were stimulated with 5 µg/mL of concanavalin A (ConA) to induce the T cells’ proliferative response or 10 µg/mL of lipopolysaccharide (LPS) to induce B cells’ proliferative response. Proliferation was assessed in terms of uptake of [^3^H]-thymidine during 8 h of pulsing with 25 µL/well of [^3^H]-thymidine, then cells will be harvested onto glass fiber filters. A Beta scintillation counter was used to count the incorporated radioactivity. Cells treated without any stimuli were used as a negative control. The immunosuppressive activity was expressed as the concentration of the compound that inhibited T or B cell proliferation to 50% (IC_50_) of the control value. Both the cytotoxicity and proliferation assessment were repeated twice.

## 3. Results and Discussion

**Figure 2:**
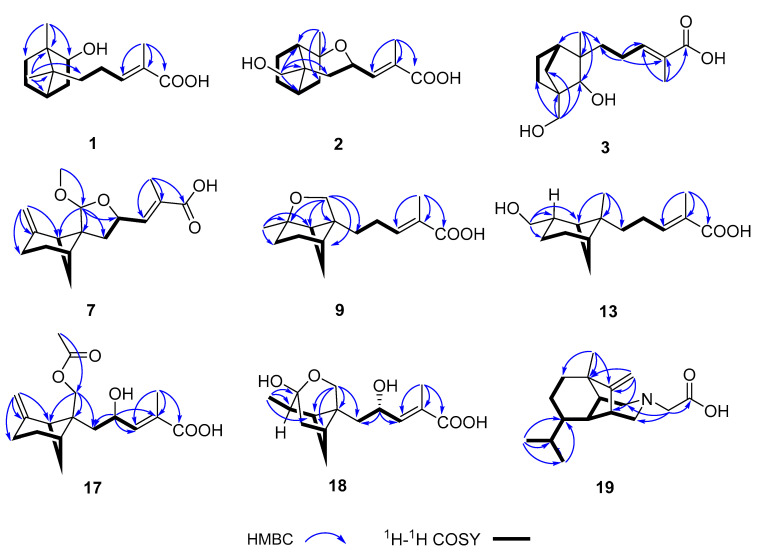
^1^H-^1^H COSY and key HMBC correlations of **1**–**3**, **7**, **9**, **13**, and **17**–**19**.

**Figure 3:**
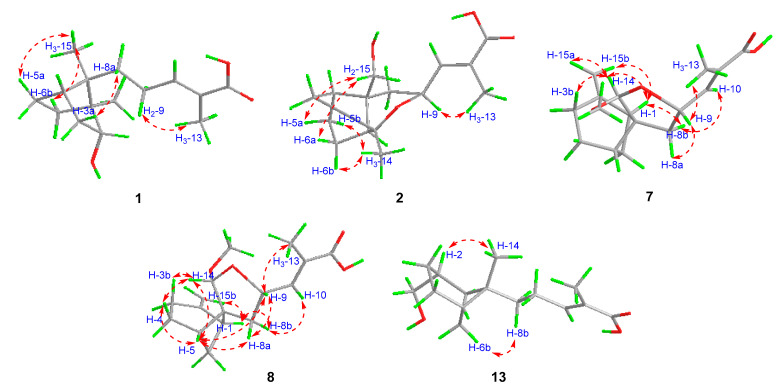
ROESY correlations of **1**, **2**, **7**, **8**, and **13**.

Compound **1** was isolated as a colorless oil. Its molecular formula of C_15_H_24_O_3_ was determined by positive high-resolution electrospray ionization mass spectrometry (HRESIMS) analysis, corresponding to four degrees of unsaturation. In the ^1^H NMR data, three singlets for methyl groups were readily observed at *δ*_H_ 1.80 (3H, s, Me-13), *δ*_H_ 0.93 (3H, s, Me-15), *δ*_H_ 0.85 (3H, s, Me-14) (Table 1). The ^13^C NMR and DEPT data revealed 15 carbon resonances including three CH_3_, five CH_2_, three CH, and four non-protonated carbons (Table 1). Of them, two olefinic carbons at *δ*_C_ 141.1 (C-10) and 128.5 (C-11), and one carboxy carbon at *δ*_C_ 171.3 (C-12) occupied two degrees of unsaturation, which suggested that **1** should be a bicycle sesquiterpenoid. Preliminary analysis of 1D and 2D NMR data, with respect to those in previous isolated from the same source, suggested that **1** might be a bergamotane-type sesquiterpenoid. The ^1^H-^1^H COSY spectrum afforded evidence to form a fragment as shown in Figure 2. Through the HMBC correlations from *δ*_H_ 0.85 (3H, s, Me-14) to *δ*_C_ 49.9 (C-1), 76.3 (C-2), 25.8 (C-6), and 50.9 (C-7), as well as correlations from *δ*_H_ 0.93 (3H, s, Me-15) to *δ*_C_ 49.9 (C-1), 41.9 (C-4), 50.9 (C-7), and 30.9 (C-8), the positions of two methyl groups including Me-14 at C-1 and Me-15 at C-7 were established (Figure 2). Furthermore, the carboxyl group was identified to be conjugated with the double bond by HMBC correlations from *δ*_H_ 1.80 (3H, s, Me-13) to *δ*_C_ 141.1 (C-10), 128.5 (C-11), and 171.3 (C-12). In the ROESY spectrum (Figure 3), the cross peaks of H_3_-15/H-5a (*δ*_H_ 1.69), H_3_-15/H-6b (*δ*_H_ 1.23), and H-3a (*δ*_H_ 2.16)/H-8a (*δ*_H_ 1.46) indicated that Me-15 and CH_2_-5, CH_2_-6 were on the same side. The cross peaks of H_2_-9/H_3_-13 and the absent correlation between H_3_-13 and H-10 confirmed the *E*-configured double bond. However, the relative configuration of 2-OH could not be determined by the ROESY data. The theoretical NMR calculations and DP4+ probability analyses were employed on two possible structures of (1S*,2R*,4S*,7R*)-**1a** and (1S*,2S*,4S*,7R*)-**1b**, and the calculations messages suggested that (1S*,2R*,4S*,7R*)-**1a** was the correct relative configuration for **1** (see Table S3 in Section S1). Finally, the absolute configuration of **1** was established to be 1*S*,2*R*,4*S*,7*R* by ECD calculations (Figure 5). Therefore, compound **1** was identified and named as craterodoratin A.

**Figure 4:**
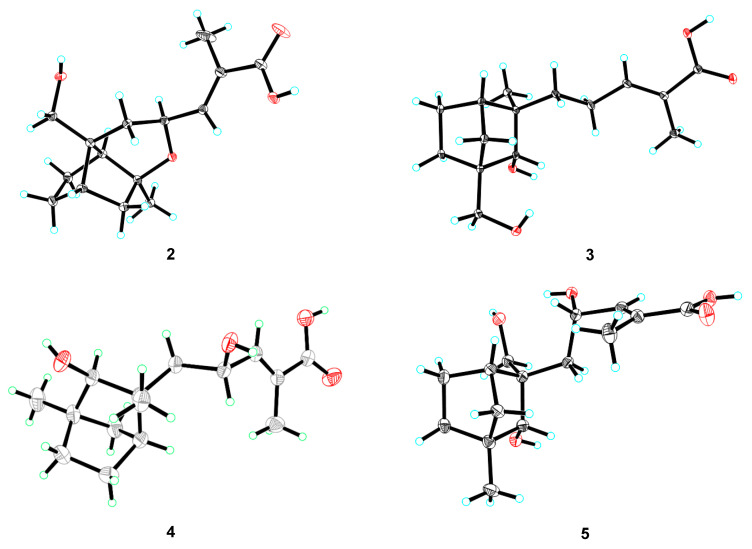
ORTEP diagrams of **2**–**5** showing absolute configurations.

Compound **2** was isolated as colorless crystals. Its molecular formula of C_15_H_22_O_4_ was determined by HRESIMS analysis, corresponding to five degrees of unsaturation. All the spectroscopic data indicated similar patterns to those of **1**, suggesting that **2** has a similar structure to **1**. Detailed analysis of 1D and 2D NMR data revealed the differences. Firstly, Me-15 was oxygenated into a hydroxymethylene in **2** as suggested by the HMBC correlations from *δ*_H_ 3.64 (1H, d, *J* = 11.0 Hz, H-15a) and *δ*_H_ 3.47 (1H, d, *J* = 11.0 Hz, H-15b) to *δ*_C_ 43.5 (C-1), 42.3 (C-4), 52.5 (C-7) and 33.7 (C-8) (Figure 2). Secondly, Me-14 shifted from C-1 to C-2 in **2** which supported by HMBC correlations from *δ*_H_ 1.25 (3H, s, Me-14) to *δ*_C_ 43.5 (C-1), 81.8 (C-2), and 48.2 (C-3). Thirdly, analysis of the MS data and carbon shifts at *δ*_C_ 81.8 (C-2) and *δ*_C_ 66.4 (C-9), as well as the HMBC from *δ*_H_ 4.64 (1H, m, H-9) to *δ*_C_ 81.8 (C-2) indicated an ether bond between C-2 and C-9. The ROESY (Figure 3) cross peaks of H_3_-14/H-5b, and H_3_-14/H-6b indicated Me-14, H-5b, and H-6b were on the same side, cross peaks of H_2_-15/H-6a, and H_2_-15/H-5a indicated CH_2_-15, H-5a, and H-6a were on the same side, while correlations between H-9/H_3_-13 and the absence of the ROESY correction of between H_3_-13/H-10 confirmed the *E*-configured double bond. Finally, the single-crystal X-ray diffraction established the structure of **2** with the absolute configuration (Flack parameter = 0.06 (7), CCDC: 2059695. Figure 4). Therefore, compound **2** was identified and named as craterodoratin B.

Compound **3** was isolated as colorless crystals. Its molecular formula of C_15_H_24_O_4_ was determined by HRESIMS data, corresponding to four degrees of unsaturation. The ^13^C NMR and DEPT data (Table 1) displayed 15 carbon resonances including two methyl carbons (*δ*_C_ 12.3 and 17.0), one olefinic methine carbons (*δ*_C_ 144.1), one oxygenated methine carbon (*δ*_C_ 79.7), six methylenes, one methine, two sp^3^ quaternary carbons (*δ*_C_ 55.8 and 43.2), and one carboxyl carbon (*δ*_C_ 172.0). Analysis of 2D NMR data including ^1^H-^1^H COSY and HMBC correlations as shown in Figure 2 suggested that **3** should be a bergamotane-type sesquiterpenoid related to (*Z*)-2*α*-hydroxyalbumol [24]. The differences were that C-12 and C-15 in **3** were oxidized into a carboxy group and a hydroxymethylene group, respectively. In the ROESY spectrum, cross peaks of H-7a and H-8 supported that C-7 and C-8 were on the same side. Besides, the double bond between C-10 and C-11 in **3** was assigned as *E* geometry by the ROESY correlation of H-9/H_3_-13. The single-crystal X-ray diffraction established the absolute configuration of **3** (Flack parameter = 0.06(6), CCDC: 2059696, Figure 4). Therefore, compound **3** was identified and named as craterodoratin C.

Compound **4** was isolated as colorless crystals. Its molecular formula of C_15_H_24_O_4_ was determined by HRESIMS data, the same as that of **3**. All the spectroscopic data showed similar patterns to those of **3**, except one oxygenated methylene group of C-15 was reduced to a methyl group in **4**. This was supported by the key HMBC correlations from *δ*_H_ 0.91 (3H, s, Me-15) to *δ*_C_ 41.5 (C-1), 83.5 (C-2), and 25.0 (C-6). In addition, C-9 was oxidized into a hydroxymethine group, as demonstrated by the HMBC correlations from *δ*_H_ 4.58 (1H, m, H-9) to *δ*_C_ 48.9 (C-8), 144.6 (C-10), and 126.6 (C-11). The single-crystal X-ray diffraction not only confirmed the planar structure as elucidated above but also established the absolute configuration (Flack parameter = 0.09(4), CCDC: 2059697, Figure 4). Therefore, compound **4** was identified and named as craterodoratin D.

Compound **5** was isolated as colorless crystals. Its molecular formula of C_15_H_24_O_5_ was determined by HRESIMS data, corresponding to four degrees of unsaturation. The 1D NMR data (Table 2) indicated that **5** has a closely related structure to that of **4**, except that one methyl group of C-14 was oxygenated into a hydroxyl methylene group in **5**. It was supported by the loss of one methyl signal in the ^1^H NMR spectrum and the HMBC correlations from *δ*_H_ 3.64 (1H, d, *J* = 11.7 Hz, H-14a) and *δ*_H_ 3.53 (1H, d, *J* = 11.7 Hz, H-14b) to *δ*_C_ 86.0 (C-2), 47.3 (C-3), 44.0 (C-4), and 47.0 (C-8). The single-crystal X-ray diffraction confirmed the planar structure and established the absolute configuration (Flack parameter = 0.26(11), CCDC: 2059698. Figure 4). Thus, compound **5** was identified and named as craterodoratin E. 

**Figure 5:**
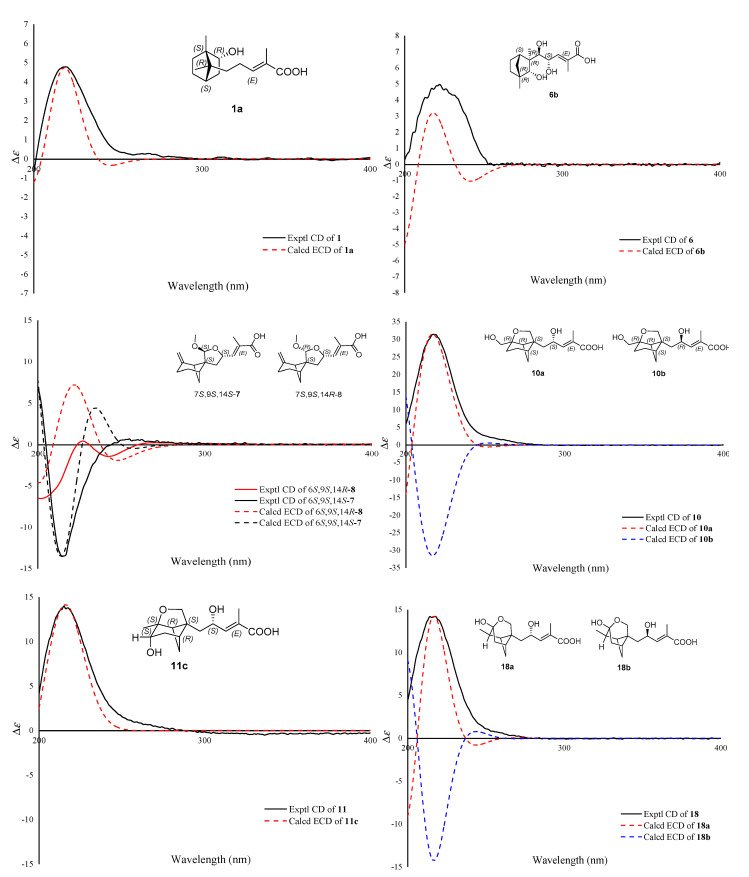
ECD calculations for compounds **1**, **6**, **7**, **8**, **10**, **11**, and **18**.

Compound **6** was isolated as a colorless oil. Its molecular formula of C_15_H_24_O_5_ was determined by HRESIMS data, corresponding to four degrees of unsaturation. All the spectroscopic data indicated that **6** had a similar structure to that of **4**. Detailed analysis of 1D and 2D NMR data revealed that C-8 was oxygenated into a hydroxymethine group in **6** as established by the shift at *δ*_C_ 80.5 (C-8) and MS data. The coupling constant (^3^*J*_H-H_ = 6.0 Hz) of H-8/H-9 of **6** observed in ^1^H NMR spectrum and a strong ROESY correlation between H-9 and H-2 suggested *anti* configuration of H-8 and H-9 [25]. To determine the stereochemistry of C-8, the theoretical NMR calculations and DP4 + probability analyses were employed on two possible relative structures (1R*,2R*,3R*,4S*,8S*,9S*)-**6a** and (1R*,2R*,3R*,4S*,8R*,9S*)-**6b**. The results suggested that (1R*,2R*,3R*,4S*,8R*,9S*)-**6b** was the correct relative configuration for **6** (see Table S6 in Section S2). Based on this, the absolute configuration of **6** was suggested to be 1*R*,2*R*,3*R*,4*S*,8*R*,9*S* by the ECD calculations (Figure 5). Therefore, compound **6** was identified and named as craterodoratin F.

Compounds **7** and **8** were isolated as a pair of epimers. They possessed the same molecular formula of C_16_H_22_O_4_, on the basis of HRESIMS data. Analysis of 1D (Table 1) and 2D NMR data suggested that the planar structures of **7** and **8** were similar to that of donacinoic acid A (**23**) [9], except the ether bond between C-2 and C-14 was cut off to give a methoxy group at C-14. In the ROESY spectra, the observed correlations of H-14/H-15a in **7** and H-5/H-14 in **8** suggested that the stereo-configurations of C-14 in **7** and **8** were different. The theoretical NMR calculations and DP4 + probability analyses were employed to elucidate the relative configurations of **7** and **8** (see Tables S9 and S10). Then, the ECD calculations established the absolute configurations to be 7*S*,9*S*,14*S* for **7** and 7*S*,9*S*,14*R* for **8** (Figure S9 in Section S3). Finally, compounds **7** and **8** were identified and named as craterodoratins G and H, respectively.

Compound **9** was isolated as a colorless oil. Its molecular formula of C_15_H_22_O_3_ was determined on the basis of the HRESIMS data, corresponding to five degrees of unsaturation. Analysis of its NMR data (Table 3) indicated similar patterns to those of massarinolin B [15], except that C-9 in massarinolin B was reduced to a methylene in **9** as determined by the MS data and ^1^H–^1^H COSY correlations (Figure 2). Therefore, compound **9** was identified and named as craterodoratin I.

Compound **10** was isolated as a colorless oil. Its molecular formula of C_15_H_22_O_5_ was determined by HRESIMS analysis, corresponding to five degrees of unsaturation. The ^1^H and ^13^C NMR (Table 3) spectroscopic characteristics were similar to those of **9**, except for two hydroxy groups placed at C-15 and C-9 in **10**. These were determined by the HMBC correlations from *δ*_H_ 4.45 (1H, td, *J* = 8.7, 3.8 Hz, H-9) to *δ*_C_ 40.2 (C-8), 143.6 (C-10), 127.3 (C-11), and from *δ*_H_ 3.49 (1H, d, *J* = 11.4 Hz, H-15a), 3.45 (1H, d, *J* = 11.4 Hz, H-15b) to *δ*_C_ 88.2 (C-2), 27.4 (C-3), 48.0 (C-1). According to the ROESY correlation between H-9 and H-13, the double bond was established as *E* form. The absolute configuration was established by ECD calculations as shown in Figure 5. Therefore, compound **10** was identified and named as craterodoratin J.

Compound **11** was isolated as a colorless oil. Its molecular formula of C_15_H_22_O_5_ was determined by HRESIMS analysis, corresponding to five degrees of unsaturation. The ^1^H and ^13^C NMR and DEPT (Table 3) data of **11** displayed signals for structural features similar to **10**, except that one hydroxy was transfered from C-15 to C-3. The location was determined by the HMBC correlations from *δ*_H_ 1.31 (3H, s, Me-15) to *δ*_C_ 87.3 (C-2), 72.2 (C-3), 51.3 (C-1), and from *δ*_H_ 3.63 (1H, d, *J* = 6.8 Hz, H-3) to *δ*_C_ 87.3 (C-2), 32.8 (C-4). The stereo-configurations for C-3 and C-9 could not be established according to the ROESY data. Thus, the theoretical NMR calculations and DP4 + probability analyses were employed on four possible structures (see Table S17 in Section S5). Based on these data, the absolute configuration of **11** was suggested to be 1*R*,2*S*,3*S*,5*R*,7*S*,9*S* by the ECD calculations (Figure 5). Therefore, compound **11** was identified and named as craterodoratin K.

Compound **12** was isolated as a colorless oil. Its molecular formula of C_15_H_18_O_5_ was determined by HRESIMS analysis, corresponding to seven degrees of unsaturation. The ^1^H and ^13^C NMR and DEPT (Table 3) data of **12** displayed signals for structural features similar to **9**, except that C-14 and C-9 were oxidized into two carbonyl carbons in **12**. The locations were determined by the HMBC correlations from *δ*_H_ 3.15 (2H, q, *J* = 3.2 Hz, H-8) to *δ*_C_ 40.2 (C-5), 53.6 (C-7), 179.3 (C-14), and 199.4 (C-9). Therefore, compound **12** was identified and named as craterodoratin L.

Compound **13** was isolated as a colorless oil. Its molecular formula of C_15_H_24_O_3_ was determined by HRESIMS analysis, corresponding to four degrees of unsaturation. The ^13^C NMR and DEPT spectrum (Table 4) displayed 15 carbon resonances including two methyl carbons, one olefinic methine carbon, one quaternary olefinic carbon, five methylenes, three methines, and one quaternary carbon. Analysis of 1D and 2D NMR data suggested that **13** had a similar structure to that of massarinolin C (**21**) [15]. The locations of 7-Me and 2-CH_2_OH were determined by the HMBC correlations from *δ*_H_ 0.89 (3H, s, Me-14) to *δ*_C_ 40.3 (C-1), 39.2 (C-5), 42.1 (C-7), 37.3 (C-8), and from *δ*_H_ 3.34 (2H, dd, *J* = 6.0, 2.6 Hz, H-15) to *δ*_C_ 40.3 (C-1), 37.2 (C-2), 18.0 (C-3). In the ROESY spectrum (Figure 3), the cross peak of H_3_-14/H-2 indicated that Me-14 and H-2 were on the same side, while the cross peak of H-9/H-13 indicated the *E* form of the double bond. Therefore, compound **13** was identified as craterodoratin M.

Compound **14** was isolated as a colorless oil. Its molecular formula of C_15_H_24_O_4_ was determined by HRESIMS analysis, corresponding to four degrees of unsaturation. All 1D and 2D NMR data suggested that **14** had a structure closely related to that of **13** except that one more hydroxy group at C-1 in **14**, which was supported by the HMBC correlations from *δ*_H_ 1.09 (3H, s, Me-14) to *δ*_C_ 73.5 (C-1), 47.3 (C-5), and 48.4 (C-7). Detailed analysis of 2D NMR data suggested that the other parts of **14** were the same as those of **13**. Therefore, compound **14** was identified and named as craterodoratin N.

Compound **15** was isolated as a colorless oil. Its molecular formula of C_15_H_22_O_3_ was determined by HRESIMS analysis, corresponding to five degrees of unsaturation. The ^1^H and ^13^C NMR and DEPT (Table 4) data of **15** displayed a close resemblance to those of **13**. One significant difference was that the hydroxy group was substituted at C-5, as evidenced by HMBCs from *δ*_H_ 2.33 (1H, m, H-6a) and 1.79 (1H, d, *J* = 9.5 Hz, H-6b) to *δ*_C_ 43.2 (C-1), 74.9 (C-5), and 48.6 (C-7). In addition, one terminal double bond was established between C-2 and C-15, as proved by HMBC correlations from *δ*_H_ 4.63 (2H, d, *J* = 2.0 Hz, H-15) to *δ*_C_ 43.2 (C-1), 149.4 (C-2), and 24.7 (C-3). Therefore, compound **15** was identified and named as craterodoratin O.

Compound **16** was isolated as a colorless oil. Its molecular formula of C_15_H_22_O_3_ was determined to be the same to that of **15** by HRESIMS data. Detailed analysis of 1D and 2D NMR data suggested that **16** had a very similar structure to that of **15** except for the hydroxy group at C-14 in **16** replaced at C-5 in **15**. It was supported by the HMBC correlations from *δ*_H_ 3.43 (1H, d, *J* = 11.7 Hz, H-14a) and *δ*_H_ 3.33 (1H, d, *J* = 11.7 Hz, H-14b) to *δ*_C_ 47.6 (C-1), 37.6 (C-5), 29.9 (C-8). Therefore, compound **16** was identified and named as craterodoratin P.

Compound **17** was isolated as a colorless oil. Its molecular formula of C_17_H_24_O_5_ was determined by HRESIMS analysis, corresponding to six degrees of unsaturation. The ^1^H and ^13^C NMR and DEPT (Table 5) data of **17** displayed a close resemblance to those of massarinolin C (**21**) [15]. Analysis of 2D NMR data suggested that one more *O*-acetyl group was substituted at C-14, as evidenced by HMBC correlations from *δ*_H_ 4.00 (1H, d, *J* = 11.9 Hz, H-14a) and 3.93 (1H, d, *J* = 11.9 Hz, H-14b) to *δ*_C_ 48.2 (C-1), 38.5 (C-5), 37.9 (C-8), 171.6 (-OO*C*CH_3_). The absolute configuration of **17** was established by ECD calculations as shown in Figure S18 in Section S6. Therefore, compound **17** was identified and named craterodoratin Q.

Compound **18** was isolated as a colorless oil. Its molecular formula of C_15_H_22_O_5_ was determined by HRESIMS data, corresponding to five degrees of unsaturation. The 1D NMR data (Table 5) indicated that **18** should have a closely related structure to that of brasilamide A [26]. One significant difference was that the amide group was replaced by a carboxy group at *δ*_C_ 172.6 (C-12), as evidenced by the MS data. In addition, C-9 and C-14 in **18** was reduced into a hydroxymethine group (*δ*_C_ 65.7) and a methylene (*δ*_C_ 69.3), respectively, as supported by the HMBC correlations from *δ*_H_ 4.45 (1H, m, H-9) to *δ*_C_ 39.5 (C-8) and 141.4 (C-10), and from *δ*_H_ 4.01 (1H, d, *J* = 10.2 Hz, H-14a) and 3.94 (1H, d, *J* = 10.2 Hz, H-14b) to *δ*_C_ 45.0 (C-1), 39.2 (C-7), 96.5 (C-3), and 37.7 (C-5). Based on the ROESY data, cross peaks of H-9/H-14 supported that H-9 and C-14 were on the same side; cross peaks of H-2/H-6b supported that H-2 and C-14 were on the same side. Finally, the absolute configuration of **18** was suggested to be 1*S*, 2*S*, 3*R*, 5*R*, 7*S*, 9*S* by the ECD calculations (Figure 5). Therefore, compound **18** was identified and named as craterodoratin R.

Compound **19** was isolated as a colorless oil. Its molecular formula of C_17_H_27_NO_2_ was determined by HRESIMS data, which suggested an *N*-containing structure. The 1D and 2D NMR data of **19** were similar to those of victoxinine [19,27], except that one oxygenated methylene carbon at C-17 in victoxinine was replaced by a carboxyl group in **19** as proved by HMBC correlation from *δ*_H_ 3.26 (2H, t, *J* = 6.0 Hz, H-16) to *δ*_C_ 174.0 (C-17). Detailed analysis of 2D NMR data suggested that the other parts of **19** were the same as that of victoxinine. Therefore, compound **19** was identified and named as craterodoratin S.

**Scheme 1:**
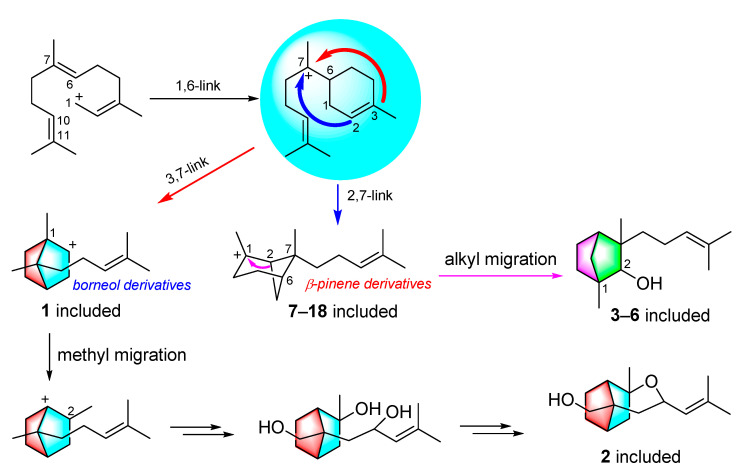
Proposed Biosynthetic Pathways for Bergamotane-type Sesquiterpenes.

In addition to the new compounds as described above, four known sesquiterpenoids obtained in this study were identified as massarinolin B (**20**) [15,28], massarinolin C (**21**) [15], massarinolin A (**22**) [15], and donacinoic acid A (**23**) [9] by comparison of their spectroscopic data with those reported in the literature. The biosynthesis of the isolated compounds **1**−**18** and **20**−**23** was proposed as shown in Scheme 1. Bergamotane sesquiterpenoids, bearing a borneol ring system or a *β*-pinene ring system, are naturally occurring in plants and fungi (Scheme 1) [9,14,15,16,17,29,30,31,32,33,34]. In this study, compounds **1** and **2** possess a rare carbon skeleton while the methyl group (C-14) in **2** had a further 1,2-migration. The sesquiterpenes **7**–**18** belong to *β*-pinene derivatives (Scheme 1), which produced compounds **3**–**6** by an alkyl migration.

All compounds were evaluated for their cytotoxicity to five human cancer cell lines (HL-60, A-549, SMMC-7721, SW480, MCF-7). However, no compounds are active at the concentration of 40 µM. In addition, all the compounds were investigated for their in vitro immunomodulatory effect on BALB/c mice T and B lymphocyte proliferation. Compounds **3**, **10**, **12**–**15**, **19**, **20**, and **23** exhibited potent inhibitory activity against LPS-induced proliferation of B lymphocyte cell with IC_50_ values ranging from 0.67 to 22.68 μM. Compounds **17** and **20** exhibited inhibitions on ConA-induced proliferation of T lymphocyte cells with IC_50_ values of 31.50 and 0.98 μM, respectively (Table 6).

Structurally, almost all bergamotane sesquiterpenoids in this study contain an *α*,*β*-unsaturated carboxylic acid moiety, which might be the key functional group for their immunosuppressive activity. However, it seems that the compounds (**10** and **12**–**15**) with a basic core of a *β*-pinene showed a wider range of biological activities. Immunosuppressants are a kind of drug that can inhibit human immunity. They are mainly used in organ transplantation to combat rejection and autoimmune diseases. At present, there are several immunosuppressive drugs that work by inhibiting T cell proliferation, but new, efficient, and safe immunosuppressive drugs inhibiting B cell proliferation are still unavailable [35,36]. In this study, many bergamotane sesquiterpenoids were found to have potential inhibition of B cell proliferation. To the best of our knowledge, bergamotane sesquiterpenoids were reported for their immunosuppressive activity for the first time.

## 4. Conclusions

In summary, a total of 23 sesquiterpenoids including 19 new ones were isolated from the edible fungus *C. ordoratus*. Of them, 22 compounds belong to bergamotane sesquiterpenoids in three carbon skeletons, while compounds **1** and **2** possess a rare ring-rearranged backbone. In addition, many bergamotane sesquiterpenoids exhibited selective inhibitions on LPS-induced B cell proliferation. This study suggests that *C. odoratus* is rich in bergamotane sesquiterpenoids with promising immunosuppressive activity and provides strong support for the further development and utilization of the edible mushroom *C. odoratus*.

## Figures and Tables

**Table 1:** ^1^H (600 MHz) and ^13^C (150 MHz) NMR Spectroscopic Data for **1**–**4** in Methanol-*d*_4_.

Entry	1		2		3		4	
	*δ*_C_, Type	*δ*_H_ (*J* in Hz)	*δ*_C_, Type	*δ*_H_ (*J* in Hz)	*δ*_C_, Type	*δ*_H_ (*J* in Hz)	*δ*_C_, Type	*δ*_H_ (*J* in Hz)
1	49.9, C	−	43.5, CH	2.25, d (4.3)	55.8, C	−	41.5, C	−
2	76.3, CH	4.02, d (9.1)	81.8, C	−	79.7, CH	3.51, s	83.5, CH	3.44, s
3a 3b	37.6, CH_2_	2.16, m 1.00, dd, (13.3, 3.3)	48.2, CH_2_	1.98, dd, (14.3, 4.3) 1.28, d, (13.7)	43.2, C	−	48.5, C	−
4	41.9, CH	1.79, m	42.3, CH	1.86, t, (4.3)	47.1, CH	1.83, m	46.5, CH	1.80, s
5a 5b	27.5, CH_2_	1.69, m 1.27, m	20.3, CH_2_	1.73, m 1.51, m	26.5, CH_2_	1.70, m 1.39, m	25.8, CH_2_	1.68, m 1.41, m
6a 6b	25.8, CH_2_	1.97, m 1.23, m	26.5, CH_2_	1.72, m 1.16, m	21.9, CH_2_	1.62, m 1.10, m	25.0, CH_2_	1.69, m 0.99, m
7a 7b	50.9, C	−	52.5, C	−	37.2, CH_2_	1.54, d, (10.1) 1.11, m	40.4, CH_2_	1.48, m 1.11, d, (10.3)
8a 8b	30.9, CH_2_	1.46, td, (12.8, 12.3, 5.1) 1.17, td, (12.8, 12.3, 5.1)	33.7, CH_2_	1.98, dd, (14.3, 4.3) 1.74, m	42.3, CH_2_	1.36, m	48.9, CH_2_	1.71, m 1.45, m
9a 9b	24.5, CH_2_	2.25, m 2.07, m	66.4, CH	4.64, m	24.7, CH_2_	2.15, m	65.3, CH	4.58, m
10	141.1, CH	6.69, t, (7.4)	141.0, CH	6.48, d, (7.8)	144.1, CH	6.72, t, (7.4)	144.6, CH	6.57, d, (9.28)
11	128.5, C	−	129.4, C	−	128.8, C	−	126.6, C	−
12	171.3, C	−	171.2, C	−	172.0, C	−	170.4, C	−
13	11.2, CH_3_	1.80, s	11.8, CH_3_	1.84, s	12.3, CH_3_	1.78, s	11.4, CH_3_	1.84, d, (1.5)
14	12.3, CH_3_	0.85, s	22.4, CH_3_	1.25, s	17.0, CH_3_	0.86, s	18.5, CH_3_	1.07, s
15a 15b	15.5, CH_3_	0.93, s	63.4, CH_2_	3.64, d, (11.0) 3.47, d, (11.0)	65.1, CH_2_	3.59, d, (11.0) 3.51, d, (11.0)	16.1, CH_3_	0.91, s

**Table 2:** ^1^H (600 MHz) and ^13^C (150 MHz) NMR Spectroscopic Data for **5**–**8** in Methanol-*d*_4_.

Entry	5		6		7		8	
	*δ_C_*, Type	*δ*_H_ (*J* in Hz)	*δ_C_*, Type	*δ*_H_ (*J* in Hz)	*δ_C_*, Type	*δ*_H_ (*J* in Hz)	*δ_C_*, Type	*δ*_H_ (*J* in Hz)
1	49.9, C	−	49.8, C	−	52.0, CH	2.64, t, (5.3, 10.8)	49.1, CH	2.79, t, (5.4)
2	86.0, CH	3.34, d (1.3)	82.2, CH	3.47, d (1.3)	150.0, C	−	151.0, C	−
3a 3b	47.3, C	−	47.8, C	−	23.1, CH_2_	2.61, m 2.33, m	24.3, CH_2_	2.65, m 2.40, m
4a 4b	44.0, CH	2.06, d, (3.8)	44.7, CH	2.23, d, (3.5)	23.5, CH_2_	2.11, m 1.82, m	24.7, CH_2_	1.96, m
5a 5b	27.0, CH_2_	1.71, m 1.47, m	27.6, CH_2_	1.65, m 1.39, m	39.4, CH	2.32, m	40.6, CH	2.27, m
6a 6b	26.7, CH_2_	1.72, m 0.99, m	26.7, CH_2_	1.66, m 0.99, m	26.5, CH_2_	2.20, m 1.47, d, (9.7)	29.1, CH_2_	2.28, m 1.42, m
7a 7b	41.9, CH_2_	1.38, d, (10.6) 1.13, d, (10.6)	41.5, CH_2_	1.47, d, (9.7) 1.07, d, (9.7)	54.7, C	−	56.5, C	−
8a 8b	47.0, CH_2_	1.70, m 1.53, dd, (2.6, 14.8)	80.5, CH	3.39, d, (6.0)	40.4, CH_2_	2.50, dd, (12.4, 9.7) 2.02, dd, (12.4, 4.2)	39.4, CH_2_	2.46, dd, (6.7, 11.9) 1.92, dd, (9.5, 11.9)
9	66.6, CH	4.60, m	70.6, CH	4.38, dd, (9.1, 6.0)	73.7, CH	4.91, m	75.7, CH	4.90, s
10	142.6, CH	6.49, d (8.2)	138.4, CH	6.53, d (8.6)	143.0, CH	6.74, dd, (1.5, 8.0)	144.2, CH	6.65, d, (8.2)
11	131.5, C	−	135.7, C	−	128.1, C	−	130.2, C	−
12	174.9, C	−	176.1, C	−	169.8, C	−	171.3, C	−
13	13.1, CH_3_	1.80, s	14.3, CH_3_	1.86, s	11.4, CH_3_	1.85, d, (1.5)	12.8, CH_3_	1.85, s
14a 14b	64.0, CH_2_	3.64, d, (11.7) 3.53, d, (11.7)	19.9, CH_3_	1.04, s	106.4, CH	4.59, s	106.8, CH	4.57, s
15a 15b	19.4, CH_3_	1.03, s	12.2, CH_3_	0.81, s	107.0, CH_2_	4.74, q, (1.8) 4.68, dt, (3.0, 1.5)	107.8, CH_2_	4.66, s 4.63, s
OCH_3_	−	−	−	−	53.3, CH_3_	3.29, s	55.4, CH_3_	3.28, s

**Table 3:** ^1^H (600 MHz) and ^13^C (150 MHz) NMR Spectroscopic Data for **9**–**12** in Methanol-*d*_4_.

Entry	9		10		11		12	
	*δ_C_*, Type	*δ*_H_ (*J* in Hz)	*δ*_C_, Type	*δ*_H_ (*J* in Hz)	*δ*_C_, Type	*δ*_H_ (*J* in Hz)	*δ*_C_, Type	*δ*_H_ (*J* in Hz)
1	51.0, CH	2.10, t, (5.4)	48.0, CH	2.29, t, (5.6)	51.3, CH	2.09, t (5.7)	47.4, CH	2.70, t, (5.3)
2	87.4, C	−	88.2, C	−	87.3, C	−	89.2, C	−
3a 3b	32.0, CH_2_	1.88, m 1.61, m	27.4, CH_2_	1.92, m 1.56, td, (10.2, 4.2)	72.2, CH	3.63, d, (6.8)	29.5, CH_2_	2.06, m 1.83, m
4a 4b	22.4, CH_2_	1.88, m 1.76, m	22.0, CH_2_	1.91, m 1.82, m	32.8, CH_2_	2.25, m 1.85, m	22.4, CH_2_	1.95, m 1.81, m
5	39.2, CH	2.24, q, (5.4)	39.2, CH	2.32, m	37.8, CH	2.27, m	40.2, CH	2.43, q, (5.3)
6a 6b	22.2, CH_2_	2.13, m 1.50, d, (10.1)	22.3, CH_2_	2.14, m 1.51, d, (10.2)	21.7, CH_2_	2.11, m 2.05, m	22.1, CH_2_	2.28, m 1.75, d, (10.9)
7	55.3, C	−	53.7, C	−	54.7, C	−	53.6, C	−
8a 8b	32.4, CH_2_	1.91, m 1.72, m	40.2, CH_2_	2.01, dd, (14.2, 4.2) 1.84, m	40.1, CH_2_	2.00, dd, (14.2, 4.0) 1.81, dd, (14.2, 4.0)	43.4, CH_2_	3.15, q, (3.2)
9a 9b	24.5, CH_2_	2.19, m 2.10, m	65.7, CH	4.45, td, (8.7, 3.8)	65.8, CH	4.42, td, (9.0, 4.0)	199.4, C	−
10	141.7, CH	6.77, t, (7.5)	143.6, CH	6.67, d, (7.7)	141.7, CH	6.57, d, (9.0)	130.6, CH	7.11, s
11	127.9, C	−	127.3, C	−	129.3, C	−	142.4, C	−
12	170.4, C	−	170.5, C	−	172.2, C	−	169.9, C	−
13	11.0, CH_3_	1.80, s	11.4, CH_3_	1.83, s	11.8, CH_3_	1.83, s	13.4, CH_3_	2.15, s
14a 14b	70.2, CH_2_	3.82, d, (8.8) 3.47, d, (8.8)	71.7, CH_2_	3.94, d, (9.4) 3.63, d, (9.4)	71.5, CH_2_	3.85, d, (9.4) 3.61, d, (9.4)	179.3, C	−
15a 15b	24.0, CH_3_	1.24, s	65.9, CH_2_	3.49, d, (11.4) 3.45, d, (11.4)	21.0, CH_3_	1.31, s	23.6, CH_3_	1.51, s

**Table 4:** ^1^H (600 MHz) and ^13^C (150 MHz) NMR Spectroscopic Data for **13**–**16** in Methanol-*d*_4_.

Entry	13		14		15		16	
	*δ_C_*, Type	*δ*_H_ (*J* in Hz)	*δ_C_*, Type	*δ*_H_ (*J* in Hz)	*δ_C_*, Type	*δ*_H_ (*J* in Hz)	*δ_C_*, Type	*δ*_H_ (*J* in Hz)
1	40.3, CH	2.04, m	73.5, C	−	43.2, CH	2.48, d, (6.7)	47.6, CH	1.87, m
2	39.2, CH	1.96, q, (5.1)	40.3, CH	1.38, m	149.4, C	−	150.2, C	−
3a 3b	37.3, CH_2_	1.84, m 1.76, m	29.6, CH_2_	1.86, m 0.93, m	24.7, CH_2_	2.64, m 2.32, m	23.5, CH_2_	2.63, m 2.33, m
4a 4b	23.9, CH_2_	3.16, m	26.7, CH_2_	1.79, m 1.08, m	30.2, CH_2_	1.95, m 1.75, m	22.6, CH_2_	1.95, m 1.88, m
5	37.3, CH	2.13, m	47.3, CH	1.33, m	74.9, C	−	37.6, CH	2.18, q, (5.4)
6a 6b	18.0, CH_2_	1.63, m 1.27, m	25.9, CH_2_	1.92, m 1.12, dd, (3.7, 12.5)	34.6, CH_2_	2.33, m 1.79, d, (9.5)	25.8, CH_2_	2.33, m 1.47, d, (10.1)
7	42.1, C	−	48.4, C	−	48.6, C	−	46.7, C	−
8a 8b	22.9, CH_2_	2.01, d, (5.7) 1.37, m	38.1, CH_2_	1.53, t, (1.5)	32.3, CH_2_	1.82, m 1.68, m	29.9, CH_2_	1.87, m
9a 9b	23.7, CH	1.84, m	22.3, CH_2_	2.21, m	23.8, CH_2_	2.28, m 2.21, m	23.5, CH_2_	2.33, m 2.23, q, (8.0)
10	140.7, CH	6.71, dd, (7.4, 14.5)	138.9, CH	6.60, t, (6.6)	142.5, CH	6.81, t, (7.0)	142.8, CH	6.84, t, (8.0)
11	129.3, C	−	130.8, C	−	127.8, C	−	127.5, C	−
12	171.3, C	−	174.5, C	−	171.3, C	−	170.8, C	−
13	11.4, CH_3_	1.81, s	11.8, CH_3_	1.81, s	11.1, CH_3_	1.82, s	11.0, CH_3_	1.83, s
14a 14b	15.8, CH_3_	0.89, s	22.0, CH_3_	1.09, s	15.6, CH_3_	0.81, s	61.1, CH_2_	3.43, d, (11.7) 3.33, d, (11.7)
15a 15b	65.3, CH_2_	3.34, dd, (2.6, 6.0)	67.3, CH_2_	3.34, d, (3.3)	106.1, CH_2_	4.63, d, (2.0)	105.9, CH_2_	4.64, br s 4.60, br s

**Table 5:** ^1^H (600 MHz) and ^13^C (150 MHz) NMR Spectroscopic Data for **17**–**19** in Methanol-*d*_4_.

Entry	17		18		19	
	*δ*_C_, Type	*δ*_H_ (*J* in Hz)	*δ*_C_, Type	*δ*_H_ (*J* in Hz)	*δ*_C_, Type	*δ*_H_ (*J* in Hz)
1	48.2, CH	3.00, t, (5.3, 10.6)	45.0, CH	2.03, t, (6.2)	40.8, CH	2.31, br s
2	149.7, C	−	44.2, CH	1.94, q, (6.9)	42.2, CH	2.82, s
3a 3b	23.2, CH_2_	2.64, m 2.34, m	96.5, C	−	159.1, C	−
4a 4b	22.6, CH_2_	1.90, m	41.0, CH_2_	2.24, m 1.87, d, (12.4)	58.7, CH_2_	3.51, m 3.08, dd, (10.6, 2.1)
5	38.5, CH	2.21, m	37.7, CH_3_	2.33, q, (6.2)	58.8, CH_2_	3.51, m
6a 6b	44.1, C	−	35.6, CH_2_	2.49, m 1.16, d, (9.7)	58.7, CH	1.99, br s
7a 7b	25.5, CH_2_	2.43, m 1.51, d, (10.0)	39.2, C	−	48.4, C	−
8a 8b	37.9, CH_2_	2.17, dd, (8.7, 15.0) 1.81, m	39.5, CH_2_	1.85, m 1.71, dd, (14.8, 4.4)	40.8, CH_2_	1.41, m 1.33, m
9a 9b	65.7, CH	4.60, m	65.7, CH	4.45, m	24.8, CH_2_	1.63, m 1.08, m
10	143.1, CH	6.65, d, (8.4)	141.4, CH	6.52, d, (8.2)	44.7, CH	1.08, m
11	127.8, C	−	129.7, C	−	30.9, CH	1.35, m
12	171.6, C	−	172.6, C	−	20.2, CH_3_	0.87, d, (6.5)
13	11.6, CH_3_	1.83, s	12.0, CH_3_	1.85, s	20.0, CH_3_	0.81, d, (6.5)
14a 14b	64.8, CH_2_	4.00, d, (11.9) 3.93, d, (11.9)	69.3, CH_2_	4.01, d, (10.2) 3.94, d, (10.2)	103.4, CH_2_	5.02, s 4.74, s
15a 15b	106.7, CH_2_	4.67, br s 4.64, br s	13.8, CH_3_	1.06, d, (6.9)	20.1, CH_3_	0.98, s
-OOCCH_3_	19.3, CH_3_	2.00, s	45.0, CH	2.03, t, (6.2)	48.2, CH_2_	3.26, t, (6.0)
-OO*C*CH_3_	171.6, C	−	44.2, CH	1.94, q, (6.9)	174.0, C	−

**Table 6:** Immunosuppressive Tests of the Isolates.

Entry	CC_50_ (μM)	Con A-Induced T-Cell Proliferation		LPS-Induced B-Cell Proliferation	
		IC_50_ (μM)	SI *^a^*	IC_50_ (μM)	SI *^a^*
3	>40	−	−	12.62 ± 1.14	>3.17
10	>40	−	−	19.40 ± 0.48	>2.06
12	>40	−	−	13.71 ± 0.65	>2.92
13	>40	−	−	15.43 ± 1.03	>2.59
14	>40	−	−	13.26 ± 1.29	>3.02
15	>40	−	−	17.12 ± 1.14	>2.34
17	>40	31.50 ± 1.79	>1.27	−	−
19	>40	−	−	22.68 ± 1.67	>1.76
20	>40	0.98 ± 0.01	>40.82	0.67 ± 0.004	>59.26
23	>40	−	−	13.23 ± 0.97	>3.02
CsA	>2.80	0.04	>70.00	0.47	>5.95

*^a^* SI (selectivity index) is determined as the ratio of the concentration of the compounds that reduced cell viability to 50% (CC_50_) to the concentration of the compounds needed to inhibit the proliferation by 50% relative to the control value (IC_50_).

## Data Availability

X-ray crystallographic data of **2**–**5** (CIF) is available free of charge at https://www.ccdc.cam.ac.uk.

## Supplementary Materials

The following are available online at https://www.mdpi.com/article/10.3390/jof7121052/s1, Section S1: Calculational details for **1**, Section S2: Calculational details for **6**, Section S3: Calculational details for 6*S*,9*S*,14*S*-**7** and 6*S*,9*S*,14*R*-**8**, Section S4: Calculational details for **10**, Section S5: Calculational details for **11**, Section S6: Calculational details for **17**,Section S7: Calculational details for **18**, Section S8: NMR and MS spectra for **1**, Section S9: NMR and MS spectra for **2**, Section S10: NMR and MS spectra for **3**, Section S11: NMR and MS spectra for **4**, Section S12: NMR and MS spectra for **5**, Section S13: NMR and MS spectra for **6**, Section S14: NMR and MS spectra for 6*S*,9*S*,14*S*-**7**, Section S15: NMR and MS spectra for 6*S*,9*S*,14*R*-**8**, Section S16: NMR and MS spectra for **9**, Section S17: NMR and MS spectra for **10**, Section S18: NMR and MS spectra for **11**, Section S19: NMR and MS spectra for **12**, Section S20: NMR and MS spectra for **13**, Section S21: NMR and MS spectra for **14**, Section S22: NMR and MS spectra for **15**, Section S23: NMR and MS spectra for **16**, Section S24: NMR and MS spectra for **17**, Section S25: NMR and MS spectra for **18**, and Section S26: NMR and MS spectra for **19**.
