# Supplementary material for: Immunosuppressive Sesquiterpenoids from the Edible Mushroom Craterellus odoratus
Source: J Fungi (Basel). 2021 Dec 8;7(12):1052. doi: 10.3390/jof7121052 (PMC8707212; doi:10.3390/jof7121052)

Supporting Information for

Sesquiterpenoids from Edible Mushroom *Craterellus odoratus* and Their Immunosuppressive Activity

Quan Dai, Fa-Lei Zhang, Zheng-Hui Li, Juan He* and Tao Feng*

School of Pharmaceutical Sciences, National Demonstration Center for Experimental Ethnopharmacology Education, South-Central University for Nationalities, Wuhan 430074, People's Republic of China

*Corresponding author: tfeng@mail.scuec.edu.cn (T. Feng); 2015048@mail.scuec.edu.cn (J. He)

Contents

Section S1. Calculational details for 1 .... S4

S1.1. Computational details for 1 (NMR) S4
S1.2. Computational details for 1 (ECD) S10

Section S2. Calculational details for 6 .... S11

S2.1. Computational details for 6 (NMR) S11
S2.2. Computational details for 6 (ECD) S15

Section S3. Calculational details for 6*S*,9*S*,14*S*-7 and 6*S*,9*S*,14*R*-8 .... S16

S3.1. Computational details for 6*S*,9*S*,14*S*-7 and 6*S*,9*S*,14*R*-8 (NMR) S16
S3.2. Computational details for 6*S*,9*S*,14*R*-8 and 6*S*,9*S*,14*S*-7 (ECD) S20

Section S4. Calculational details for 10 .... S21

S4.1. Computational details for 10 (ECD) S21

Section S5. Calculational details for 11 .... S24

S5.1. Computational details for 11 (NMR) S24
S5.2. Computational details for 11 (ECD) S30

Section S6. Calculational details for 17 .... S31

S6.1. Computational details for 17 (ECD) S31

Section S7. Calculational details for 18 .... S35

S7.1. Computational details for 18 (ECD) S35

References S39

Section S8. NMR and MS spectra for 1 .... S40

Section S9. NMR and MS spectra for 2 .... S47

Section S10. NMR and MS spectra for 3 .... S54

Section S11. NMR and MS spectra for 4 .... S61

Section S12. NMR and MS spectra for 5 .... S68

Section S13. NMR and MS spectra for 6 .... S75

Section S14. NMR and MS spectra for 6*S*,9*S*,14*S*-7 .... S82

Section S15. NMR and MS spectra for 6*S*,9*S*,14*R*-8 .... S89

Section S16. NMR and MS spectra for 9 .... S96

Section S17. NMR and MS spectra for 10 .... S103

Section S18. NMR and MS spectra for 11 .... S110

Section S19. NMR and MS spectra for 12 .... S117

Section S20. NMR and MS spectra for 13 .... S124

Section S21. NMR and MS spectra for 14 .... S131

Section S22. NMR and MS spectra for 15 .... S138

Section S23. NMR and MS spectra for 16 .... S145

Section S24. NMR and MS spectra for 17 .... S152

Section S25. NMR and MS spectra for 18 .... S159

Section S26. NMR and MS spectra for 19 .... S166

Section S1. Calculational details for 1

S1.1. Computational details for 1 (NMR)

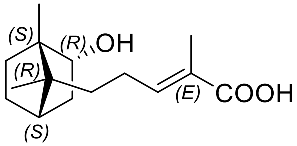

1a

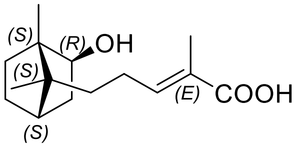

1b

Conformation search based on molecular mechanics with MMFF force fields were performed for **1a** and **1b** gave 15 and 16 stable conformers with populations higher than 1%, respectively.[1,2] All these conformers were further optimized by the density functional theory method at the B3LYP/6-31G(d) level by Gaussian 16 program package.[3] Gauge Independent Atomic Orbital (GIAO) calculations of their ^{1}H and ^{13}C NMR chemical shifts using density functional theory (DFT) at the mPW1PW91/6-311+G(d,p) level with the PCM model in methanol. The calculated NMR data of these conformers were averaged according to the Boltzmann distribution theory and their relative Gibbs free energy. The ^{1}H and ^{13}C NMR chemical shifts for TMS were also calculated by the same procedures and used as the reference. After calculation, the experimental and calculated data were evaluated by the improved probability DP4+ method.[4]

Table S1. Energy analysis for conformers of **1aA–1aO** at B3LYP/6-31G(d) level in the gas phase

Species	*E'=E+ZPE*	*E*	*H*	*G*	*ΔG*	*ΔE*(kcal/mol)	*PE*%
**1aA**	-811.346396	-811.327397	-811.326453	-811.395115	0	0	19.86%
**1aB**	-811.345763	-811.326685	-811.325741	-811.395024	0.000091	0.057103	18.03%
**1aC**	-811.346155	-811.327270	-811.326326	-811.394244	0.000871	0.546561	7.89%
**1aD**	-811.345542	-811.326569	-811.325625	-811.393639	0.001476	0.926204	4.16%
**1aE**	-811.346505	-811.327616	-811.326672	-811.394869	0.000246	0.154367	15.30%
**1aF**	-811.345807	-811.326871	-811.325927	-811.394120	0.000995	0.624372	6.92%
**1aG**	-811.345696	-811.326792	-811.325848	-811.393802	0.001313	0.823920	4.94%
**1aH**	-811.346282	-811.327479	-811.326535	-811.394301	0.000814	0.510793	8.38%
**1aI**	-811.343685	-811.324821	-811.323877	-811.391425	0.003690	2.315510	0.40%
**1aJ**	-811.344259	-811.325515	-811.324570	-811.391727	0.003388	2.126002	0.55%
**1aK**	-811.345655	-811.326666	-811.325721	-811.394425	0.000690	0.432982	9.56%

**1aL**	-811.342392	-811.323667	-811.322723	-811.389609	0.005506	3.455067	0.06%
**1aM**	-811.345470	-811.326570	-811.325626	-811.393508	0.001607	1.008408	3.62%
**1aN**	-811.343690	-811.324969	-811.324025	-811.390977	0.004138	2.596634	0.25%
**1aO**	-811.343007	-811.324371	-811.323427	-811.390108	0.005007	3.141940	0.10%

E, *E*′, *H*, *G*: total energy, total energy with zero point energy (*ZPE*), enthalpy, and Gibbs free energy

Figure S1. Main conformers of **1a** in NMR and ECD calculations.

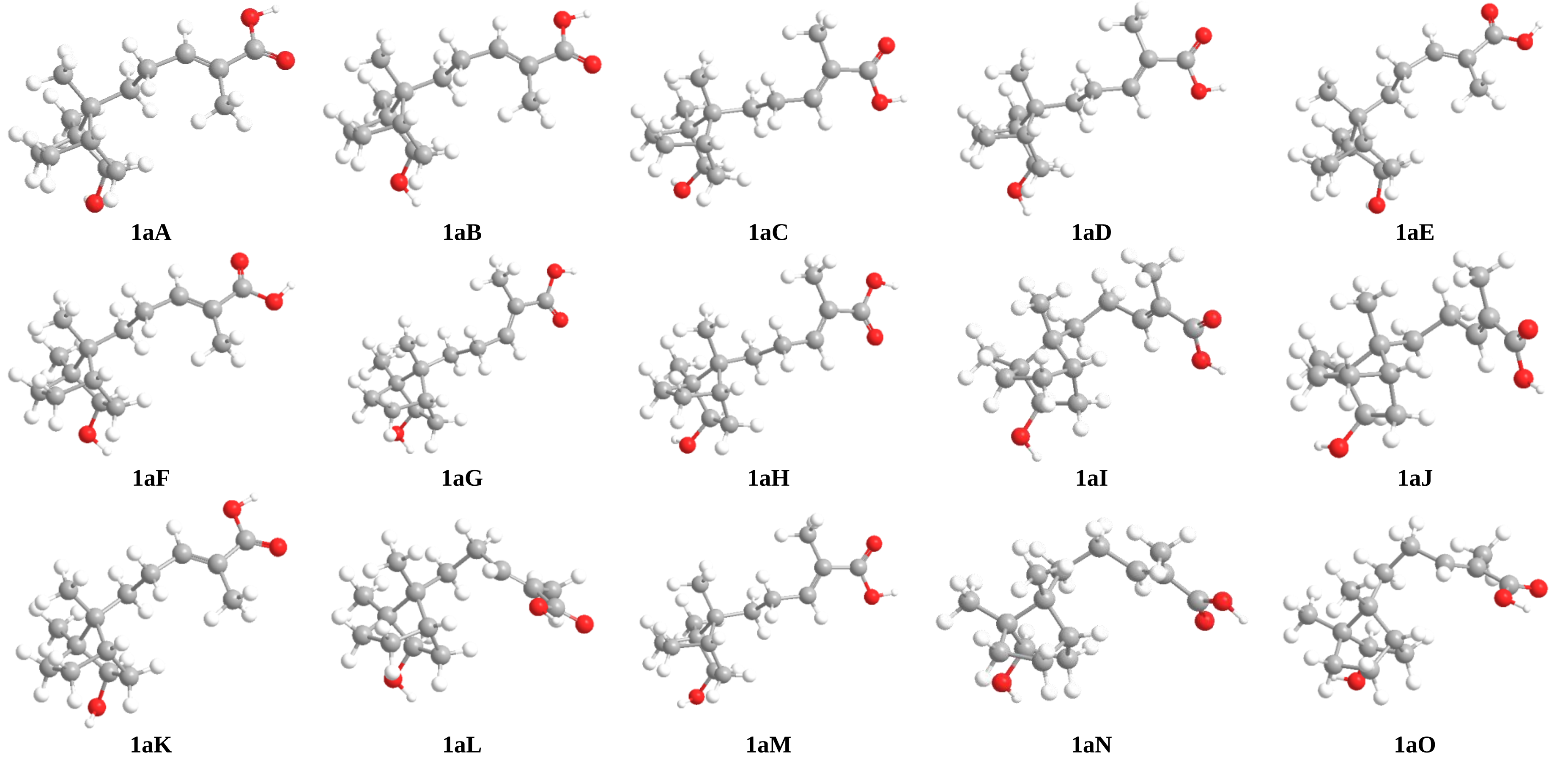

Table S2. Energy analysis for conformers of **1bA–1bP** at B3LYP/6-31G(d) level in the gas phase

Species	*E'*=*E*+*ZPE*	*E*	*H*	*G*	*ΔG*	*ΔE*(kcal/mol)	*PE*%
**1bA**	-811.345766	-811.326731	-811.325786	-811.394434	0.000619	0.388428	14.06%
**1bB**	-811.346763	-811.327870	-811.326926	-811.395053	0	0	27.10%
**1bC**	-811.345556	-811.326611	-811.325667	-811.393516	0.001537	0.964482	5.32%
**1bD**	-811.346640	-811.327785	-811.326841	-811.394428	0.000625	0.392193	13.97%
**1bE**	-811.346793	-811.328048	-811.327104	-811.394436	0.000617	0.387173	14.09%
**1bF**	-811.345728	-811.326874	-811.325929	-811.393457	0.001596	1.001505	4.99%
**1bG**	-811.345653	-811.326791	-811.325847	-811.393483	0.001570	0.985190	5.13%
**1bH**	-811.343373	-811.324551	-811.323607	-811.390913	0.004140	2.597889	0.34%
**1bI**	-811.342002	-811.323287	-811.322343	-811.389135	0.005918	3.713601	0.05%
**1bJ**	-811.346722	-811.327950	-811.327006	-811.394361	0.000692	0.434237	13.02%
**1bK**	-811.344366	-811.325637	-811.324693	-811.391957	0.003096	1.942769	1.02%
**1bL**	-811.343380	-811.324693	-811.323749	-811.390469	0.004584	2.876504	0.21%
**1bM**	-811.343062	-811.324413	-811.323469	-811.390130	0.004923	3.089229	0.15%
**1bN**	-811.343313	-811.324423	-811.323479	-811.390889	0.004164	2.612950	0.33%
**1bO**	-811.342109	-811.323443	-811.322498	-811.389226	0.005827	3.656498	0.06%
**1bP**	-811.343167	-811.324573	-811.323629	-811.390197	0.004856	3.047186	0.16%

E, *E'*, *H*, *G*: total energy, total energy with zero point energy (*ZPE*), enthalpy, and Gibbs free energy

Figure S2. Main conformers of **1b** in NMR calculation.

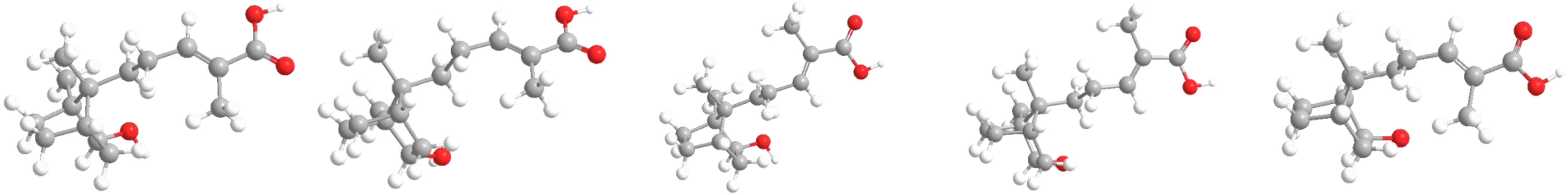

1bA **1bB** **1bC** **1bD** **1bE**

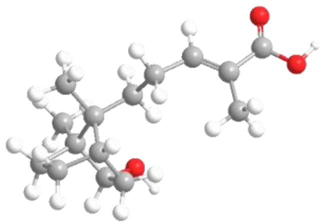

1bF

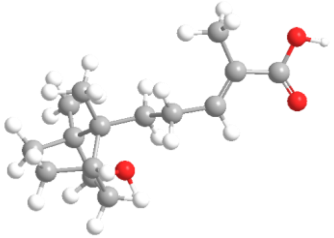

1bG

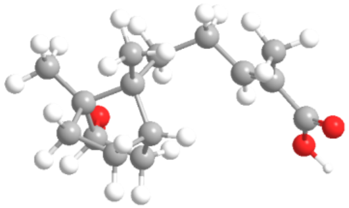

1bH

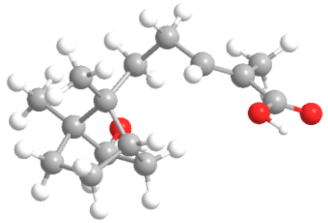

1bI

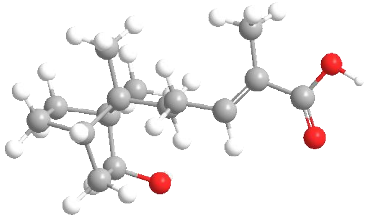

1bJ

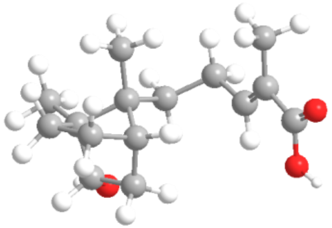

1bK

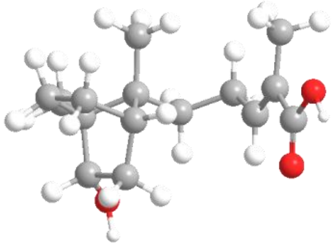

1bL

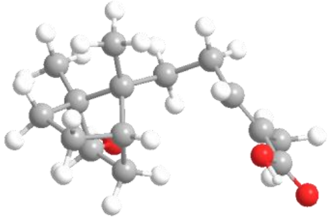

1bM

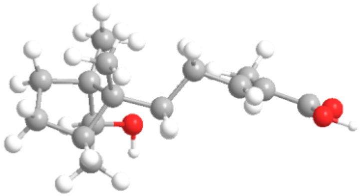

1bN

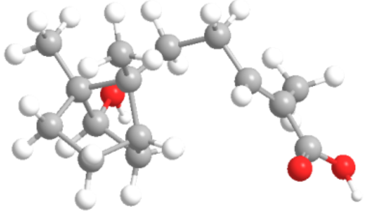

1bO

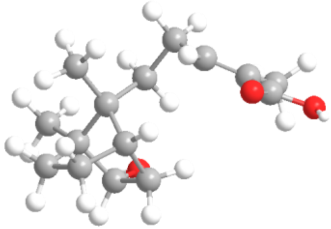

1bP

Table S3. DP4+ analysis results of **1a** (Isomer 1) and **1b** (Isomer 2) with **1**

	A	B	C	D	E	F	G	H
1	Functional		Solvent?		Basis Set		Type of Data	
2	mPW1PW91		PCM		6-311+G(d,p)		Shielding Tensors	
3								
12			DP4+	100.00%	0.00%	–	–	–
14	Nuclei	sp2?	xperimenta	Isomer 1	Isomer 2	Isomer 3	Isomer 4	Isomer 5
15	C		49.9	131.3	131.5			
16	C		76.3	106.4	103.1			
17	C		37.6	146.4	145.3			
18	C		41.9	140.7	141.4			
19	C		27.5	156.6	157.3			
20	C		25.8	158.9	150.9			
21	C		50.9	130.1	132.2			
22	C		30.9	153.2	153.3			
23	C		24.5	157.6	158.2			
24	C	x	141.1	29.1	28.4			
25	C	x	128.5	55.5	56.0			
26	C	x	171.3	11.6	11.5			
27	C		11.2	173.4	173.5			
28	C		15.5	169.8	170.0			
29	C		12.3	173.4	175.6			
30								
31	H		4.02	27.67	28.06			
32	H		2.16	29.53	30.00			
33	H		1	30.81	30.13			
34	H		1.79	29.95	29.93			
35	H		1.27	30.05	30.09			
36	H		1.69	30.49	30.70			
37	H		1.23	29.67	30.85			
38	H		1.97	30.47	30.21			
39	H		1.46	30.48	29.21			
40	H		1.17	30.58	30.81			
41	H		2.07	29.42	29.43			
42	H		2.25	29.55	29.49			
43	H	x	6.69	24.30	24.29			
44	H		1.8	29.69	29.65			
45	H		1.8	30.02	30.02			
46	H		1.8	30.05	30.04			
47	H		0.93	31.11	31.10			
48	H		0.93	30.65	30.90			
49	H		0.93	30.64	30.53			
50	H		0.85	30.96	30.67			
51	H		0.85	30.74	30.98			
52	H		0.85	30.99	30.92			

	A	B	C	D	E	F	G	H
1	Functional		Solvent?		Basis Set		Type of Data	
2	mPW1PW91		PCM		6-311+G(d,p)		Shielding Tensors	
3								
4			Isomer 1	Isomer 2	Isomer 3	Isomer 4	Isomer 5	Isomer 6
5	sDP4+ (H data)		100.00%	0.00%	-	-	-	-
6	sDP4+ (C data)		99.96%	0.04%	-	-	-	-
7	sDP4+ (all data)		100.00%	0.00%	-	-	-	-
8	uDP4+ (H data)		99.99%	0.01%	-	-	-	-
9	uDP4+ (C data)		99.99%	0.01%	-	-	-	-
10	uDP4+ (all data)		100.00%	0.00%	-	-	-	-
11	DP4+ (H data)		100.00%	0.00%	-	-	-	-
12	DP4+ (C data)		100.00%	0.00%	-	-	-	-
13	DP4+ (all data)		100.00%	0.00%	-	-	-	-

S1.2. Computational details for 1 (ECD)

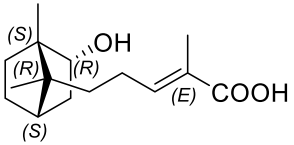

1a

Conformation search based on molecular mechanics with MMFF force fields were performed for **1a** gave 15 stable conformers with populations higher than 1%. All these conformers were further optimized by the density functional theory method at the B3LYP/6-31G(d) level by Gaussian 16 program package. The ECD were calculated using density functional theory (TDDFT) at B3LYP/6-31+G(d) level in methanol with IEFPCM model. The calculated ECD curves were all generated using SpecDis 1.71 with σ = 0.30 eV, UV shift -10 nm.[5]

Figure S3. Comparison of the calculated ECD spectra for possible structure with the experimental spectrum of **1** in methanol with PCM model.

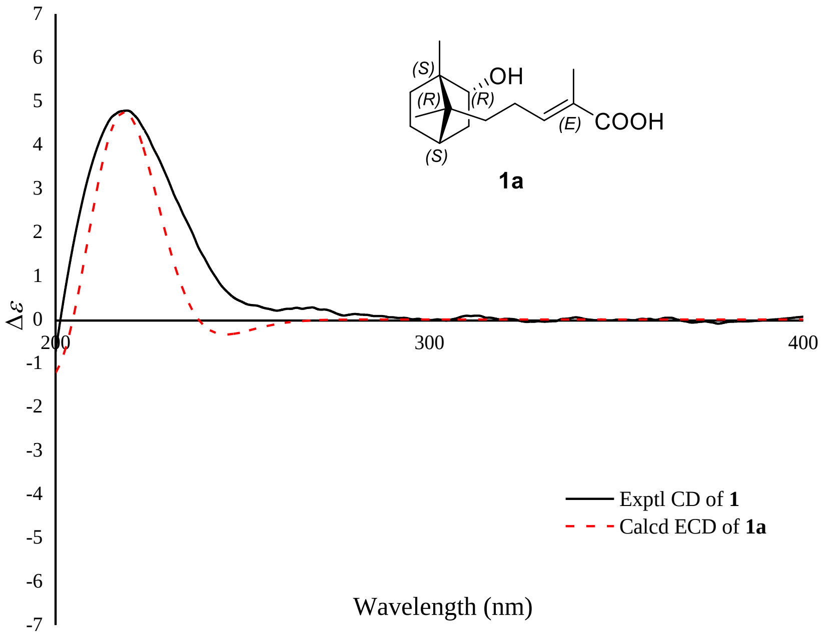

Section S2. Calculational details for 6

S2.1. Computational details for 6 (NMR)

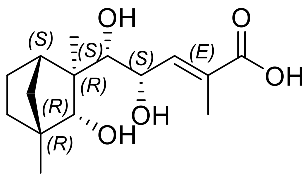

6a

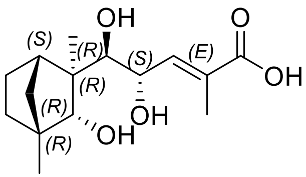

6b

Conformation search based on molecular mechanics with MMFF force fields were performed for **6a** and **6b** gave 14 and 6 stable conformers with populations higher than 1%, respectively. All these conformers were further optimized by the density functional theory method at the B3LYP/6-31G(d) level by Gaussian 16 program package. Gauge Independent Atomic Orbital (GIAO) calculations of their ^{1}H and ^{13}C NMR chemical shifts using density functional theory (DFT) at the mPW1PW91/6-311+G(d,p) level with the PCM model in methanol. The calculated NMR data of these conformers were averaged according to the Boltzmann distribution theory and their relative Gibbs free energy. The ^{1}H and ^{13}C NMR chemical shifts for TMS were also calculated by the same procedures and used as the reference. After calculation, the experimental and calculated data were evaluated by the improved probability DP4+ method.

Table S4. Energy analysis for conformers of **6aA–6aN** at B3LYP/6-31G(d) level in the gas phase

Species	*E′*=*E*+*ZPE*	*E*	*H*	*G*	*ΔG*	*ΔE*(kcal/mol)	*PE*%
**6aA**	-961.762350	-961.741395	-961.740451	-961.811097	0.002207	1.384913	3.09%
**6aB**	-961.762329	-961.741476	-961.740532	-961.810880	0.002424	1.521083	2.45%
**6aC**	-961.763374	-961.742292	-961.741348	-961.813304	0	0	32.01%
**6aD**	-961.763197	-961.742209	-961.741264	-961.812730	0.000574	0.360190	17.42%
**6aE**	-961.763360	-961.742398	-961.741453	-961.812880	0.000424	0.266064	20.42%
**6aF**	-961.762085	-961.741187	-961.740243	-961.810939	0.002365	1.484060	2.61%
**6aG**	-961.761002	-961.740193	-961.739249	-961.809492	0.003812	2.392066	0.56%
**6aH**	-961.761637	-961.740848	-961.739904	-961.810356	0.002948	1.849898	1.41%
**6aI**	-961.762385	-961.741255	-961.740311	-961.812327	0.000977	0.613077	11.37%
**6aJ**	-961.761694	-961.741115	-961.740171	-961.809567	0.003737	2.345003	0.61%
**6aK**	-961.761297	-961.740323	-961.739379	-961.810997	0.002307	1.447664	2.78%
**6aL**	-961.762100	-961.741290	-961.740346	-961.810727	0.002577	1.617092	2.09%
**6aM**	-961.763188	-961.742920	-961.741975	-961.810351	0.002953	1.853036	1.40%
**6aN**	-961.761903	-961.741079	-961.740135	-961.810582	0.002722	1.708081	1.79%

E, *E′*, *H*, *G*: total energy, total energy with zero point energy (*ZPE*), enthalpy, and Gibbs free energy

Figure S4. Main conformers of **6a** in NMR calculation.

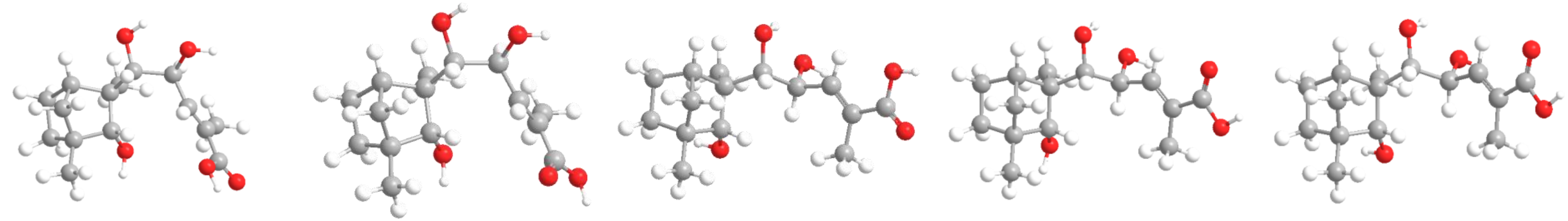

6aA **6aB** **6aC** **6aD** **6aE**

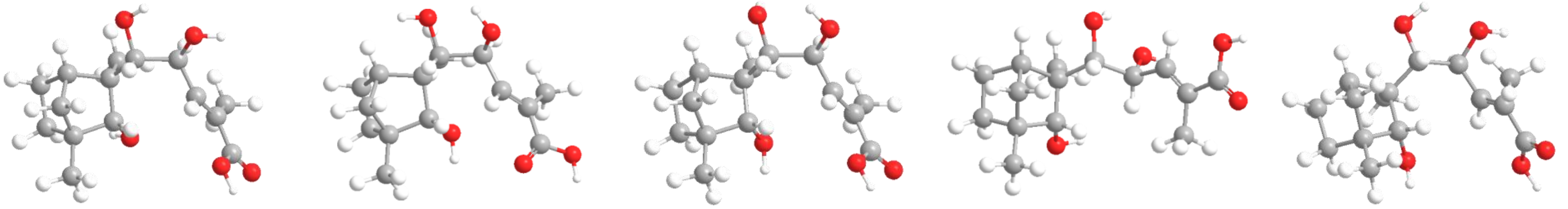

6aF **6aG** **6aH** **6aI** **6aJ**

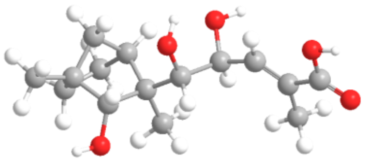

6aK

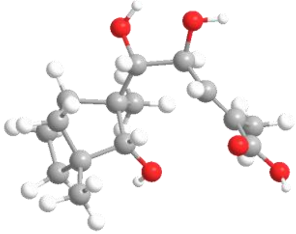

6aL

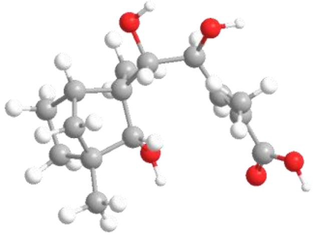

6aM

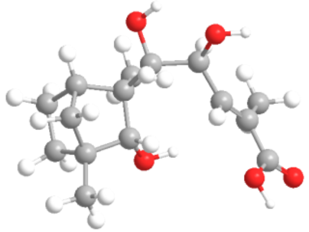

6aN

Table S5. Energy analysis for conformers of **6bA–6bF** at B3LYP/6-31G(d) level in the gas phase

Species	*E′*=*E*+*ZPE*	*E*	*H*	*G*	*ΔG*	*ΔE*(kcal/mol)	*PE*%
**6bA**	-961.759791	-961.738977	-961.738032	-961.808480	0.003372	2.115962	1.60%
**6bB**	-961.759853	-961.739083	-961.738139	-961.808436	0.003416	2.143572	1.53%
**6bC**	-961.762214	-961.741234	-961.740290	-961.811852	0	0	57.17%
**6bD**	-961.762236	-961.741352	-961.740407	-961.811482	0.000370	0.232179	38.63%
**6bE**	-961.758853	-961.738074	-961.737130	-961.807592	0.004260	2.673190	0.63%
**6bF**	-961.758789	-961.738105	-961.737160	-961.807263	0.004589	2.879641	0.44%

E, *E′*, *H*, *G*: total energy, total energy with zero point energy (*ZPE*), enthalpy, and Gibbs free energy

Figure S5. Main conformers of **6b** in NMR and ECD calculations.

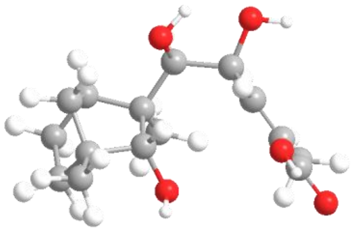

6bA

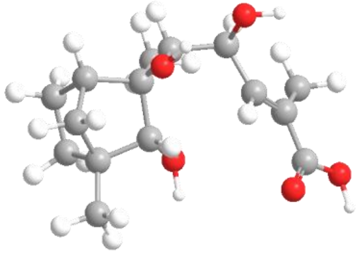

6bB

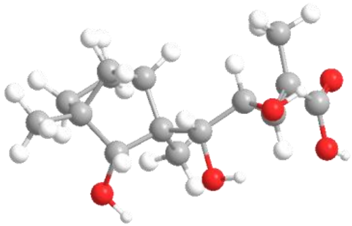

6bC

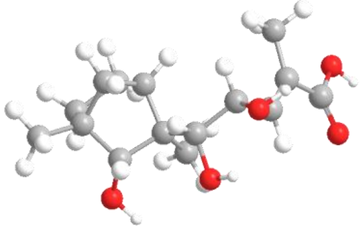

6bD

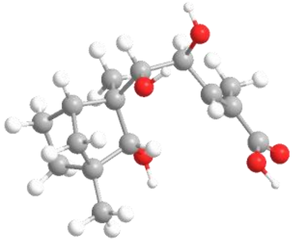

6bE

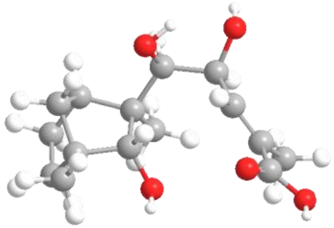

6bF

Table S6. DP4+ analysis results of **6a** (Isomer 1) and **6b** (Isomer 2) with **6**

	A	B	C	D	E	F	G	H
1	Functional		Solvent?		Basis Set		Type of Data	
2	mPW1PW91		PCM		6-311+G(d,p)		Shielding Tensors	
3								
12			DP4+	0.00%	100.00%	-	-	-
14	Nuclei	sp2?	xperimenta	Isomer 1	Isomer 2	Isomer 3	Isomer 4	Isomer 5
15	C		49.8	133.4	134.1			
16	C		82.2	103.1	100.4			
17	C		47.8	134.1	136.8			
18	C		44.7	139.4	139.4			
19	C		27.6	158.2	158.2			
20	C		26.7	159.4	159.5			
21	C		41.5	145.1	144.0			
22	C		80.5	107.7	104.1			
23	C		70.6	117.3	115.5			
24	C	x	138.4	36.2	37.0			
25	C	x	135.7	52.5	51.5			
26	C	x	176.1	11.3	11.6			
27	C		14.3	172.7	172.8			
28	C		19.9	175.0	175.1			
29	C		12.2	167.4	166.8			

	A	B	C	D	E	F	G	H
1	Functional		Solvent?		Basis Set		Type of Data	
2	mPW1PW91		PCM		6-311+G(d,p)		Shielding Tensors	
3								
4			Isomer 1	Isomer 2	Isomer 3	Isomer 4	Isomer 5	Isomer 6
5	sDP4+ (H data)		-	-	-	-	-	-
6	sDP4+ (C data)		0.60%	99.40%	-	-	-	-
7	sDP4+ (all data)		0.60%	99.40%	-	-	-	-
8	uDP4+ (H data)		-	-	-	-	-	-
9	uDP4+ (C data)		0.14%	99.86%	-	-	-	-
10	uDP4+ (all data)		0.14%	99.86%	-	-	-	-
11	DP4+ (H data)		-	-	-	-	-	-
12	DP4+ (C data)		0.00%	100.00%	-	-	-	-
13	DP4+ (all data)		0.00%	100.00%	-	-	-	-

S2.2. Computational details for 6 (ECD)

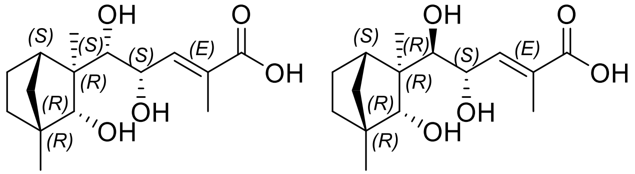

6a **6b**

Conformation search based on molecular mechanics with MMFF force fields were performed for **6b** gave 6 stable conformers with populations higher than 1%. All these conformers were further optimized by the density functional theory method at the B3LYP/6-31G(d) level by Gaussian 16 program package. The ECD were calculated using density functional theory (TDDFT) at B3LYP/6-31+G(d) level in methanol with IEFPCM model. The calculated ECD curves were all generated using SpecDis 1.71.

Figure S6. Comparison of the calculated ECD spectra for possible structure with the experimental spectrum of **6** in methanol with PCM model.

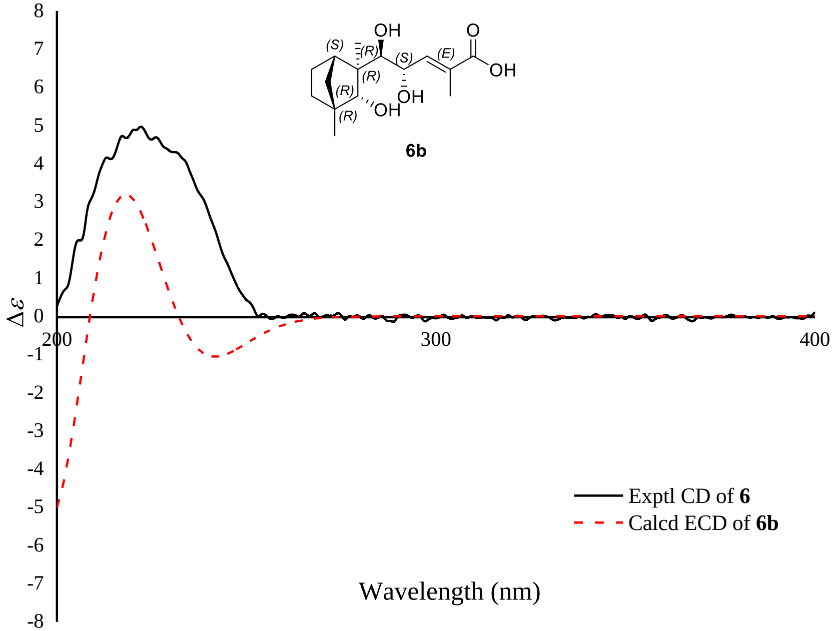

Section S3. Calculational details for 6*S*,9*S*,14*S*-7 and 6*S*,9*S*,14*R*-8

S3.1. Computational details for 6S,9S,14S-7 and 6S,9S,14R-8 (NMR)

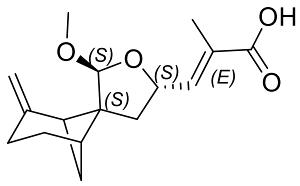

7*S*,9*S*,14*S*-**7**

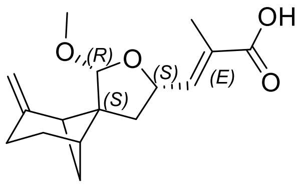

7*S*,9*S*,14*R*-**8**

Conformation search based on molecular mechanics with MMFF force fields were performed for 7*S*,9*S*,14*S*-**7** and 7*S*,9*S*,14*R*-**8** gave 5 and 6 stable conformers with populations higher than 1%, respectively. All these conformers were further optimized by the density functional theory method at the B3LYP/6-31G(d) level by Gaussian 16 program package. Gauge Independent Atomic Orbital (GIAO) calculations of their ^{1}H and ^{13}C NMR chemical shifts using density functional theory (DFT) at the mPW1PW91/6-311+G(d,p) level with the PCM model in methanol. The calculated NMR data of these conformers were averaged according to the Boltzmann distribution theory and their relative Gibbs free energy. The ^{1}H and ^{13}C NMR chemical shifts for TMS were also calculated by the same procedures and used as the reference. After calculation, the experimental and calculated data were evaluated by linear correlation coefficients (R^2) and the improved probability DP4+ method.

Table S7. Energy analysis for conformers of 7*S*,9*S*,14*R*-**8A**–7*S*,9*S*,14*R*-**8F** at B3LYP/6-31G(d) level in the gas phase

Species	*E'*=*E*+*ZPE*	*E*	*H*	*G*	*ΔG*	*ΔE*(kcal/mol)	*PE*%
7*S*,9*S*,14*R*-**8A**	-923.429467	-923.409966	-923.409022	-923.478920	0.000002	0.001255	25.87%
7*S*,9*S*,14*R*-**8B**	-923.429467	-923.409966	-923.409022	-923.478922	0	0	25.92%
7*S*,9*S*,14*R*-**8C**	-923.429472	-923.410060	-923.409116	-923.478765	0.000157	0.098519	21.95%
7*S*,9*S*,14*R*-**8D**	-923.429466	-923.409965	-923.409021	-923.478917	0.000005	0.003138	25.78%
7*S*,9*S*,14*R*-**8E**	-923.424220	-923.404699	-923.403755	-923.474439	0.004483	2.813125	0.22%
7*S*,9*S*,14*R*-**8F**	-923.425652	-923.406341	-923.405397	-923.474571	0.004351	2.730294	0.26%

E, *E'*, *H*, *G*: total energy, total energy with zero point energy (*ZPE*), enthalpy, and Gibbs free energy

Figure S7. Main conformers of 7*S*,9*S*,14*R*-**8** in NMR and ECD calculations.

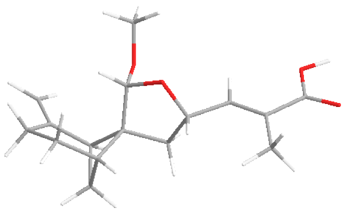
7*S*,9*S*,14*R*-**8A**

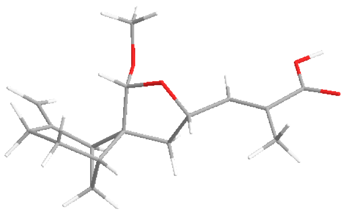
7*S*,9*S*,14*R*-**8B**

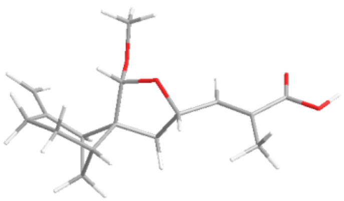
7*S*,9*S*,14*R*-**8C**

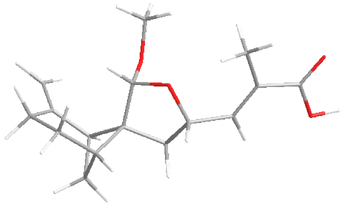
7*S*,9*S*,14*R*-**8D**

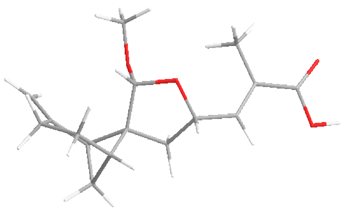
7*S*,9*S*,14*R*-**8E**

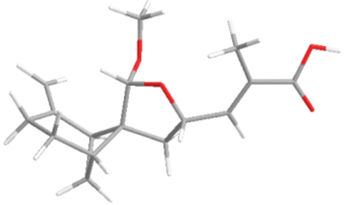
7*S*,9*S*,14*R*-**8F**

Table S8. Energy analysis for conformers of 6*S*,9*S*,14*S*-**7A**–6*S*,9*S*,14*S*-**7E** at B3LYP/6-31G(d) level in the gas phase

Species	*E'*=*E*+*ZPE*	*E*	*H*	*G*	*ΔG*	*ΔE*(kcal/mol)	*PE*%
7*S*,9*S*,14*S*-**7A**	-923.430034	-923.410552	-923.409608	-923.479402	0	0	46.18%
7*S*,9*S*,14*S*-**7B**	-923.430071	-923.410679	-923.409735	-923.479299	0.000103	0.064633	41.41%
7*S*,9*S*,14*S*-**7C**	-923.427379	-923.407883	-923.406939	-923.477329	0.002073	1.300827	5.13%
7*S*,9*S*,14*S*-**7D**	-923.427568	-923.408186	-923.407242	-923.477005	0.002397	1.504140	3.64%
7*S*,9*S*,14*S*-**7E**	-923.427568	-923.408186	-923.407242	-923.477005	0.002397	1.504140	3.64%

Figure S8. Main conformers of 7*S*,9*S*,14*S*-**7** in NMR and ECD calculations.

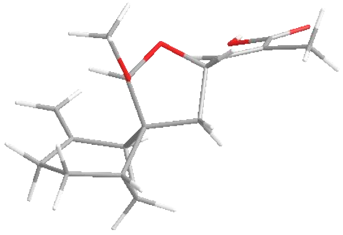
7*S*,9*S*,14*S*-**7A**

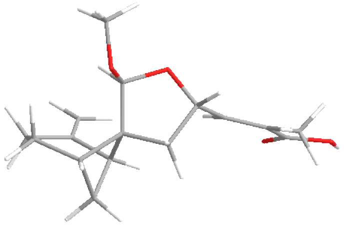
7*S*,9*S*,14*S*-**7B**

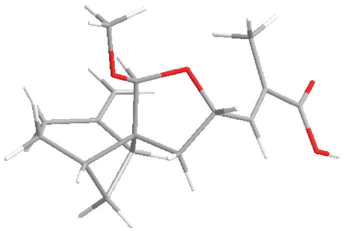
7*S*,9*S*,14*S*-**7C**

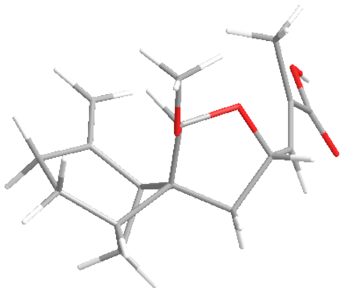
7*S*,9*S*,14*S*-**7D**

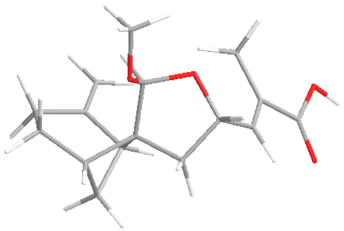
7*S*,9*S*,14*S*-**7E**

Table S9. DP4+ analysis results of 7*S*,9*S*,14*R*-**8** (Isomer 1) and 7*S*,9*S*,14*S*-**7** (Isomer 2) with 7*S*,9*S*,14*R*-**8**

	A	B	C	D	E	F	G	H
1	Functional		Solvent?		Basis Set		Type of Data	
2	mPW1PW91		PCM		6-311+G(d,p)		Shielding Tensors	
3								
12			DP4+	86.91%	13.09%	-	-	-
14	Nuclei	sp2?	xperimenta	Isomer 1	Isomer 2	Isomer 3	Isomer 4	Isomer 5
15	C		49.1	134.7	132.2			
16	C	x	151	24.8	24.2			
17	C		24.3	159.9	159.5			
18	C		24.7	160.6	160.3			
19	C		40.6	144.3	144.2			
20	C		56.5	126.7	127.2			
21	C		29.1	159.6	160.2			
22	C		39.4	146.4	144.3			
23	C		75.7	108.9	108.9			
24	C	x	144.2	30.0	30.4			
25	C	x	130.2	55.0	55.1			
26	C	x	171.3	11.7	11.5			
27	C		12.8	173.3	172.9			
28	C		106.8	78.8	77.3			
29	C	x	107.8	75.1	74.8			
30	C		55.4	132.3	132.6			

	A	B	C	D	E	F	G	H
1	Functional		Solvent?		Basis Set		Type of Data	
2	mPW1PW91		PCM		6-311+G(d,p)		Shielding Tensors	
3								
4			Isomer 1	Isomer 2	Isomer 3	Isomer 4	Isomer 5	Isomer 6
5	sDP4+ (H data)		-	-	-	-	-	-
6	sDP4+ (C data)		89.98%	10.02%	-	-	-	-
7	sDP4+ (all data)		89.98%	10.02%	-	-	-	-
8	uDP4+ (H data)		-	-	-	-	-	-
9	uDP4+ (C data)		42.51%	57.49%	-	-	-	-
10	uDP4+ (all data)		42.51%	57.49%	-	-	-	-
11	DP4+ (H data)		-	-	-	-	-	-
12	DP4+ (C data)		86.91%	13.09%	-	-	-	-
13	DP4+ (all data)		86.91%	13.09%	-	-	-	-

Table S10. DP4+ analysis results of 7*S*,9*S*,14*R*-**8** (Isomer 1) and 7*S*,9*S*,14*S*-**7** (Isomer 2) with 7*S*,9*S*,14*S*-**7**

	A	B	C	D	E	F	G	H
1	Functional		Solvent?		Basis Set		Type of Data	
2	mPW1PW91		PCM		6-311+G(d,p)		Shielding Tensors	
3								
12			DP4+	0.11%	99.89%	-	-	-
14	Nuclei	sp2?	xperimenta	Isomer 1	Isomer 2	Isomer 3	Isomer 4	Isomer 5
15	C		52	134.7	132.2			
16	C	x	150	24.8	24.2			
17	C		23.1	159.9	159.5			
18	C		23.5	160.6	160.3			
19	C		39.4	144.3	144.2			
20	C		54.7	126.7	127.2			
21	C		26.5	159.6	160.2			
22	C		40.4	146.4	144.3			
23	C		73.7	108.9	108.9			
24	C	x	143	30.0	30.4			
25	C	x	128.1	55.0	55.1			
26	C	x	169.8	11.7	11.5			
27	C		11.4	173.3	172.9			
28	C		106.4	78.8	77.3			
29	C	x	107	75.1	74.8			
30	C		53.3	132.3	132.6			

	A	B	C	D	E	F	G	H
1	Functional		Solvent?		Basis Set		Type of Data	
2	mPW1PW91		PCM		6-311+G(d,p)		Shielding Tensors	
3								
4			Isomer 1	Isomer 2	Isomer 3	Isomer 4	Isomer 5	Isomer 6
5	sDP4+ (H data)		-	-	-	-	-	-
6	sDP4+ (C data)		2.83%	97.17%	-	-	-	-
7	sDP4+ (all data)		2.83%	97.17%	-	-	-	-
8	uDP4+ (H data)		-	-	-	-	-	-
9	uDP4+ (C data)		3.72%	96.28%	-	-	-	-
10	uDP4+ (all data)		3.72%	96.28%	-	-	-	-
11	DP4+ (H data)		-	-	-	-	-	-
12	DP4+ (C data)		0.11%	99.89%	-	-	-	-
13	DP4+ (all data)		0.11%	99.89%	-	-	-	-

S3.2. Computational details for 7S,9S,14R-8 and 7S,9S,14S-7 (ECD)

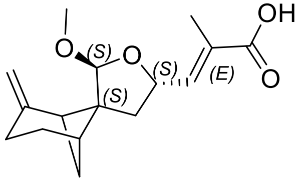

7*S*,9*S*,14*S*-**7**

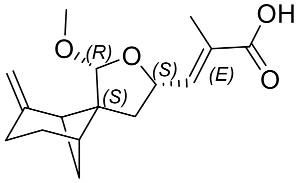

7*S*,9*S*,14*R*-**8**

Conformation search based on molecular mechanics with MMFF force fields were performed for 7*S*,9*S*,14*S*-**7** and 7*S*,9*S*,14*R*-**8** gave 5 and 6 stable conformers with populations higher than 1%, respectively. All these conformers were further optimized by the density functional theory method at the B3LYP/6-31G(d) level by Gaussian 16 program package. The ECD were calculated using density functional theory (TDDFT) at B3LYP/6-31+G(d) level in methanol with IEFPCM model. The calculated ECD curves were all generated using SpecDis 1.71 with σ = 0.30 eV, UV shift -20 and -10 nm, respectively.

Figure S9. Comparison of the calculated ECD spectra for possible structure with the experimental spectrum of 7*S*,9*S*,14*S*-**7** and 7*S*,9*S*,14*R*-**8** in methanol.

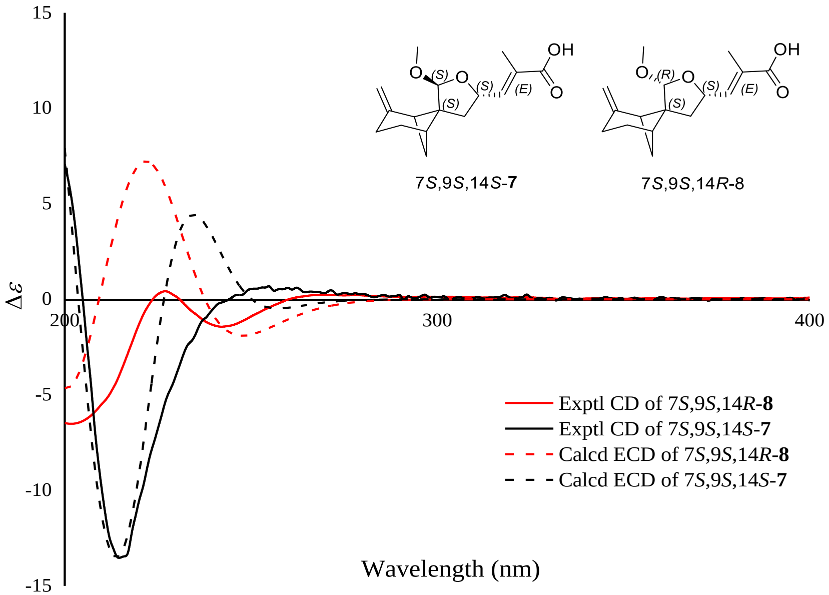

Section S4. Calculational details for 10

S4.1. Computational details for 10 (ECD)

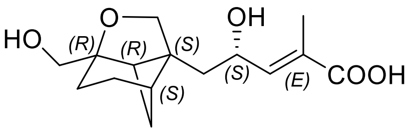

10a

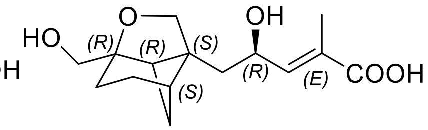

10b

Conformation search based on molecular mechanics with MMFF force fields were performed for **10a** and **10b** gave 9 and 12 stable conformers with populations higher than 1%, respectively. All these conformers were further optimized by the density functional theory method at the B3LYP/6-31G(d) level by Gaussian 16 program package. The ECD were calculated using density functional theory (TDDFT) at B3LYP/6-31+G(d) level in methanol with IEFPCM model. The calculated ECD curves were all generated using SpecDis 1.71 with σ = 0.30 eV, UV shift -18 and -18 nm, respectively.

Figure S10. Comparison of the calculated ECD spectra for possible structure with the experimental spectrum of **10** in methanol with PCM model.

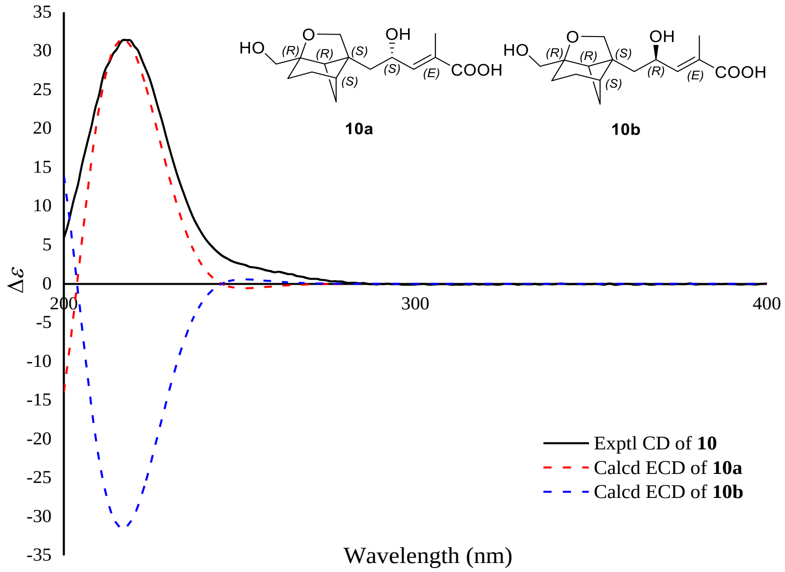

Table S11. Energy analysis for conformers of **10aA–10aI** at B3LYP/6-31G(d) level in the gas phase

Species	*E′*=*E*+*ZPE*	*E*	*H*	*G*	*ΔG*	*ΔE*(kcal/mol)	*PE*%
**10aA**	-960.558406	-960.538984	-960.538040	-960.607670	0	0	38.64%
**10aB**	-960.558367	-960.539069	-960.538125	-960.607301	0.000369	0.231551	26.14%
**10aC**	-960.556762	-960.537217	-960.536273	-960.606432	0.001238	0.776857	10.41%
**10aD**	-960.557455	-960.538090	-960.537146	-960.606438	0.001232	0.773092	10.47%
**10aE**	-960.556308	-960.537096	-960.536152	-960.604867	0.002803	1.758909	1.98%
**10aF**	-960.556735	-960.537309	-960.536365	-960.605920	0.001750	1.098142	6.05%
**10aG**	-960.556376	-960.537295	-960.536351	-960.604531	0.003139	1.969752	1.39%
**10aH**	-960.555902	-960.536542	-960.535598	-960.604874	0.002796	1.754517	2.00%
**10aI**	-960.556216	-960.536805	-960.535860	-960.605236	0.002434	1.527358	2.93%

E, *E′*, *H*, *G*: total energy, total energy with zero point energy (*ZPE*), enthalpy, and Gibbs free energy

Figure S11. Main conformers of **10a** in ECD calculation.

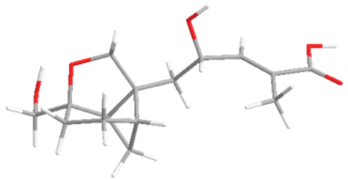

10aA

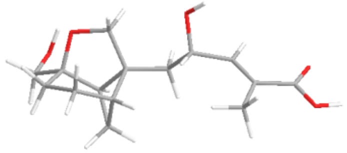

10aB

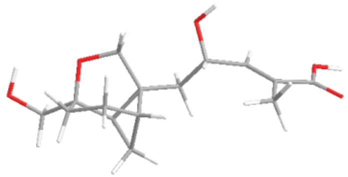

10aC

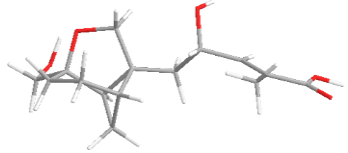

10aD

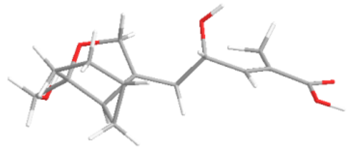

10aE

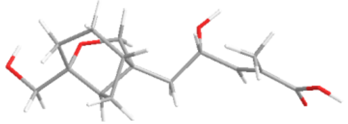

10aF

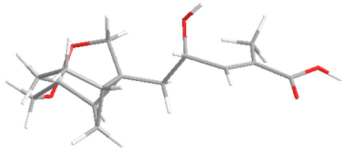

10aG

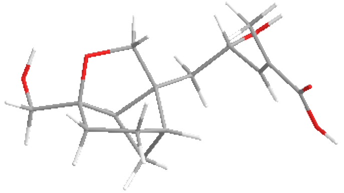

10aH

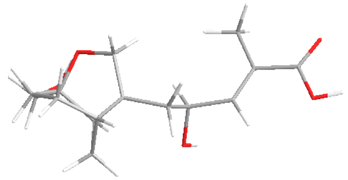

10aI

Table S12. Energy analysis for conformers of **10bA–10bL** at B3LYP/6-31G(d) level in the gas phase

Species	*E′*=*E*+*ZPE*	*E*	*H*	*G*	*ΔG*	*ΔE*(kcal/mol)	*PE*%
**10bA**	-960.557174	-960.537738	-960.536794	-960.606304	0	0	29.81%
**10bB**	-960.555406	-960.536074	-960.535130	-960.604205	0.002099	1.317142	3.22%
**10bC**	-960.557188	-960.537843	-960.536899	-960.606157	0.000147	0.092244	25.51%
**10bD**	-960.556102	-960.536710	-960.535766	-960.605386	0.000918	0.576054	11.27%
**10bE**	-960.555361	-960.536147	-960.535203	-960.603769	0.002535	1.590737	2.03%

**10bF**	-960.555494	-960.535950	-960.535006	-960.604963	0.001341	0.841490	7.20%
**10bG**	-960.556164	-960.536849	-960.535905	-960.605279	0.001025	0.643197	10.06%
**10bH**	-960.555043	-960.535766	-960.534822	-960.604090	0.002214	1.389306	2.85%
**10bI**	-960.553717	-960.534287	-960.533343	-960.602677	0.003627	2.275977	0.64%
**10bJ**	-960.556008	-960.536753	-960.535809	-960.604568	0.001736	1.089356	4.73%
**10bK**	-960.554282	-960.534900	-960.533956	-960.602934	0.003370	2.114707	0.84%
**10bL**	-960.555095	-960.535952	-960.535008	-960.603677	0.002627	1.648467	1.84%

E, *E*′, *H*, *G*: total energy, total energy with zero point energy (*ZPE*), enthalpy, and Gibbs free energy

Figure S12. Main conformers of **10b** in ECD calculation.

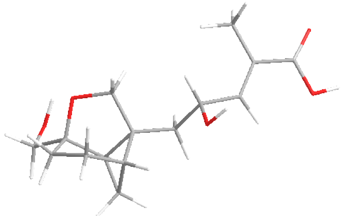

10bA

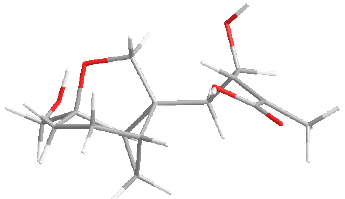

10bB

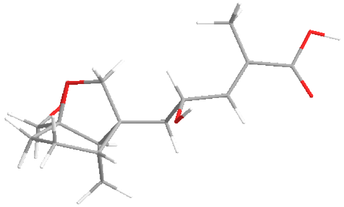

10bC

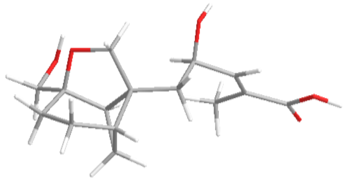

10bD

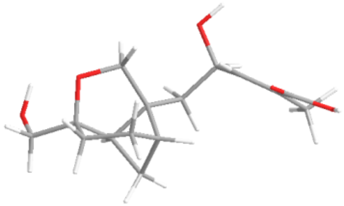

10bE

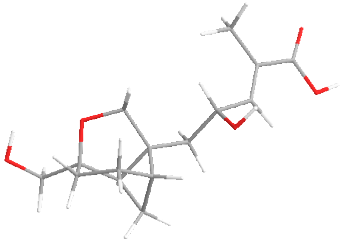

10bF

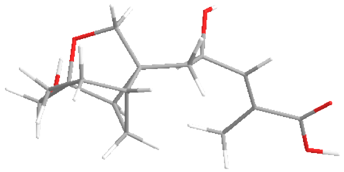

10bG

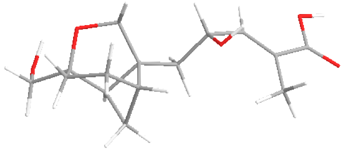

10bH

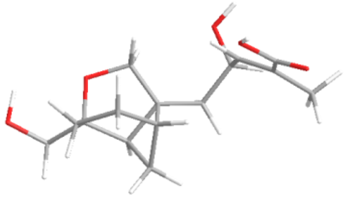

10bI

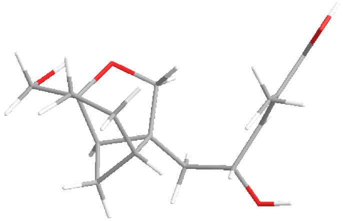

10bJ

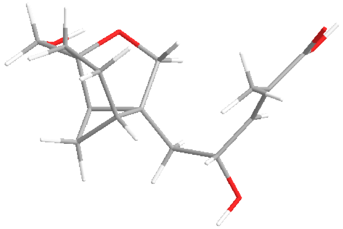

10bK

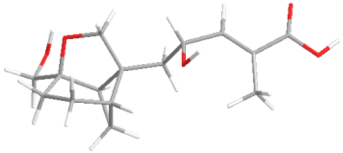

10bL

Section S5. Calculational details for 11

S5.1. Computational details for 11 (NMR)

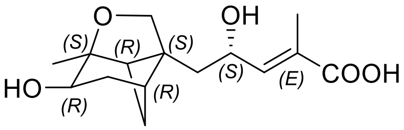

11a

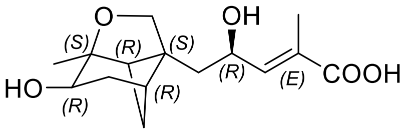

11b

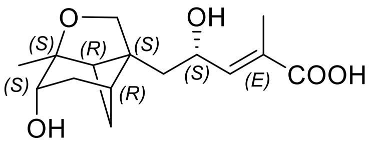

11a

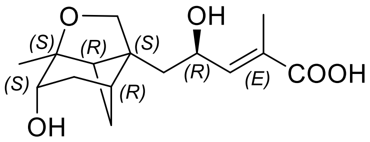

11b

Conformation search based on molecular mechanics with MMFF force fields were performed for **11a**, **11b**, **11c** and **11d** gave 8, 14, 3 and 4 stable conformers with populations higher than 1%, respectively. All these conformers were further optimized by the density functional theory method at the B3LYP/6-31G(d) level by Gaussian 16 program package. Gauge Independent Atomic Orbital (GIAO) calculations of their ^{1}H and ^{13}C NMR chemical shifts using density functional theory (DFT) at the mPW1PW91/6-311+G(d,p) level with the PCM model in methanol. The calculated NMR data of these conformers were averaged according to the Boltzmann distribution theory and their relative Gibbs free energy. The ^{1}H and ^{13}C NMR chemical shifts for TMS were also calculated by the same procedures and used as the reference. After calculation, the experimental and calculated data were evaluated by the improved probability DP4+ method.

Table S13. Energy analysis for conformers of **11aA–11aH** at B3LYP/6-31G(d) level in the gas phase

Species	*E′=E+ZPE*	*E*	*H*	*G*	*ΔG*	*ΔE*(kcal/mol)	*PE*%
**11aA**	-960.564000	-960.544556	-960.543611	-960.612659	0	0	33.74%
**11aB**	-960.564017	-960.544659	-960.543714	-960.612494	0.000165	0.103539	28.33%
**11aC**	-960.561967	-960.542677	-960.541733	-960.610252	0.002407	1.510415	2.63%
**11aD**	-960.563096	-960.543659	-960.542715	-960.611756	0.000903	0.566641	12.96%
**11aE**	-960.562085	-960.542885	-960.541941	-960.610227	0.002432	1.526103	2.56%
**11aF**	-960.563234	-960.543882	-960.542938	-960.611794	0.000865	0.542796	13.49%
**11aG**	-960.561573	-960.542129	-960.541185	-960.610009	0.002650	1.662900	2.03%
**11aH**	-960.561958	-960.542440	-960.541496	-960.610703	0.001956	1.227409	4.25%

E, *E′*, *H*, *G*: total energy, total energy with zero point energy (*ZPE*), enthalpy, and Gibbs free energy

Figure S13. Main conformers of **11a** in NMR calculation.

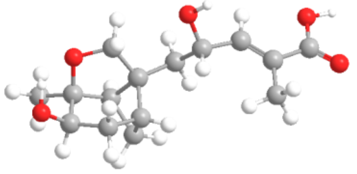

11aA

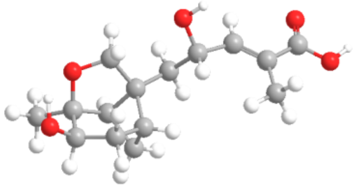

11aB

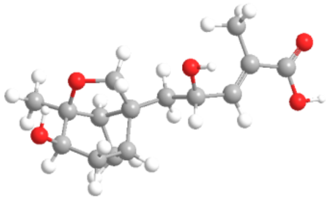

11aC

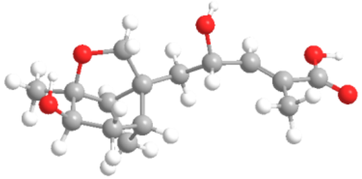

11aD

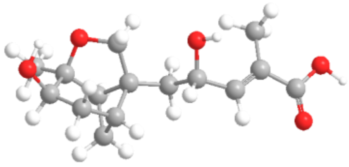

11aE

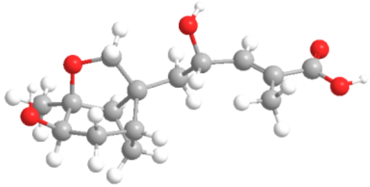

11aF

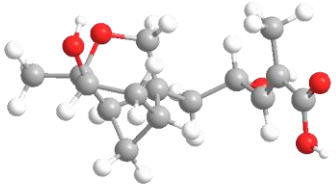

11aG

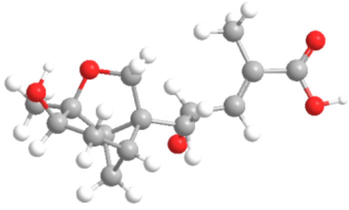

11aH

Table S14. Energy analysis for conformers of **11bA–11bN** at B3LYP/6-31G(d) level in the gas phase

Species	*E′*=*E*+*ZPE*	*E*	*H*	*G*	*ΔG*	*ΔE*(kcal/mol)	*PE*%
**11bA**	-960.562876	-960.543336	-960.542392	-960.611789	0.000304	0.190763	22.95%
**11bB**	-960.561084	-960.541698	-960.540754	-960.609465	0.002628	1.649095	1.96%
**11bC**	-960.562955	-960.543476	-960.542532	-960.612093	0	0	31.68%
**11bD**	-960.561578	-960.542164	-960.541220	-960.609996	0.002097	1.315887	3.43%
**11bE**	-960.561044	-960.541770	-960.540825	-960.609041	0.003052	1.915159	1.25%
**11bF**	-960.561830	-960.542299	-960.541354	-960.611060	0.001033	0.648217	10.60%
**11bG**	-960.560382	-960.540978	-960.540034	-960.608983	0.003110	1.951555	1.17%
**11bH**	-960.560787	-960.541426	-960.540482	-960.609337	0.002756	1.729416	1.71%
**11bI**	-960.561946	-960.542458	-960.541514	-960.610675	0.001418	0.889808	7.05%
**11bJ**	-960.561607	-960.542290	-960.541345	-960.609776	0.002317	1.453940	2.72%
**11bK**	-960.562044	-960.542663	-960.541719	-960.610520	0.001573	0.987072	5.98%
**11bL**	-960.560888	-960.541631	-960.540687	-960.609218	0.002875	1.804090	1.50%
**11bM**	-960.561806	-960.542394	-960.541450	-960.610718	0.001375	0.862826	7.38%
**11bN**	-960.559956	-960.540460	-960.539516	-960.608382	0.003711	2.328688	0.62%

E, *E′*, *H*, *G*: total energy, total energy with zero point energy (*ZPE*), enthalpy, and Gibbs free energy

Figure S14. Main conformers of **11b** in NMR calculation.

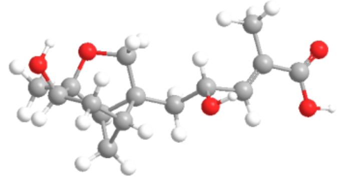
11bA

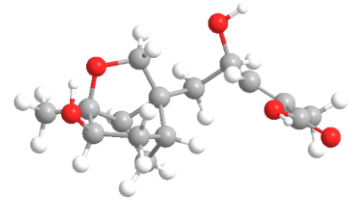
11bB

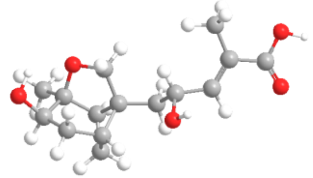
11bC

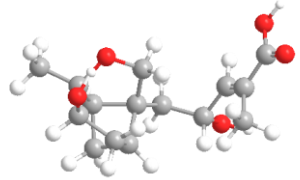
11bD

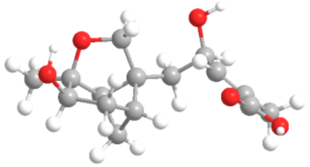
11bE

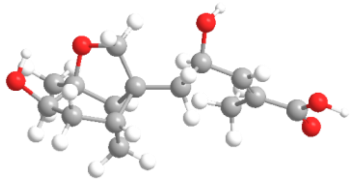
11bF

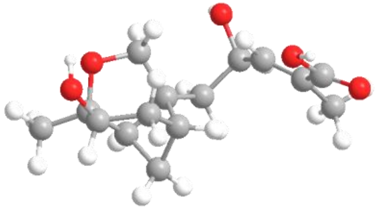
11bG

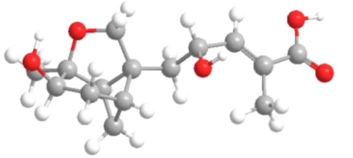
11bH

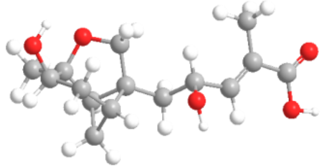
11bI

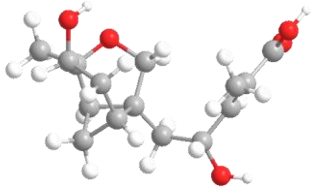
11bJ

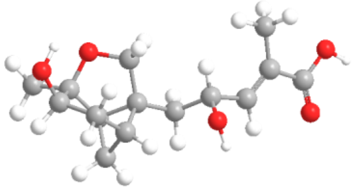
11bK

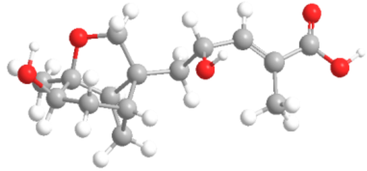
11bL

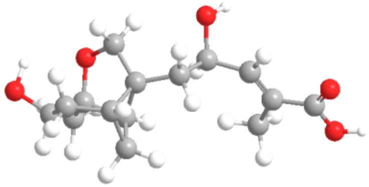
11bM

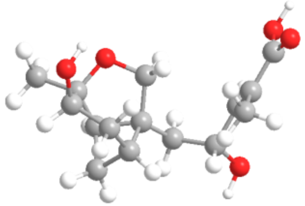
11bN

Table S15. Energy analysis for conformers of **11cA–11cC** at B3LYP/6-31G(d) level in the gas phase

Species	*E′*=*E*+*ZPE*	*E*	*H*	*G*	*ΔG*	*ΔE*(kcal/mol)	*PE*%
**11cA**	-960.559341	-960.539753	-960.538809	-960.608210	0	0	89.73%
**11cB**	-960.557064	-960.537596	-960.536652	-960.605459	0.002751	1.726279	4.86%
**11cC**	-960.557322	-960.537958	-960.537014	-960.605559	0.002651	1.663528	5.40%

E, *E′*, *H*, *G*: total energy, total energy with zero point energy (*ZPE*), enthalpy, and Gibbs free energy

Figure S15. Main conformers of **11c** in NMR and ECD calculations.

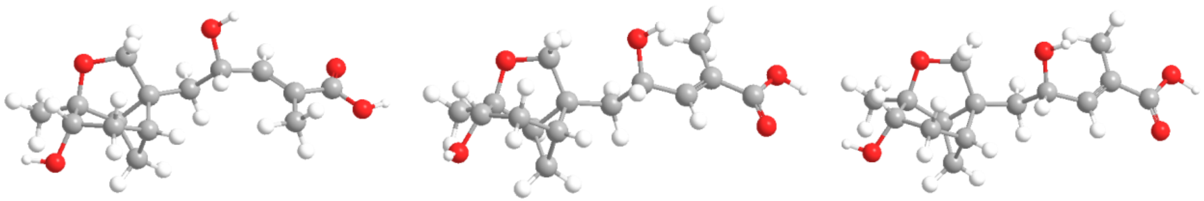

11cA **11cB** **11cC**

Table S16. Energy analysis for conformers of **11dA–11dD** at B3LYP/6-31G(d) level in the gas phase

Species	*E′*=*E*+*ZPE*	*E*	*H*	*G*	*ΔG*	*ΔE*(kcal/mol)	*PE*%
**11dA**	-960.558201	-960.538472	-960.537528	-960.607671	0	0	43.87%
**11dB**	-960.557972	-960.538260	-960.537316	-960.607436	0.000235	0.147465	34.20%
**11dC**	-960.555786	-960.536186	-960.535242	-960.604696	0.002975	1.866841	1.87%
**11dD**	-960.557404	-960.537690	-960.536745	-960.606932	0.000739	0.463730	20.05%

E, *E′*, *H*, *G*: total energy, total energy with zero point energy (*ZPE*), enthalpy, and Gibbs free energy

Figure S16. Main conformers of **11d** in NMR calculation.

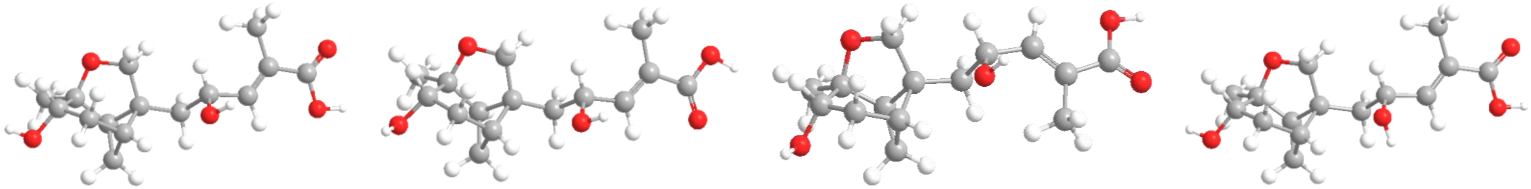

11dA **11dB** **11dC** **11dD**

Table S17. DP4+ analysis results of **11a** (Isomer 1), **11b** (Isomer 2), **11c** (Isomer 3) and **11d** (Isomer 4) with **11**

	A	B	C	D	E	F	G	H
1	Functional		Solvent?		Basis Set		Type of Data	
2	mPW1PW91		PCM		6-311+G(d,p)		Shielding Tensors	
3								
12			DP4+	0.00%	0.00%	100.00%	0.00%	-
14	Nuclei	sp2?	xperimenta	Isomer 1	Isomer 2	Isomer 3	Isomer 4	Isomer 5
15	C		87.4	95.4	93.9	96.6	94.6	
16	C		72.3	114.6	114.6	109.3	109.5	
17	C		32.9	149.9	150.3	151.9	151.4	
18	C		37.9	145.5	143.2	146.5	144.0	
19	C		22.8	163.2	163.7	162.9	163.5	
20	C		51.3	133.1	133.0	132.7	132.0	
21	C		54.7	128.4	128.6	127.8	128.1	
22	C		71.5	111.2	113.5	111.6	114.1	
23	C		21	165.5	165.4	165.7	165.1	
24	C		40.1	146.1	147.1	146.1	146.0	
25	C		65.8	116.5	114.9	116.9	114.1	
26	C	x	141.8	30.7	30.6	29.6	30.5	
27	C	x	129.3	54.6	54.5	54.5	54.7	
28	C	x	172.2	11.6	11.6	11.5	11.6	
29	C		11.8	172.8	172.8	172.8	172.8	
30								
31	H		3.63	28.11	28.15	28.07	28.03	
32	H		2.25	29.36	29.43	29.43	29.40	
33	H		1.85	30.25	30.26	29.85	30.00	
34	H		2.27	29.45	29.32	29.45	29.14	
35	H		2.12	29.82	29.78	29.84	29.75	
36	H		2.06	30.32	30.38	29.61	29.61	
37	H		2.1	29.53	29.53	29.71	29.73	
38	H		3.85	28.07	27.90	28.08	28.13	
39	H		3.61	27.78	27.86	27.76	27.74	
40	H		1.31	30.03	30.01	30.53	30.52	
41	H		1.31	30.85	30.83	30.69	30.72	
42	H		1.31	30.61	30.61	30.23	30.17	
43	H		2	29.90	29.68	29.90	29.62	
44	H		1.81	30.00	30.22	30.00	30.35	
45	H		4.42	27.13	27.16	27.02	27.25	
46	H	x	6.57	24.54	24.55	24.47	24.56	
47	H		1.83	30.03	29.87	29.65	29.92	
48	H		1.83	29.85	29.99	29.96	29.93	
49	H		1.83	29.82	29.90	29.84	30.04	

Functional	Solvent?	Basis Set	Type of Data
mPW1PW91	PCM	6-311+G(d,p)	Shielding Tensors

	Isomer 1	Isomer 2	Isomer 3	Isomer 4	Isomer 5	Isomer 6
sDP4+ (H data)	0.16%	0.12%	98.00%	1.72%	-	-
sDP4+ (C data)	11.64%	0.05%	86.80%	1.51%	-	-
sDP4+ (all data)	0.02%	0.00%	99.95%	0.03%	-	-
uDP4+ (H data)	0.33%	0.60%	75.68%	23.40%	-	-
uDP4+ (C data)	38.20%	0.41%	59.62%	1.77%	-	-
uDP4+ (all data)	0.27%	0.01%	98.81%	0.91%	-	-
DP4+ (H data)	0.00%	0.00%	99.46%	0.54%	-	-
DP4+ (C data)	7.91%	0.00%	92.04%	0.05%	-	-
DP4+ (all data)	0.00%	0.00%	100.00%	0.00%	-	-

S5.2. Computational details for 11 (ECD)

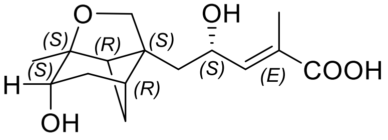

11c

Conformation search based on molecular mechanics with MMFF force fields were performed for **11c** gave 3 stable conformers with populations higher than 1%. All these conformers were further optimized by the density functional theory method at the B3LYP/6-31G(d) level by Gaussian 16 program package. The ECD were calculated using density functional theory (TDDFT) at B3LYP/6-31+G(d) level in methanol with IEFPCM model. The calculated ECD curves were all generated using SpecDis 1.71.

Figure S17. Comparison of the calculated ECD spectra for possible structure with the experimental spectrum of **11** in methanol with PCM model.

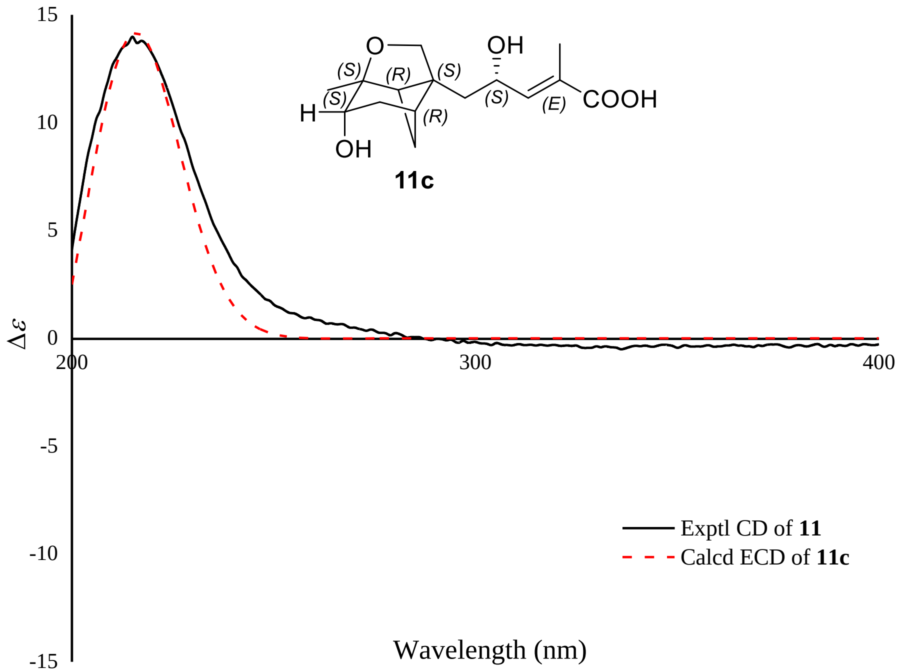

Section S6. Calculational details for 17

S6.1. Computational details for 17 (ECD)

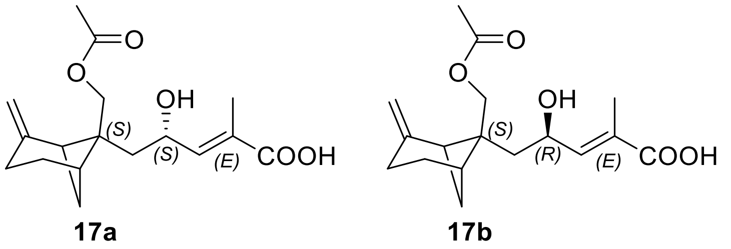

Conformation search based on molecular mechanics with MMFF force fields were performed for **17a** and **17b** gave 19 and 13 stable conformers with populations higher than 1%, respectively. All these conformers were further optimized by the density functional theory method at the B3LYP/6-31G(d) level by Gaussian 16 program package. The ECD were calculated using density functional theory (TDDFT) at B3LYP/6-31+G(d) level in methanol with IEFPCM model. The calculated ECD curves were all generated using SpecDis 1.71 with σ = 0.30 eV, UV shift -30, and -27 nm, respectively.

Figure S18. Comparison of the calculated ECD spectra for possible structure with the experimental spectrum of **17** in methanol with PCM model.

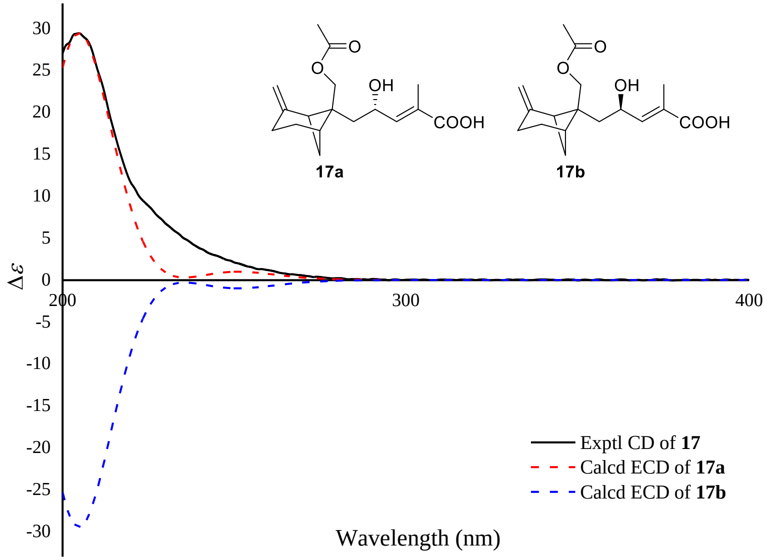

Table S18. Energy analysis for conformers of **17aA–17aS** at B3LYP/6-31G(d) level in the gas phase

Species	*E'*=*E*+*ZPE*	*E*	*H*	*G*	*ΔG*	*ΔE*(kcal/mol)	*PE*%
**17aA**	-1037.954922	-1037.931906	-1037.930962	-1038.009872	0.000998	0.626254	8.07%
**17aB**	-1037.954900	-1037.931995	-1037.931051	-1038.009603	0.001267	0.795055	6.07%
**17aC**	-1037.952727	-1037.929956	-1037.929012	-1038.008229	0.002641	1.657253	1.41%
**17aD**	-1037.954573	-1037.931695	-1037.930751	-1038.009911	0.000959	0.601782	8.41%
**17aE**	-1037.956899	-1037.934403	-1037.933459	-1038.010870	0	0	23.23%
**17aF**	-1037.952910	-1037.929807	-1037.928863	-1038.007908	0.002962	1.858683	1.01%
**17aG**	-1037.952671	-1037.930038	-1037.929093	-1038.007451	0.003419	2.145455	0.62%
**17aH**	-1037.954512	-1037.931415	-1037.930470	-1038.010222	0.000648	0.406626	11.69%
**17aI**	-1037.952702	-1037.929941	-1037.928997	-1038.006025	0.004845	3.040284	0.14%
**17aJ**	-1037.956917	-1037.934534	-1037.933590	-1038.010575	0.000295	0.185115	16.99%
**17aK**	-1037.952971	-1037.930055	-1037.929111	-1038.007230	0.003640	2.284135	0.49%
**17aL**	-1037.954477	-1037.931736	-1037.930792	-1038.009324	0.001546	0.970130	4.51%
**17aM**	-1037.952423	-1037.929553	-1037.928609	-1038.007503	0.003367	2.112824	0.66%
**17aN**	-1037.952905	-1037.929889	-1037.928945	-1038.007778	0.003092	1.940259	0.88%
**17aO**	-1037.953996	-1037.931149	-1037.930205	-1038.008502	0.002368	1.485942	1.89%
**17aP**	-1037.951652	-1037.928838	-1037.927894	-1038.005231	0.005639	3.538526	0.06%
**17aQ**	-1037.954566	-1037.931535	-1037.930591	-1038.010238	0.000632	0.396586	11.89%
**17aR**	-1037.952907	-1037.929841	-1037.928897	-1038.008204	0.002666	1.672940	1.38%
**17aS**	-1037.954282	-1037.931902	-1037.930958	-1038.007455	0.003415	2.142945	0.62%

E, *E'*, *H*, *G*: total energy, total energy with zero point energy (*ZPE*), enthalpy, and Gibbs free energy

Figure S19. Main conformers of **17a** in NMR calculation.

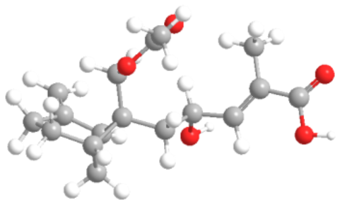

17aA

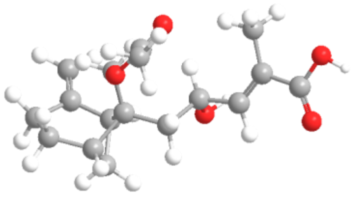

17aB

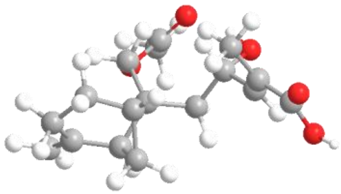

17aC

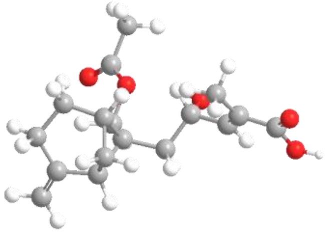

17aD

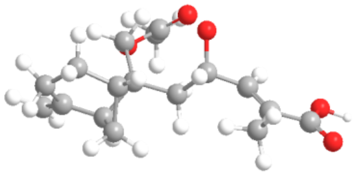

17aE

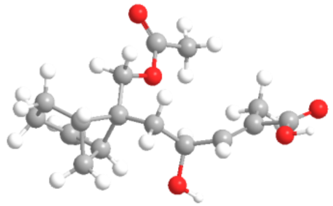

17aF

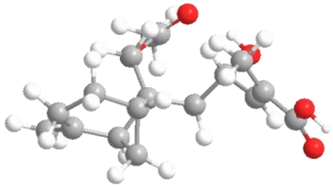

17aG

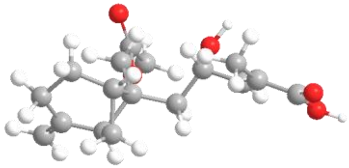

17aH

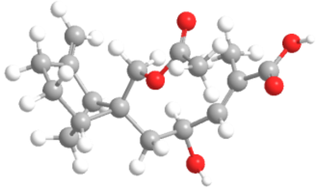

17aI

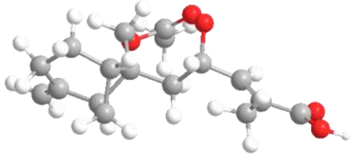

17aJ

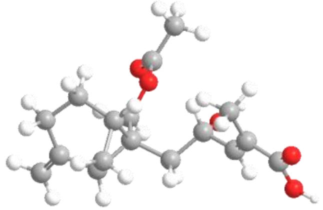

17aK

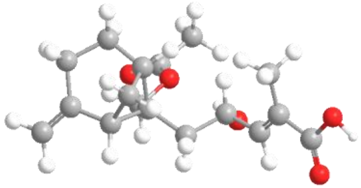

17aL

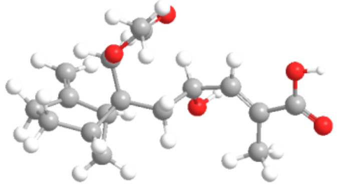

17aM

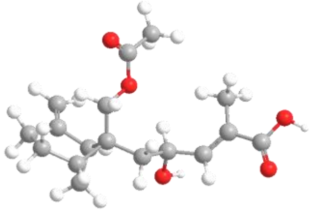

17aN

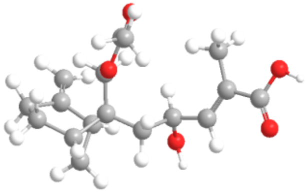

17aO

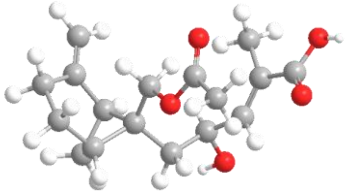

17aP

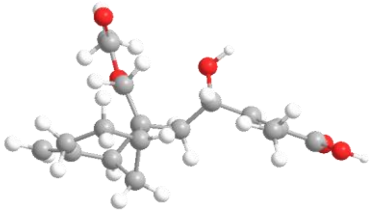

17aQ

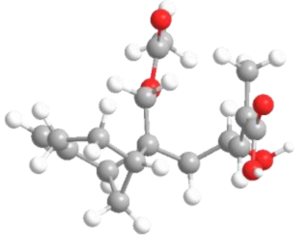

17aR

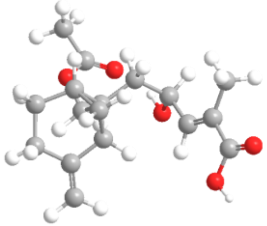

17aS

Table S19. Energy analysis for conformers of **17bA–17bM** at B3LYP/6-31G(d) level in the gas phase

Species	*E′*=*E*+*ZPE*	*E*	*H*	*G*	*ΔG*	*ΔE*(kcal/mol)	*PE*%
**17bA**	-1037.953692	-1037.931004	-1037.930060	-1038.008273	0.003730	2.340610	0.63%
**17bB**	-1037.953707	-1037.931091	-1037.930147	-1038.008255	0.003748	2.351906	0.61%
**17bC**	-1037.957696	-1037.935163	-1037.934219	-1038.012003	0	0	32.61%
**17bD**	-1037.957682	-1037.935279	-1037.934335	-1038.011465	0.000538	0.337600	18.44%
**17bE**	-1037.952088	-1037.929485	-1037.928541	-1038.007015	0.004988	3.130017	0.17%
**17bF**	-1037.955135	-1037.931994	-1037.931050	-1038.010634	0.001369	0.859061	7.64%
**17bG**	-1037.955134	-1037.931994	-1037.931049	-1038.010630	0.001373	0.861571	7.61%
**17bH**	-1037.953943	-1037.931073	-1037.930129	-1038.009434	0.002569	1.612072	2.14%
**17bI**	-1037.955129	-1037.932096	-1037.931152	-1038.010980	0.001023	0.641942	11.03%
**17bJ**	-1037.952229	-1037.929774	-1037.928830	-1038.006576	0.005427	3.405494	0.10%
**17bK**	-1037.953621	-1037.930410	-1037.929466	-1038.009994	0.002009	1.260667	3.88%
**17bL**	-1037.955175	-1037.932115	-1037.931171	-1038.010661	0.001342	0.842118	7.87%

**17bM**	-1037.955170	-1037.932116	-1037.931172	-1038.010586	0.001417	0.889181	7.26%

E, *E*′, *H*, *G*: total energy, total energy with zero point energy (*ZPE*), enthalpy, and Gibbs free energy

Figure S20. Main conformers of **17b** in NMR calculation.

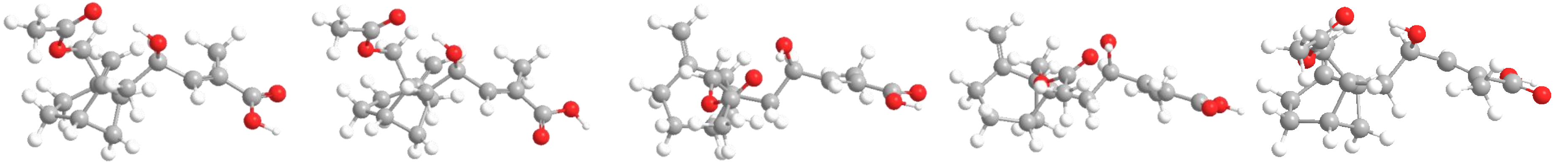

17bA **17bB** **17bC** **17bD** **17bE**

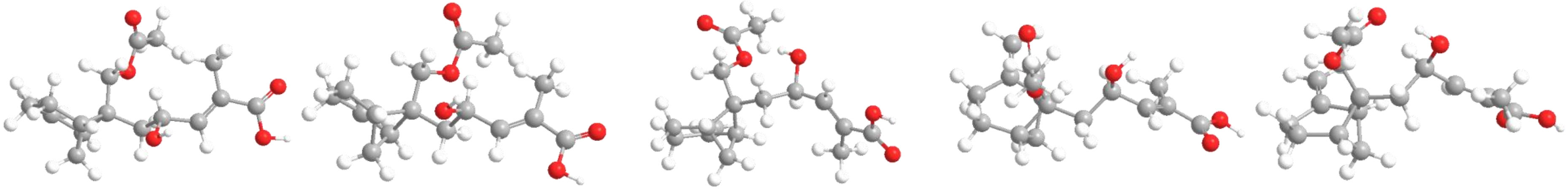

17bF **17bG** **17bH** **17bI** **17bJ**

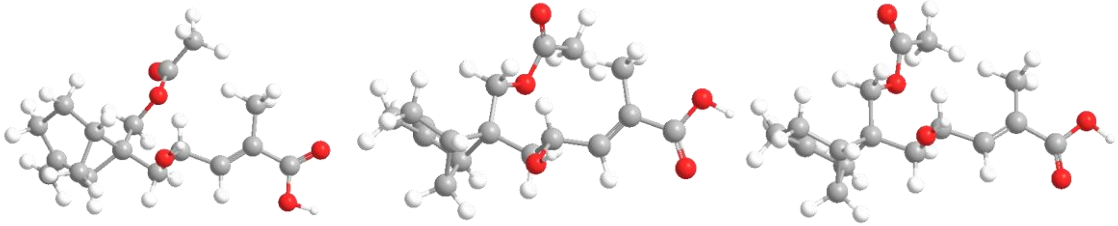

17bK **17bL** **17bM**

Section S7. Calculational details for 18

S7.1. Computational details for 18 (ECD)

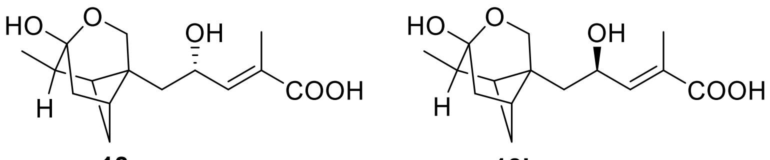

Conformation search based on molecular mechanics with MMFF force fields were performed for **18a** and **18b** gave 15 and 15 stable conformers with populations higher than 1%, respectively. All these conformers were further optimized by the density functional theory method at the B3LYP/6-31G(d) level by Gaussian 16 program package. The ECD were calculated using density functional theory (TDDFT) at B3LYP/6-31+G(d) level in methanol with IEFPCM model. The calculated ECD curves were all generated using SpecDis 1.71 with σ = 0.30 eV, UV shift -15 nm, respectively.

Figure S21. Comparison of the calculated ECD spectra for possible structure with the experimental spectrum of **18** in methanol with PCM model.

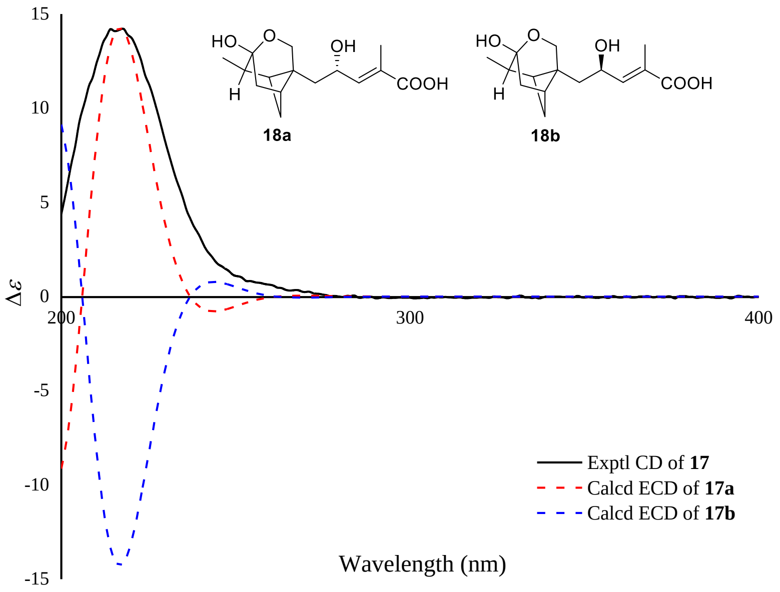

Table S20. Energy analysis for conformers of **18aA–18aO** at B3LYP/6-31G(d) level in the gas phase

Species	*E′*=*E*+*ZPE*	*E*	*H*	*G*	*ΔG*	*ΔE*(kcal/mol)	*PE*%
**18aA**	-960.561201	-960.541471	-960.540527	-960.610254	0.000720	0.451807	12.44%
**18aB**	-960.561263	-960.541510	-960.540566	-960.610561	0.000413	0.259161	17.22%
**18aC**	-960.561689	-960.541944	-960.540999	-960.610974	0	0	26.68%
**18aD**	-960.561723	-960.541980	-960.541036	-960.610728	0.000246	0.154367	20.56%
**18aE**	-960.557772	-960.538018	-960.537074	-960.606245	0.004729	2.967492	0.18%
**18aF**	-960.557257	-960.537630	-960.536686	-960.605515	0.005459	3.425574	0.08%
**18aG**	-960.559111	-960.539480	-960.538535	-960.607200	0.003774	2.368221	0.49%
**18aH**	-960.560759	-960.541057	-960.540113	-960.609676	0.001298	0.814507	6.74%
**18aI**	-960.557588	-960.538003	-960.537059	-960.605721	0.005253	3.296307	0.10%
**18aJ**	-960.560599	-960.540903	-960.539959	-960.609559	0.001415	0.887926	5.95%
**18aK**	-960.560580	-960.540882	-960.539938	-960.609372	0.001602	1.005270	4.88%
**18aL**	-960.557152	-960.537439	-960.536495	-960.605472	0.005502	3.452557	0.08%
**18aM**	-960.560295	-960.540578	-960.539634	-960.609233	0.001741	1.092494	4.22%
**18aN**	-960.558630	-960.538988	-960.538044	-960.606814	0.004160	2.610440	0.32%
**18aO**	-960.557747	-960.538275	-960.537331	-960.605187	0.005787	3.631397	0.06%

E, *E′*, *H*, *G*: total energy, total energy with zero point energy (*ZPE*), enthalpy, and Gibbs free energy

Figure S22. Main conformers of **18a** in ECD calculation.

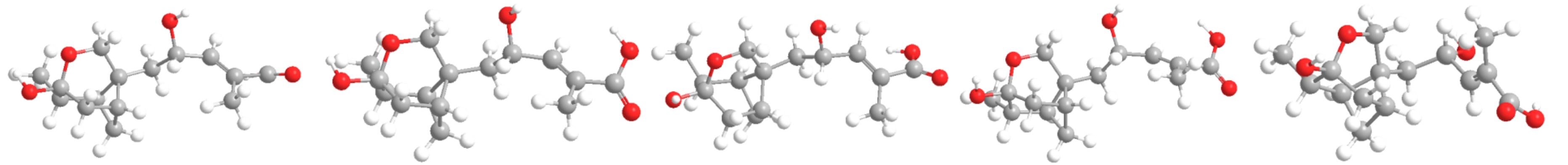

18aA **18aB** **18aC** **18aD** **18aE**

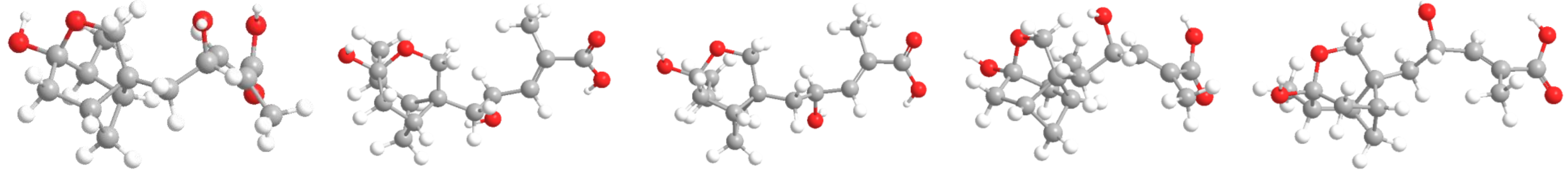

18aF **18aG** **18aH** **18aI** **18aJ**

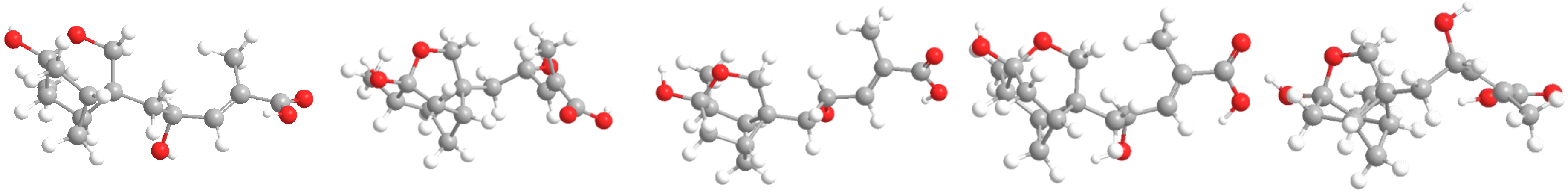

18aK **18aL** **18aM** **18aN** **18aO**

Table S21. Energy analysis for conformers of **18bA–18bO** at B3LYP/6-31G(d) level in the gas phase

Species	*E′*=*E*+*ZPE*	*E*	*H*	*G*	*ΔG*	*ΔE*(kcal/mol)	*PE*%
**18bA**	-960.561660	-960.541916	-960.540972	-960.610939	0	0	30.89%
**18bB**	-960.561246	-960.541492	-960.540548	-960.610729	0.000210	0.131777	24.73%
**18bC**	-960.557222	-960.537523	-960.536579	-960.605340	0.005599	3.513426	0.08%
**18bD**	-960.557619	-960.537922	-960.536977	-960.605701	0.005238	3.286895	0.12%
**18bE**	-960.560570	-960.540854	-960.539910	-960.609889	0.001050	0.658885	10.15%
**18bF**	-960.557739	-960.538128	-960.537183	-960.605867	0.005072	3.182728	0.14%
**18bG**	-960.561247	-960.541528	-960.540584	-960.610209	0.000730	0.458082	14.25%
**18bH**	-960.560178	-960.540471	-960.539527	-960.609523	0.001416	0.888553	6.89%
**18bI**	-960.558765	-960.539080	-960.538136	-960.607242	0.003697	2.319903	0.61%
**18bJ**	-960.560659	-960.540957	-960.540013	-960.609619	0.001320	0.828313	7.63%
**18bK**	-960.558814	-960.539218	-960.538274	-960.606926	0.004013	2.518196	0.44%
**18bL**	-960.559207	-960.539530	-960.538586	-960.607645	0.003294	2.067016	0.94%
**18bM**	-960.557203	-960.537608	-960.536663	-960.605332	0.005607	3.518446	0.08%
**18bN**	-960.559270	-960.539651	-960.538707	-960.607454	0.003485	2.186871	0.77%
**18bO**	-960.559280	-960.539695	-960.538751	-960.608480	0.002459	1.543046	2.28%

E, *E′*, *H*, *G*: total energy, total energy with zero point energy (*ZPE*), enthalpy, and Gibbs free energy

Figure S23. Main conformers of **18b** in ECD calculation.

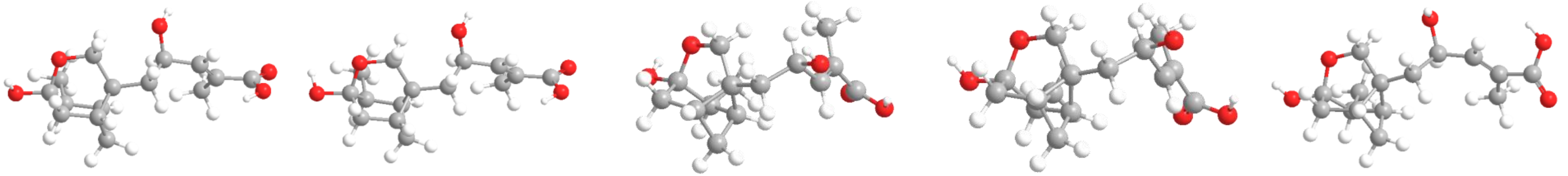

18bA **18bB** **18bC** **18bD** **18bE**

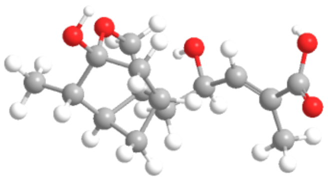

18bF

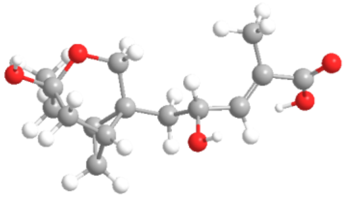

18bG

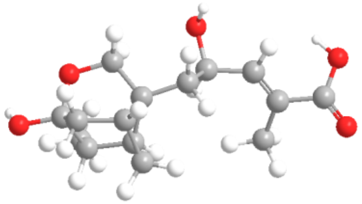

18bH

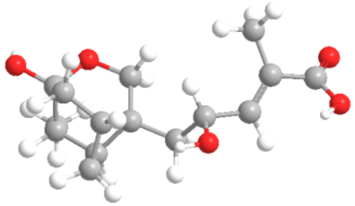

18bI

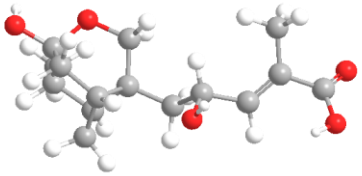

18bJ

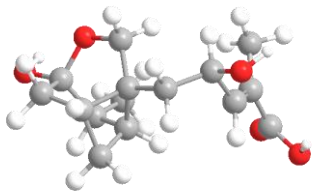

18bK

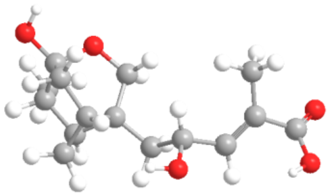

18bL

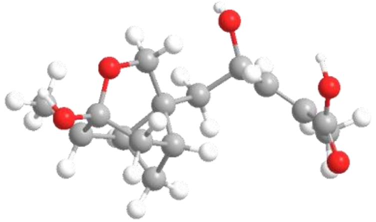

18bM

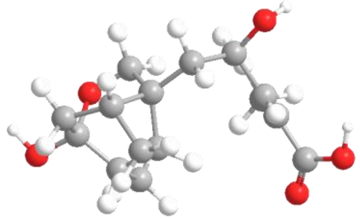

18bN

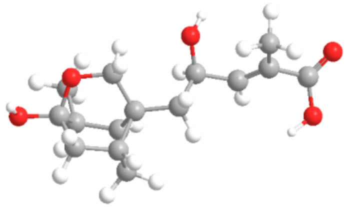

18bO

References

(1) Hehre, W. J. A guide to molecular mechanics and quantum chemical calculations. *Wavefunction, Inc., Irvine, CA* **2003**, 1-812.

(2) Shao, Y.; Molnar, L. F.; Jung, Y.; Kussmann, J.; Ochsenfeld, C.; Brown, S. T.; Gilbert, A. T. B.; Slipchenko, L. V.; Levchenko, S. V.; O'Neill, D. P.; DiStasio Jr, R. A.; Lochan, R. C.; Wang, T.; Beran, G. J. O.; Besley, N. A.; Herbert, J. M.; Yeh Lin, C.; Van Voorhis, T.; Hung Chien, S.; Sodt, A.; Steele, R. P.; Rassolov, V. A.; Maslen, P. E.; Korambath, P. P.; Adamson, R. D.; Austin, B.; Baker, J.; Byrd, E. F. C.; Dachsel, H.; Doerksen, R. J.; Dreuw, A.; Dunietz, B. D.; Dutoi, A. D.; Furlani, T. R.; Gwaltney, S. R.; Heyden, A.; Hirata, S.; Hsu, C.-P.; Kedziora, G.; Khalliulin, R. Z.; Klunzinger, P.; Lee, A. M.; Lee, M. S.; Liang, W.; Lotan, I.; Nair, N.; Peters, B.; Proynov, E. I.; Pieniazek, P. A.; Min Rhee, Y.; Ritchie, J.; Rosta, E.; David Sherrill, C.; Simmonett, A. C.; Subotnik, J. E.; Lee Woodcock Iii, H.; Zhang, W.; Bell, A. T.; Chakraborty, A. K.; Chipman, D. M.; Keil, F. J.; Warshel, A.; Hehre, W. J.; Schaefer Iii, H. F.; Kong, J.; Krylov, A. I.; Gill, P. M. W.; Head-Gordon, M. *Phys. Chem. Chem. Phys.* **2006,** *8*, 3172-3191.

(3) Frisch, M. J.; Trucks, G. W.; Schlegel, H. B.; Scuseria, G. E.; Robb, M. A.; Cheeseman, J. R.; Scalmani, G.; Barone, V.; Petersson, G. A.; Nakatsuji, H.; Li, X.; Caricato, M.; Marenich, A.; Bloino, J.; Janesko, B. G.; Gomperts, R.; Mennucci, B.; Hratchian, H. P.; Ortiz, J. V.; Izmaylov, A. F.; Sonnenberg, J. L.; Williams-Young, D.; Ding, F.; Lipparini, F.; Egidi, F.; Goings, J.; Peng, B.; Petrone, A.; Henderson, T.; Ranasinghe, D.; Zakrzewski, V. G.; Gao, J.; Rega, N.; Zheng, G.; Liang, W.; Hada, M.; Ehara, M.; Toyota, K.; Fukuda, R.; Hasegawa, J.; Ishida, M.; Nakajima, T.; Honda, Y.; Kitao, O.; Nakai, H.; Vreven, T.; Throssell, K.; Montgomery, J. A.; Jr.; Peralta, J. E.; Ogliaro, F.; Bearpark, M.; Heyd, J. J.; Brothers, E.; Kudin, K. N.; Staroverov, V. N.; Keith, T.; Kobayashi, R.; Normand, J.; Raghavachari, K.; Rendell, A.; Burant, J. C.; Iyengar, S. S.; Tomasi, J.; Cossi, M.; Millam, J. M.; Klene, M.; Adamo, C.; Cammi, R.; Ochterski, J. W.; Martin, R. L.; Morokuma, K.; Farkas, O.; Foresman, J. B.; Fox, D. J. *Gaussian 16, Revision B.01* Gaussian, Inc. Wallingford CT, **2016**.

(4) Grimblat, N.; Zanardi, M. M.; Sarotti, A. M. *J. Org. Chem.* **2015,** *80*, 12526-12534.

(5) Bruhn, T.; Schaumlöffel, A.; Hemberger, Y.; Bringmann, G. *Chirality* **2013,** *25*, 243-249.

Section S8. NMR and MS spectra for 1

Figure S24. ^{1}H NMR of compound **1** in CD_3OD

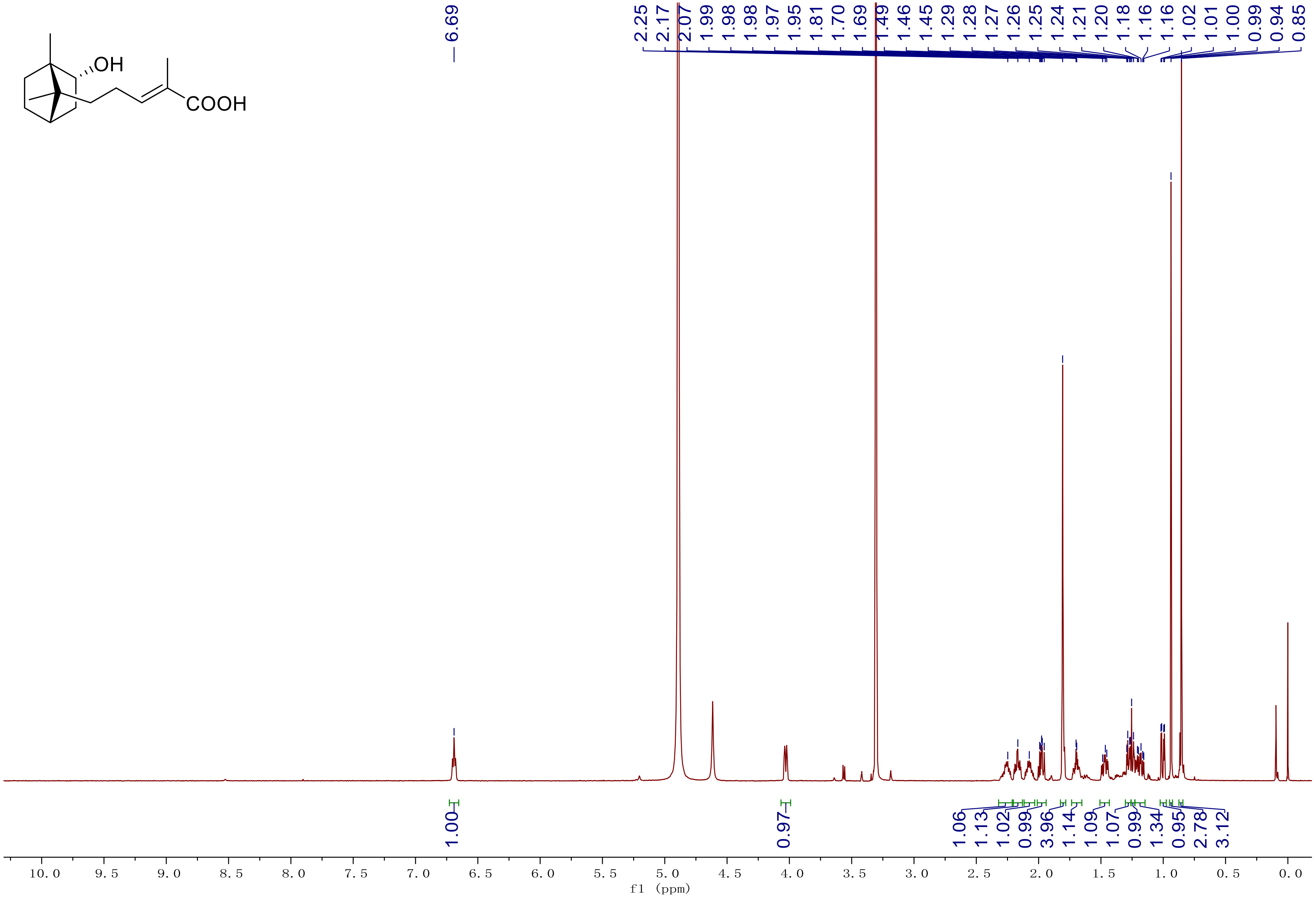

Figure S25. ^{13}C NMR and DEPT of compound **1** in CD_3OD

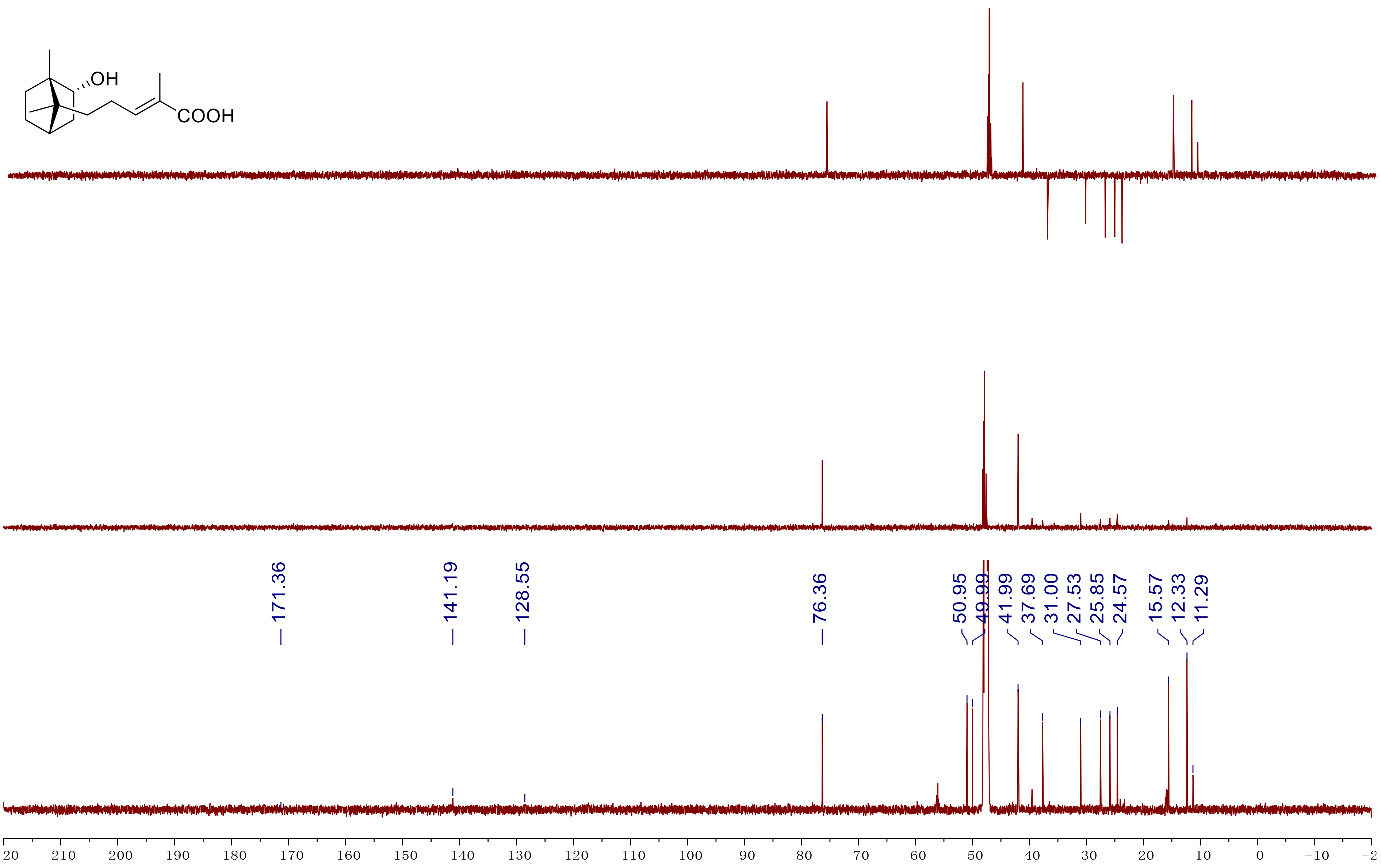

Figure S26. HSQC of compound **1** in CD_3OD

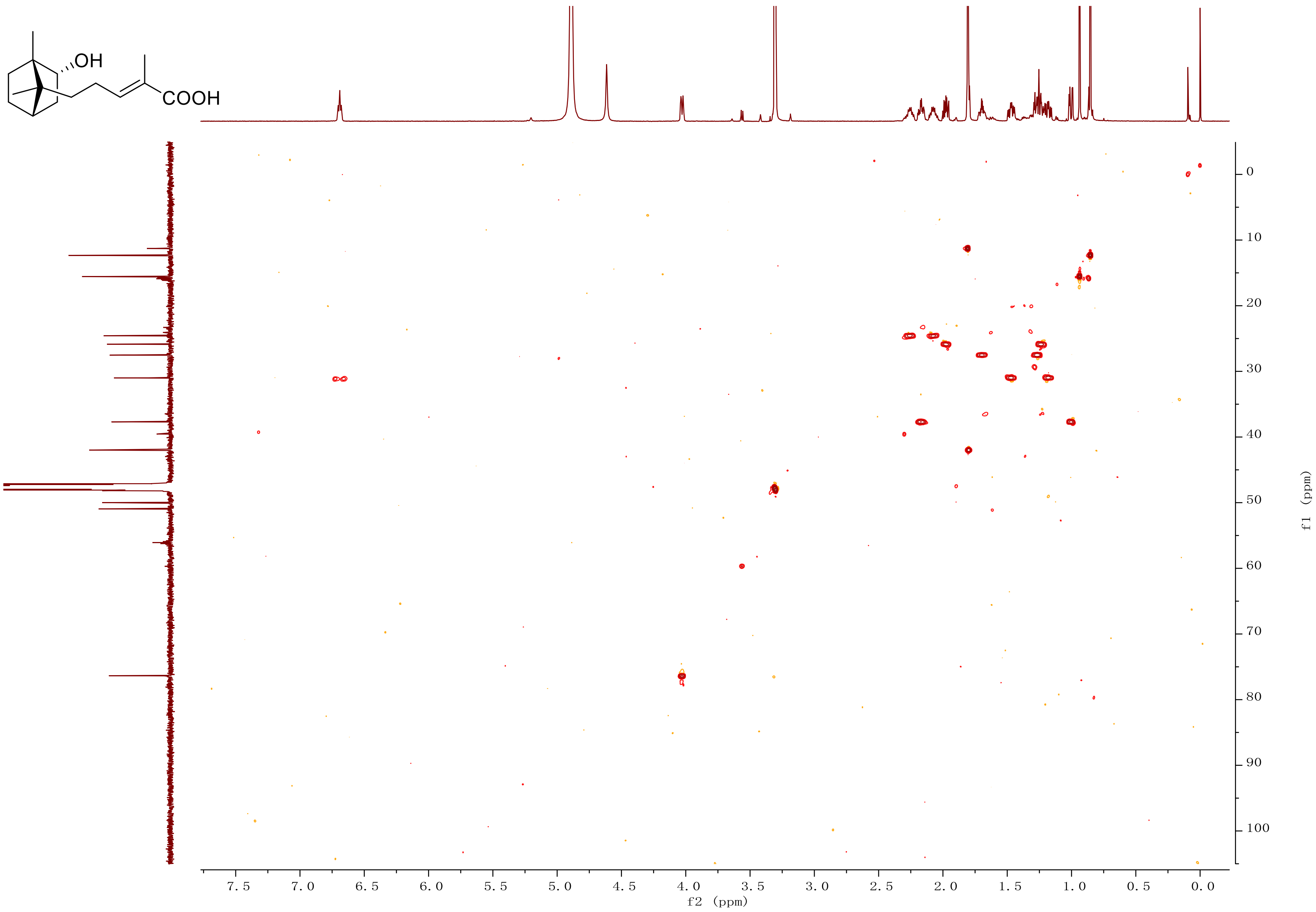

Figure S27. HMBC of compound **1** in CD_3OD

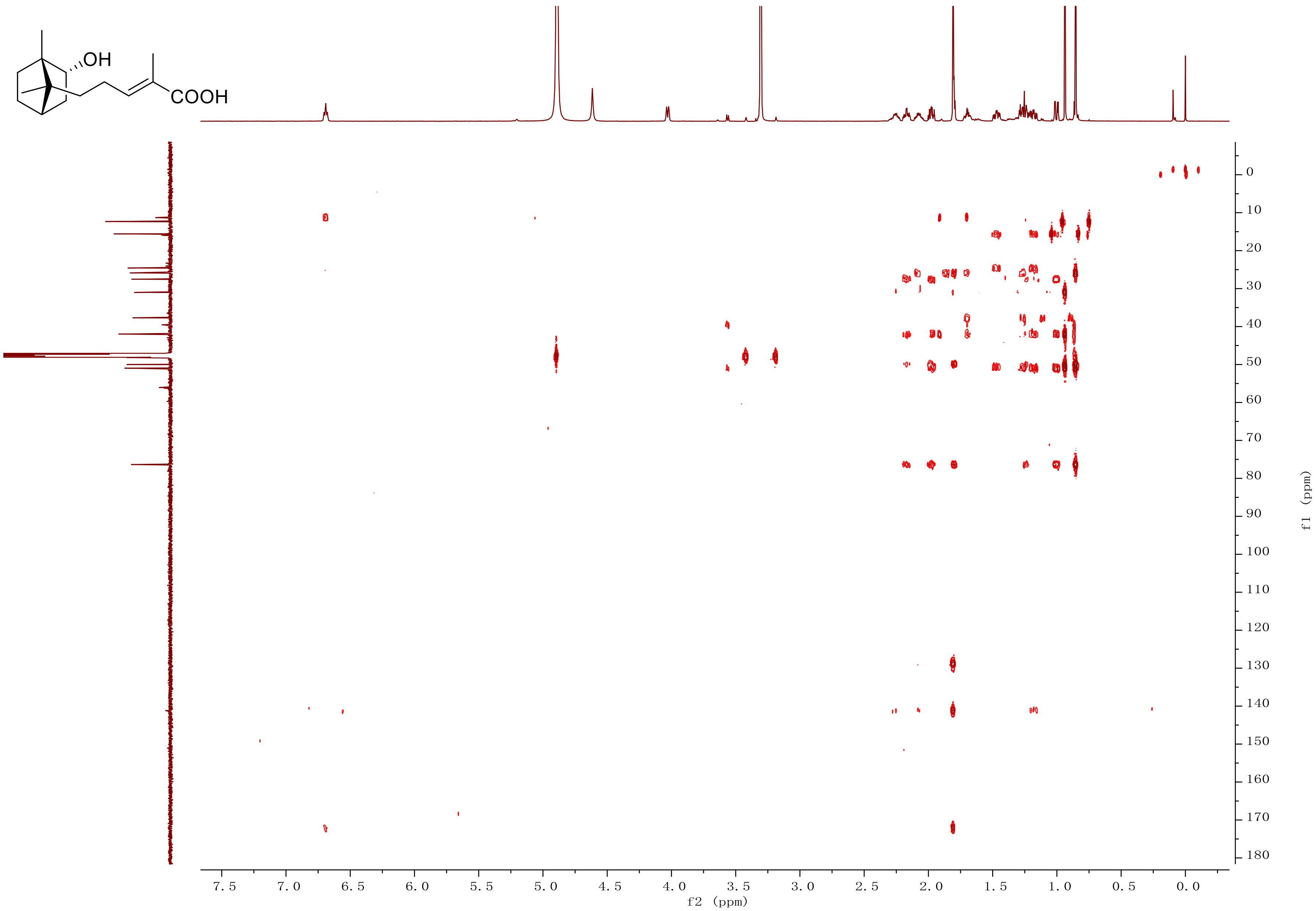

Figure S28. ^{1}H-^{1}H COSY of compound **1** in CD_3OD

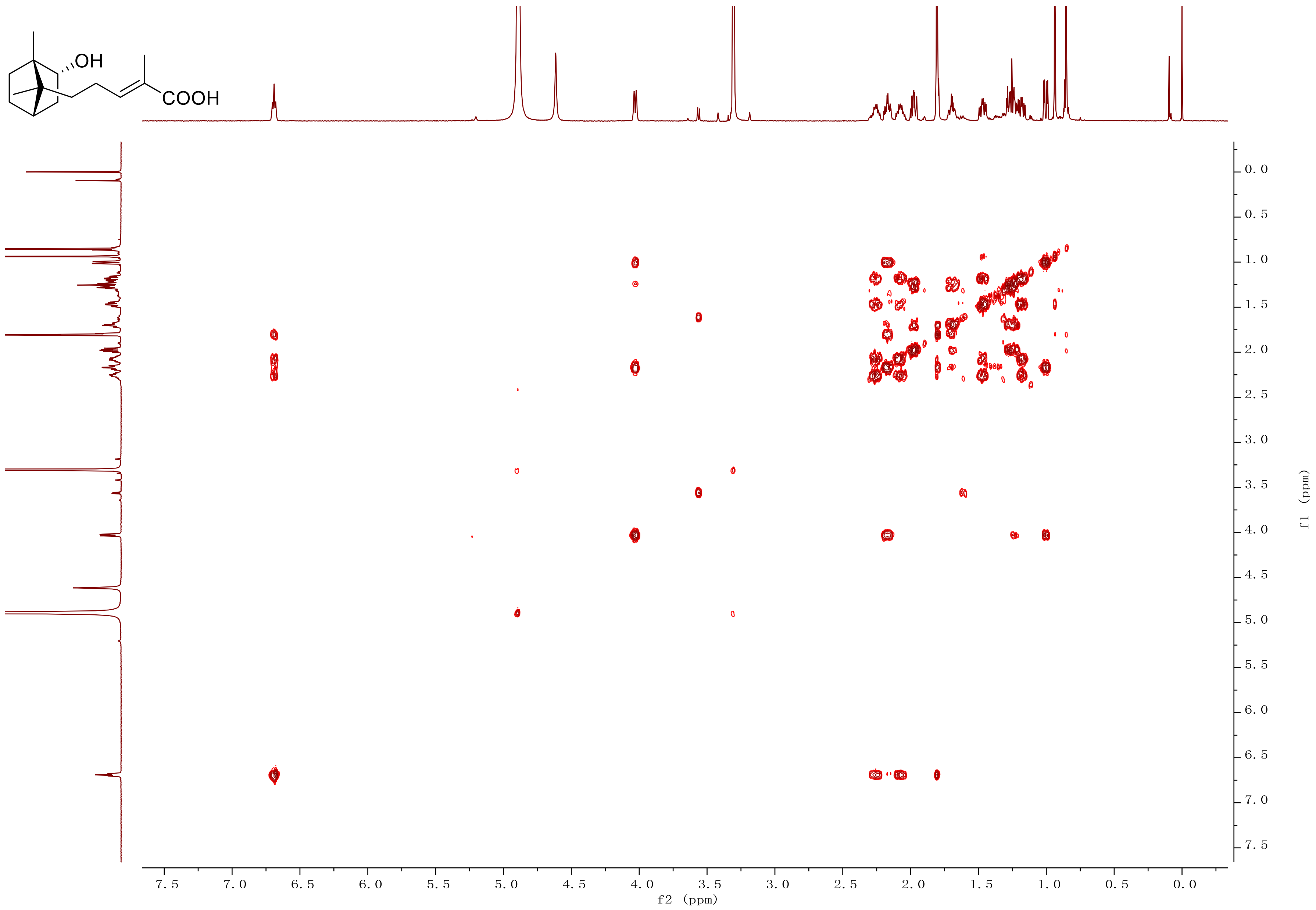

Figure S29. ROESY of compound **1** in CD_3OD

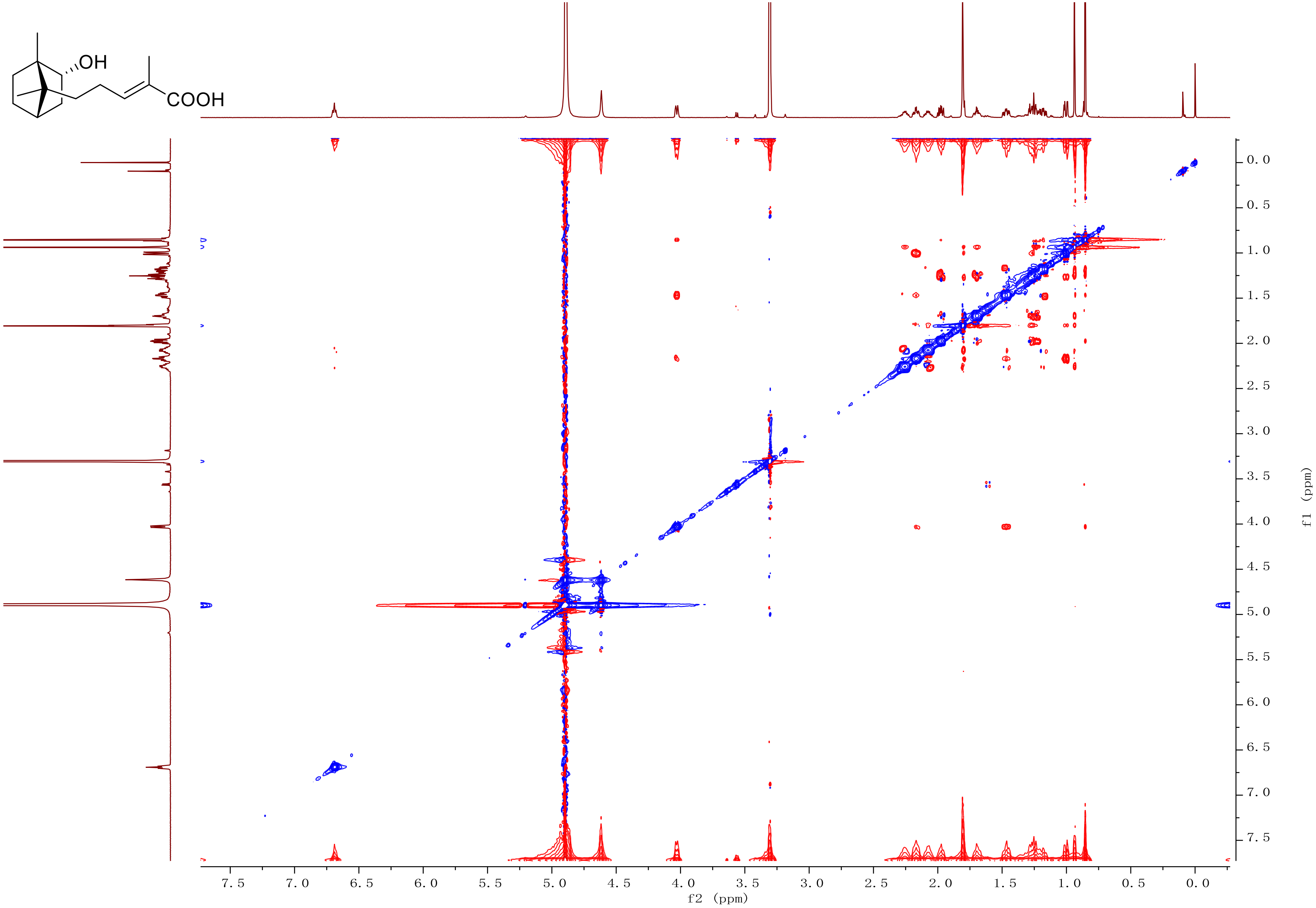

Figure S30. HR-ESIMS of compound **1**

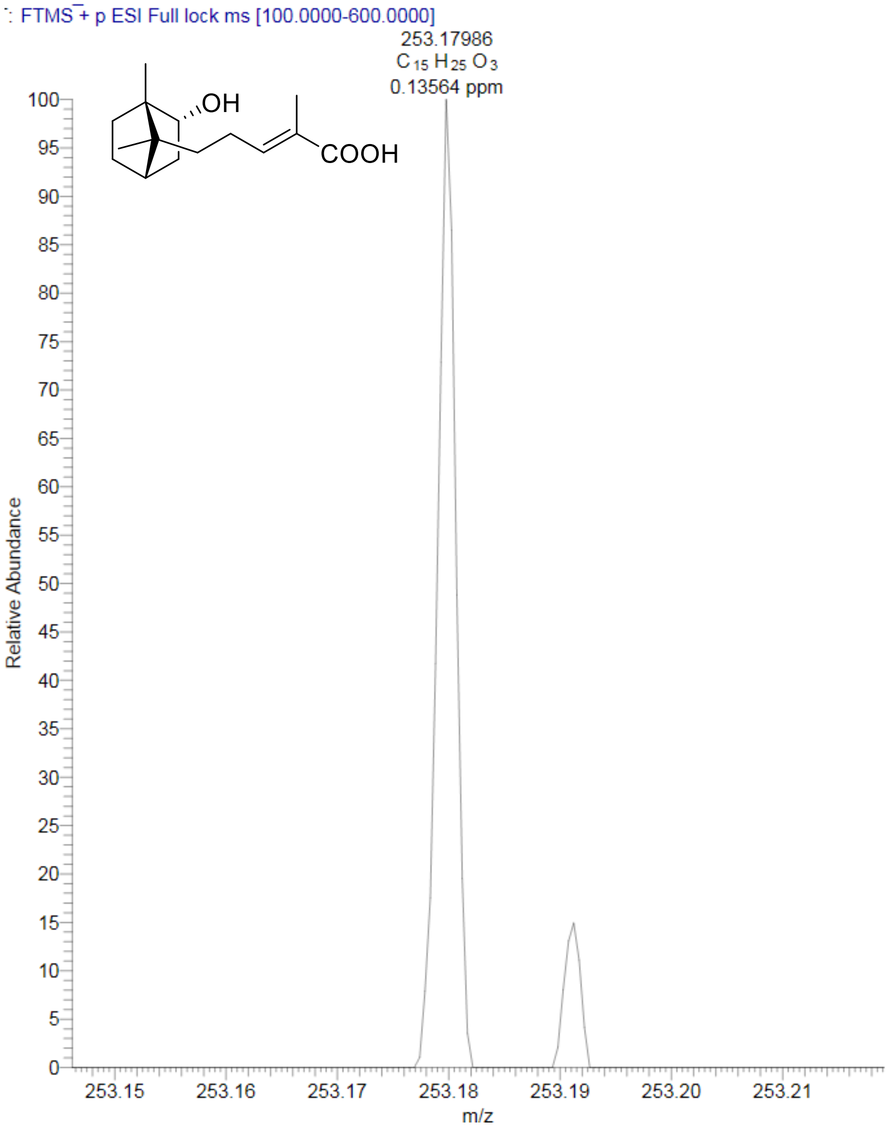

Section S9. NMR and MS spectra for 2

Figure S31. ^{1}H NMR of compound **2** in CD_3OD

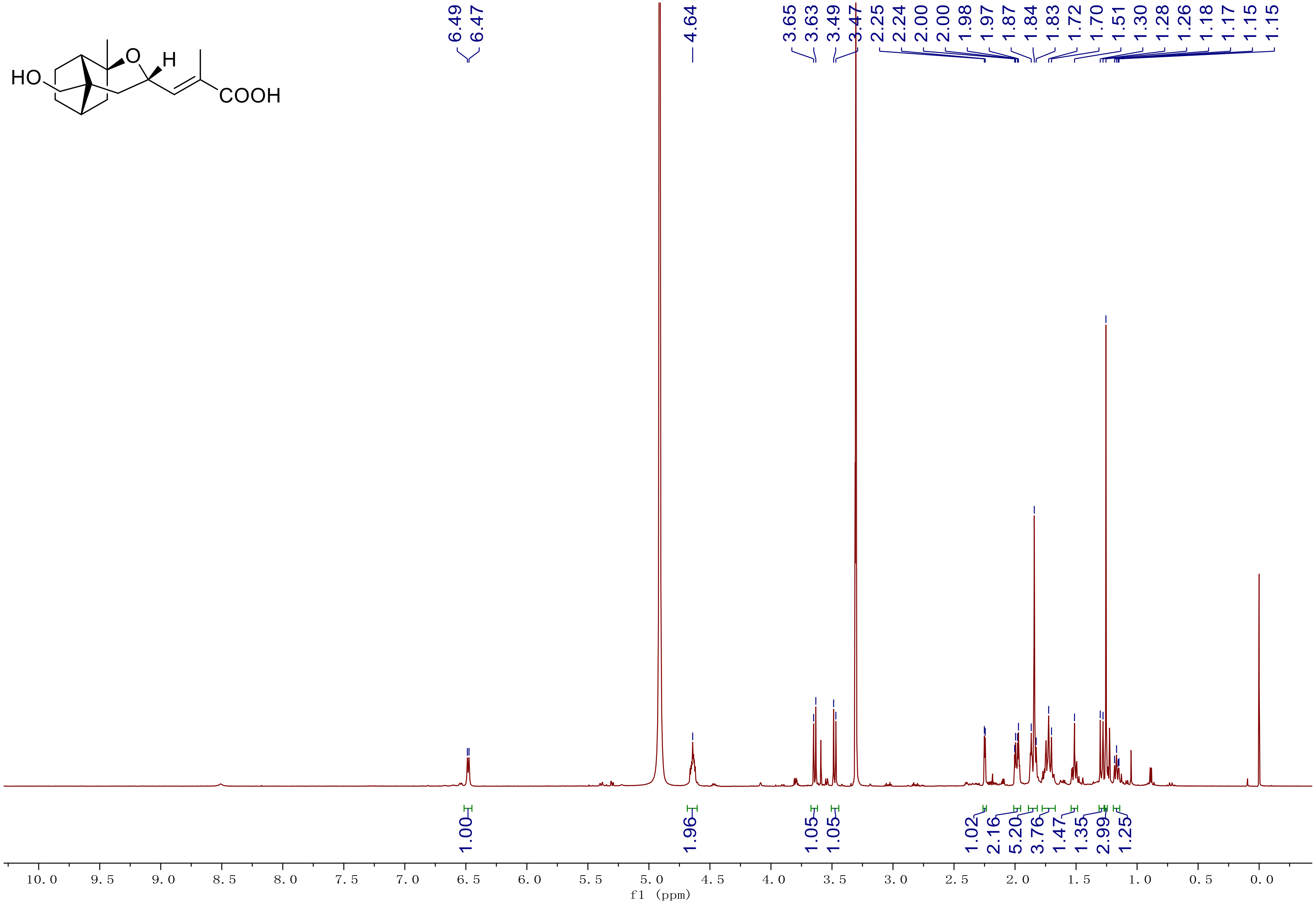

Figure S32. ^{13}C NMR and DEPT of compound **2** in CD_3OD

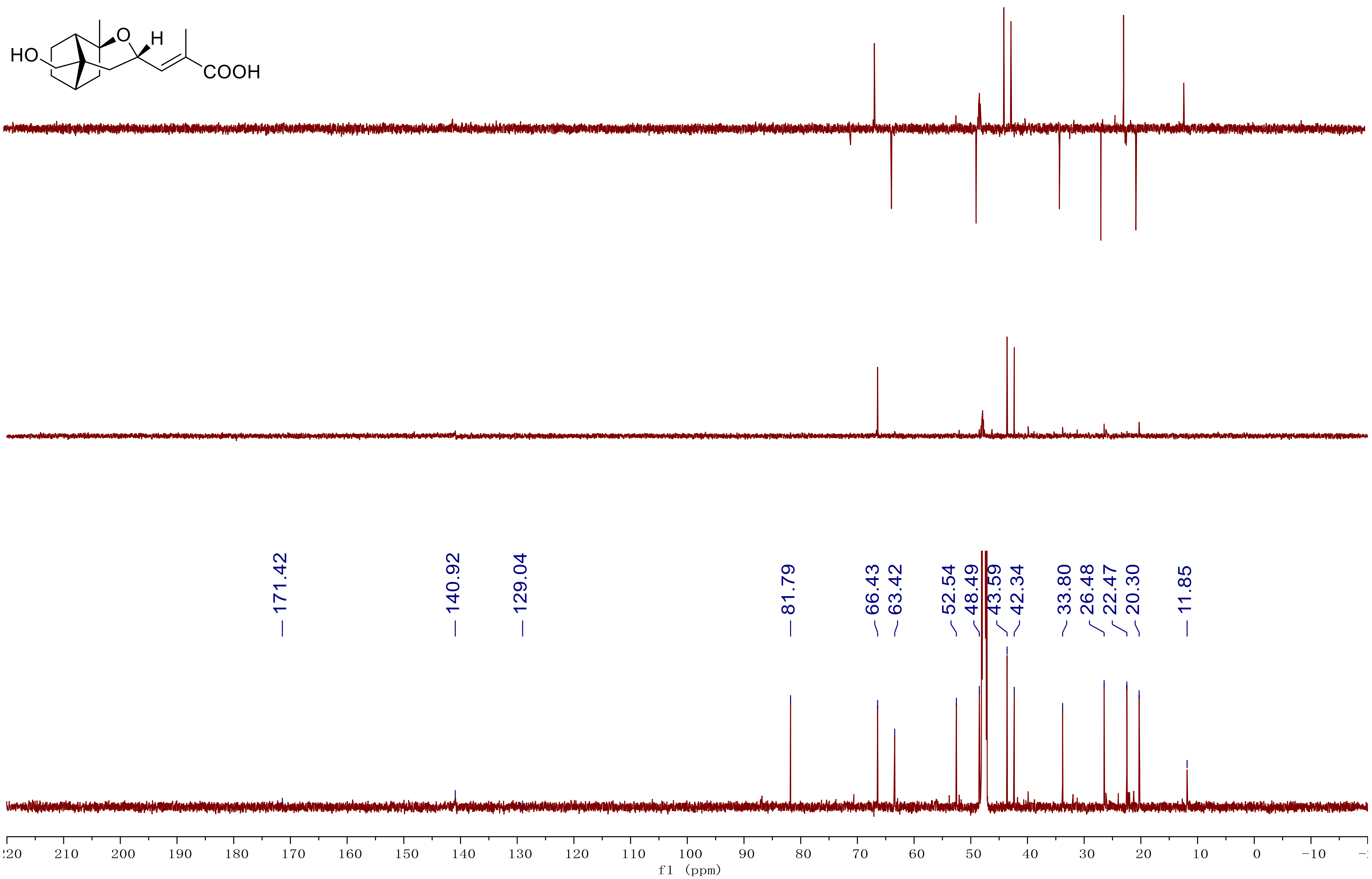

Figure S33. HSQC of compound **2** in CD_3OD

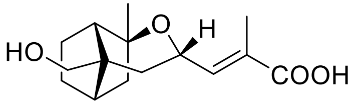

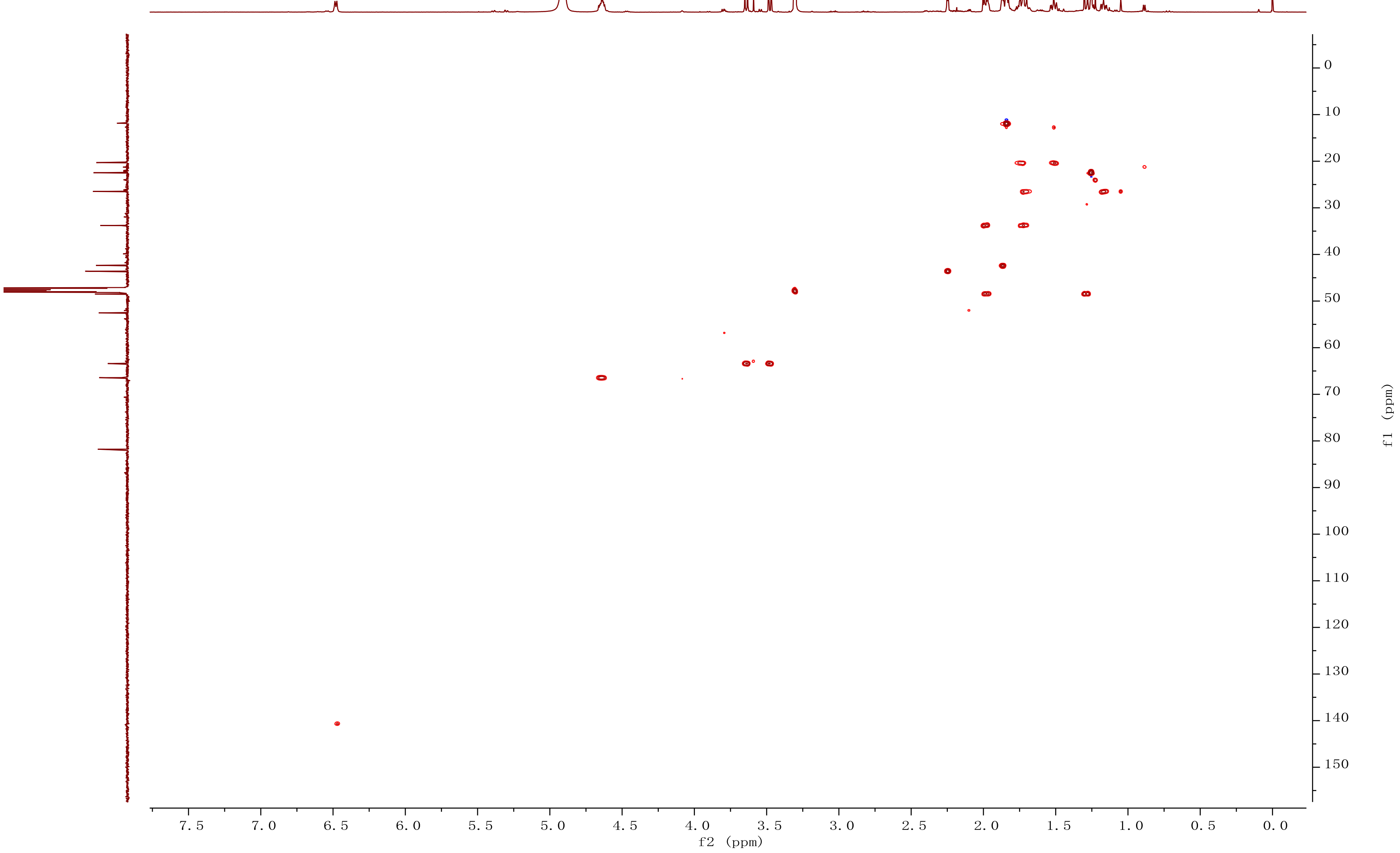

Figure S34. HMBC of compound **2** in CD_3OD

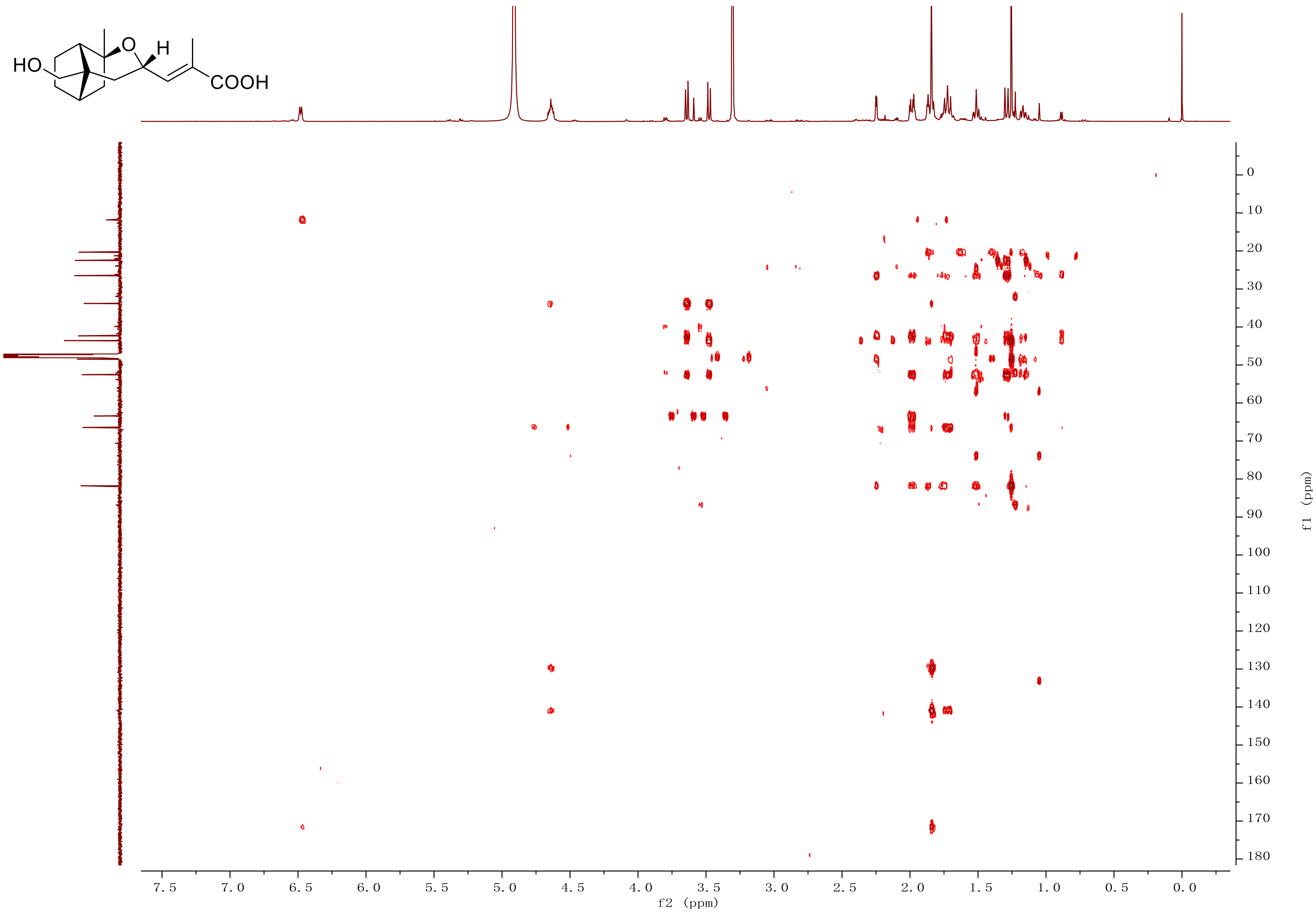

Figure S35. ^{1}H-^{1}H COSY of compound **2** in CD_3OD

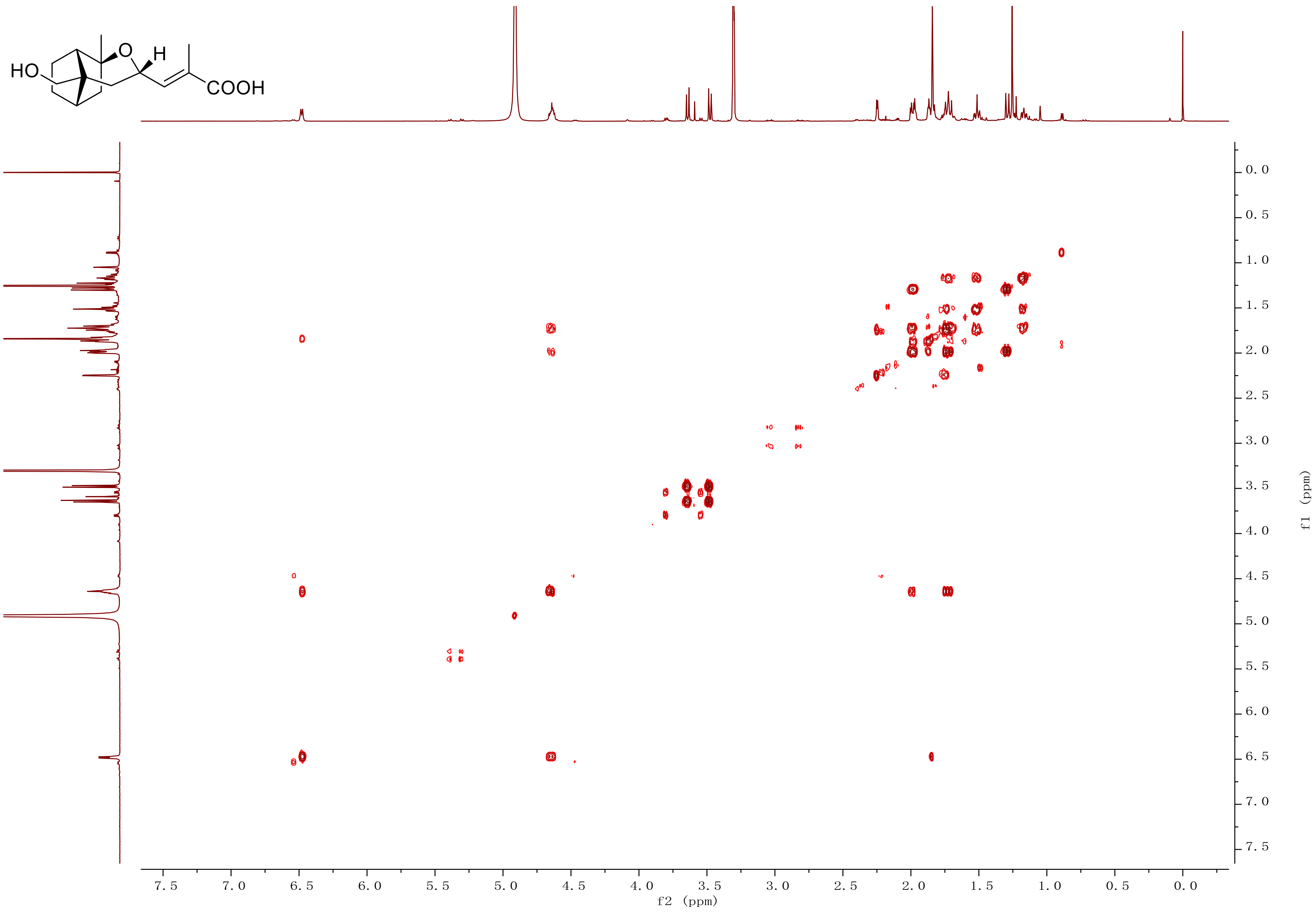

Figure S36. ROESY of compound **2** in CD_3OD

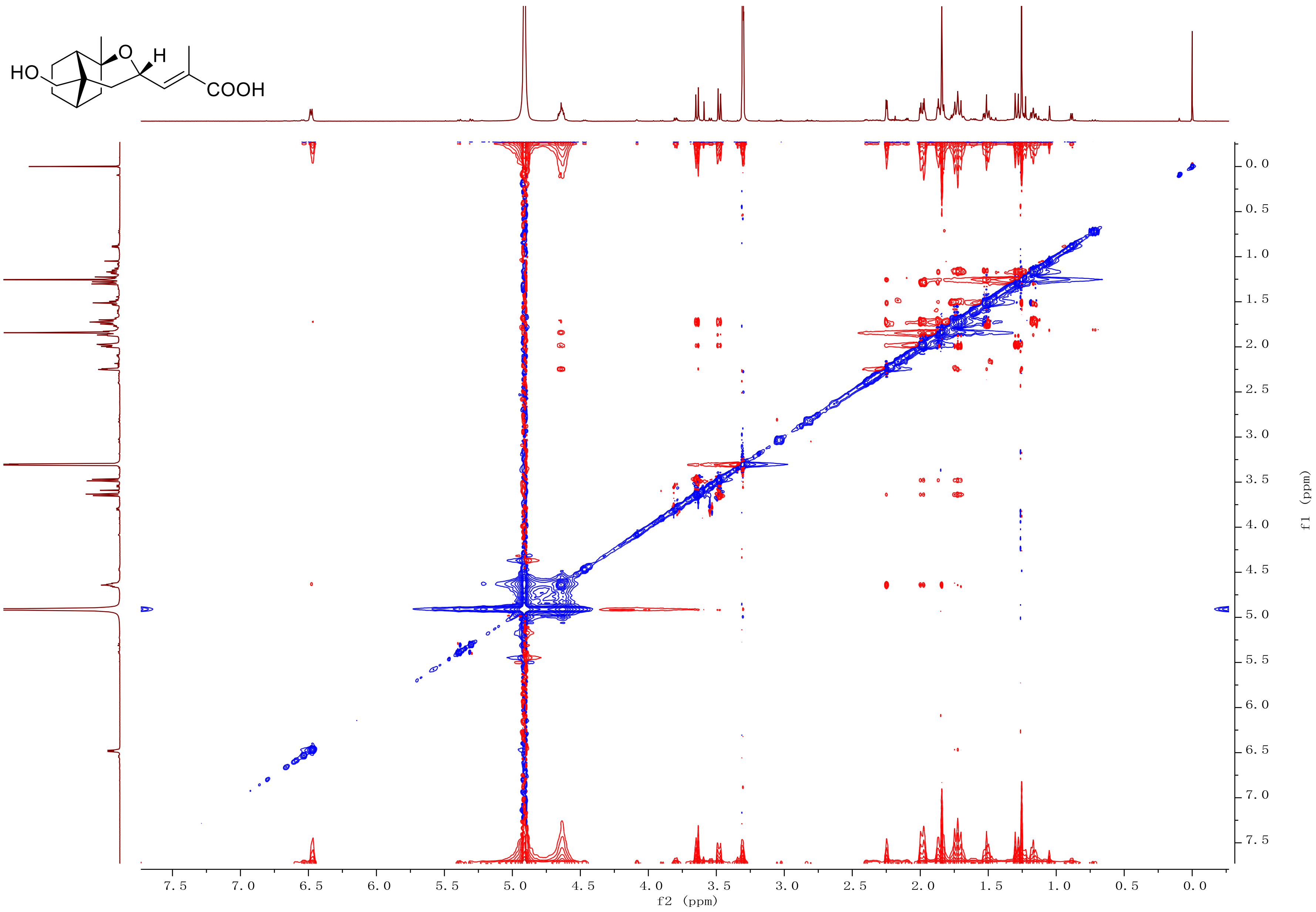

Figure S37. HR-ESIMS of compound **2**

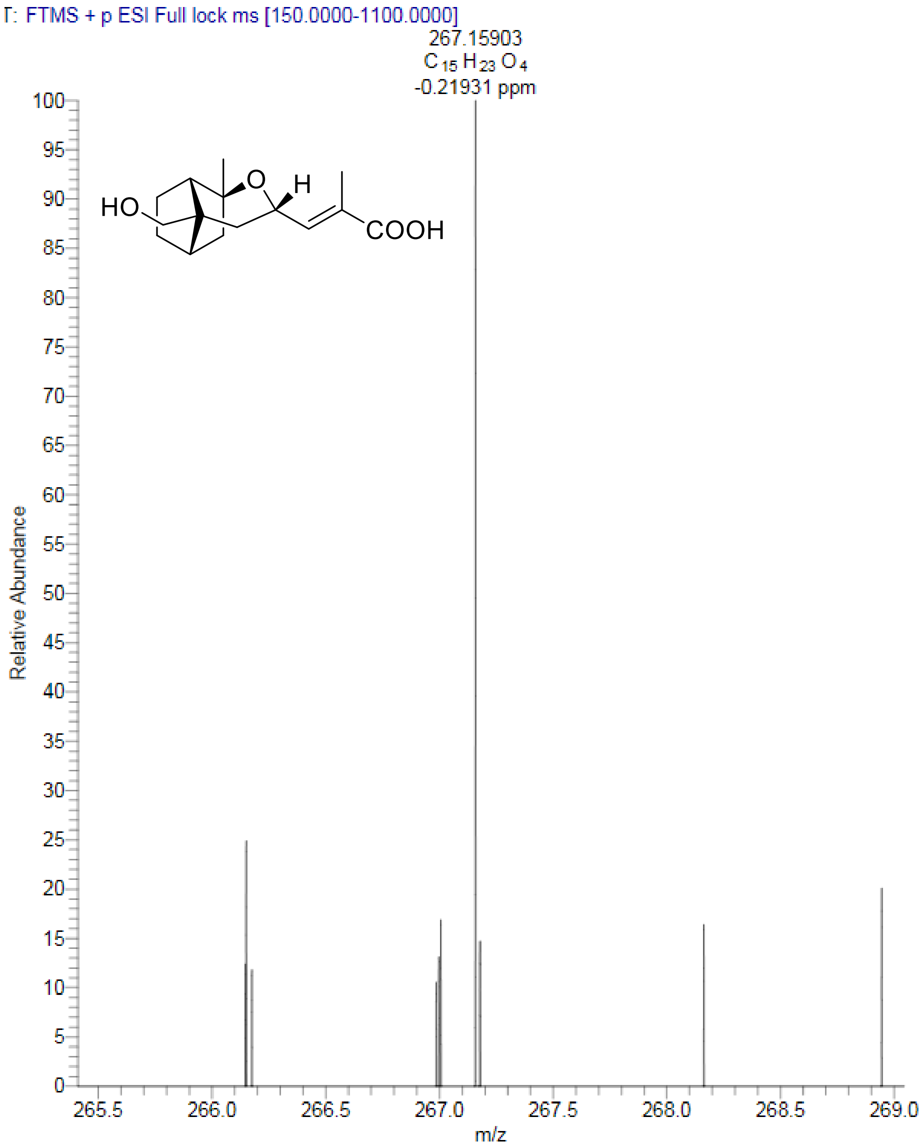

Section S10. NMR and MS spectra for 3

Figure S38. ^{1}H NMR of compound **3** in CD_3OD

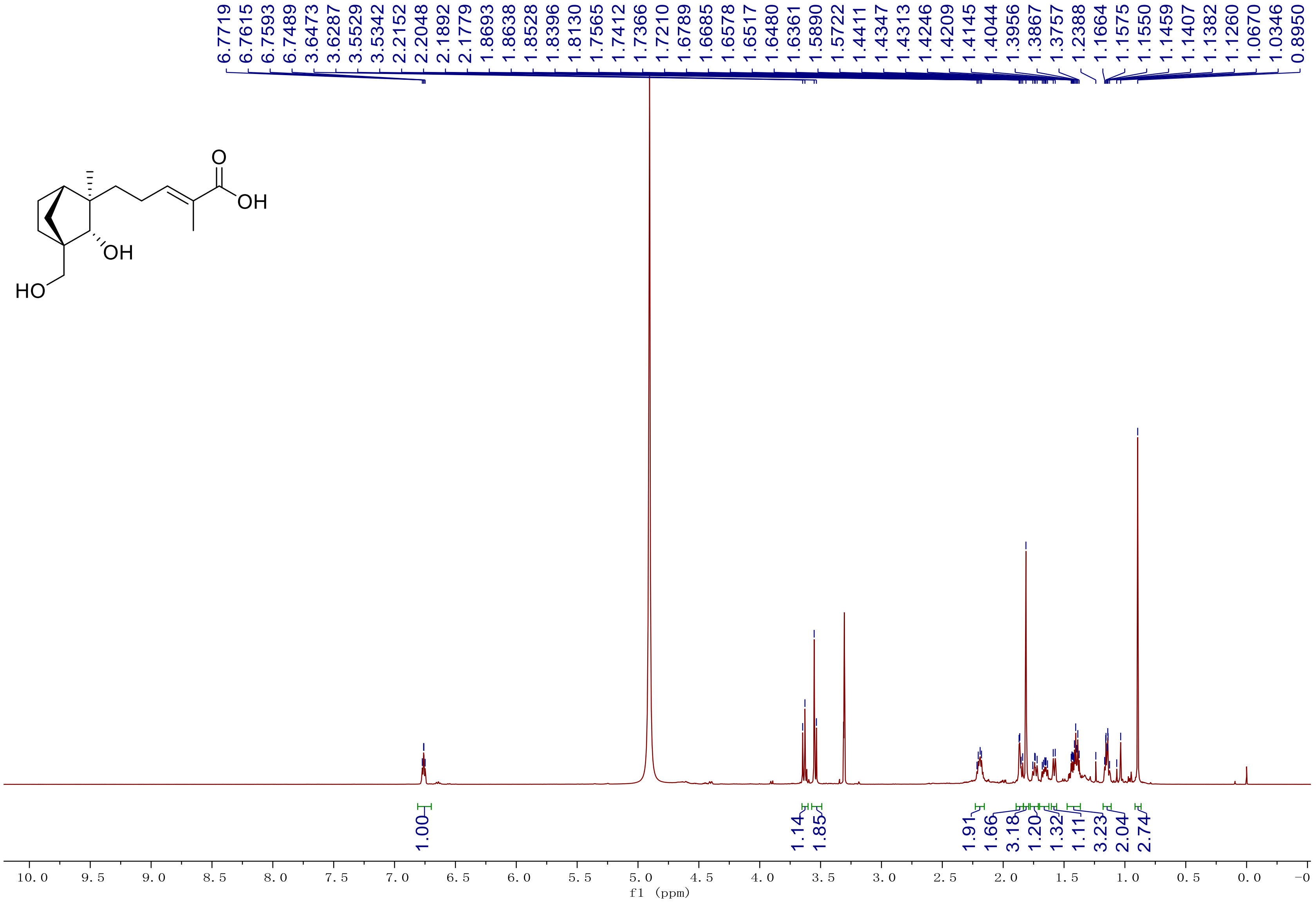

Figure S39. ^{13}C NMR and DEPT of compound **3** in CD_3OD

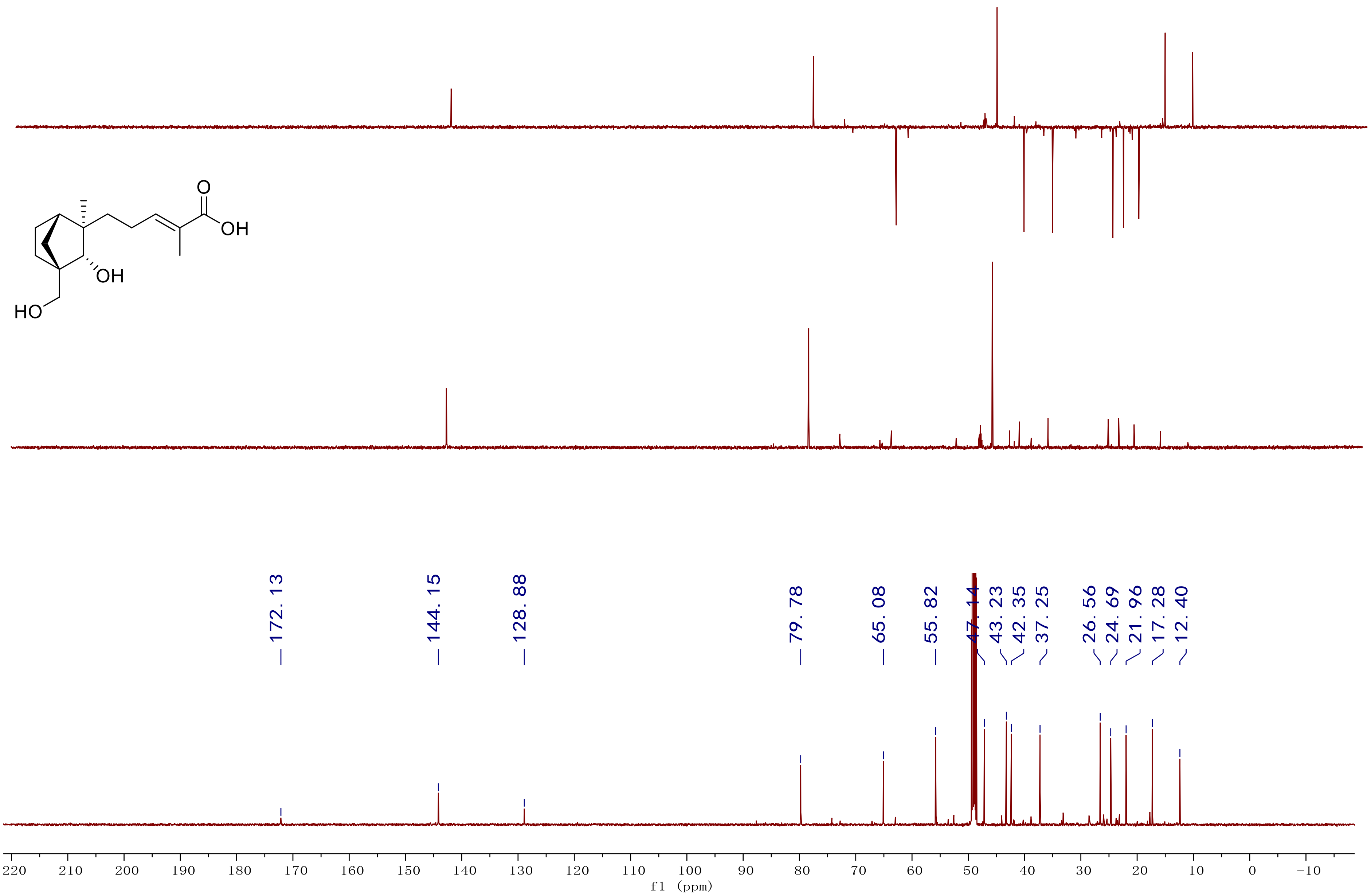

Figure S40. HSQC of compound **3** in CD_3OD

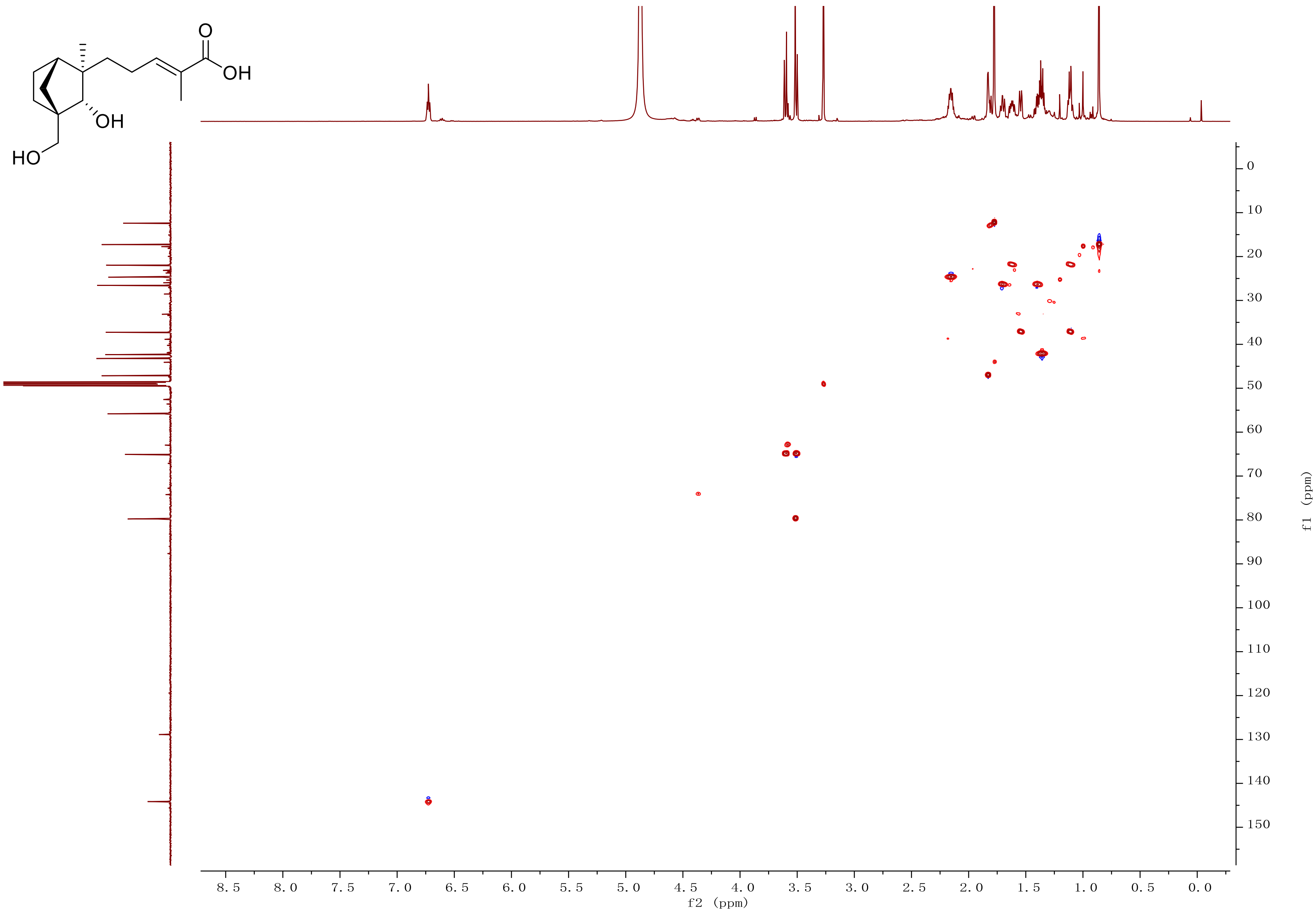

Figure S41. HMBC of compound **3** in CD_3OD

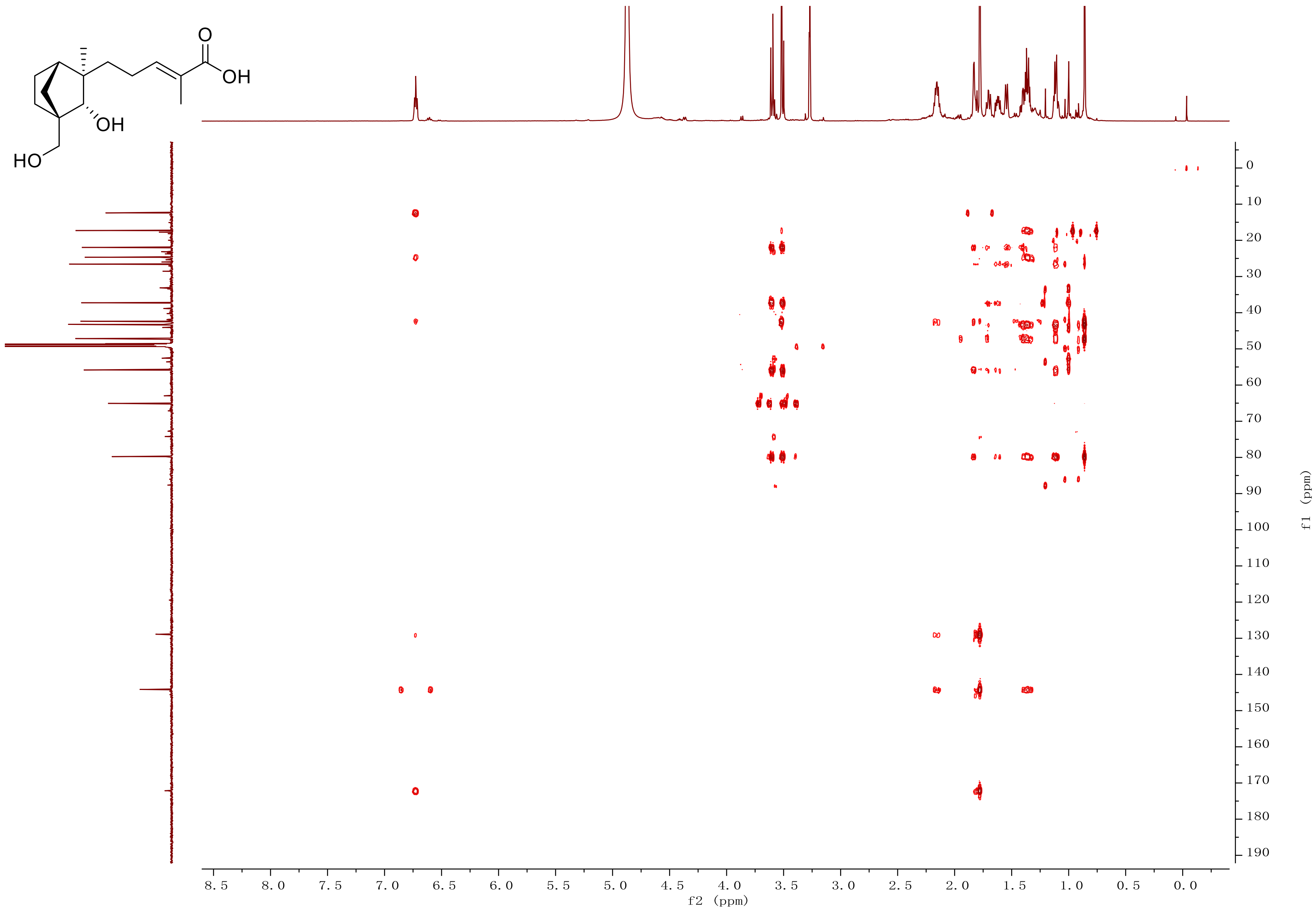

Figure S42. ^{1}H-^{1}H COSY of compound **3** in CD_3OD

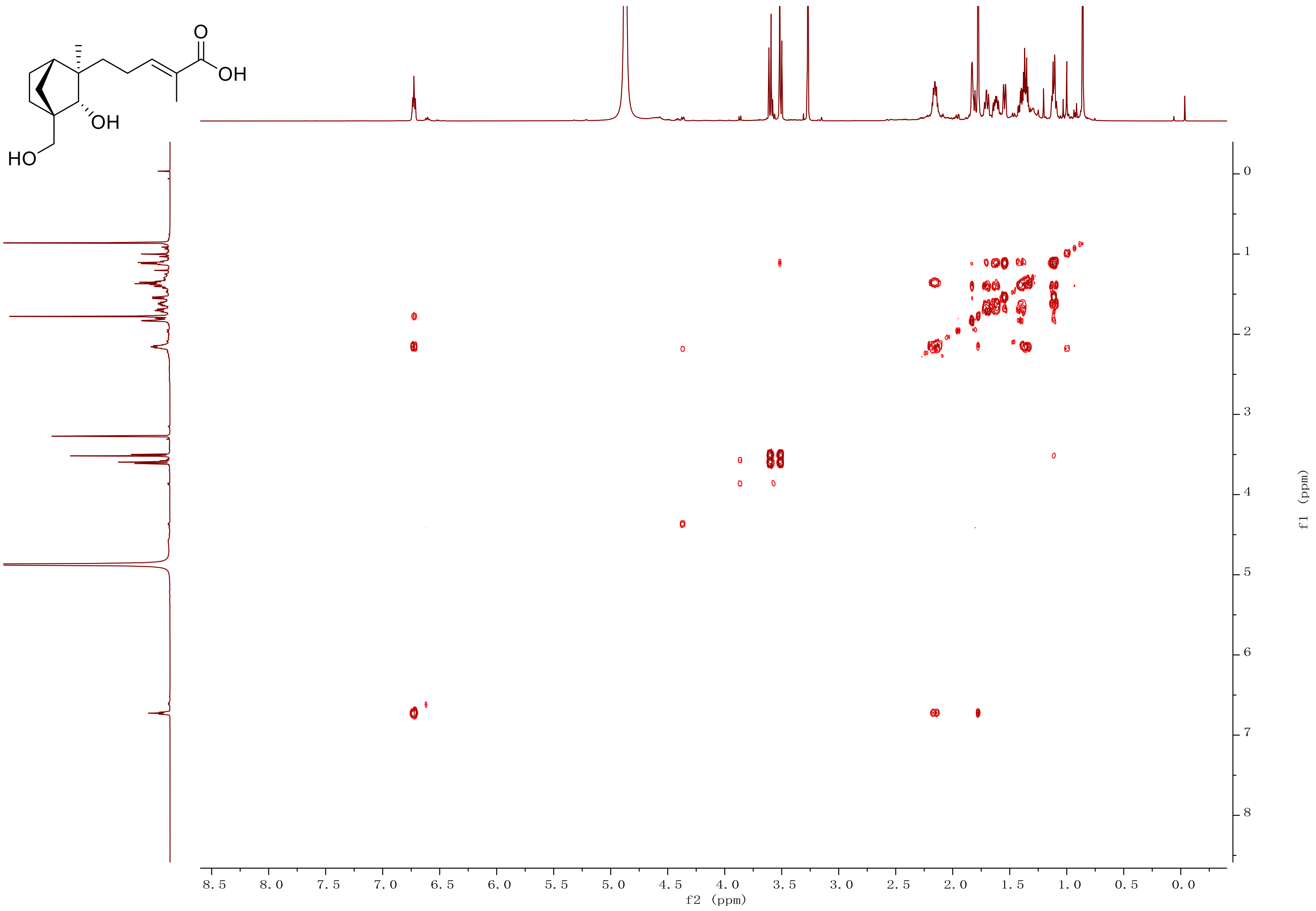

Figure S43. ROESY of compound **3** in CD_3OD

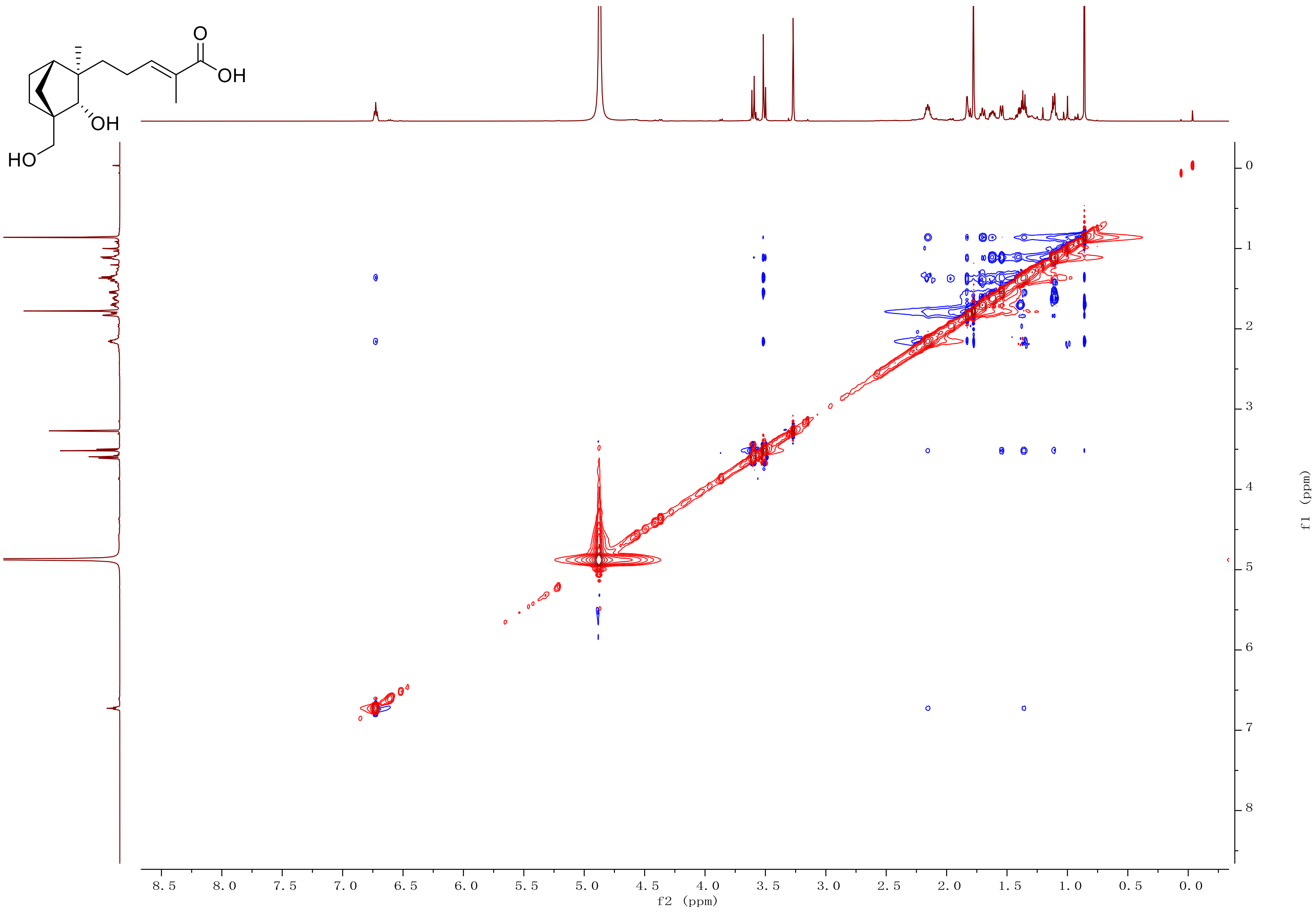

Figure S44. HR-ESIMS of compound **3**

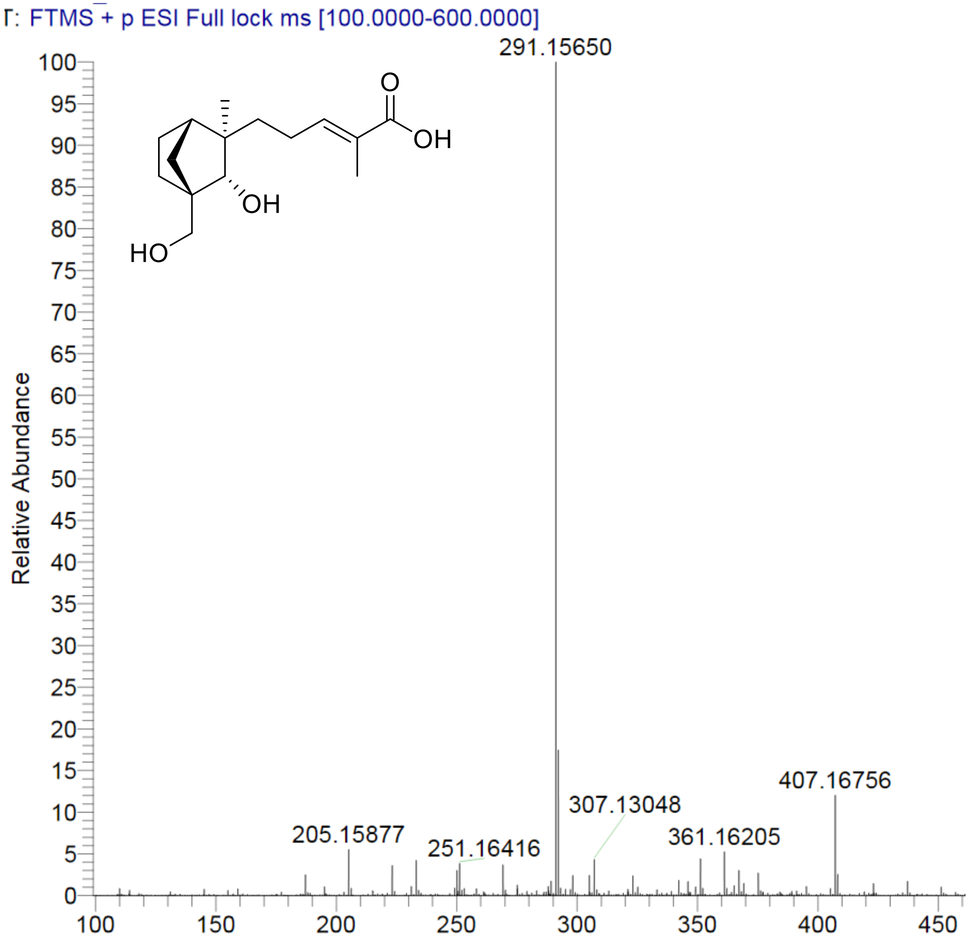

Section S11. NMR and MS spectra for 4

Figure S45. ^{1}H NMR of compound **4** in CD_3OD

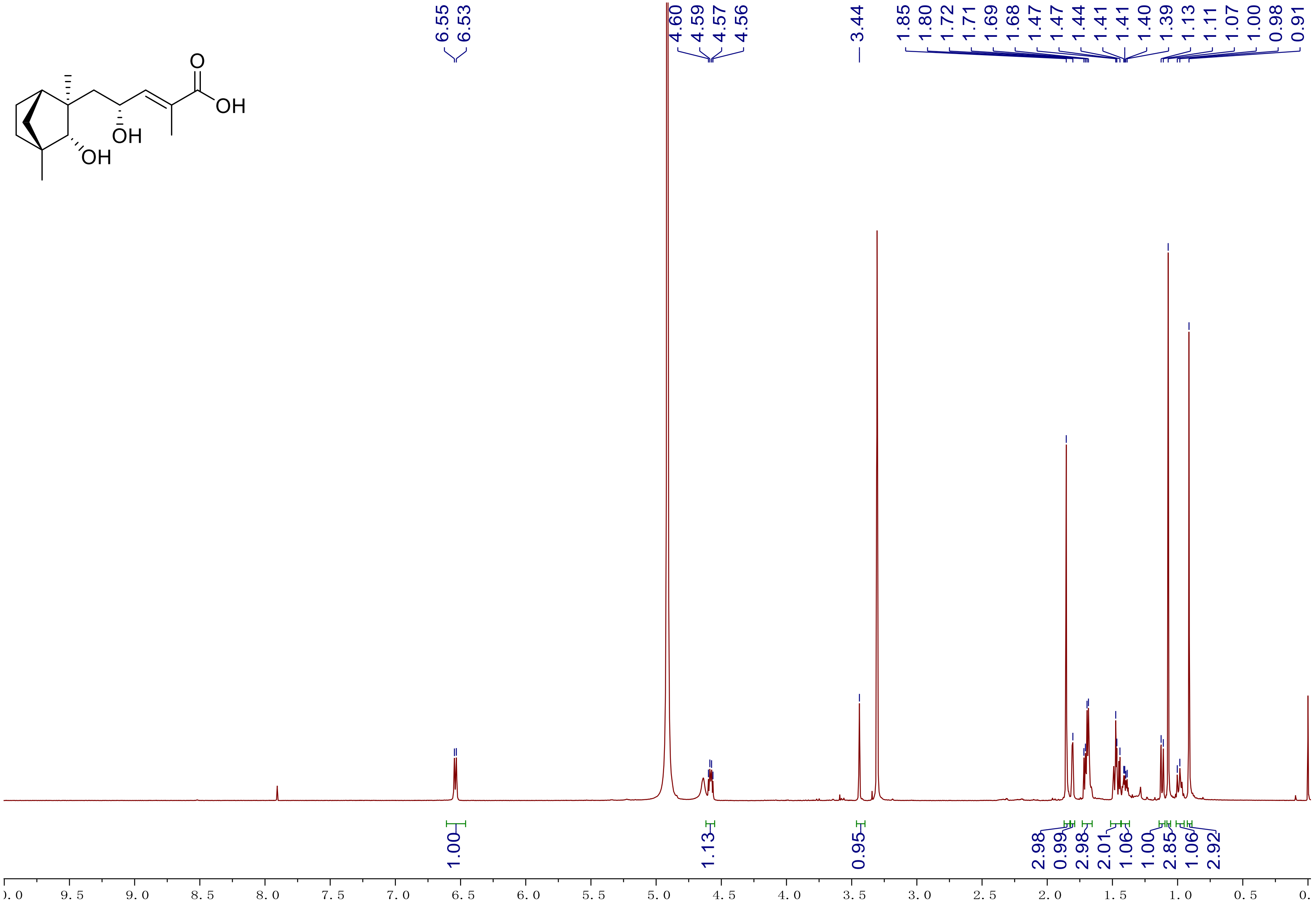

Figure S46. ^{13}C NMR and DEPT of compound **4** in CD_3OD

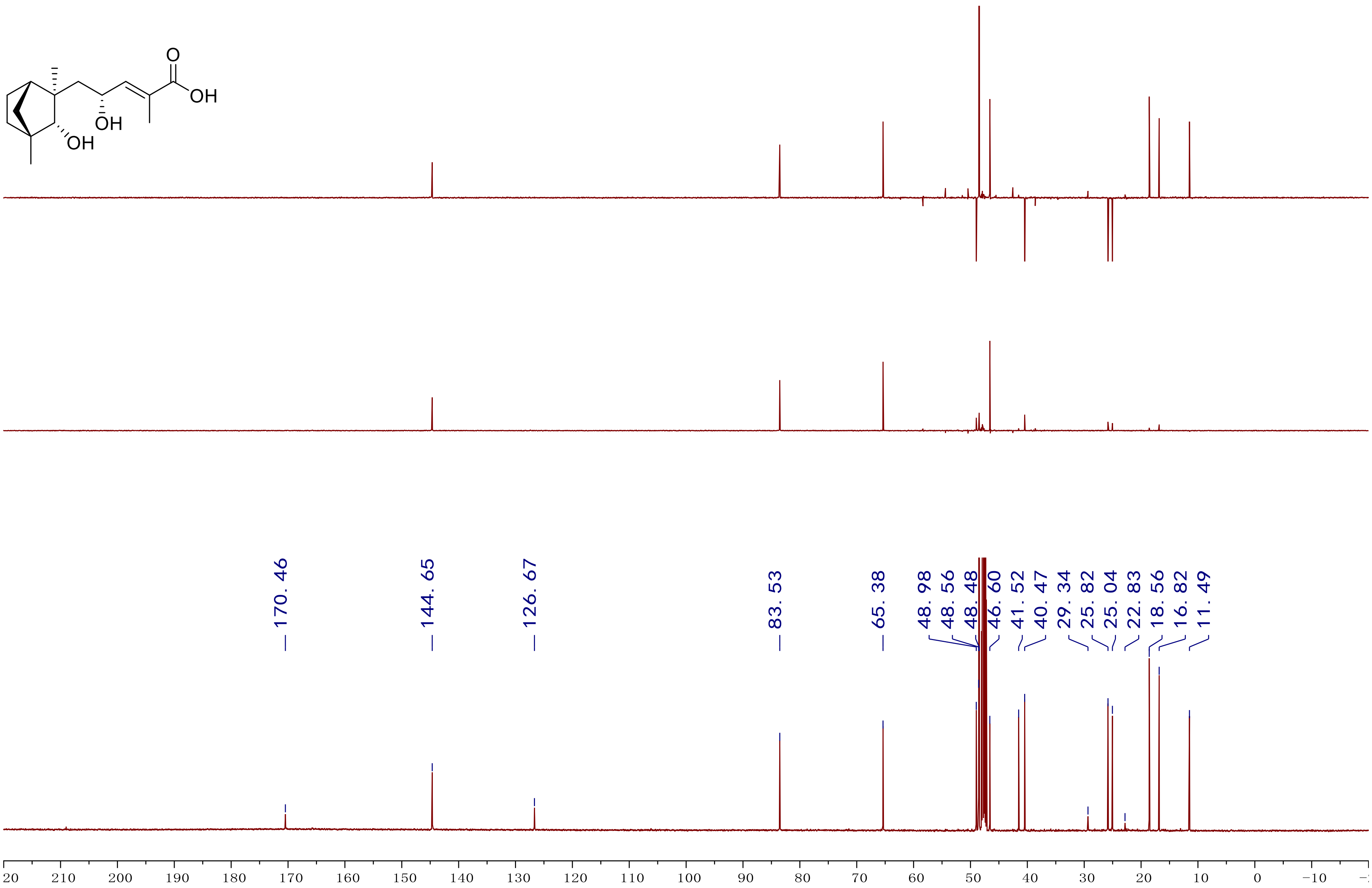

Figure S47. HSQC of compound **4** in CD_3OD

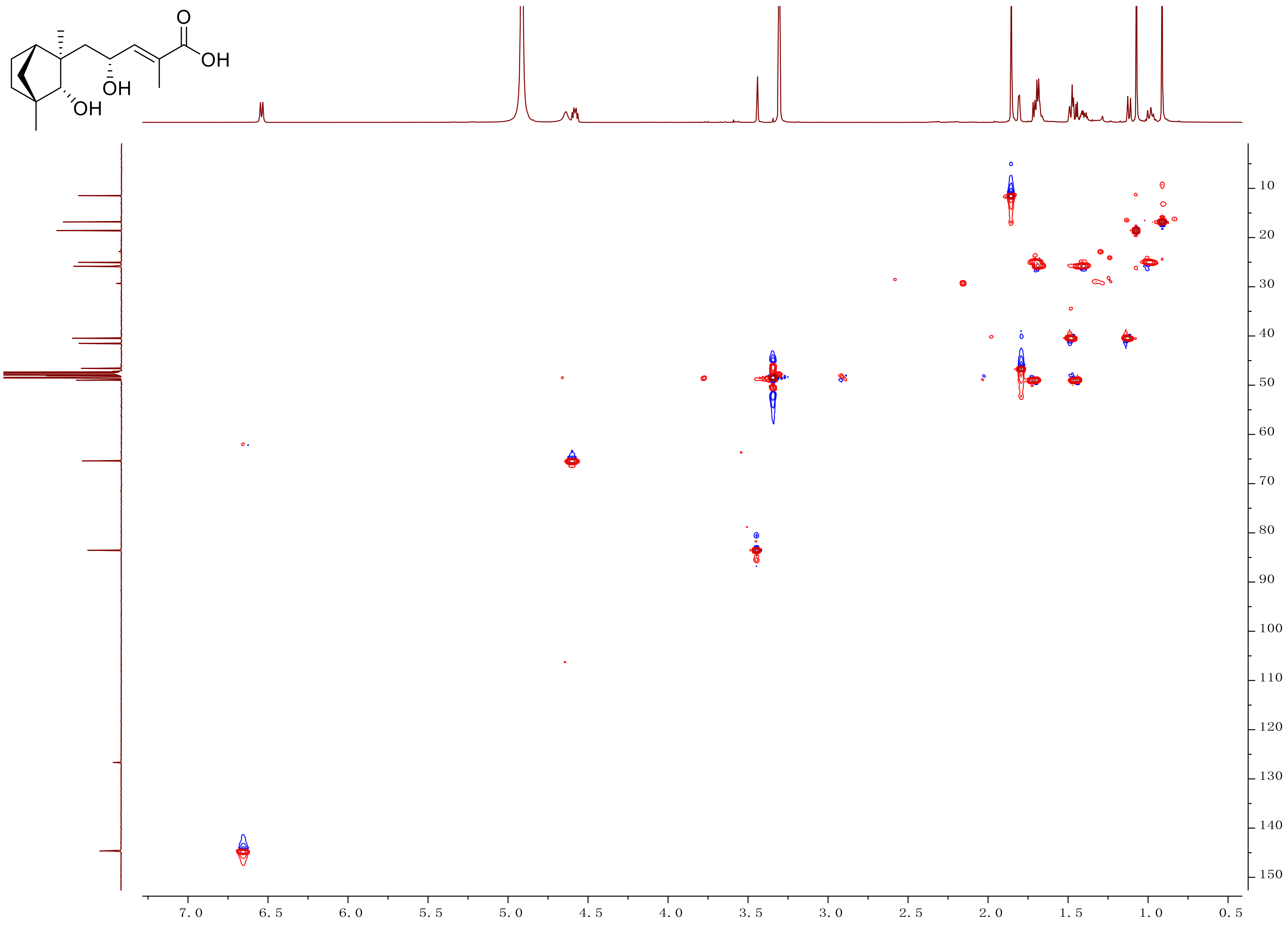

Figure S48. HMBC of compound **4** in CD_3OD

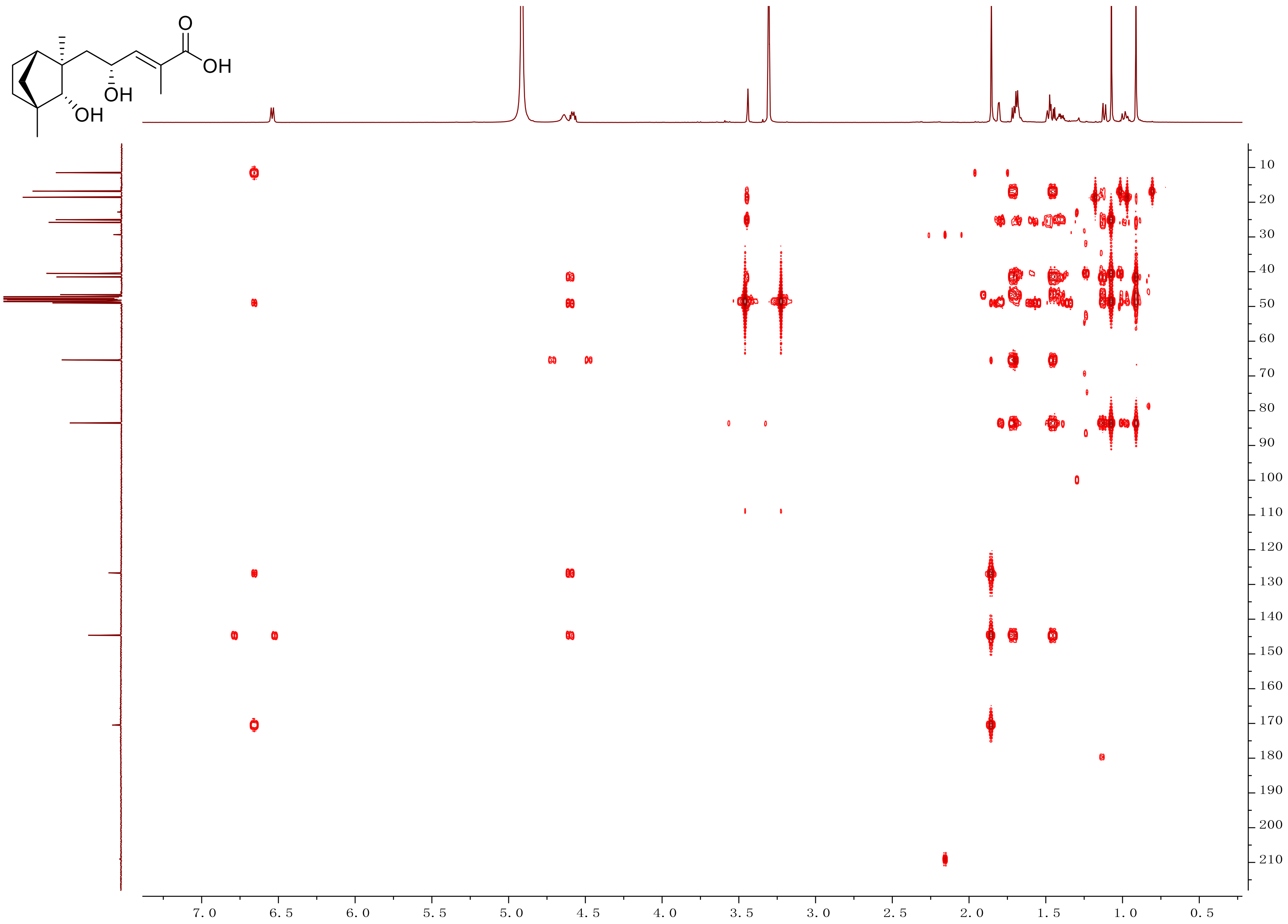

Figure S49. ^{1}H-^{1}H COSY of compound **4** in CD_3OD

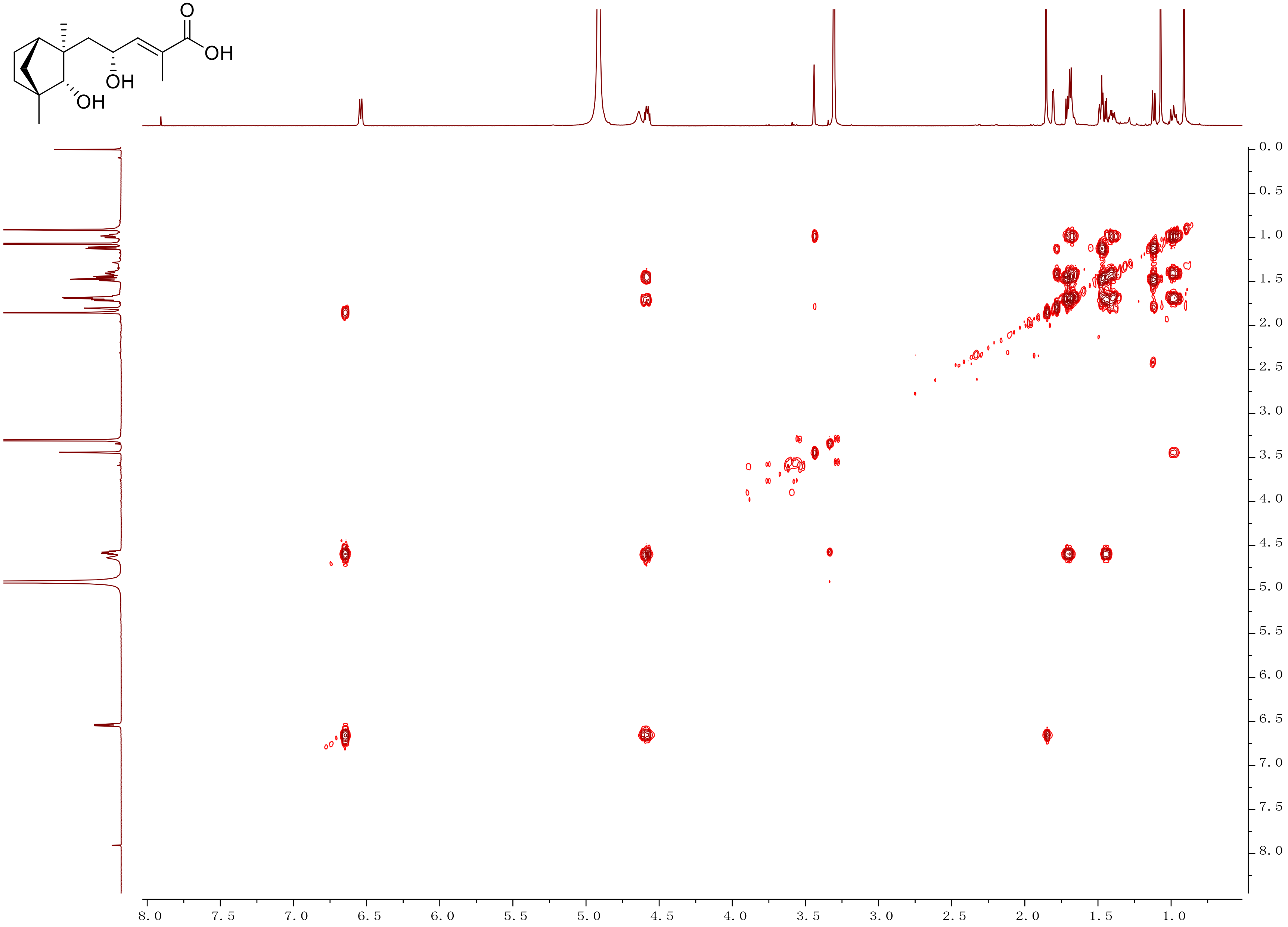

Figure S50. ROESY of compound **4** in CD_3OD

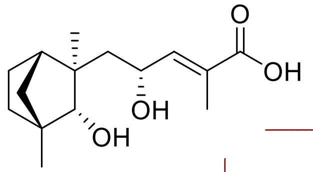

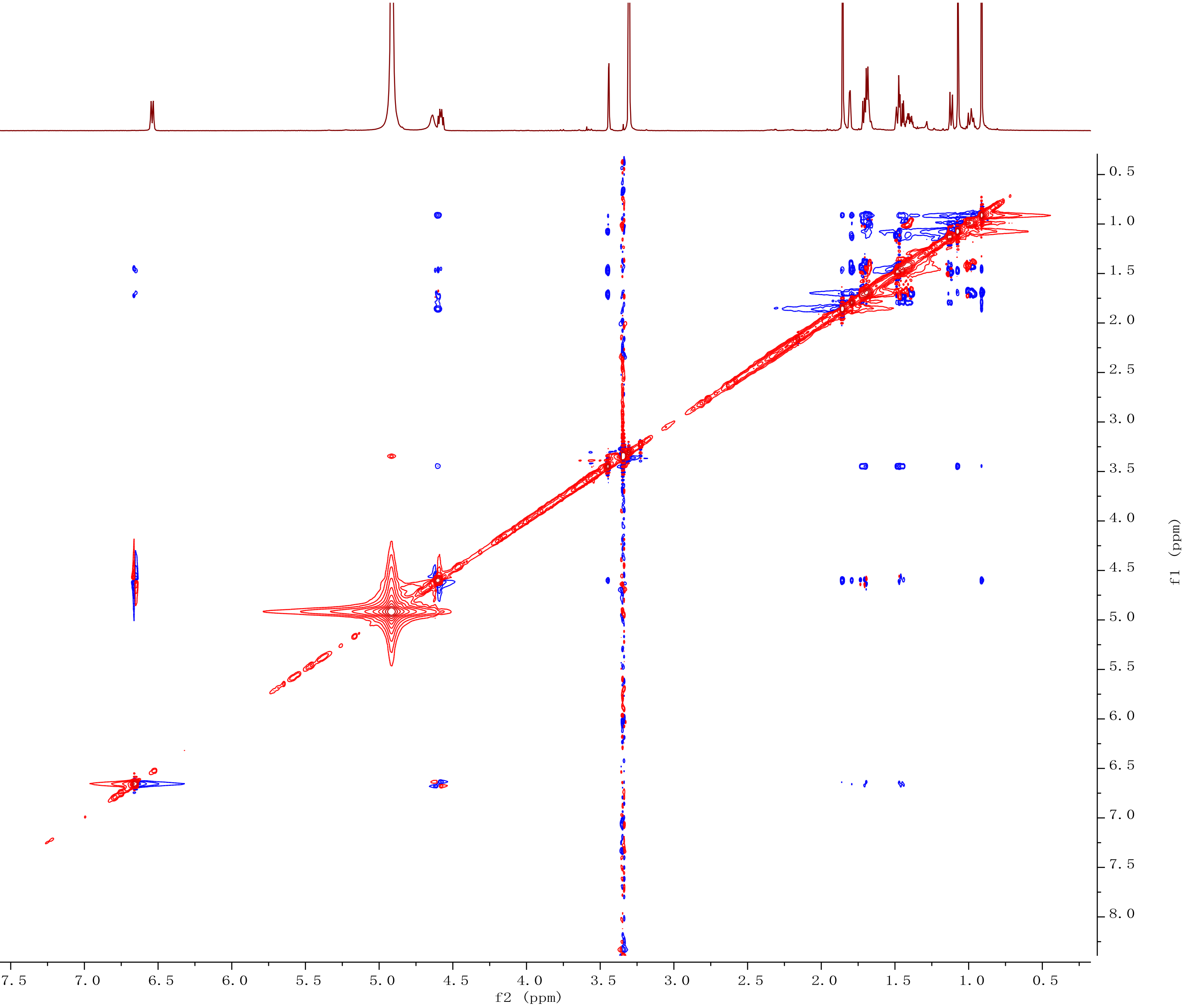

Figure S51. HR-ESIMS of compound **4**

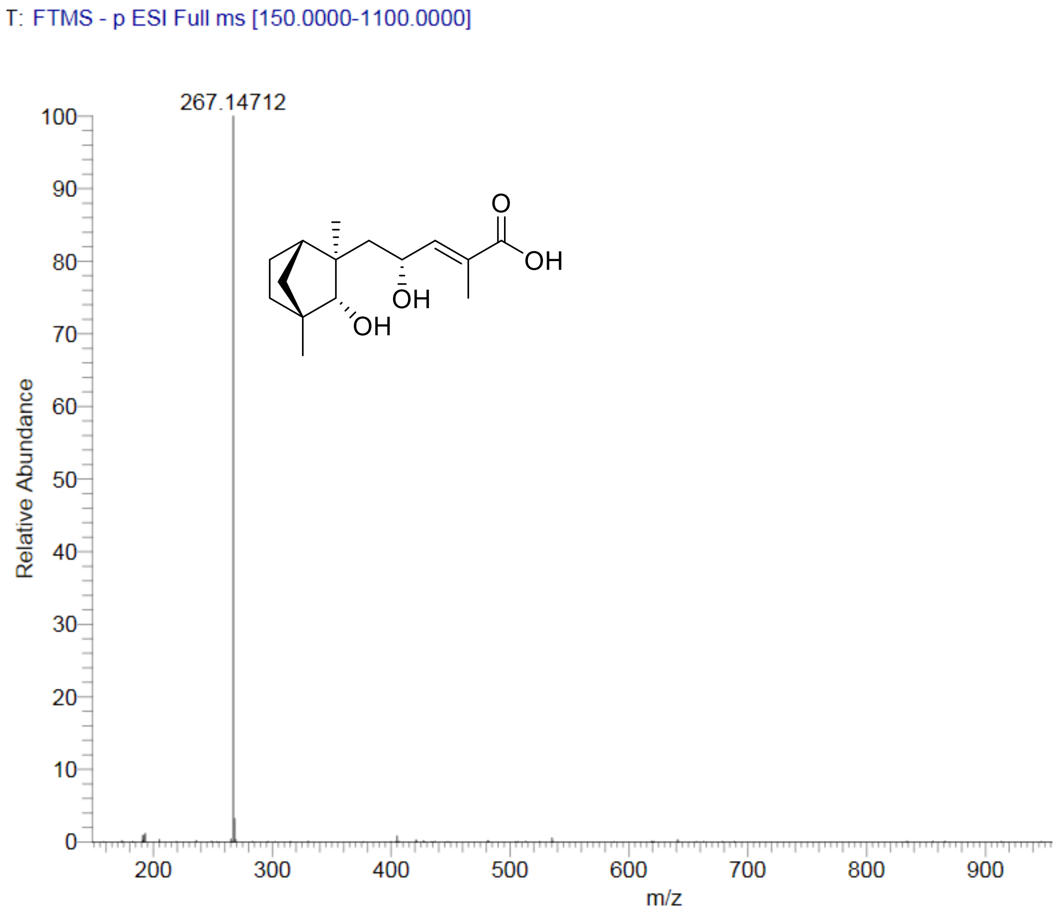

Section S12. NMR and MS spectra for 5

Figure S52. ^{1}H NMR of compound **5** in CD_3OD

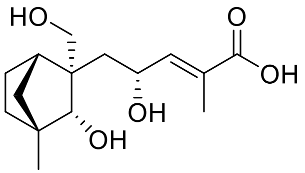

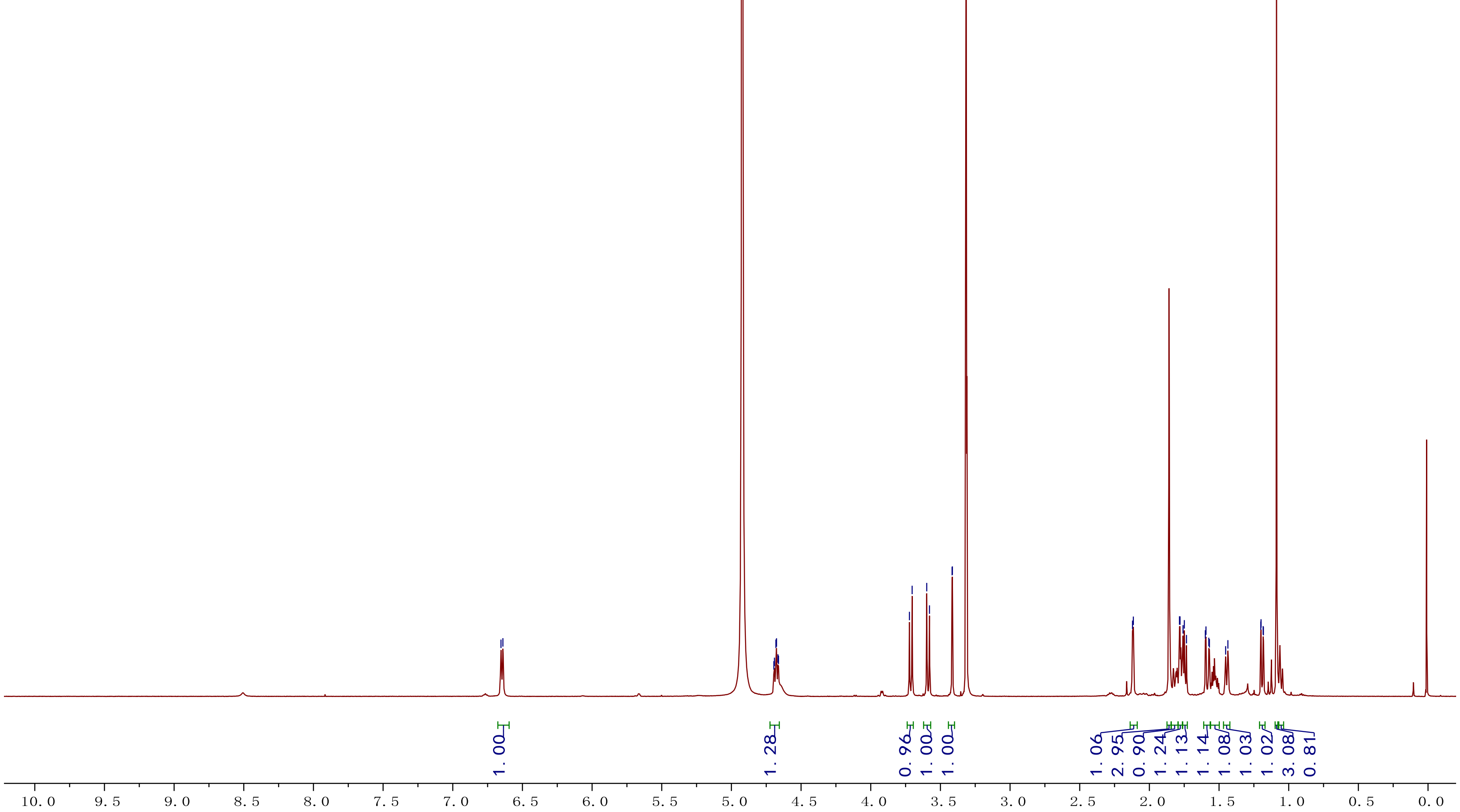

Figure S53. ^{13}C NMR and DEPT of compound **5** in CD_3OD

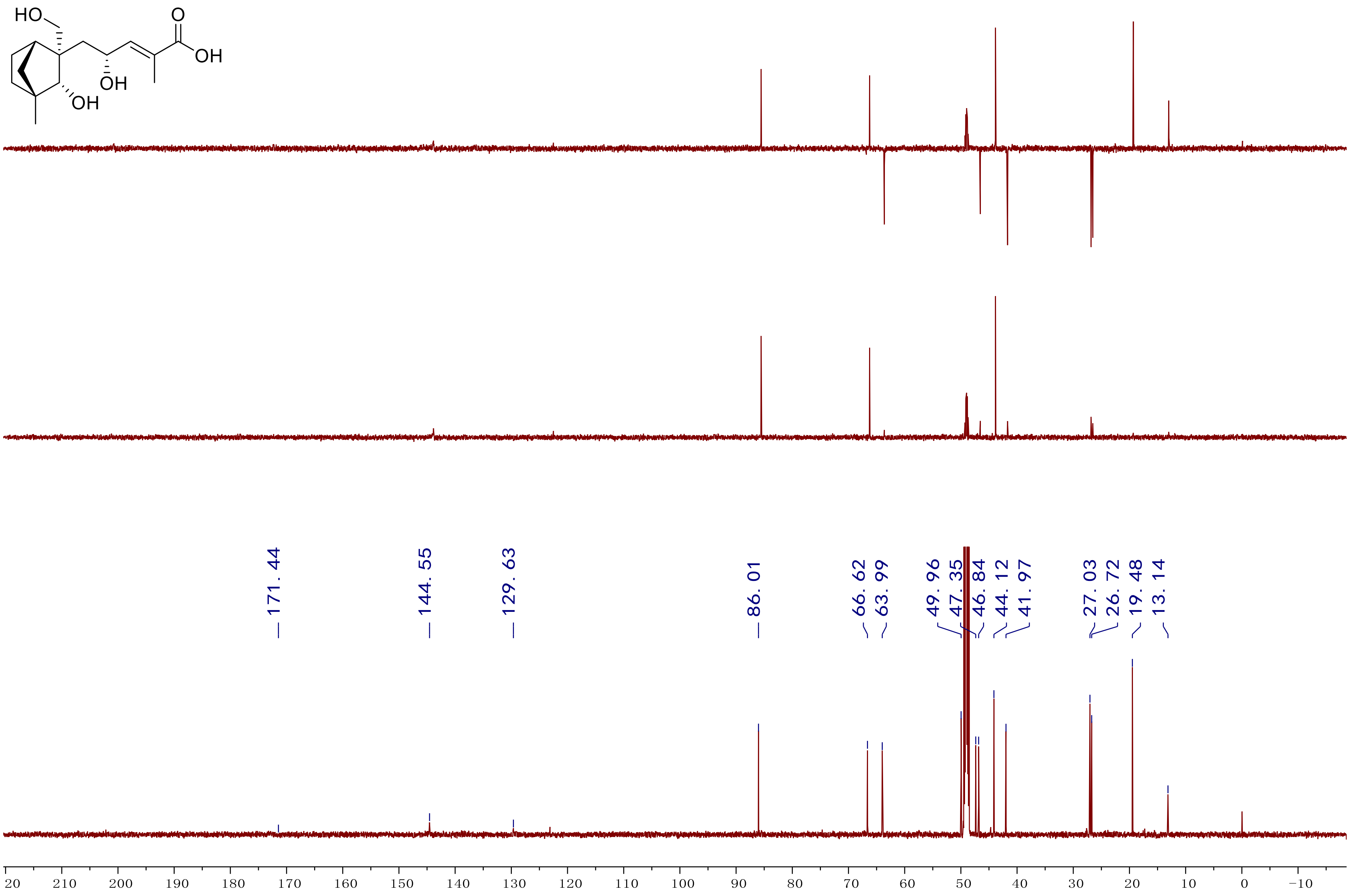

Figure S54. HSQC of compound **5** in CD_3OD

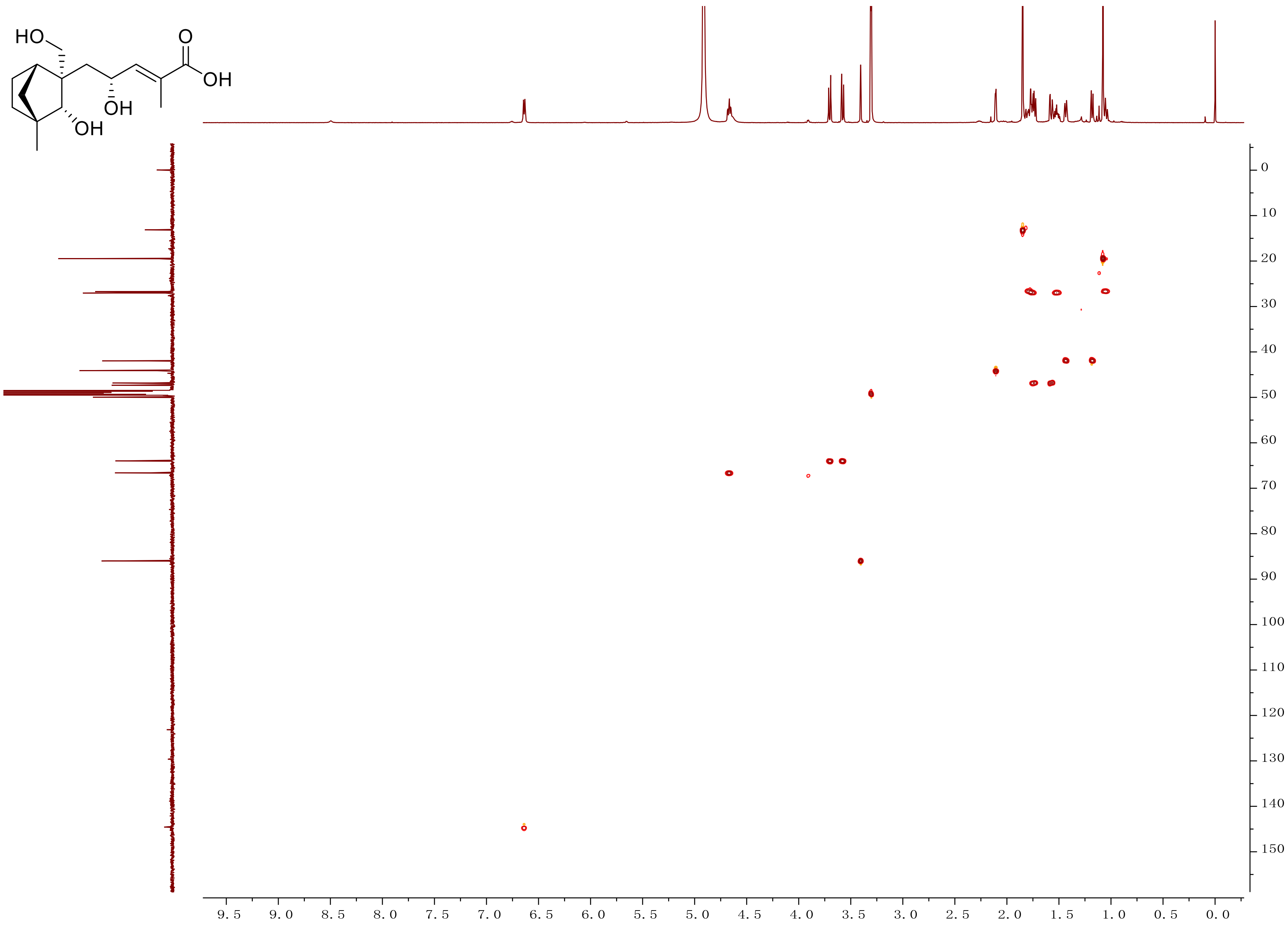

Figure S55. HMBC of compound **5** in CD_3OD

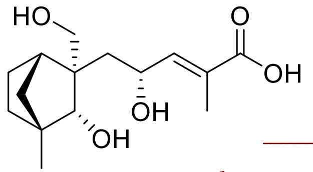

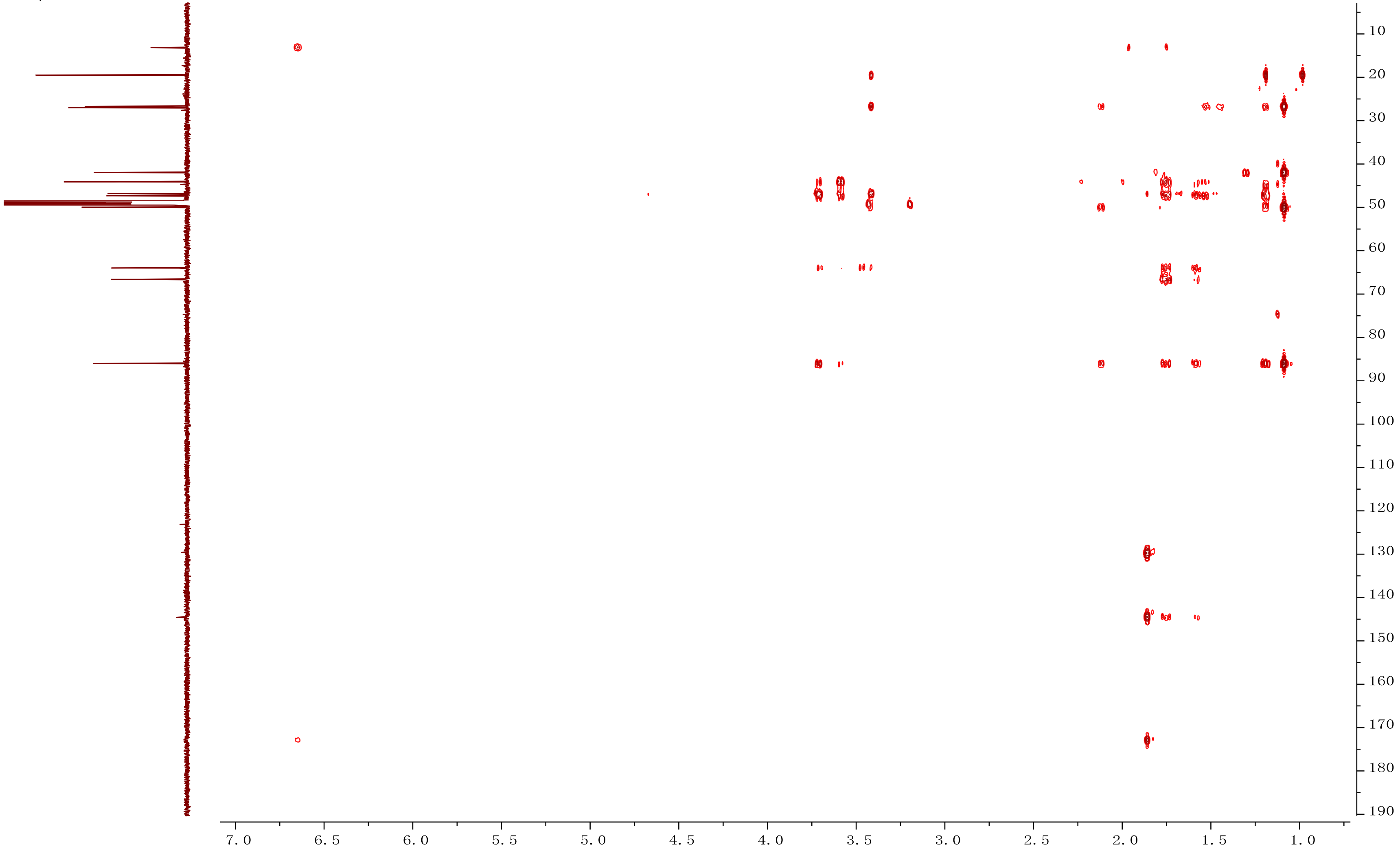

Figure S56. ¹H-¹H COSY of compound **5** in CD_3OD

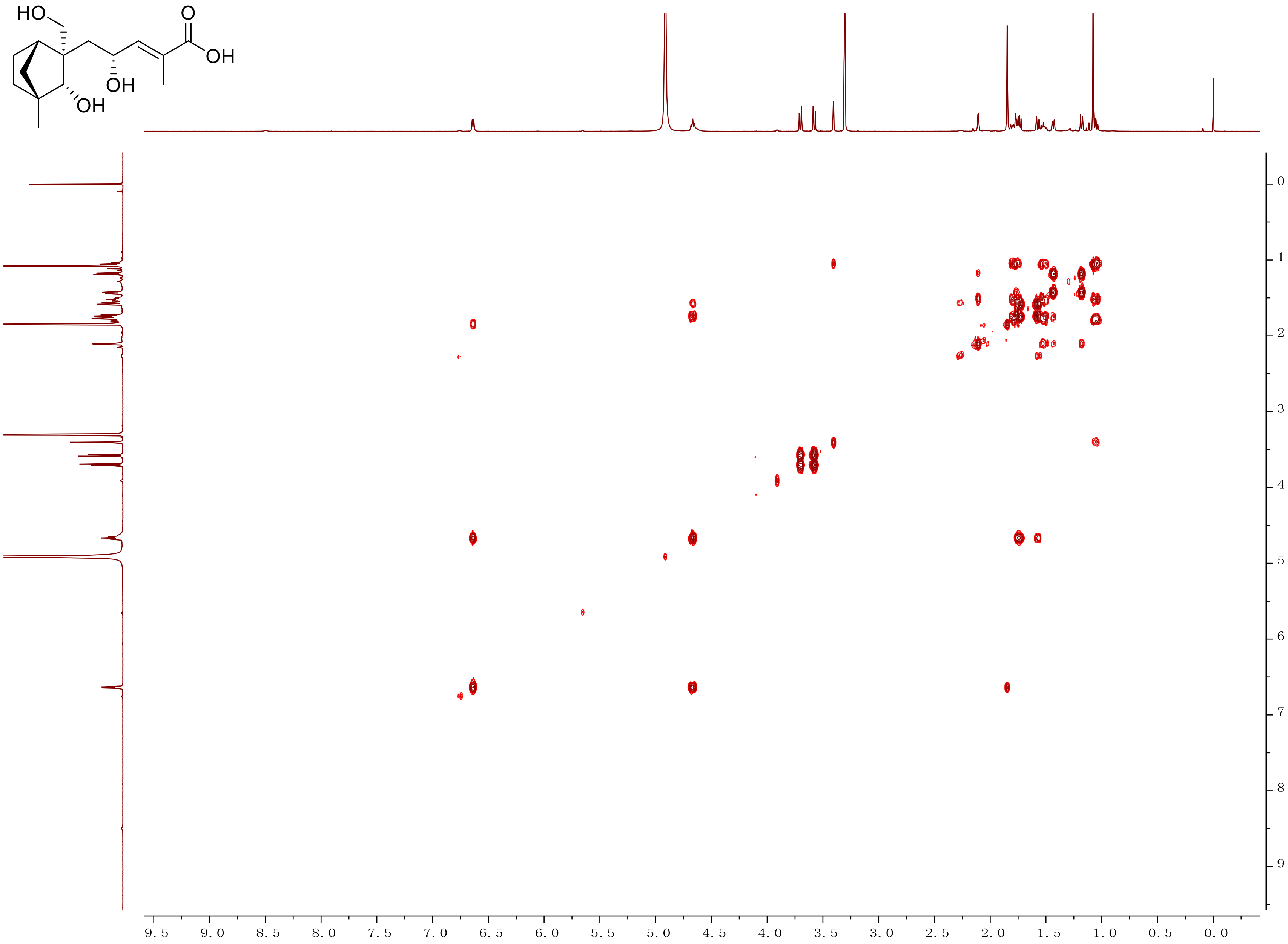

Figure S57. ROESY of compound **5** in CD_3OD

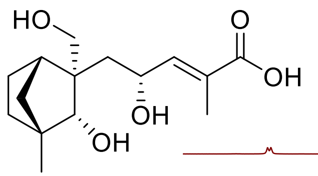

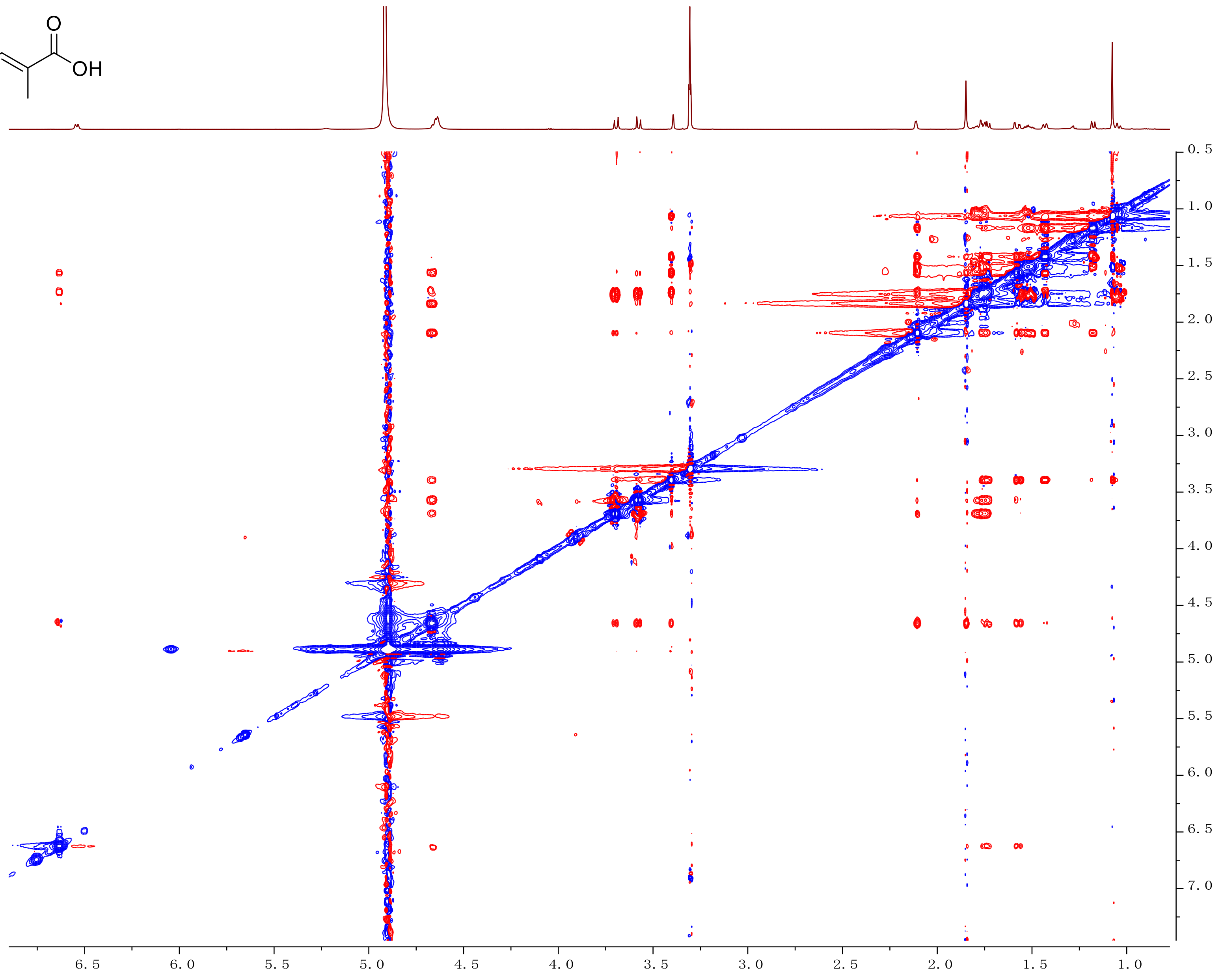

Figure S58. HR-ESIMS of compound **5**

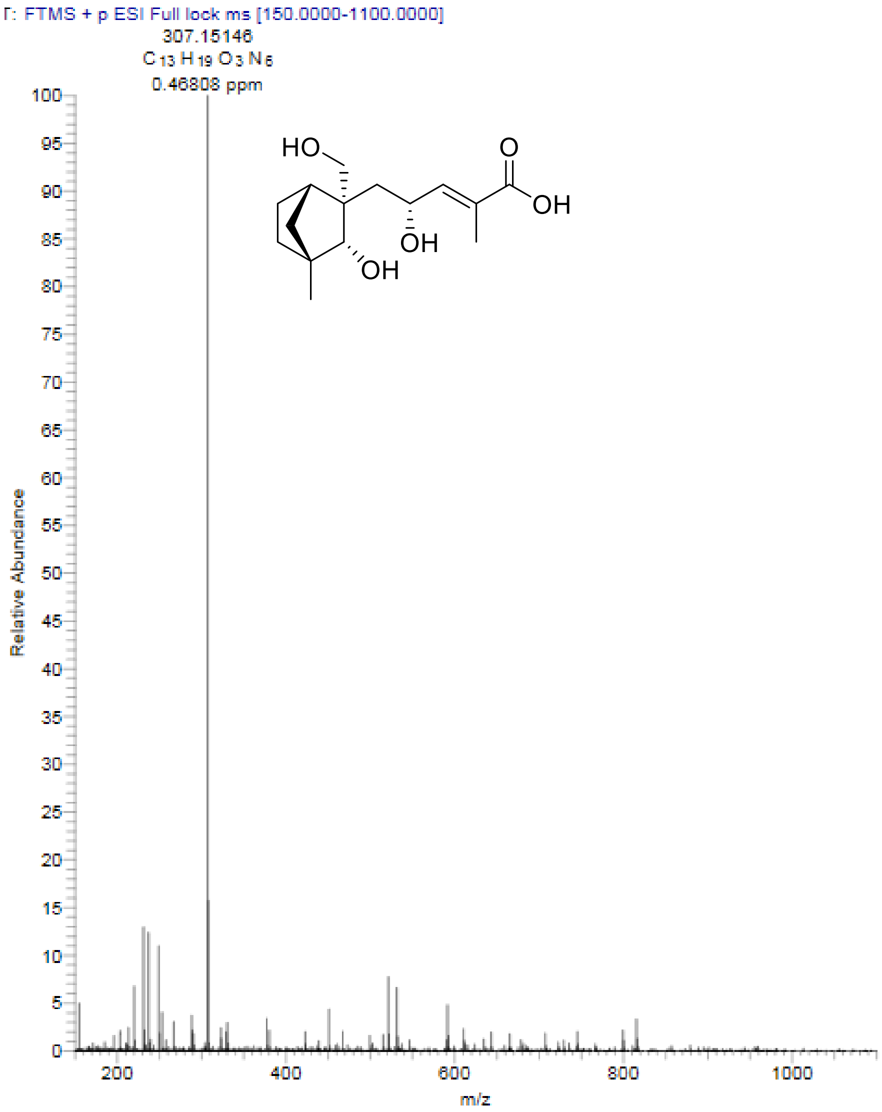

Section S13. NMR and MS spectra for 6

Figure S59. ^{1}H NMR of compound **6** in CD_3OD

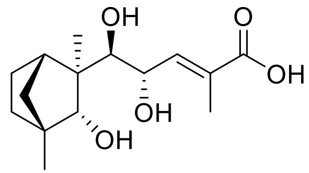

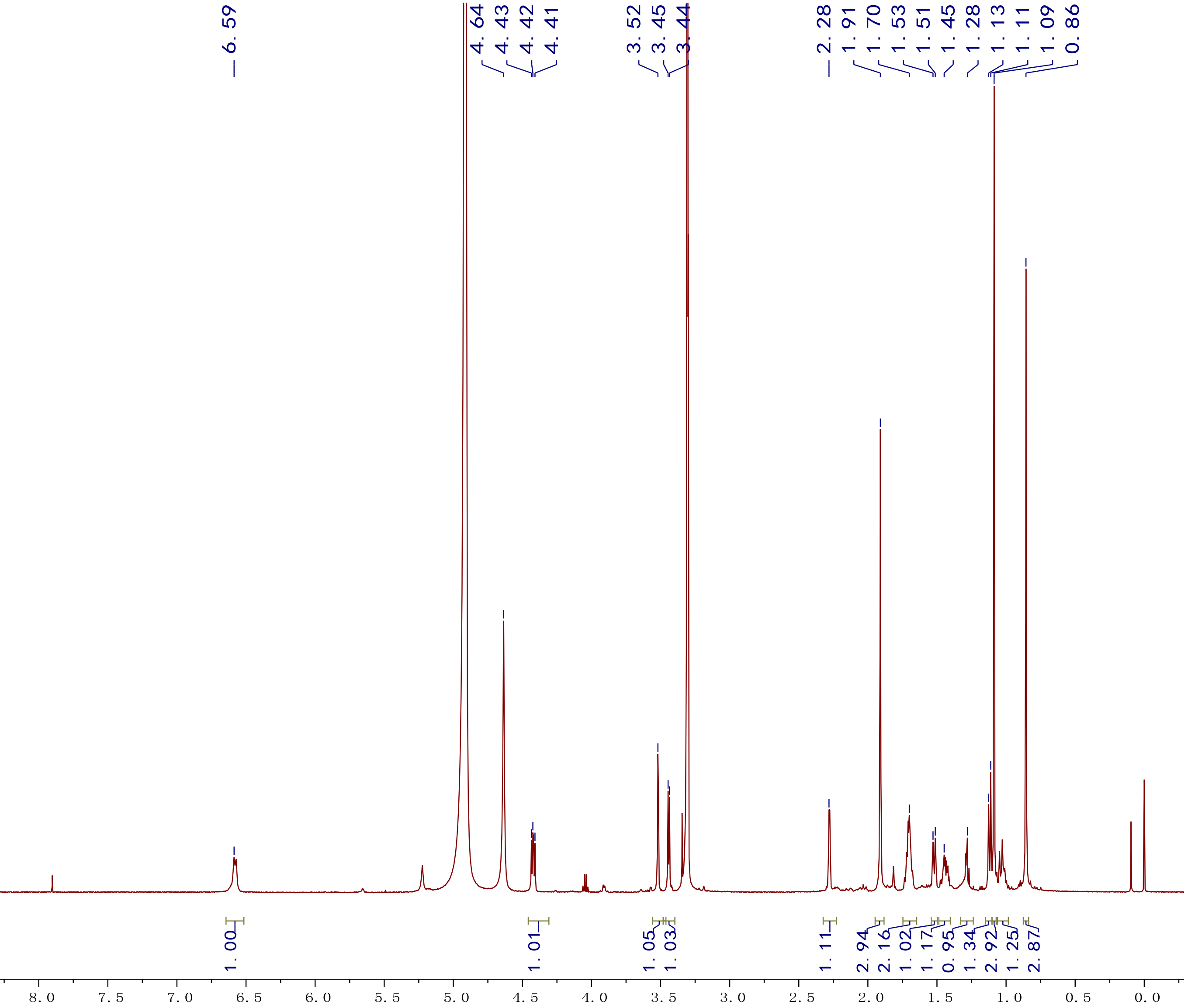

Figure S60. ^{13}C NMR and DEPT of compound **6** in CD_3OD

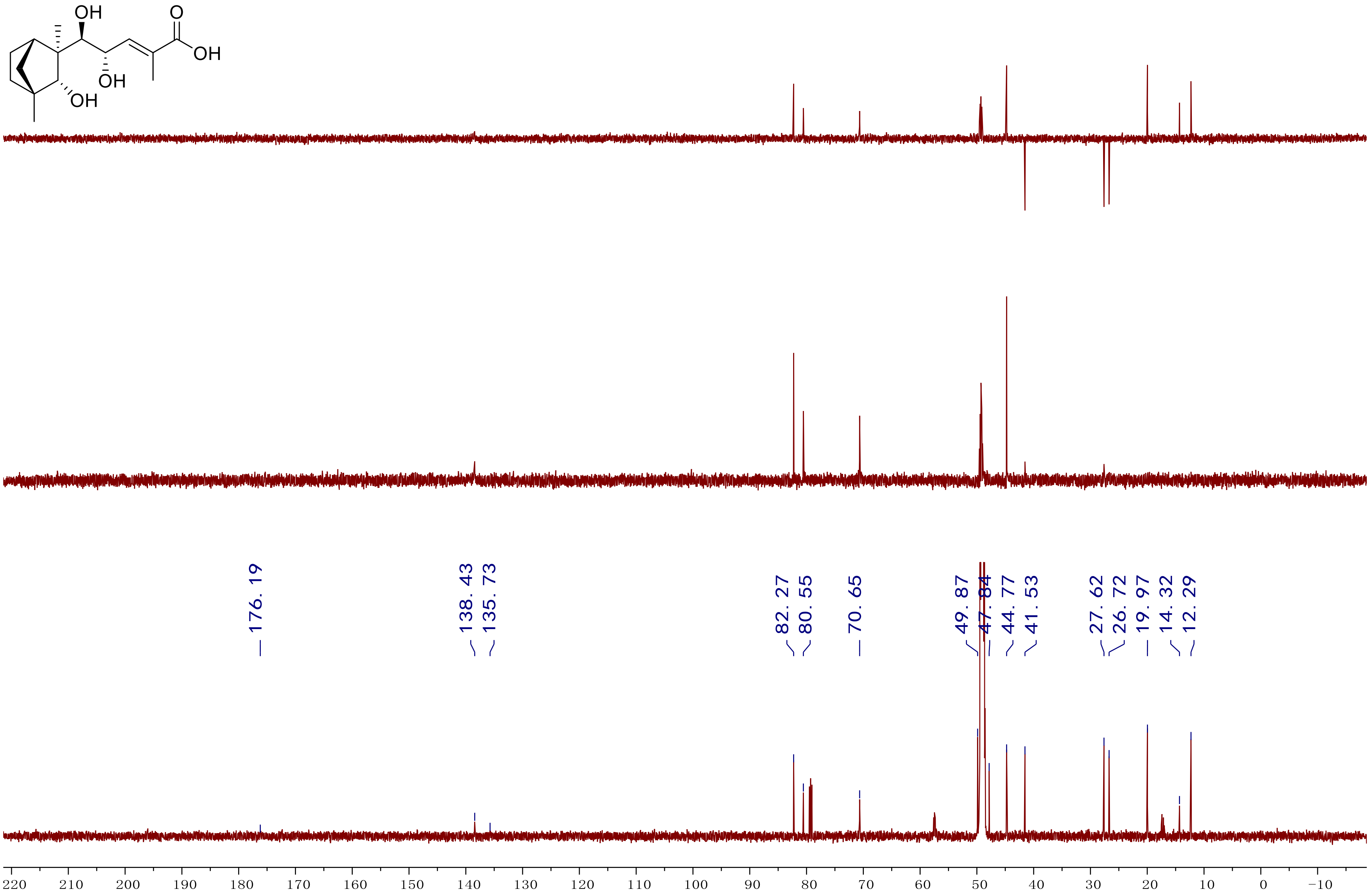

Figure S61. HSQC of compound **6** in CD_3OD

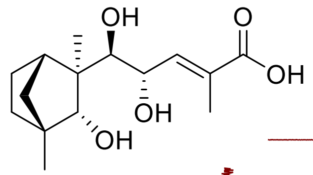

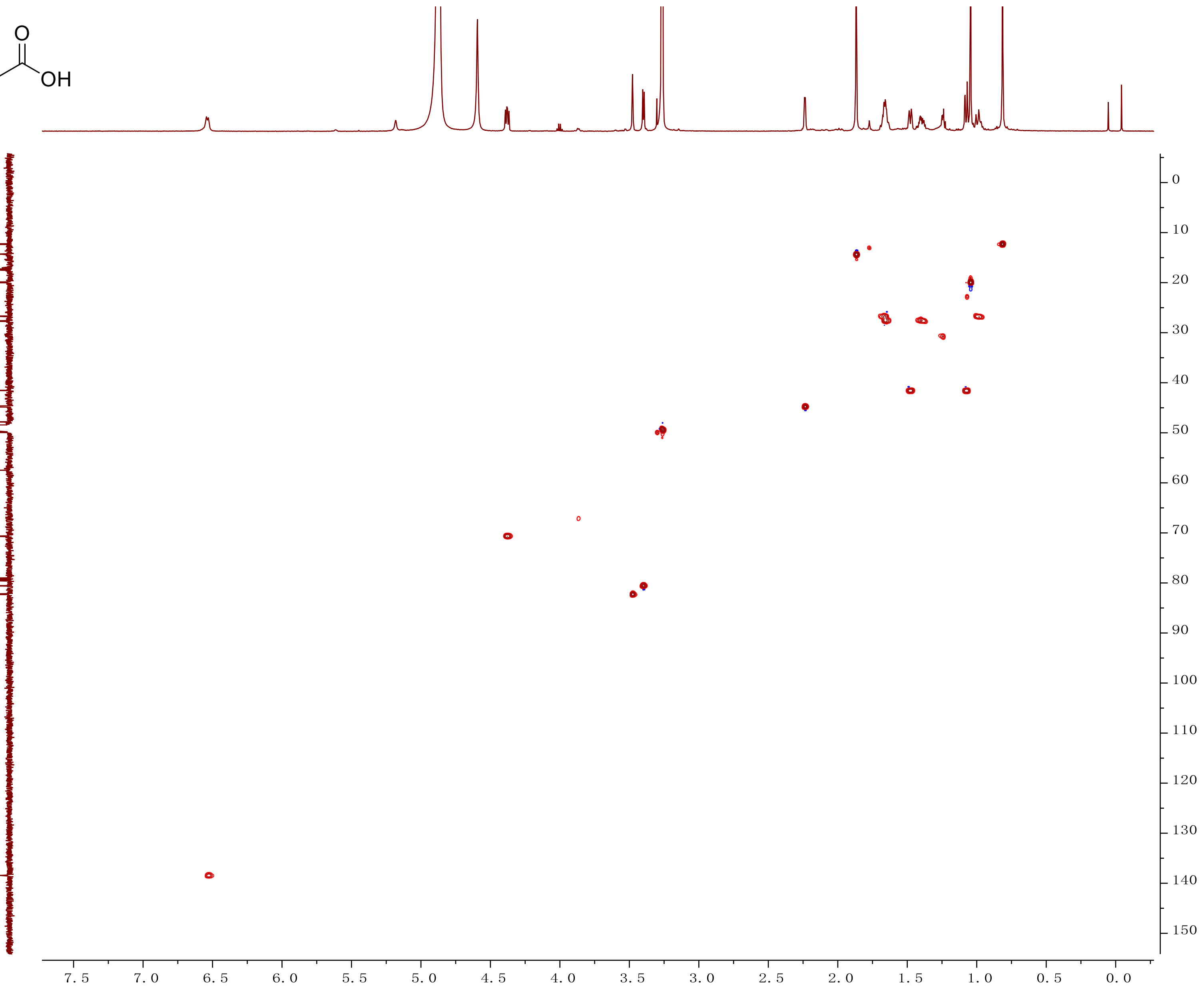

Figure S62. HMBC of compound **6** in CD_3OD

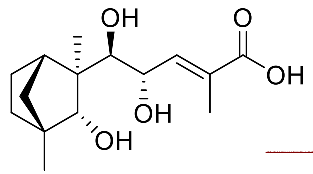

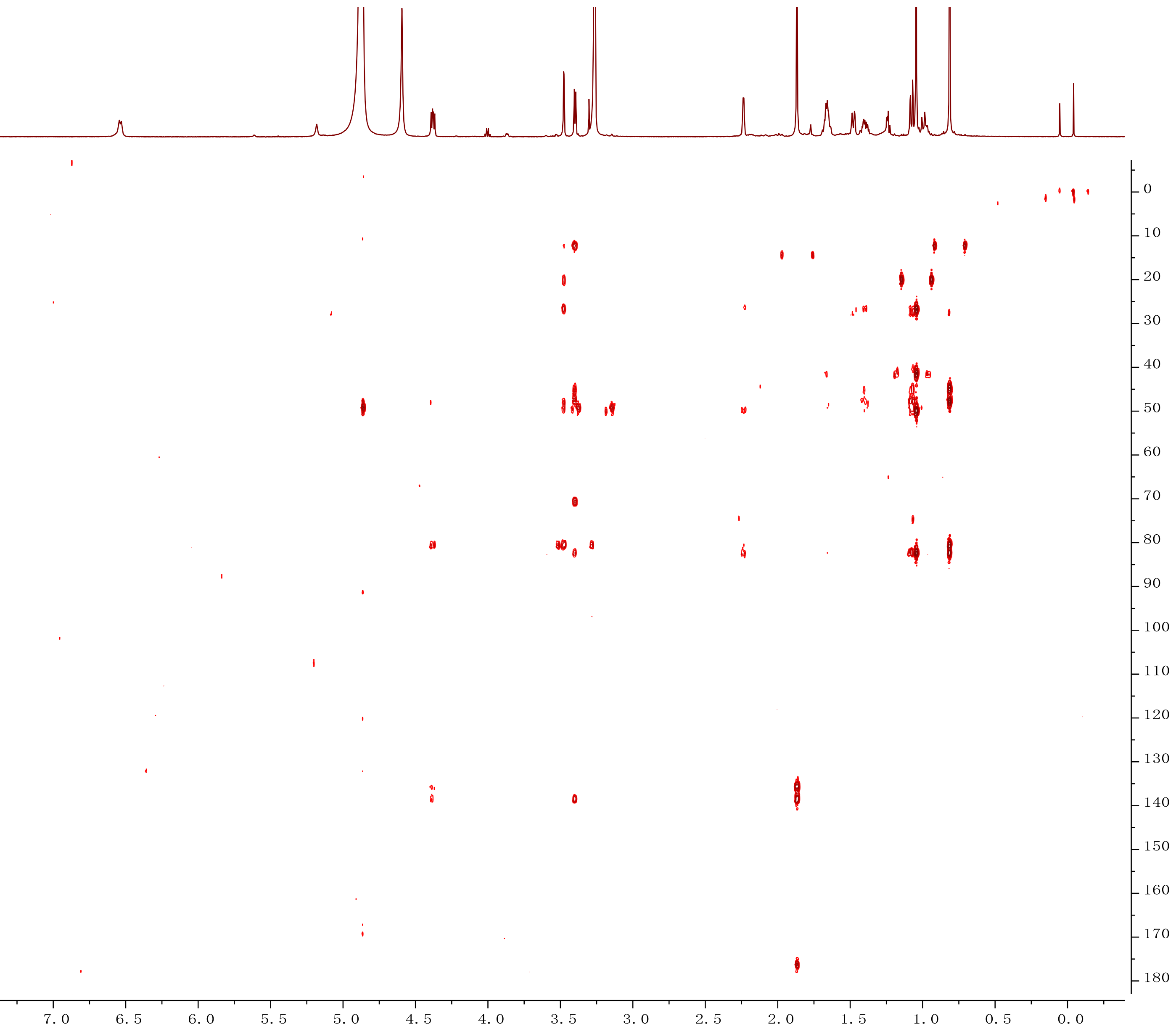

Figure S63. ^{1}H-^{1}H COSY of compound **6** in CD_3OD

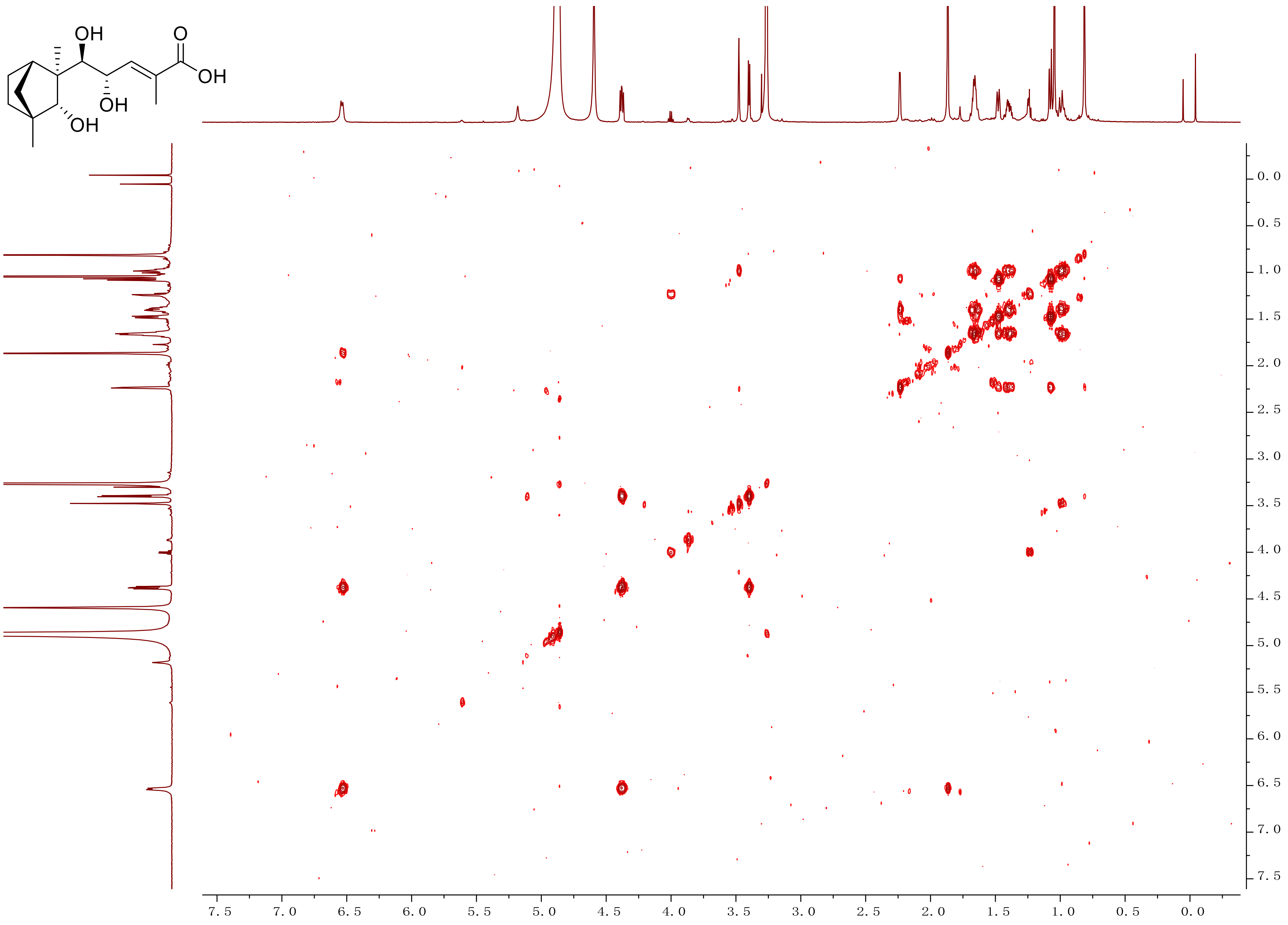

Figure S64. ROESY of compound **6** in CD_3OD

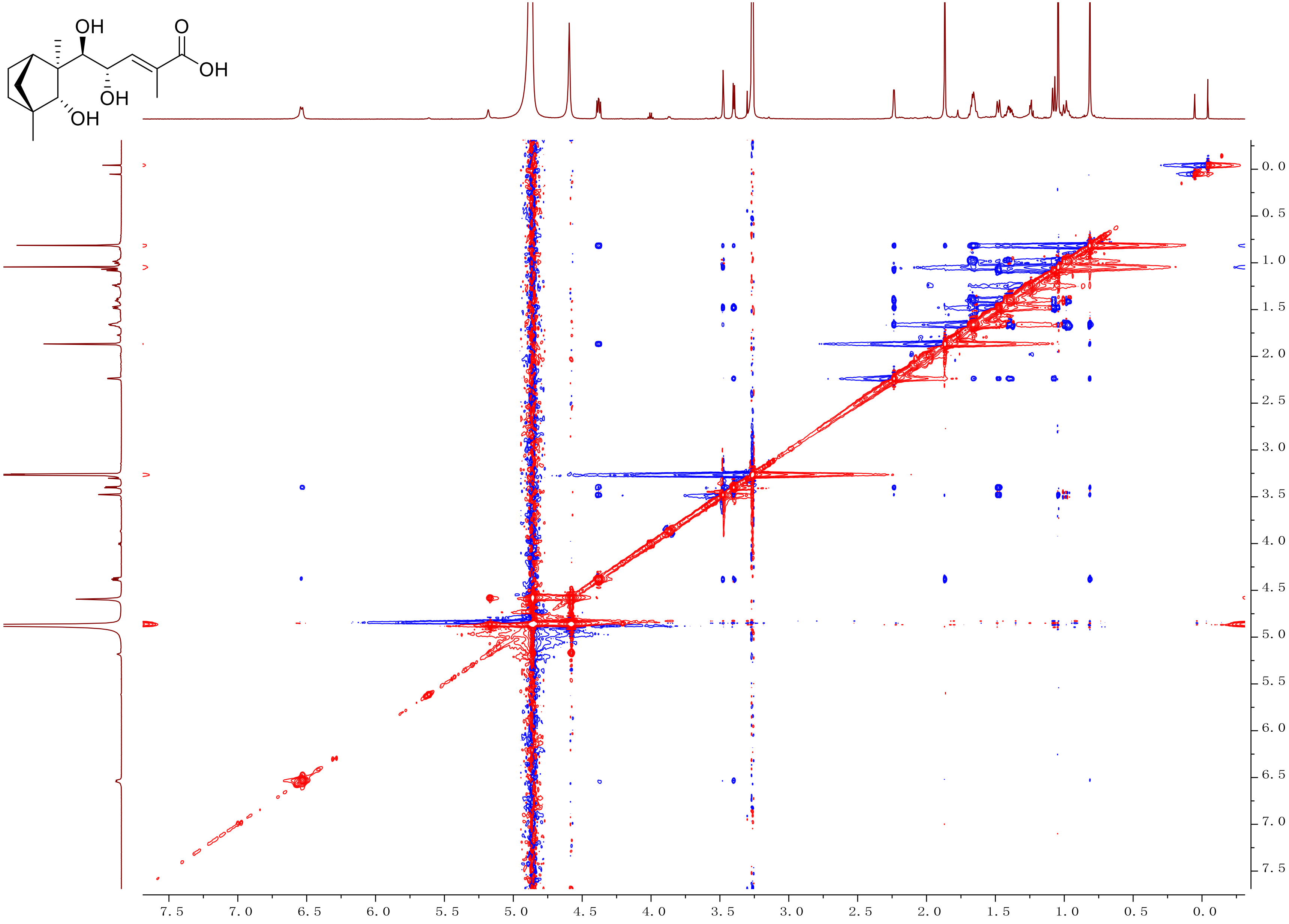

Figure S65. HR-ESIMS of compound **6**

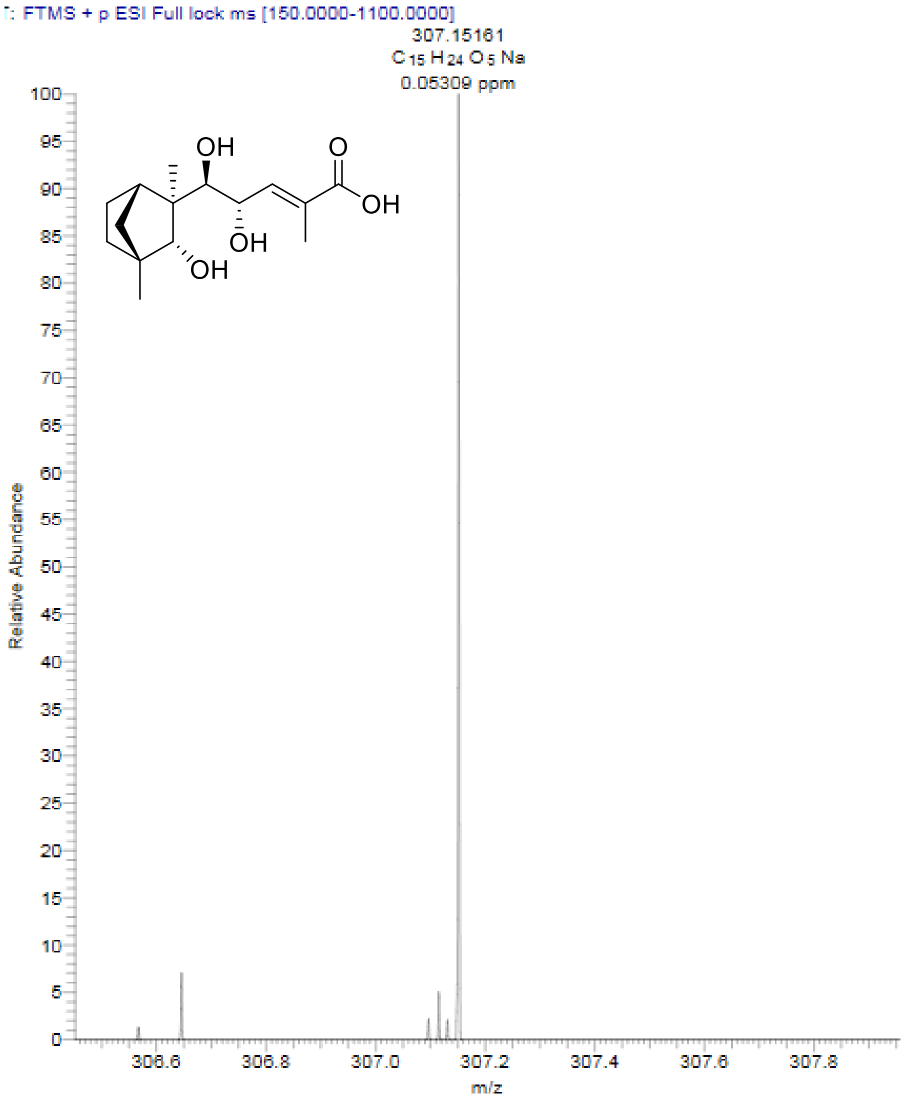

Section S14. NMR and MS spectra for 7*S*,9*S*,14*S*-7

Figure S66. ^{1}H NMR of compound 7*S*,9*S*,14*S*-**7** in CD_3OD

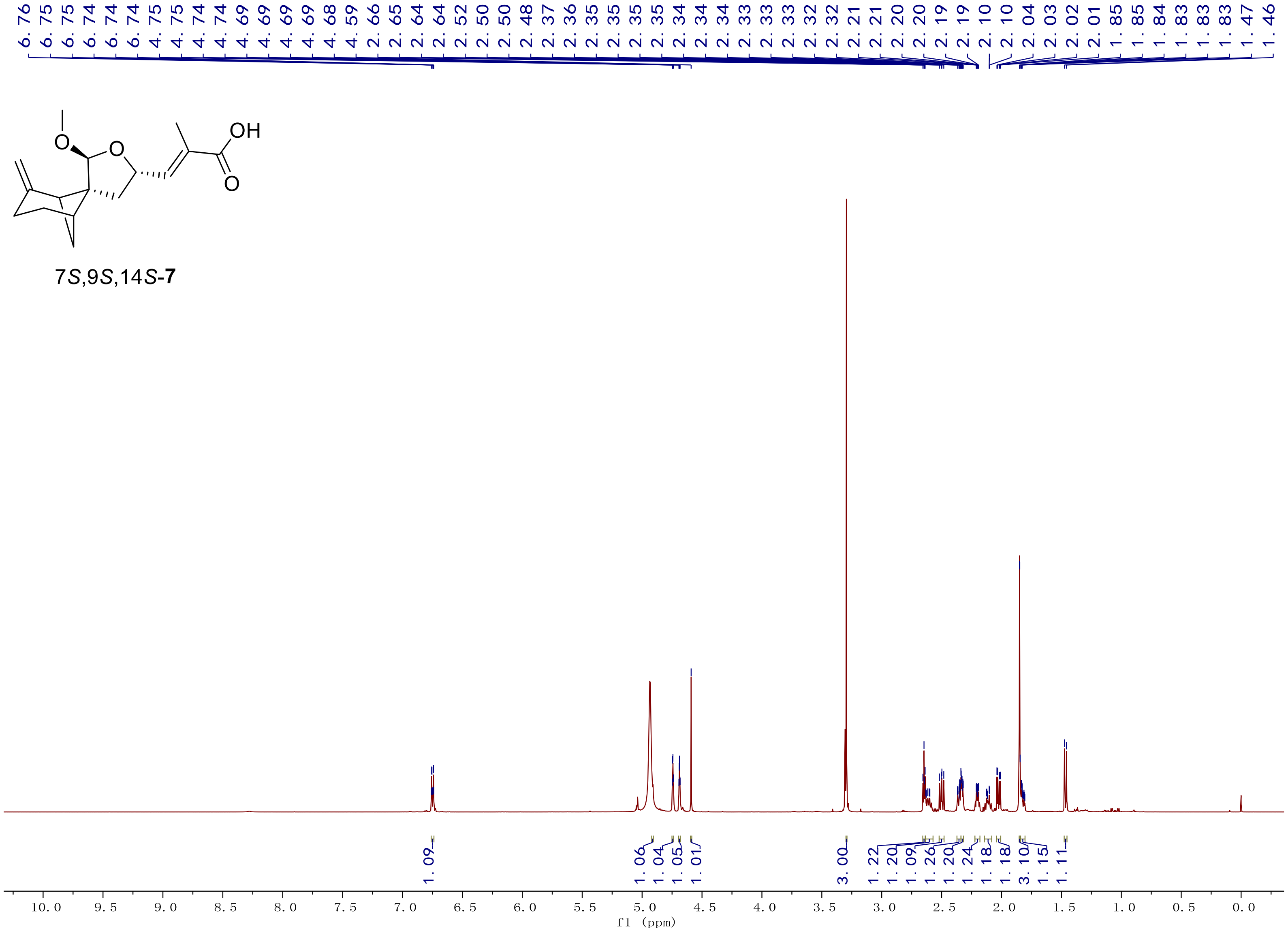

Figure S67. ^{13}C NMR and DEPT of compound 7*S*,9*S*,14*S*-**7** in CD_3OD

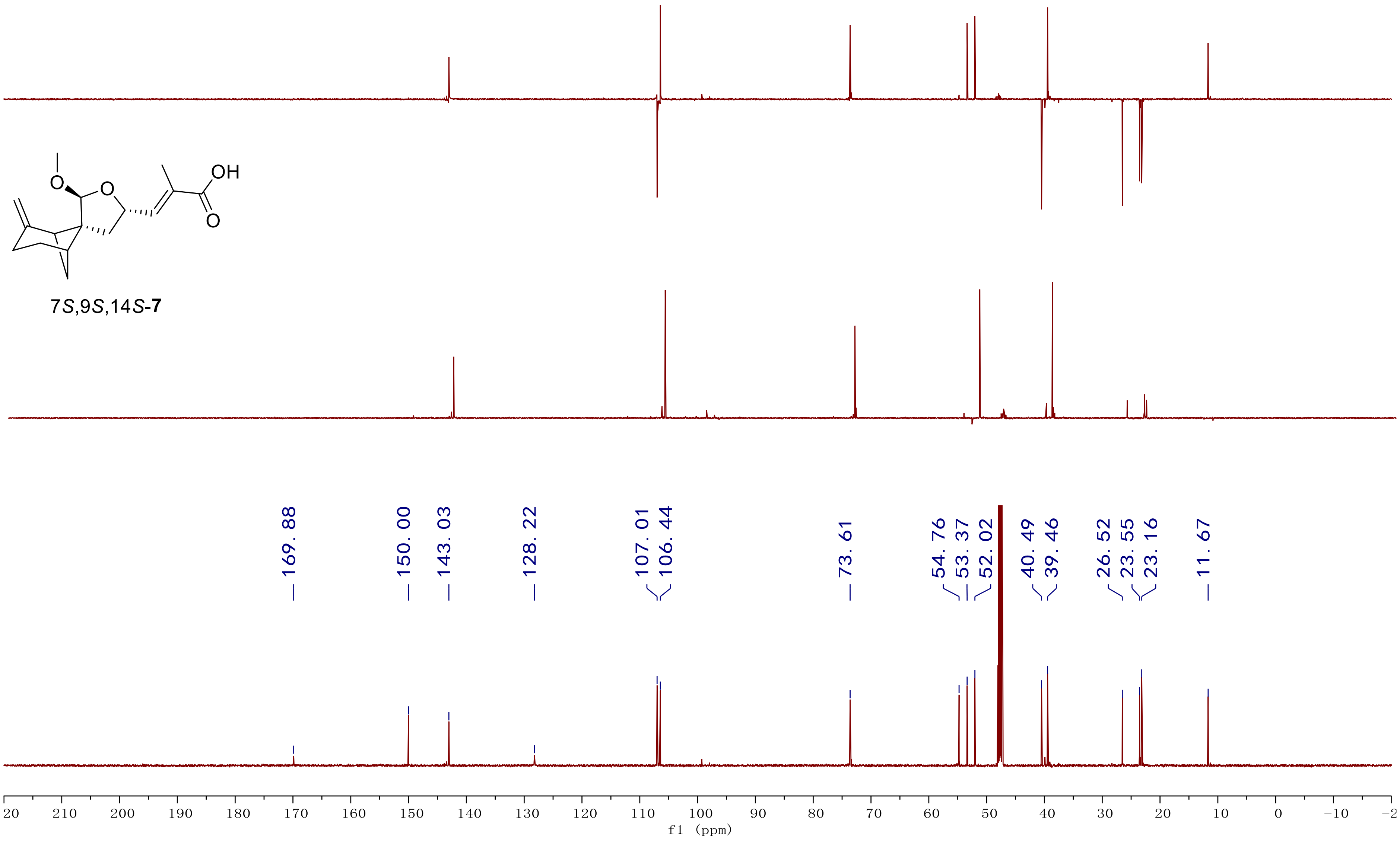

Figure S68. HSQC of compound 7*S*,9*S*,14*S*-**7** in CD_3OD

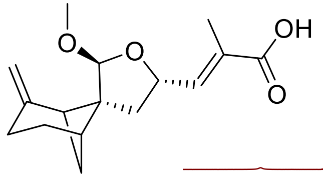

7*S*,9*S*,14*S*-**7**

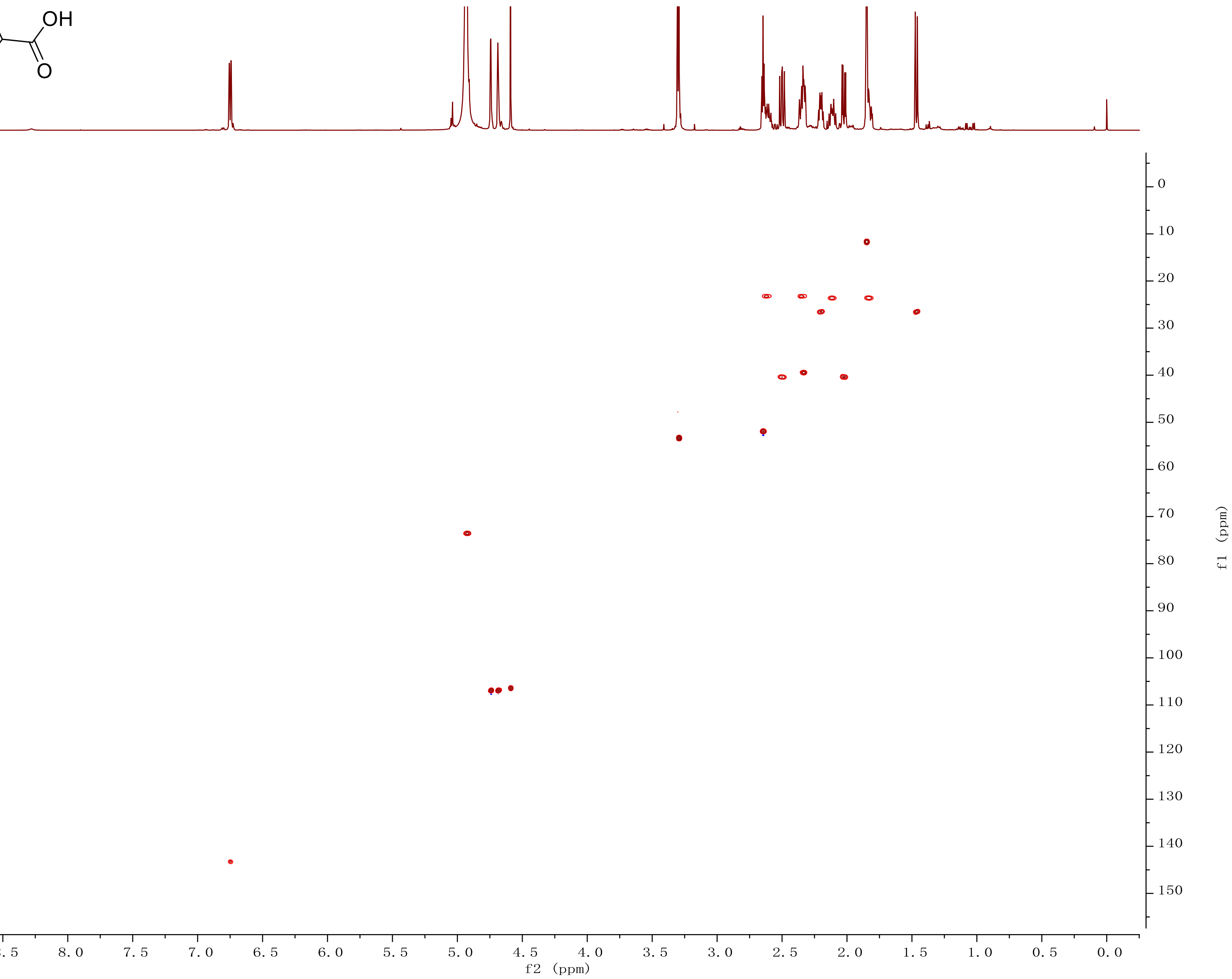

Figure S69. HMBC of compound 7*S*,9*S*,14*S*-**7** in CD_3OD

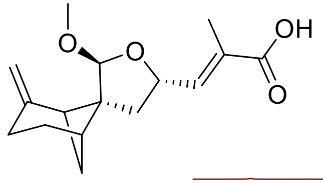

7*S*,9*S*,14*S*-**7**

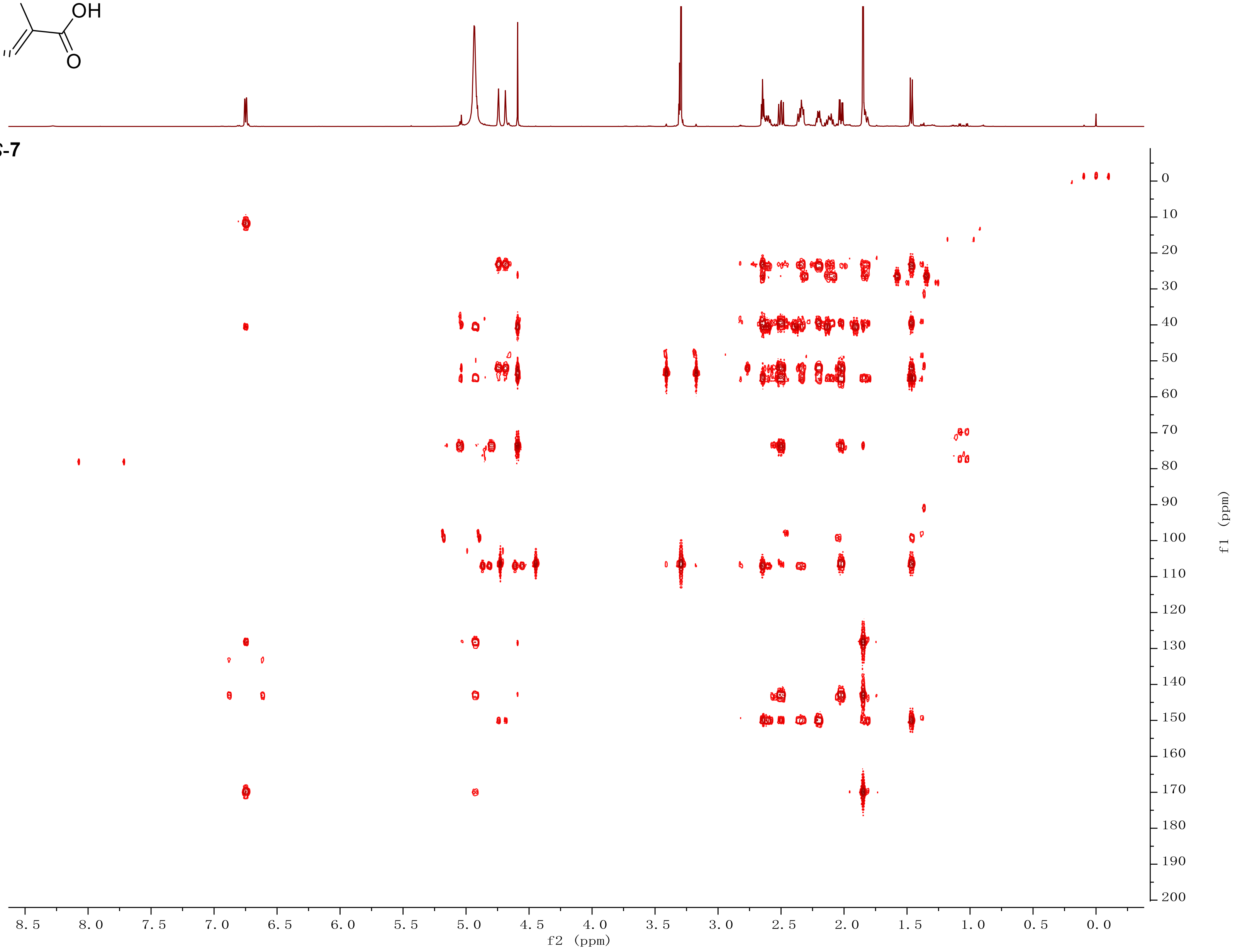

Figure S70. ^{1}H-^{1}H COSY of compound 7*S*,9*S*,14*S*-**7** in CD_3OD

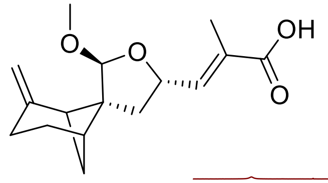

7*S*,9*S*,14*S*-**7**

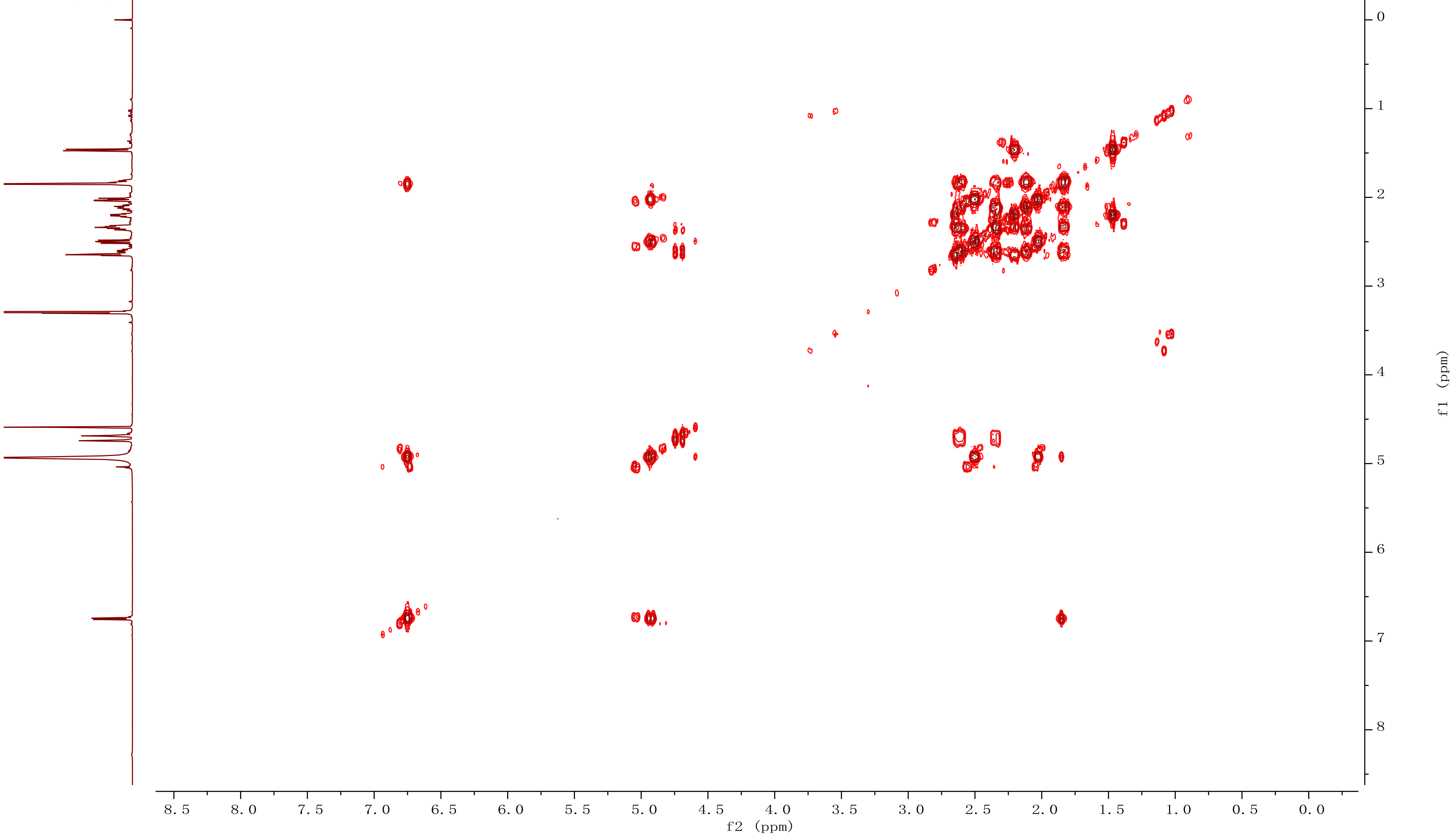

Figure S71. ROESY of compound 7*S*,9*S*,14*S*-**7** in CD_3OD

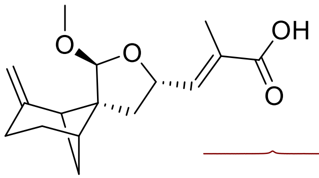

7*S*,9*S*,14*S*-**7**

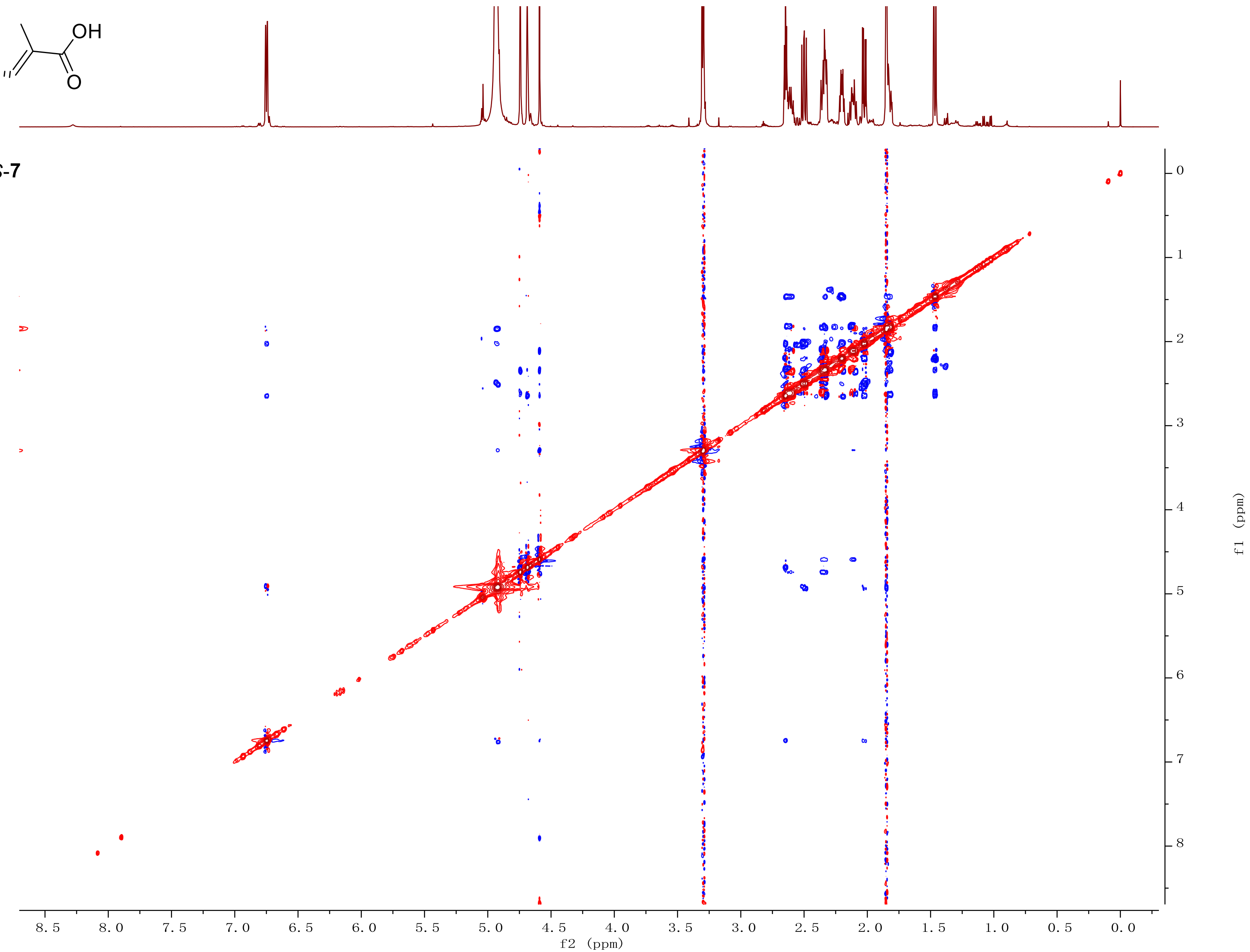

Figure S72. HR-ESIMS of compound 7*S*,9*S*,14*S*-**7**

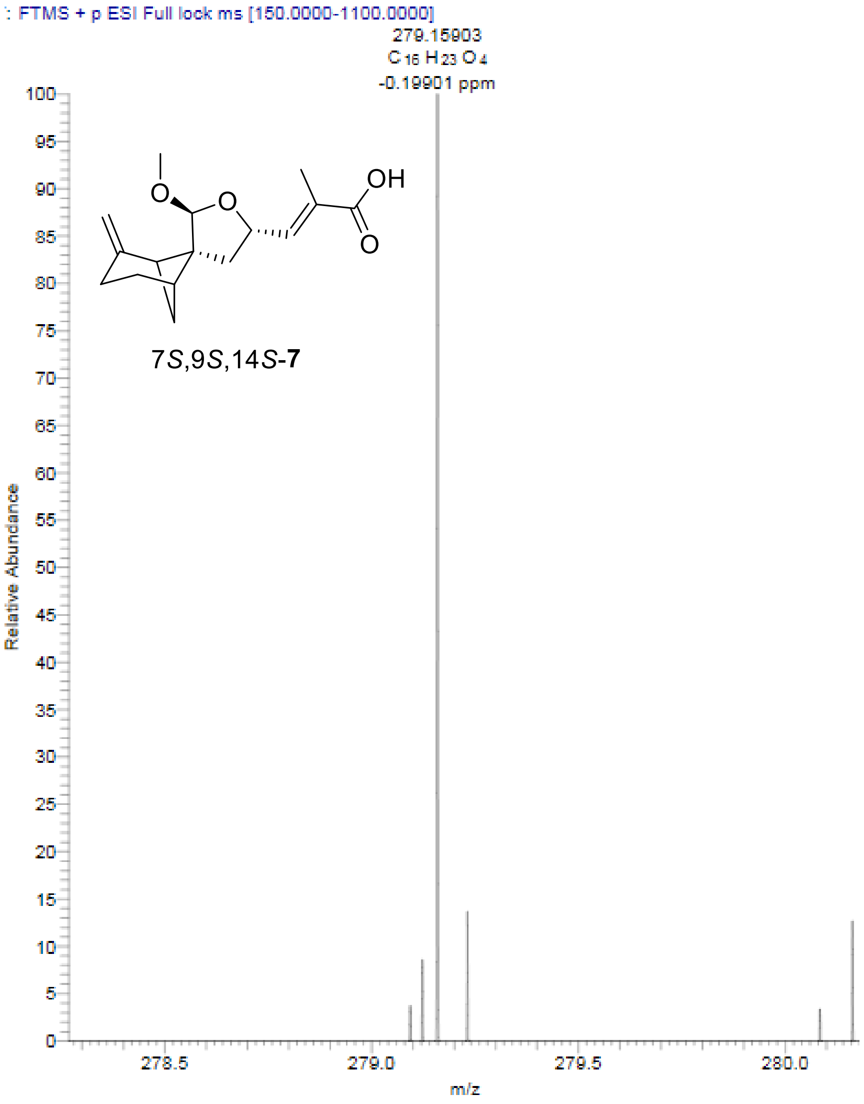

Section S15. NMR and MS spectra for 7*S*,9*S*,14*R*-8

Figure S73. ^{1}H NMR of compound 7*S*,9*S*,14*R*-**8** in CD_3OD

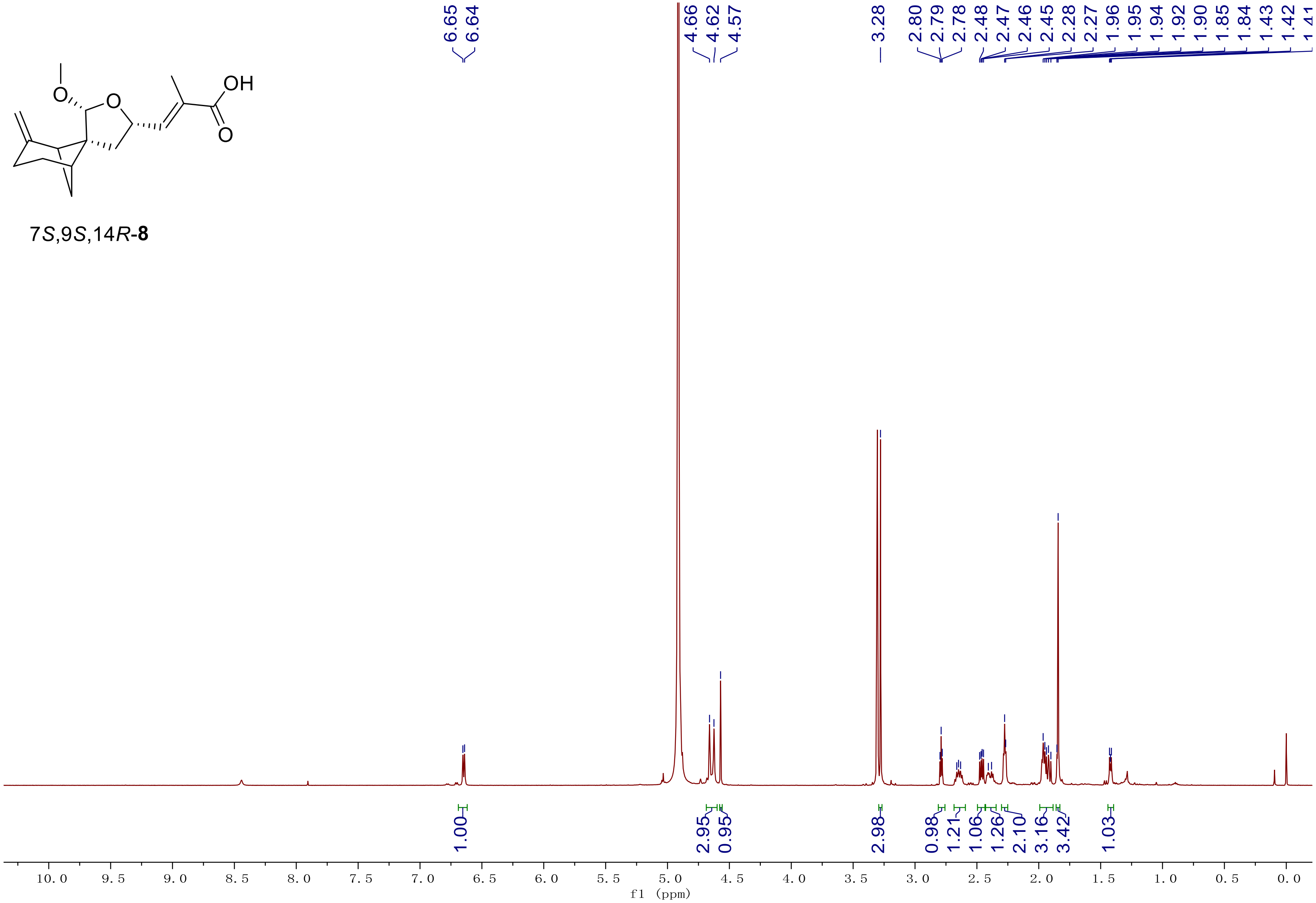

Figure S74. ^{13}C NMR and DEPT of compound 7*S*,9*S*,14*R*-**8** in CD_3OD

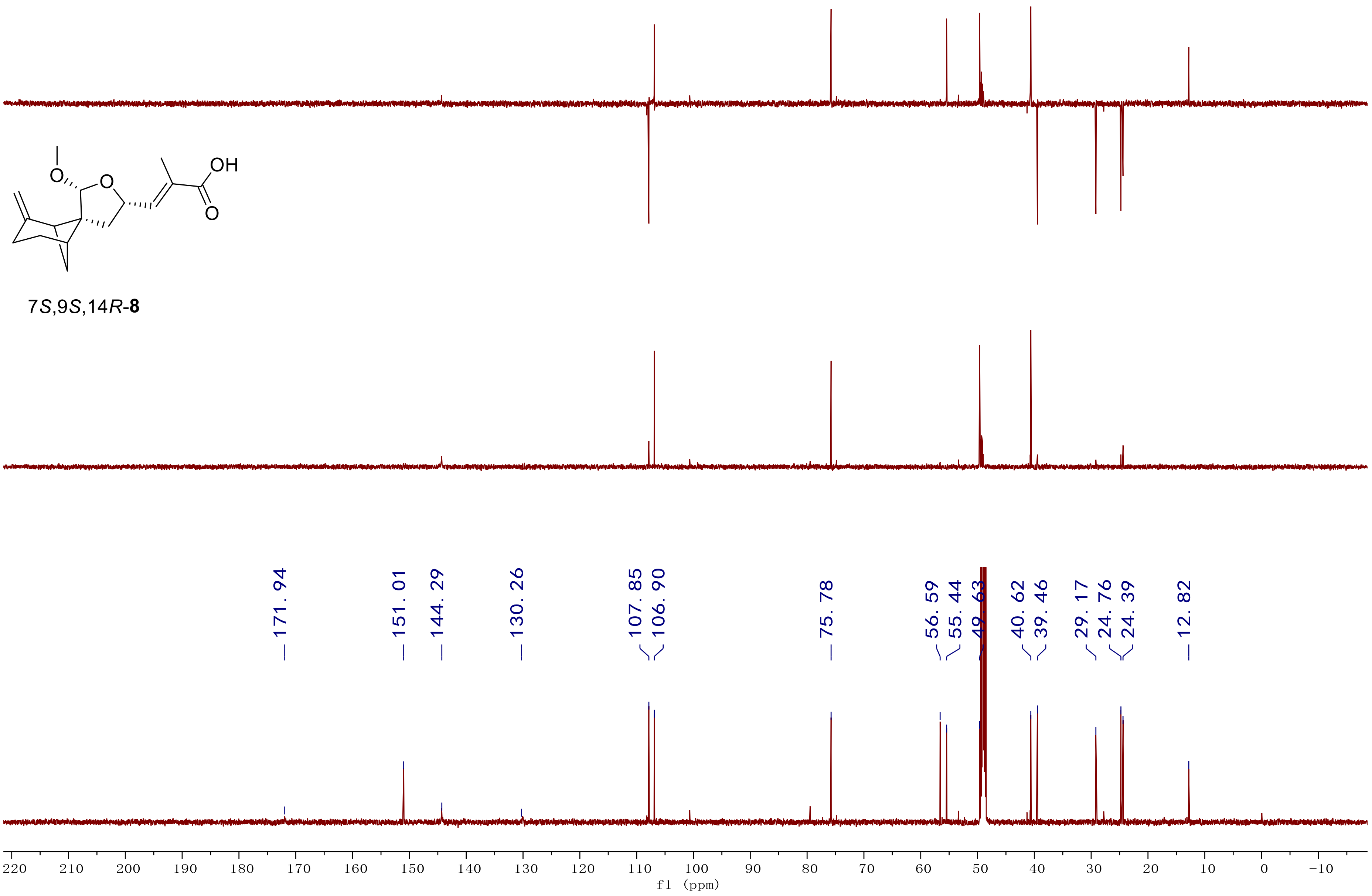

Figure S75. HSQC of compound 7*S*,9*S*,14*R*-**8** in CD_3OD

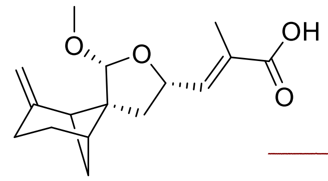

7*S*,9*S*,14*R*-**8**

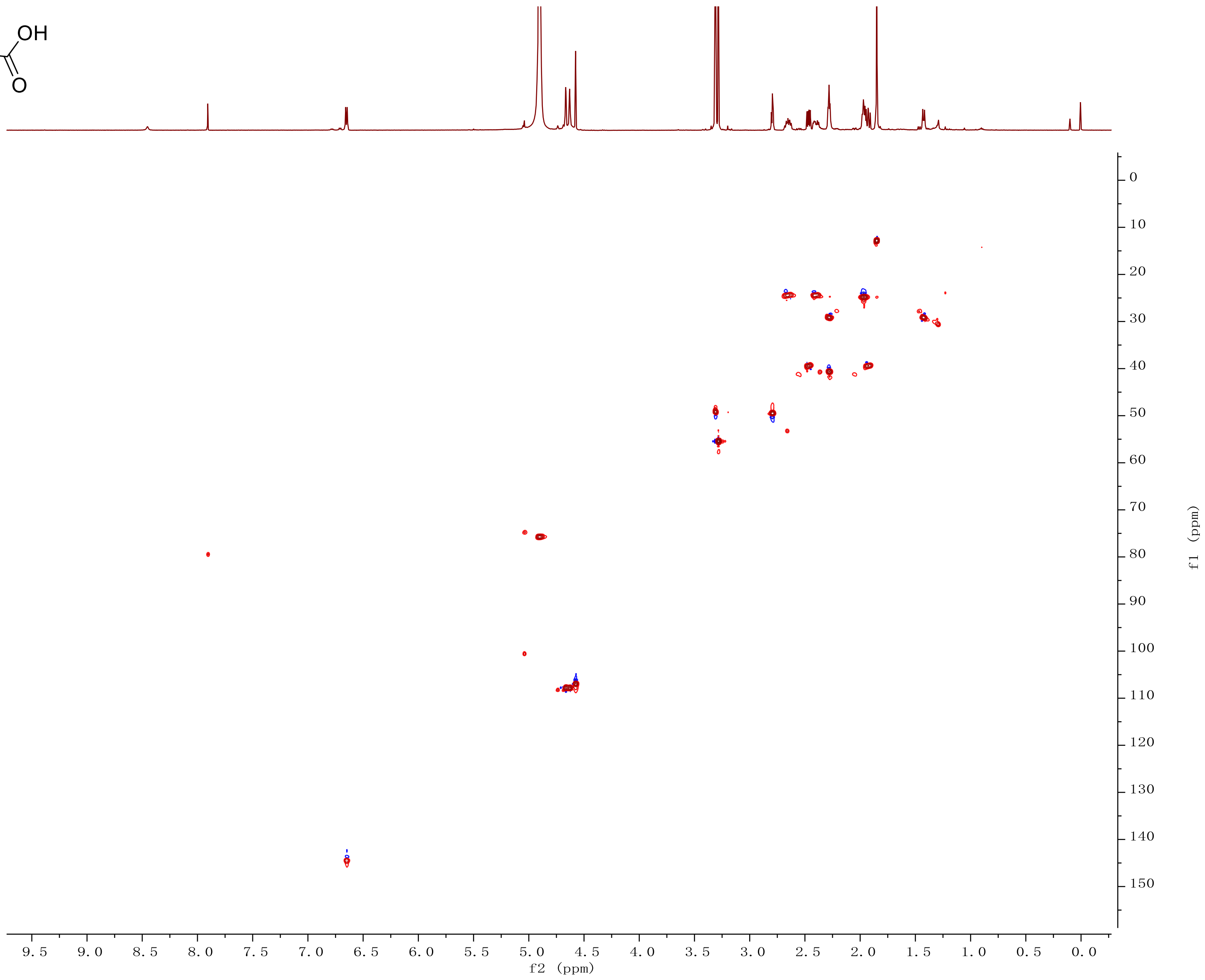

Figure S76. HMBC of compound 7*S*,9*S*,14*R*-**8** in CD_3OD

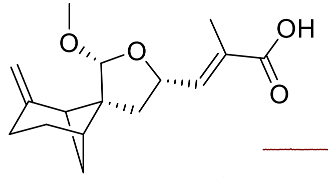

7*S*,9*S*,14*R*-**8**

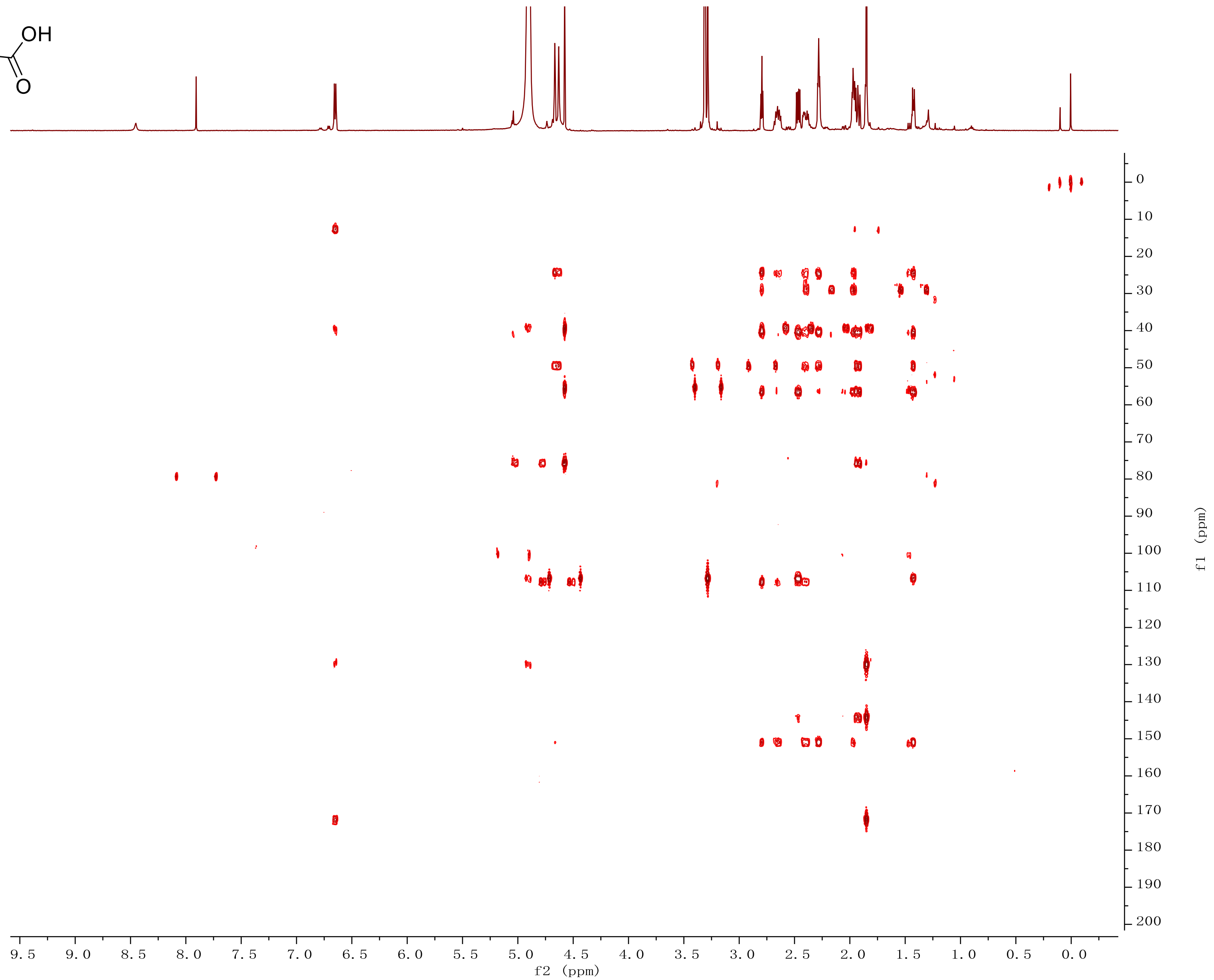

Figure S77. ^1H-^1H COSY of compound 7*S*,9*S*,14*R*-**8** in CD_3OD

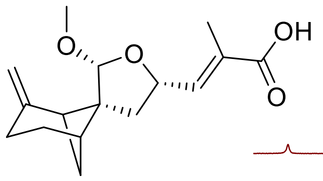

7*S*,9*S*,14*R*-**8**

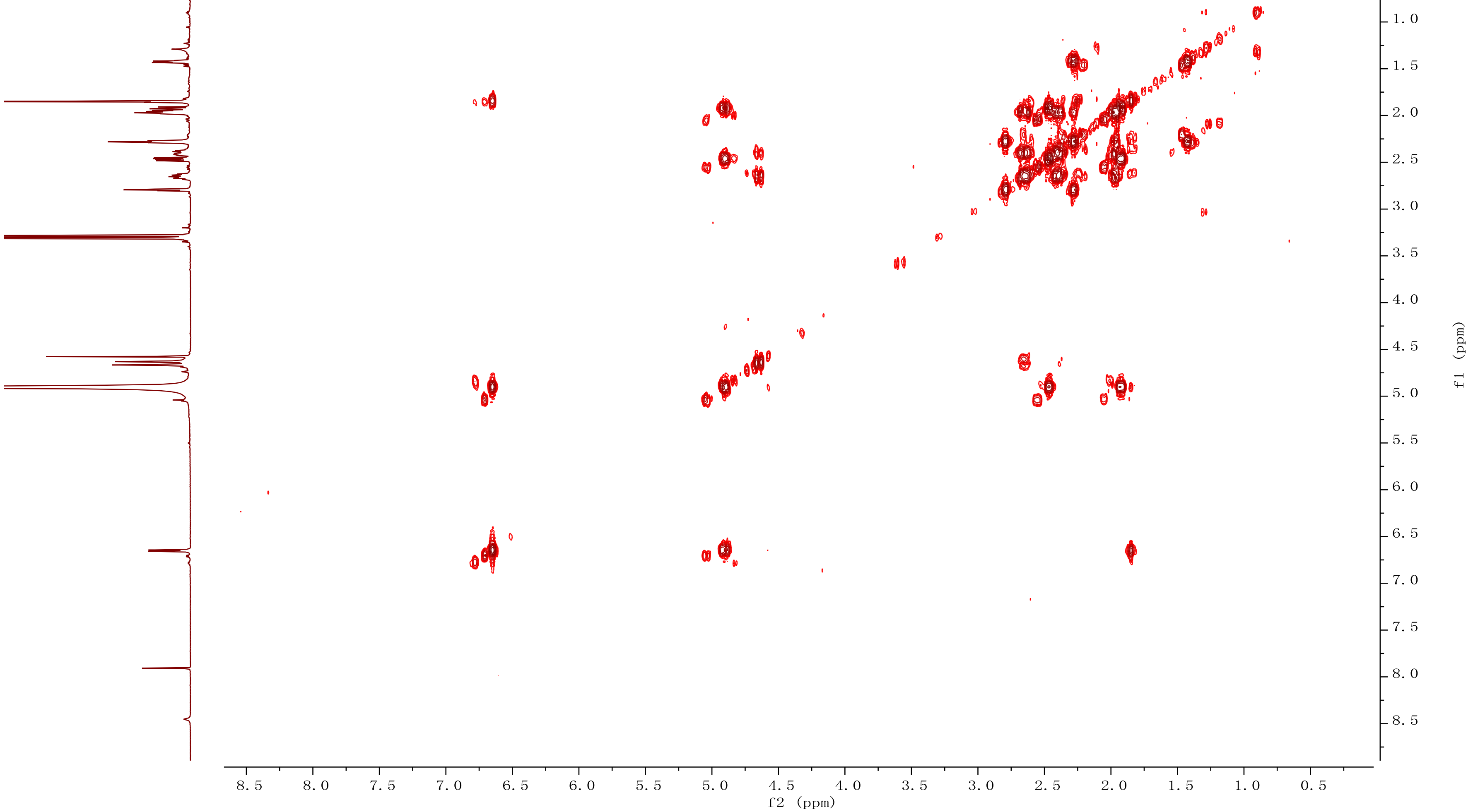

Figure S78. ROESY of compound 7*S*,9*S*,14*R*-**8** in CD_3OD

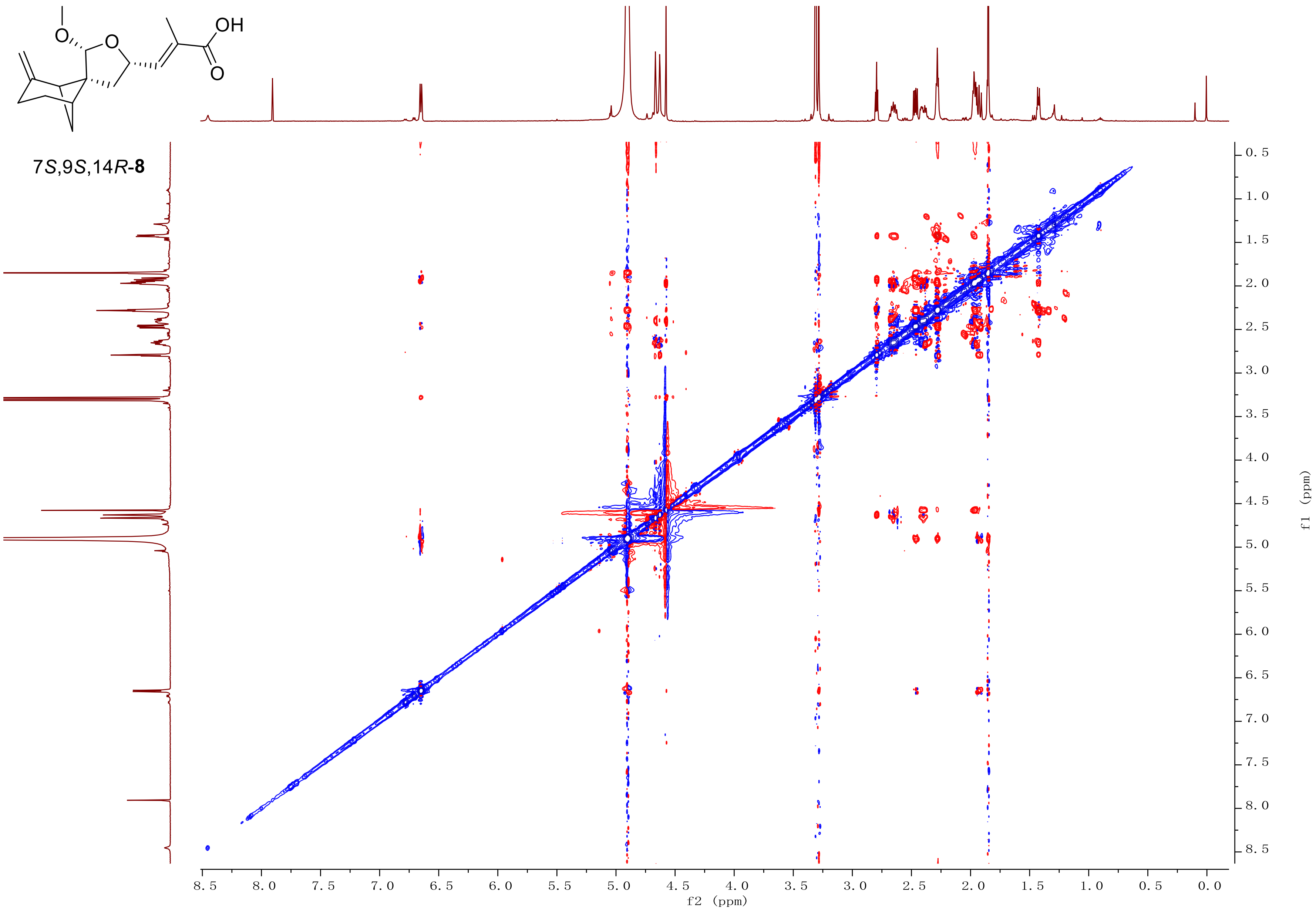

Figure S79. HR-ESIMS of compound 7*S*,9*S*,14*R*-**8**

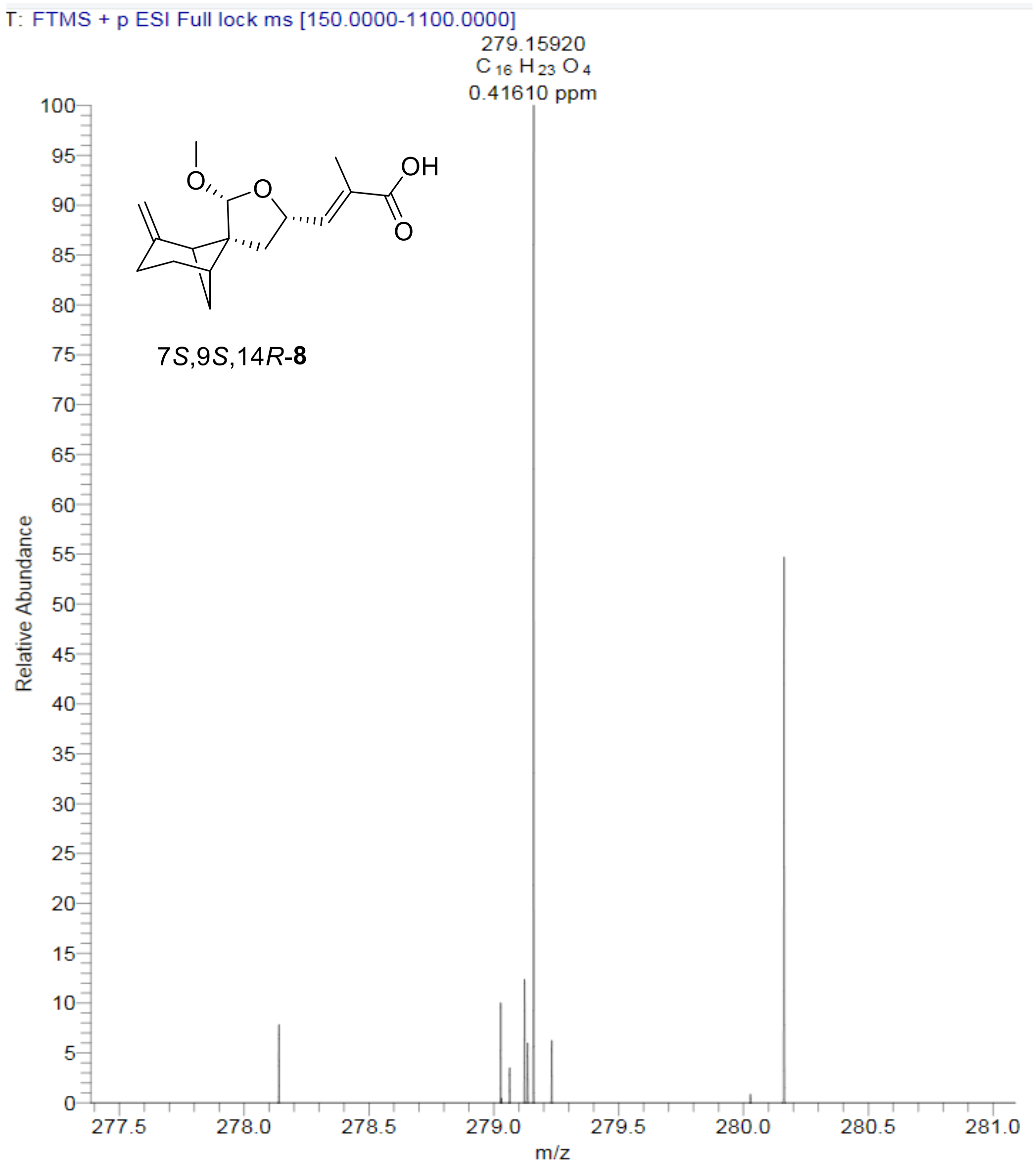

Section S16. NMR and MS spectra for 9

Figure S80. ^{1}H NMR of compound **9** in CD_3OD

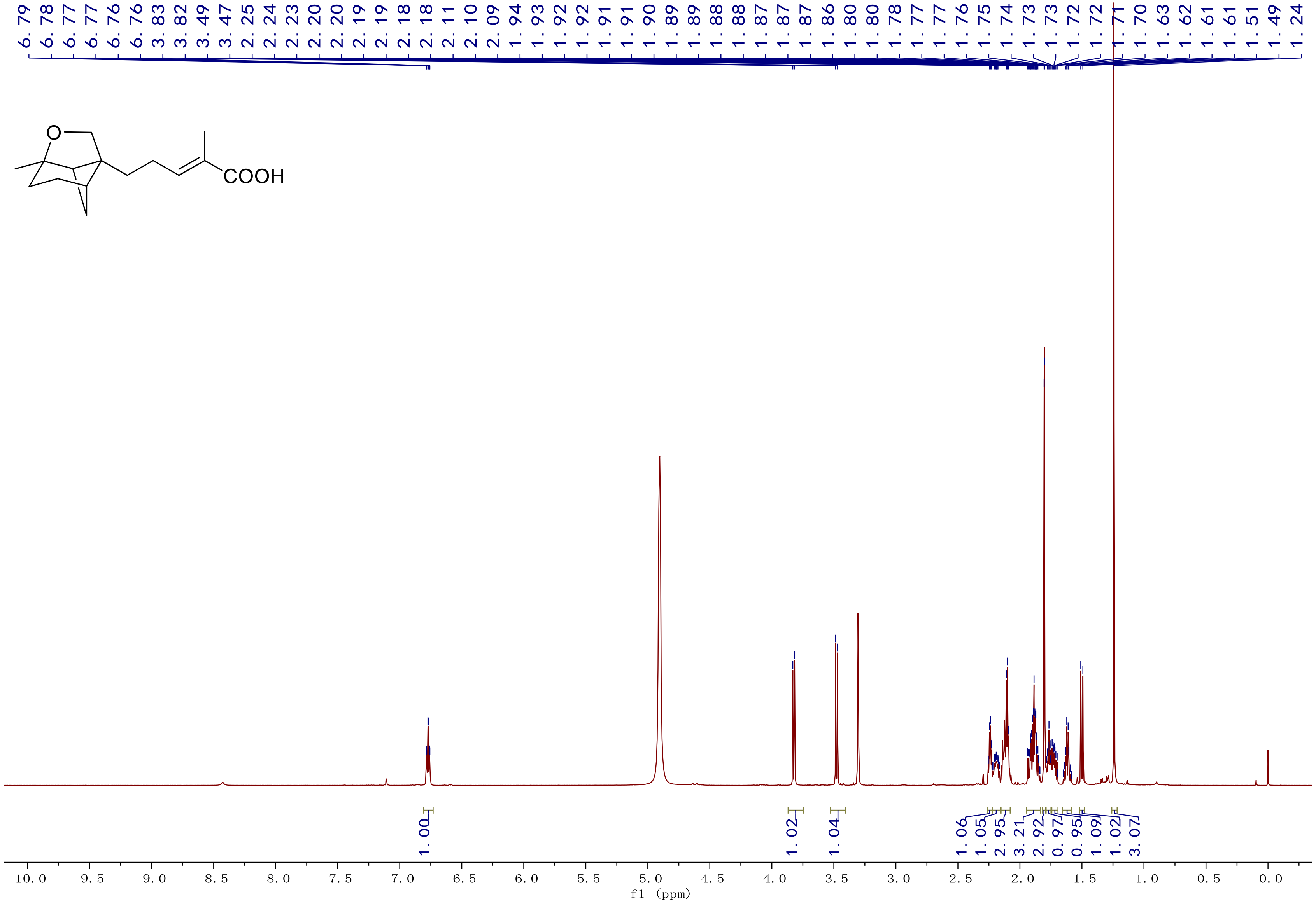

Figure S81. ^{13}C NMR and DEPT of compound **9** in CD_3OD

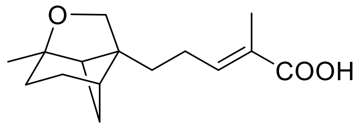

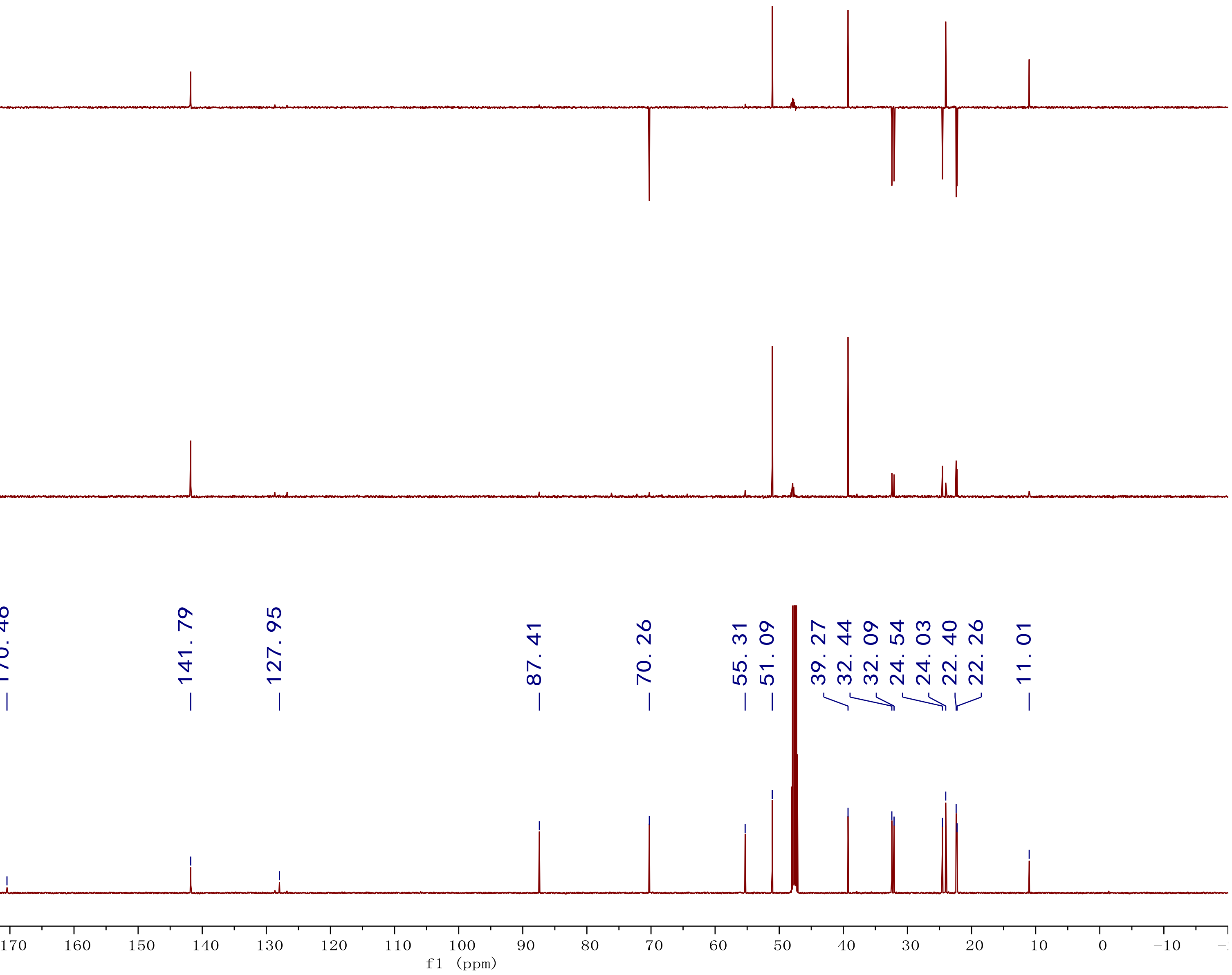

Figure S82. HSQC of compound **9** in CD_3OD

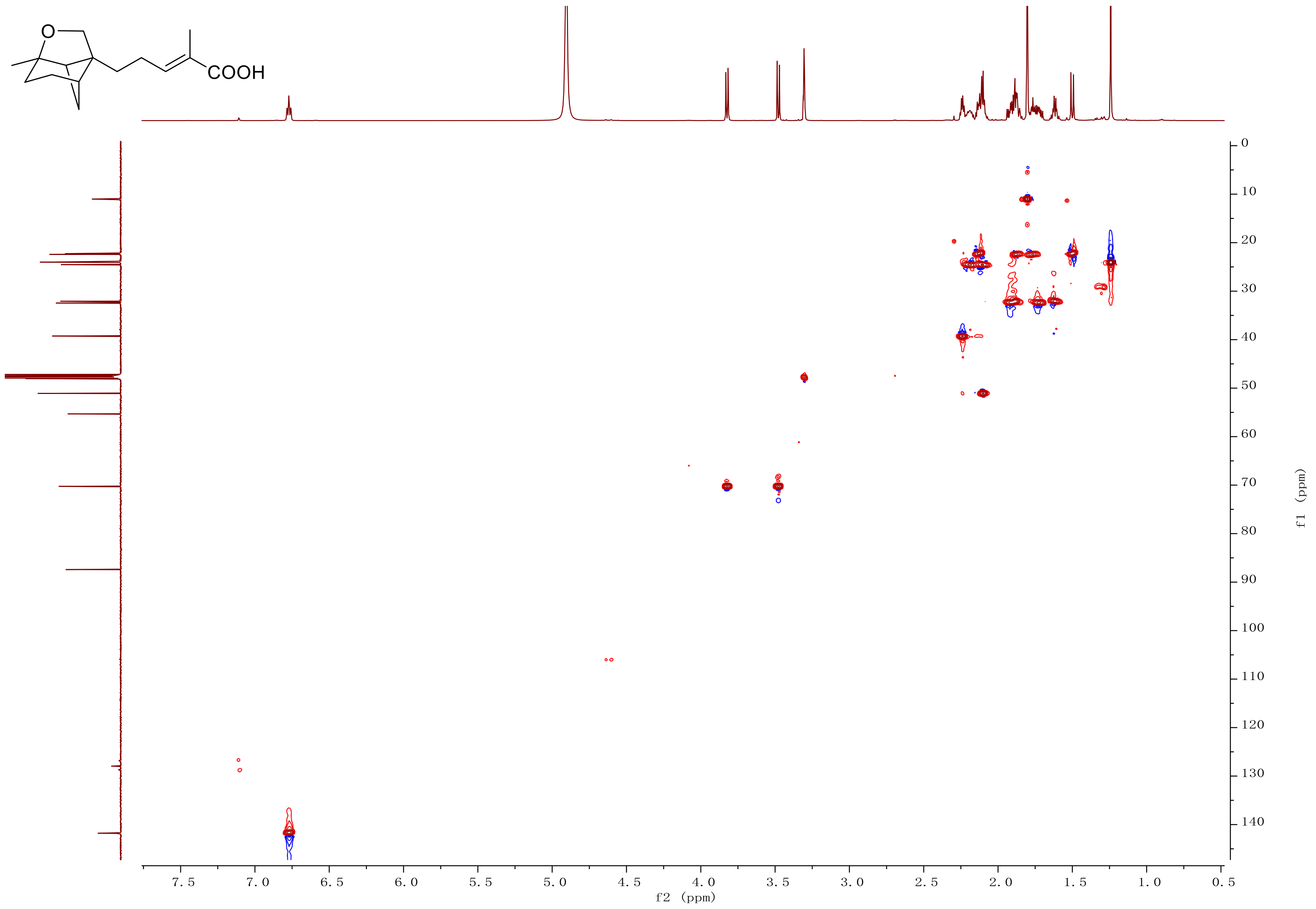

Figure S83. HMBC of compound **9** in CD_3OD

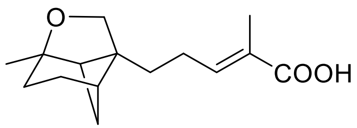

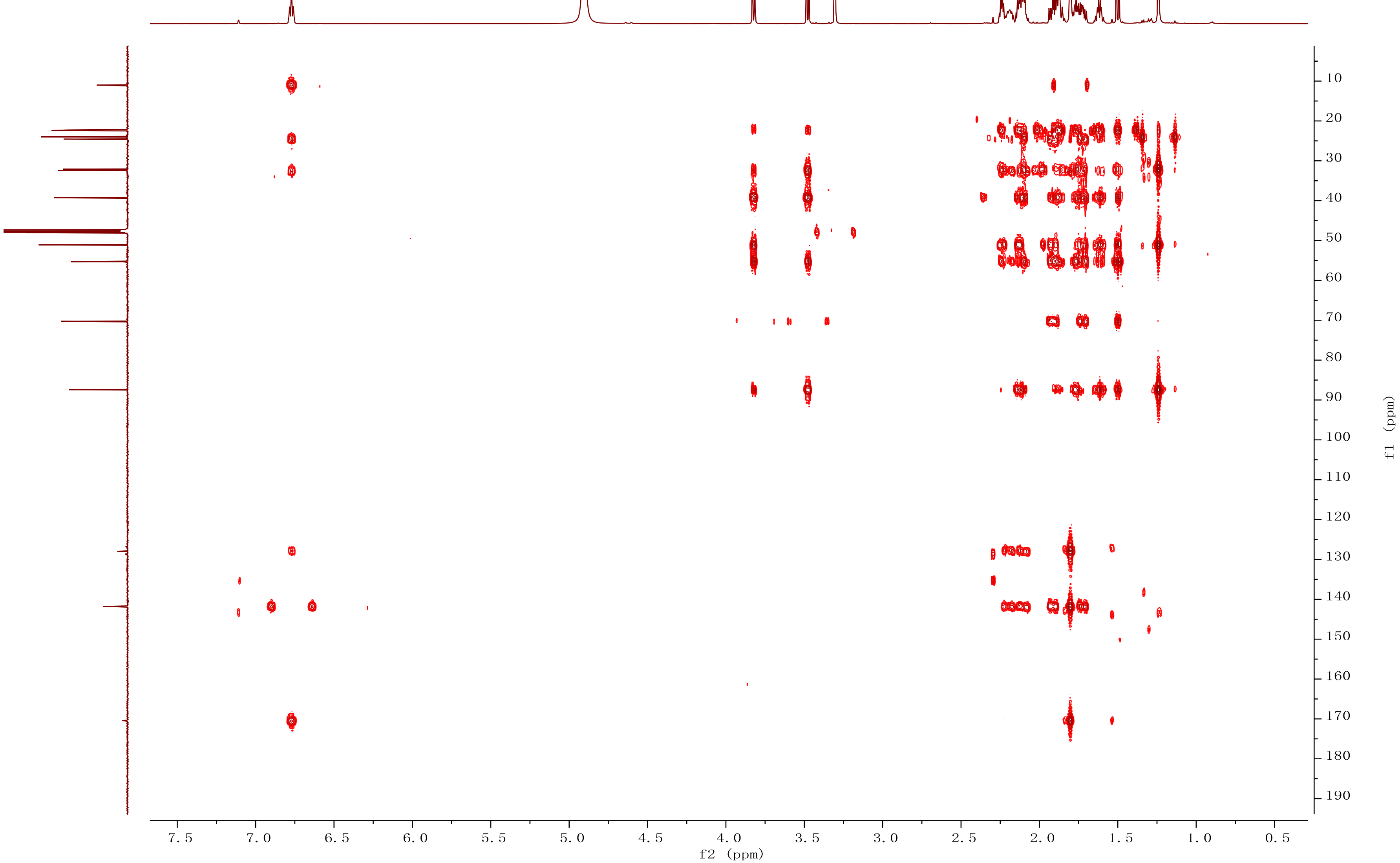

Figure S84. ^{1}H-^{1}H COSY of compound **9** in CD_3OD

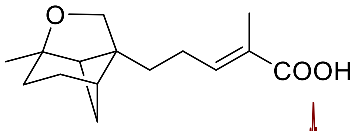

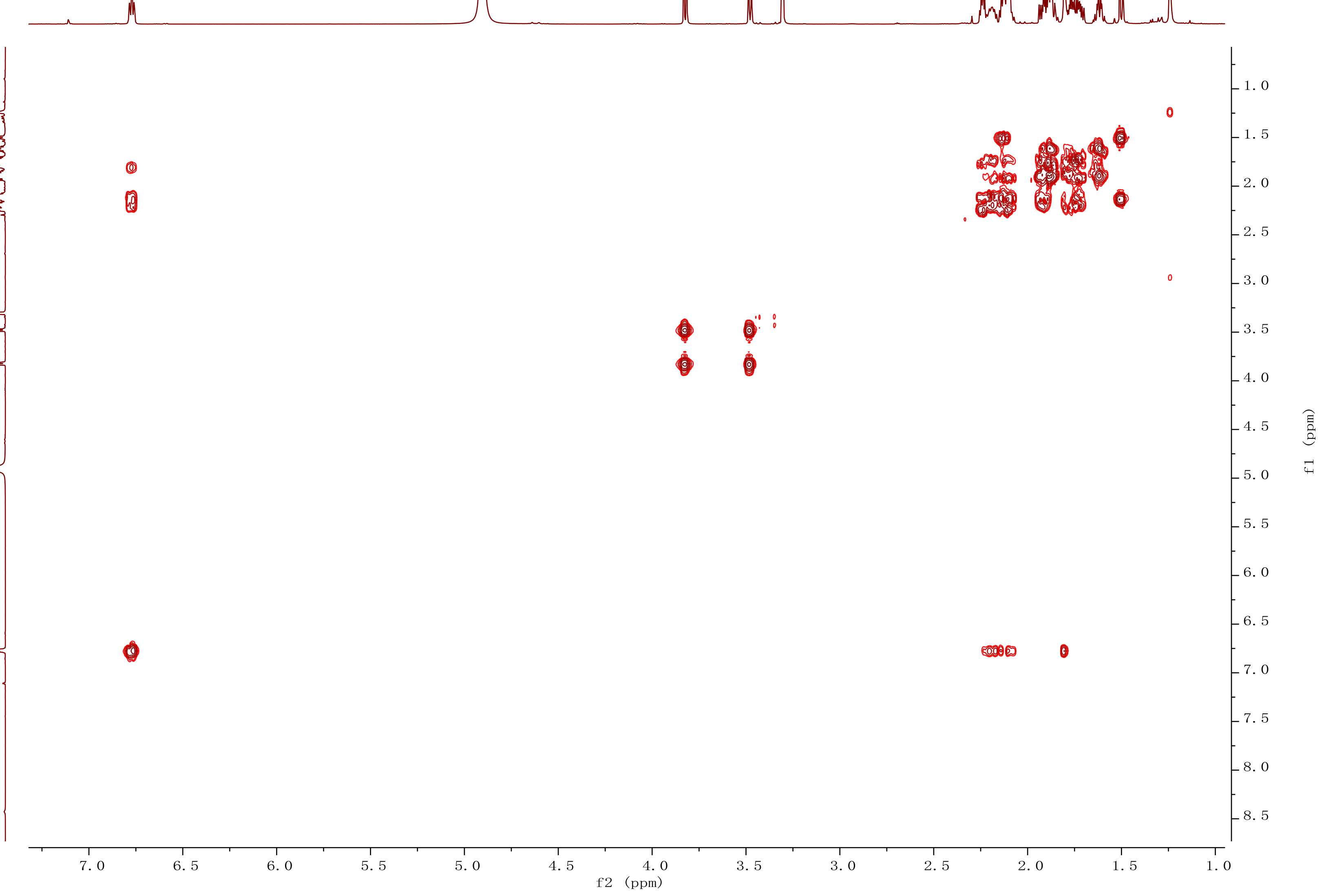

Figure S85. ROESY of compound **9** in CD_3OD

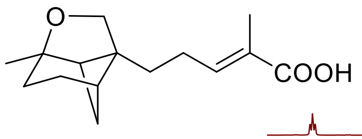

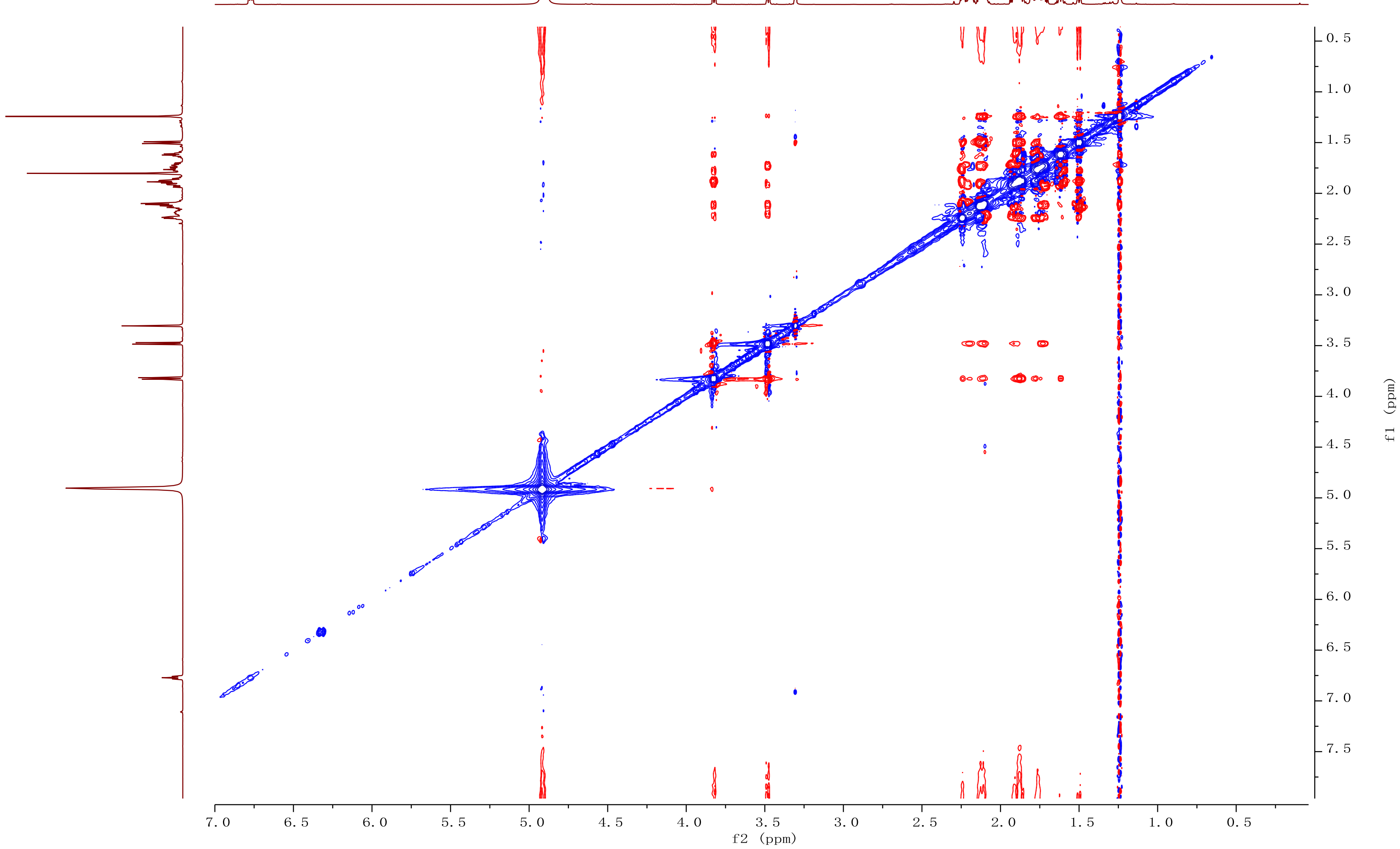

Figure S86. HR-ESIMS of compound **9**

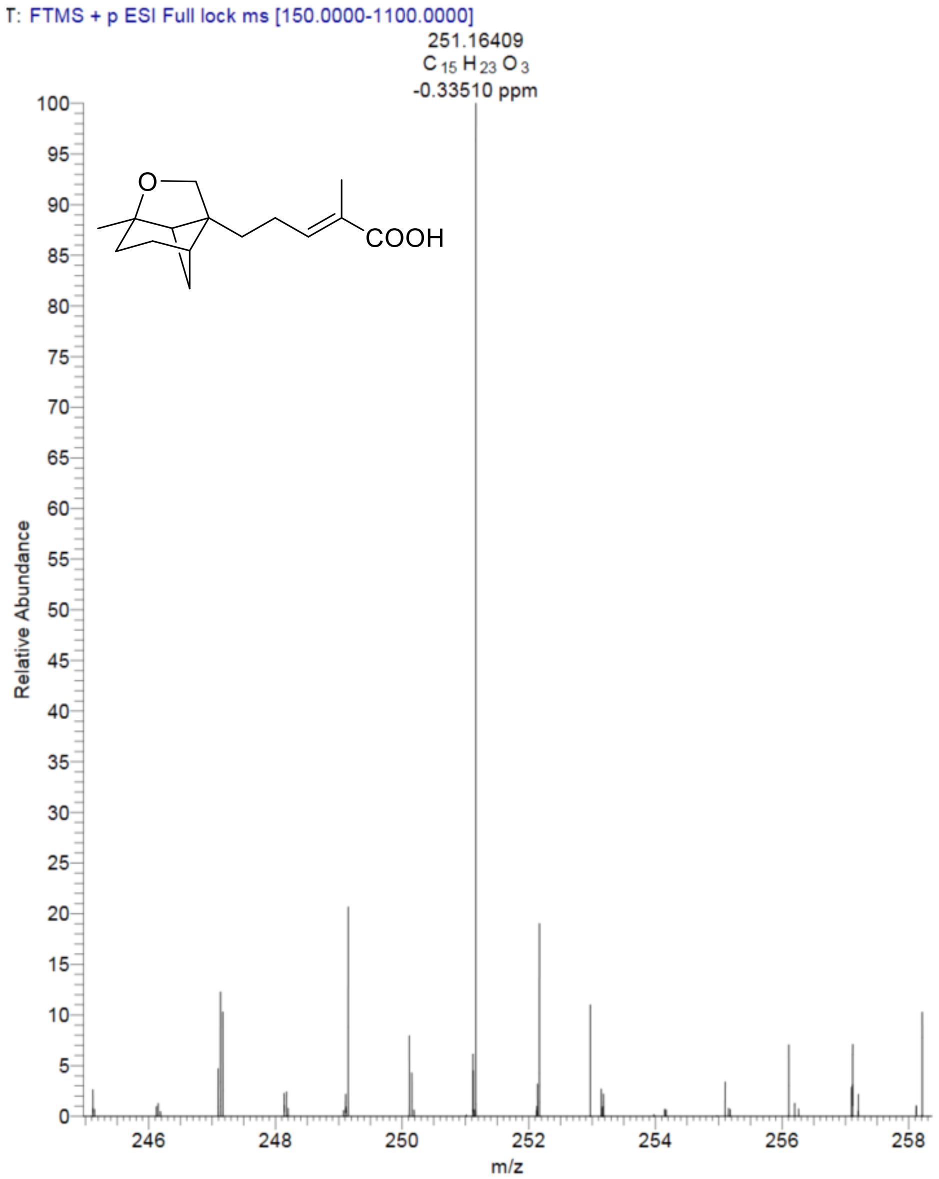

Section S17. NMR and MS spectra for 10

Figure S87. ^{1}H NMR of compound **10** in CD_3OD

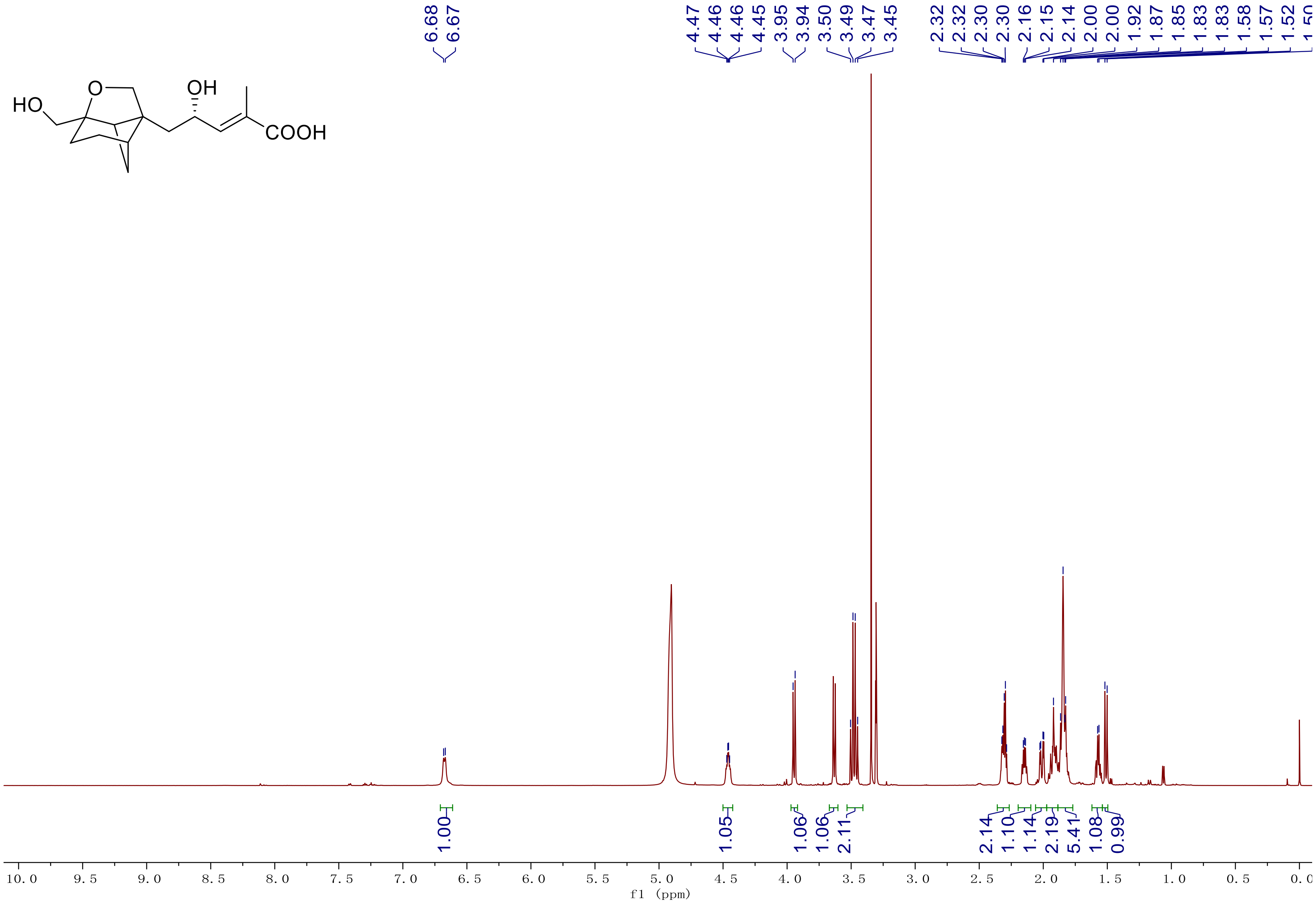

Figure S88. ^{13}C NMR and DEPT of compound **10** in CD_3OD

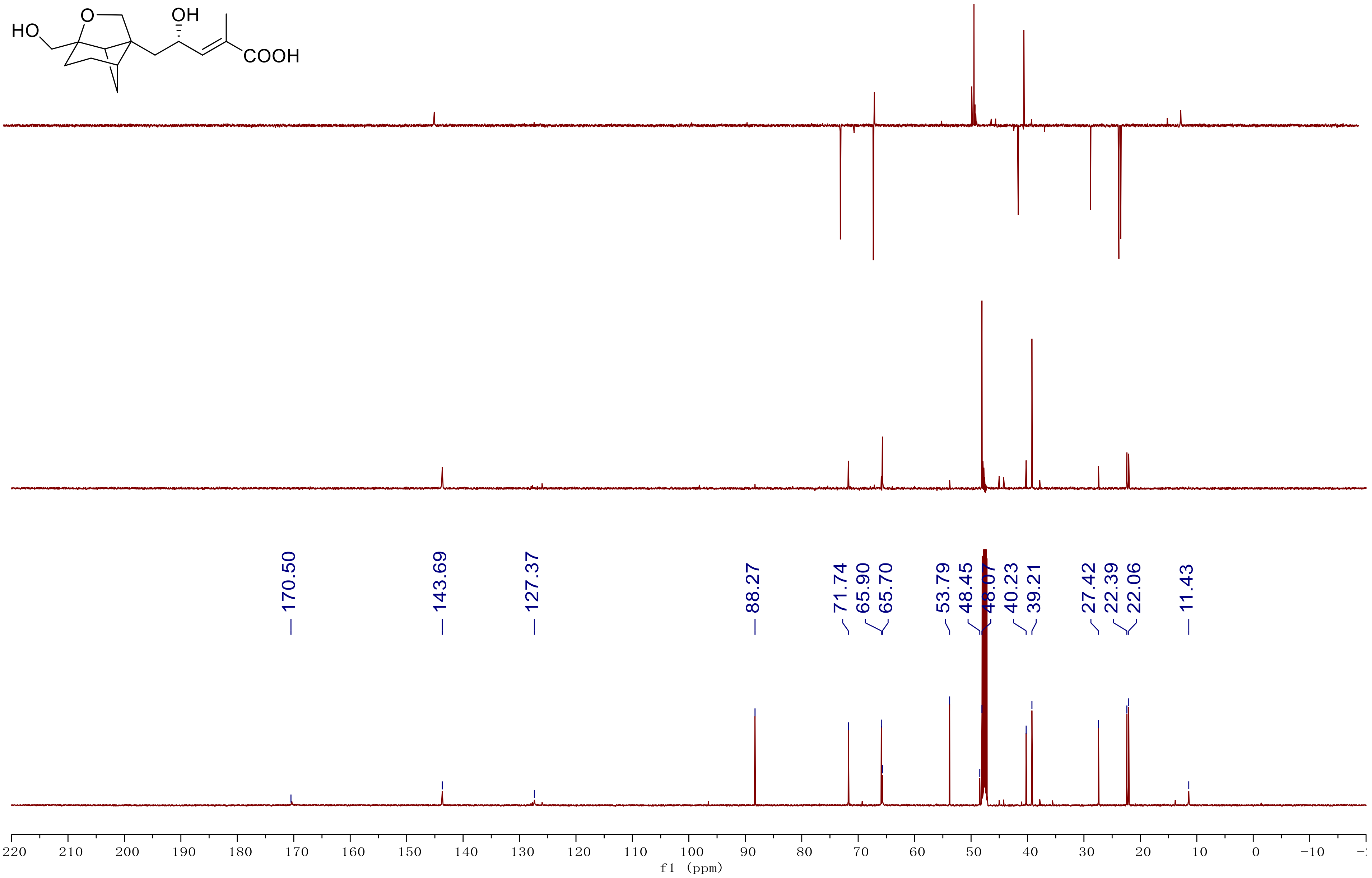

Figure S89. HSQC of compound **10** in CD_3OD

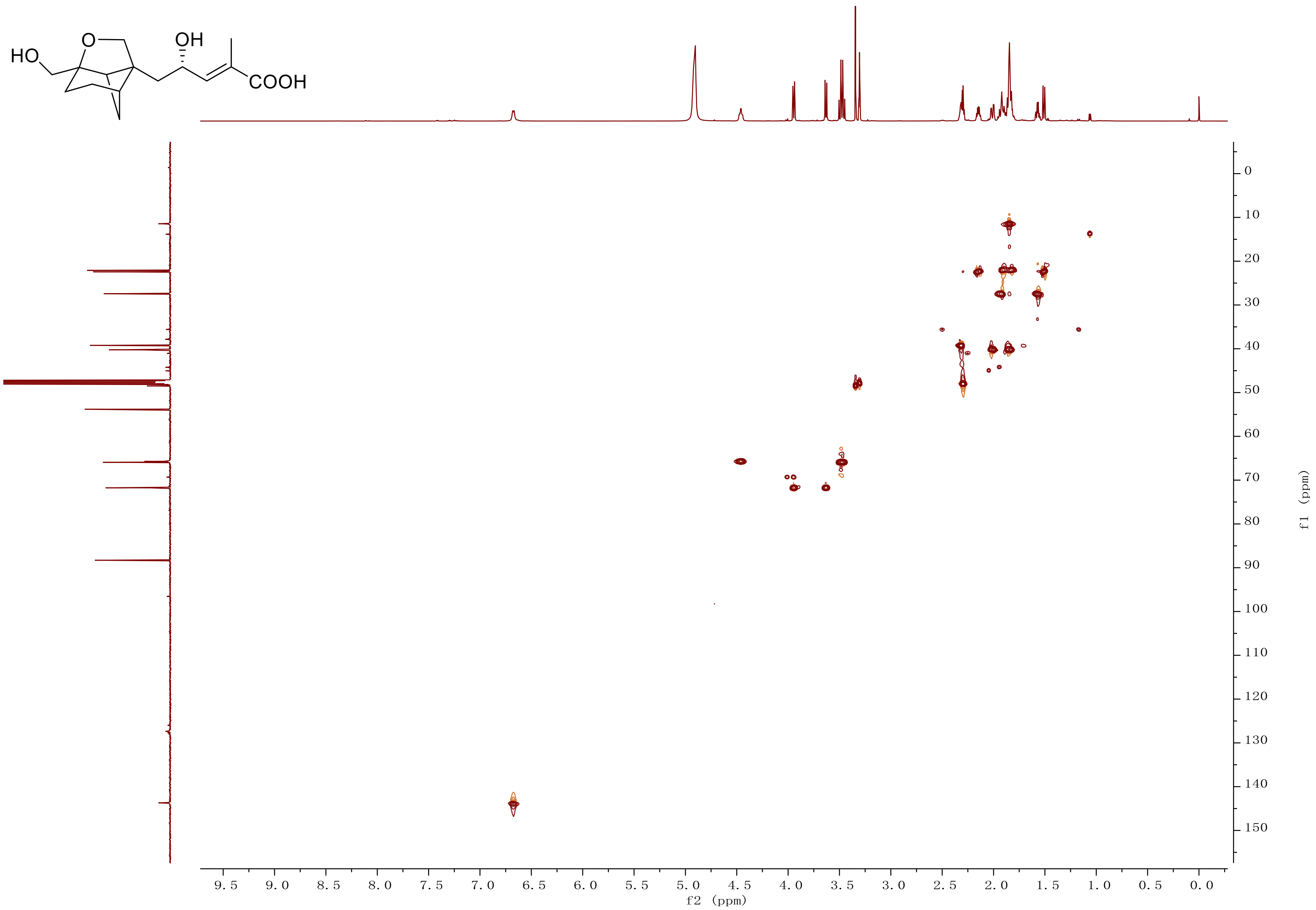

Figure S90. HMBC of compound **10** in CD_3OD

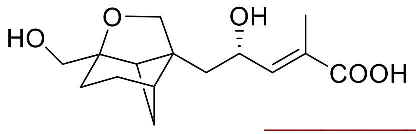

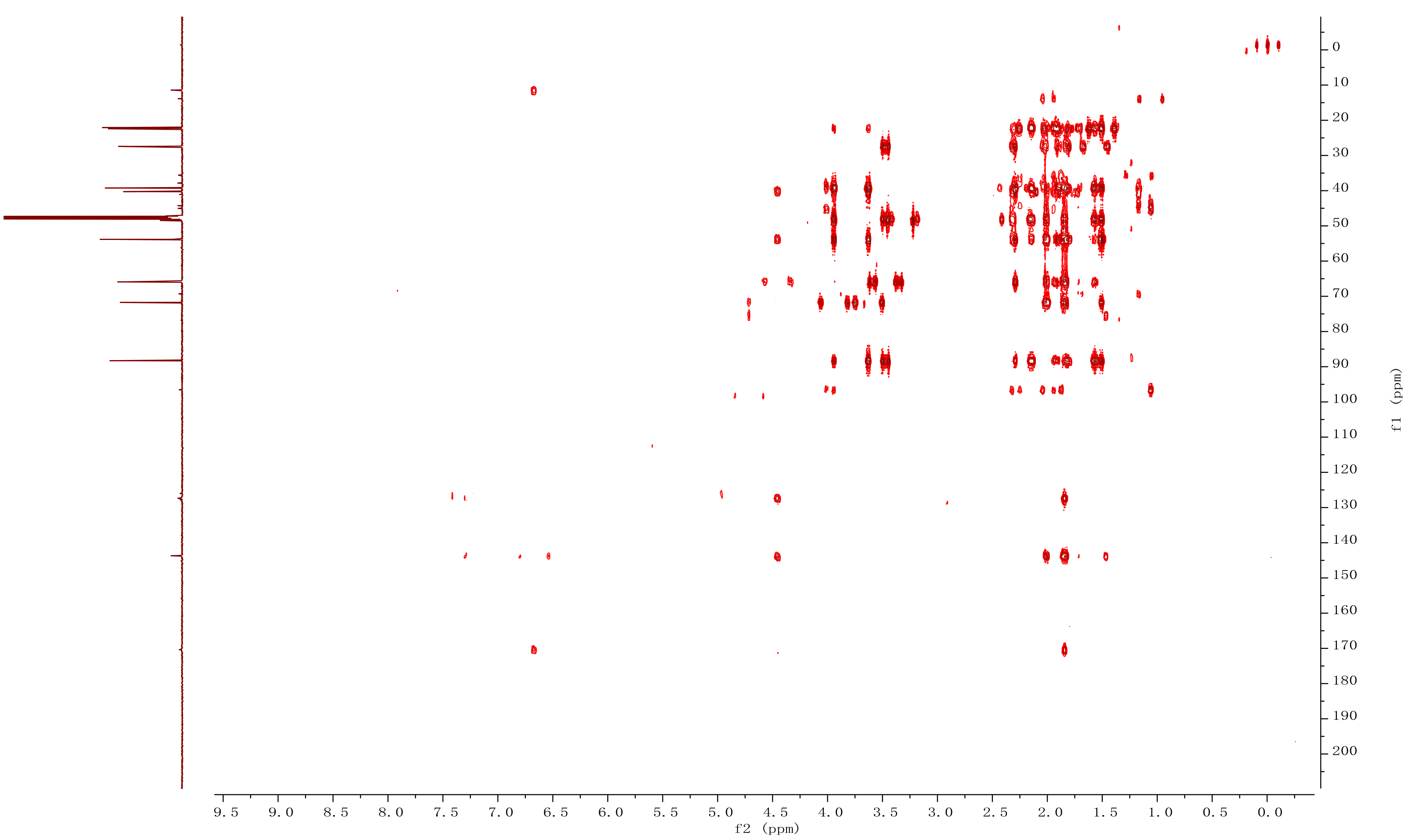

Figure S91. ^{1}H-^{1}H COSY of compound **10** in CD_3OD

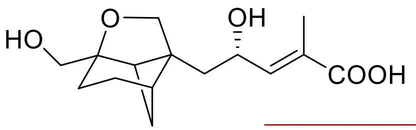

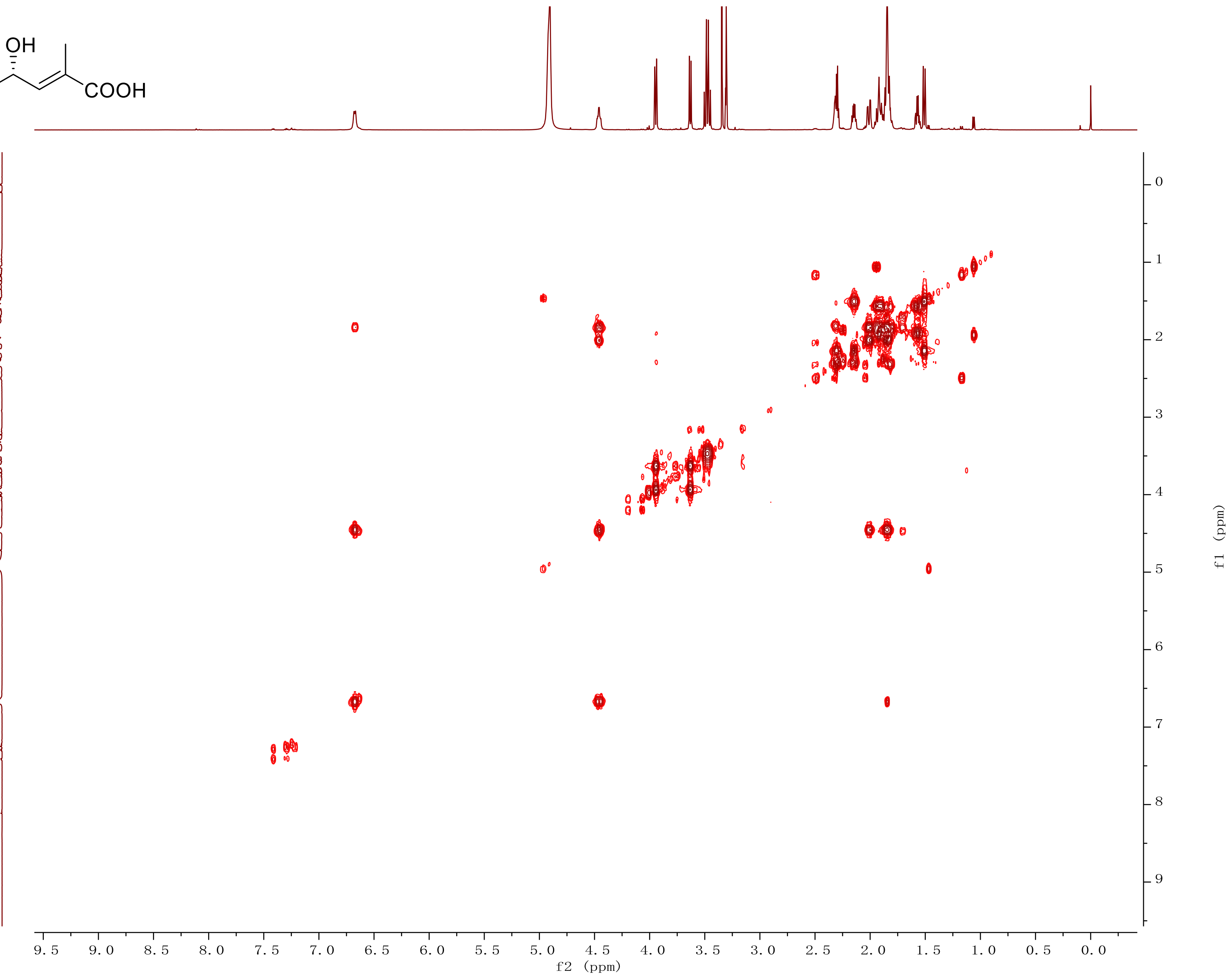

Figure S92. ROESY of compound **10** in CD_3OD

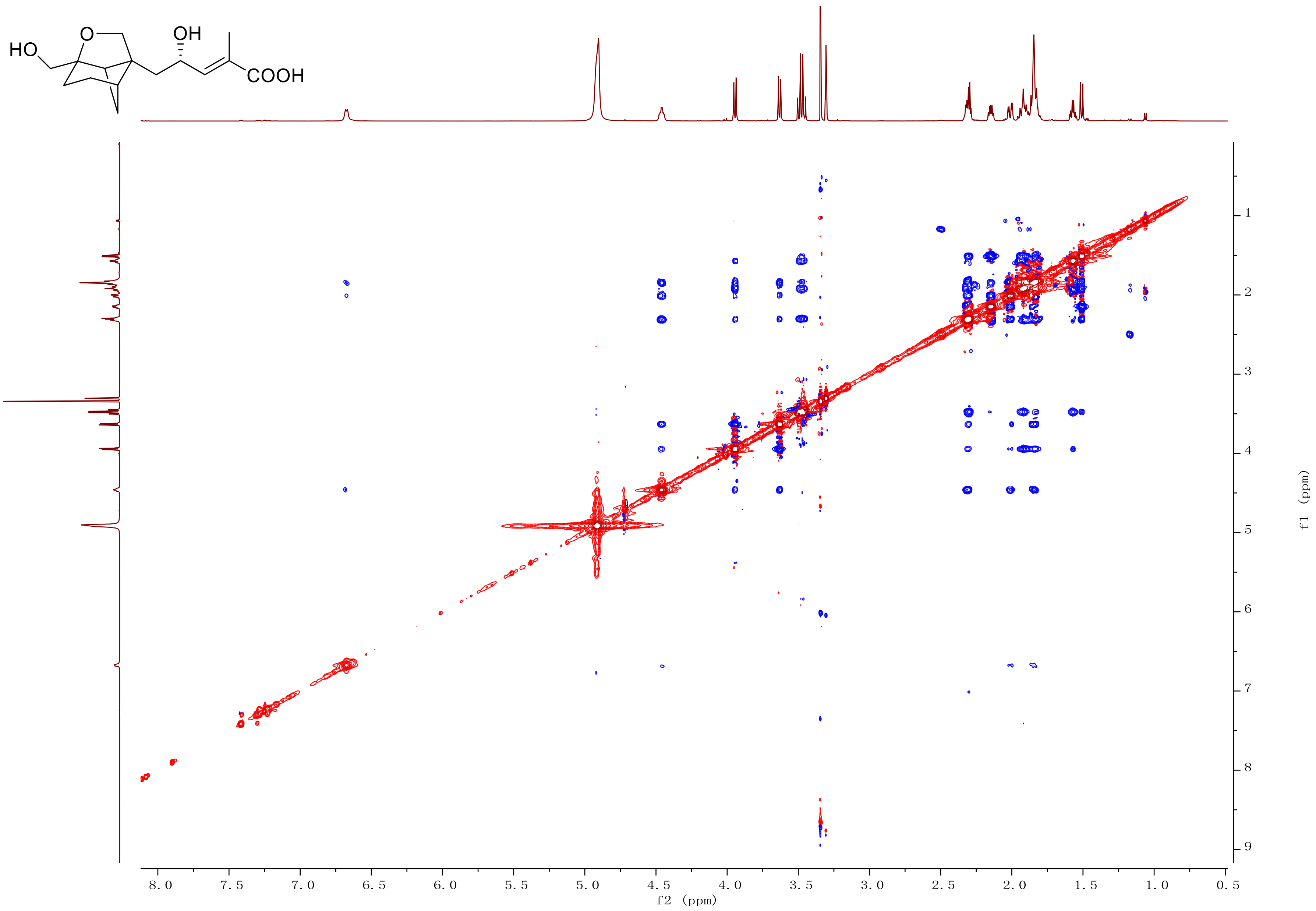

Figure S93. HR-ESIMS of compound **10**

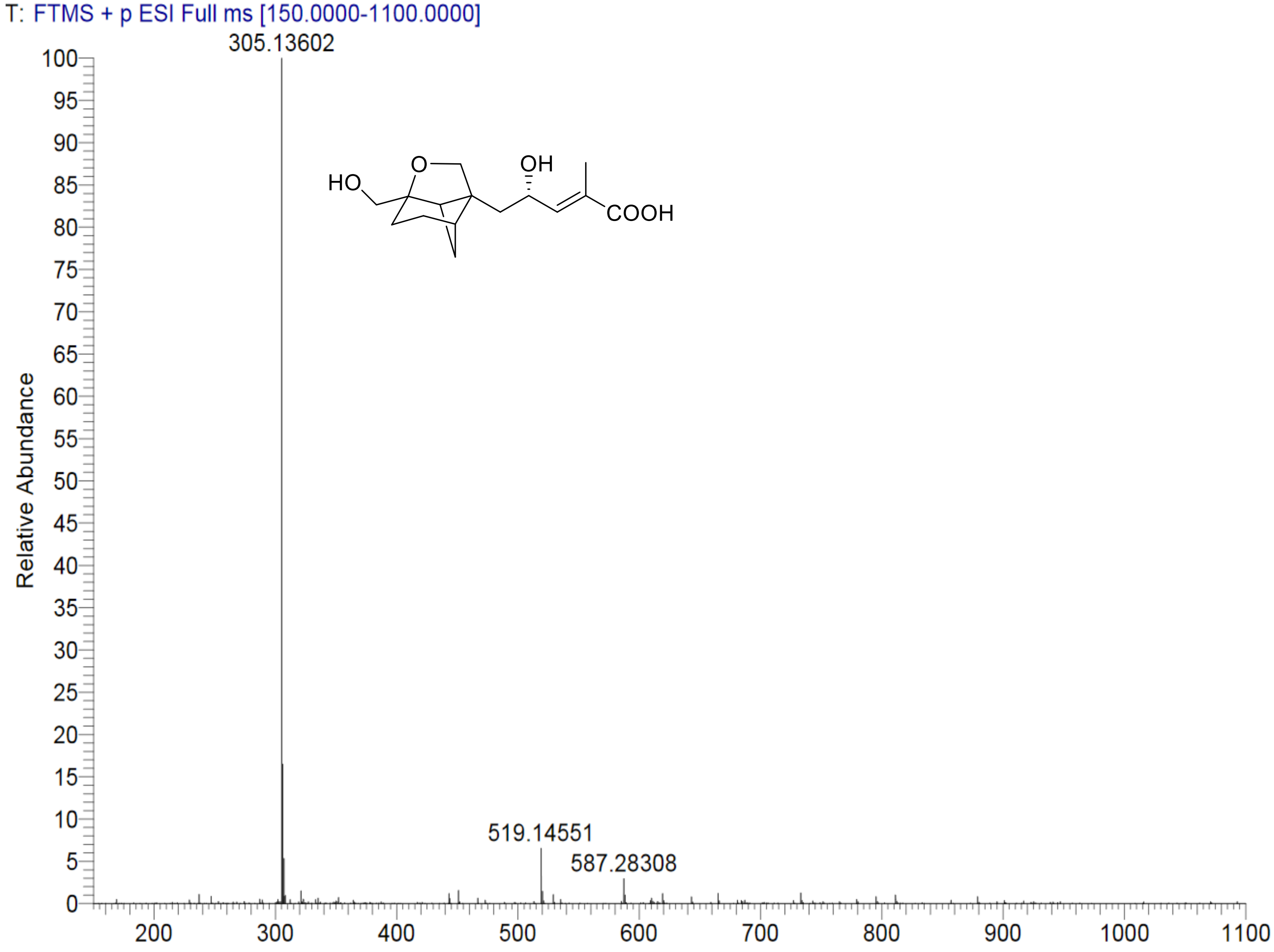

Section S18. NMR and MS spectra for 11

Figure S94. ^{1}H NMR of compound **11** in CD_3OD

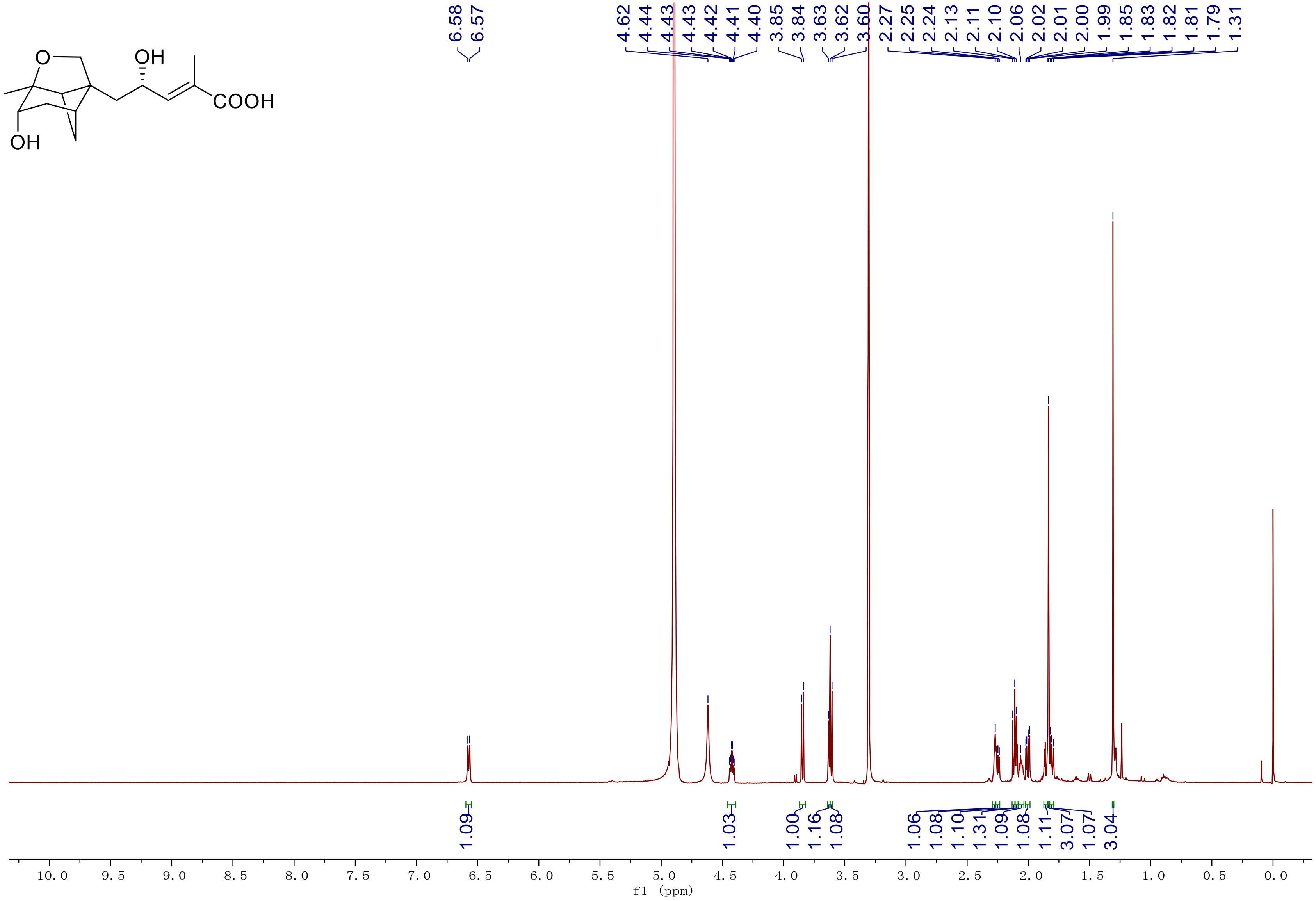

Figure S95. ^{13}C NMR and DEPT of compound **11** in CD_3OD

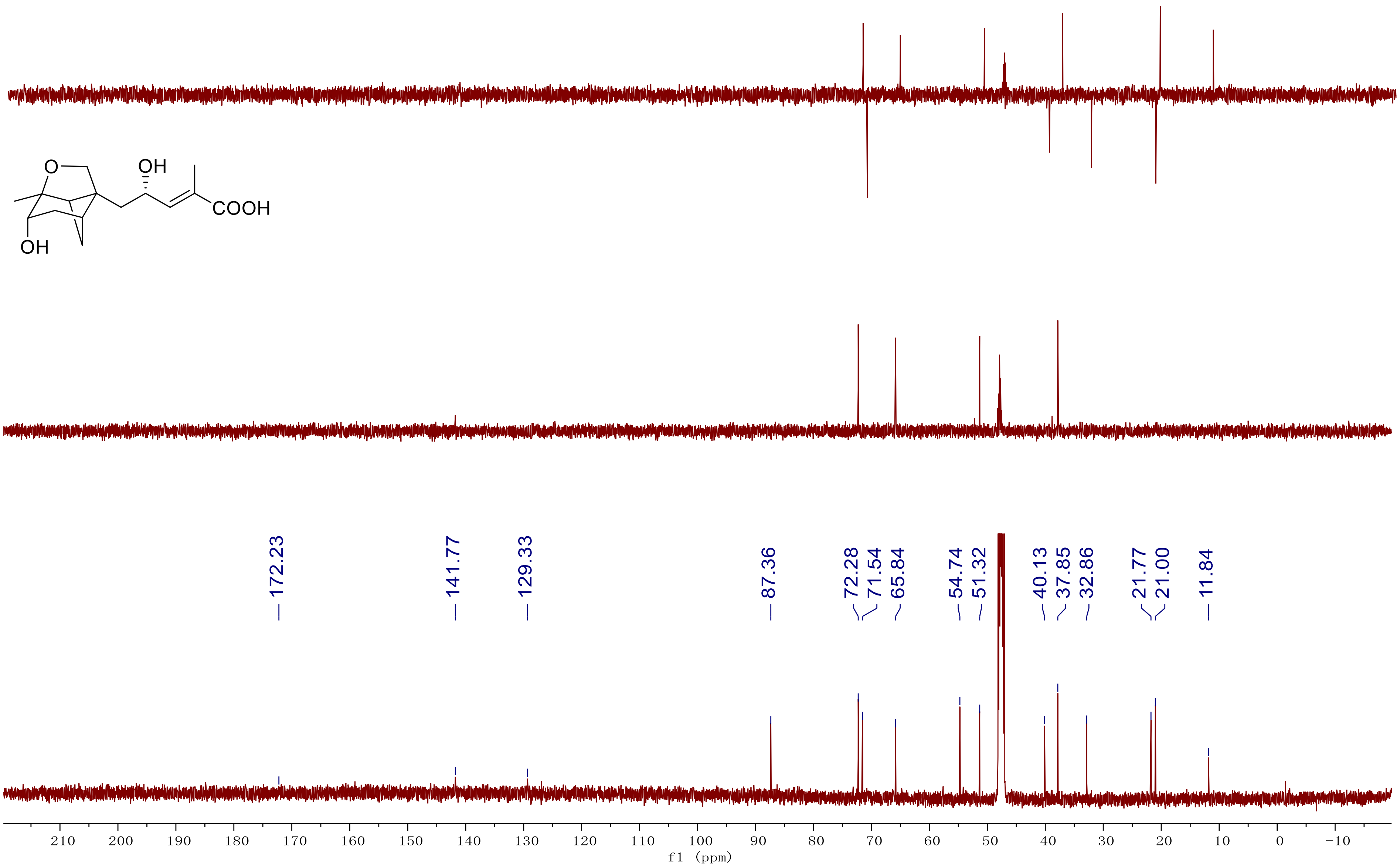

Figure S96. HSQC of compound **11** in CD_3OD

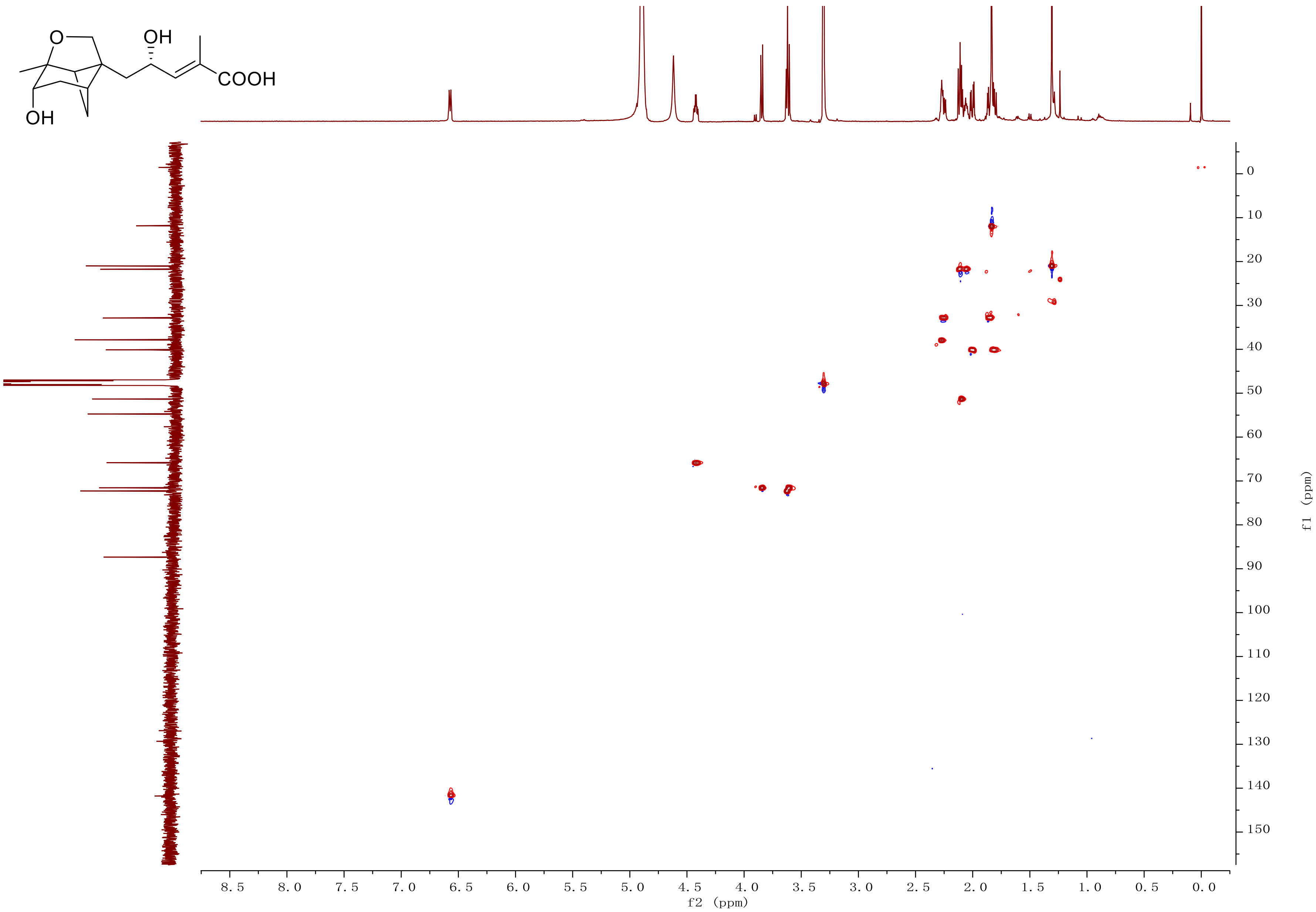

Figure S97. HMBC of compound **11** in CD_3OD

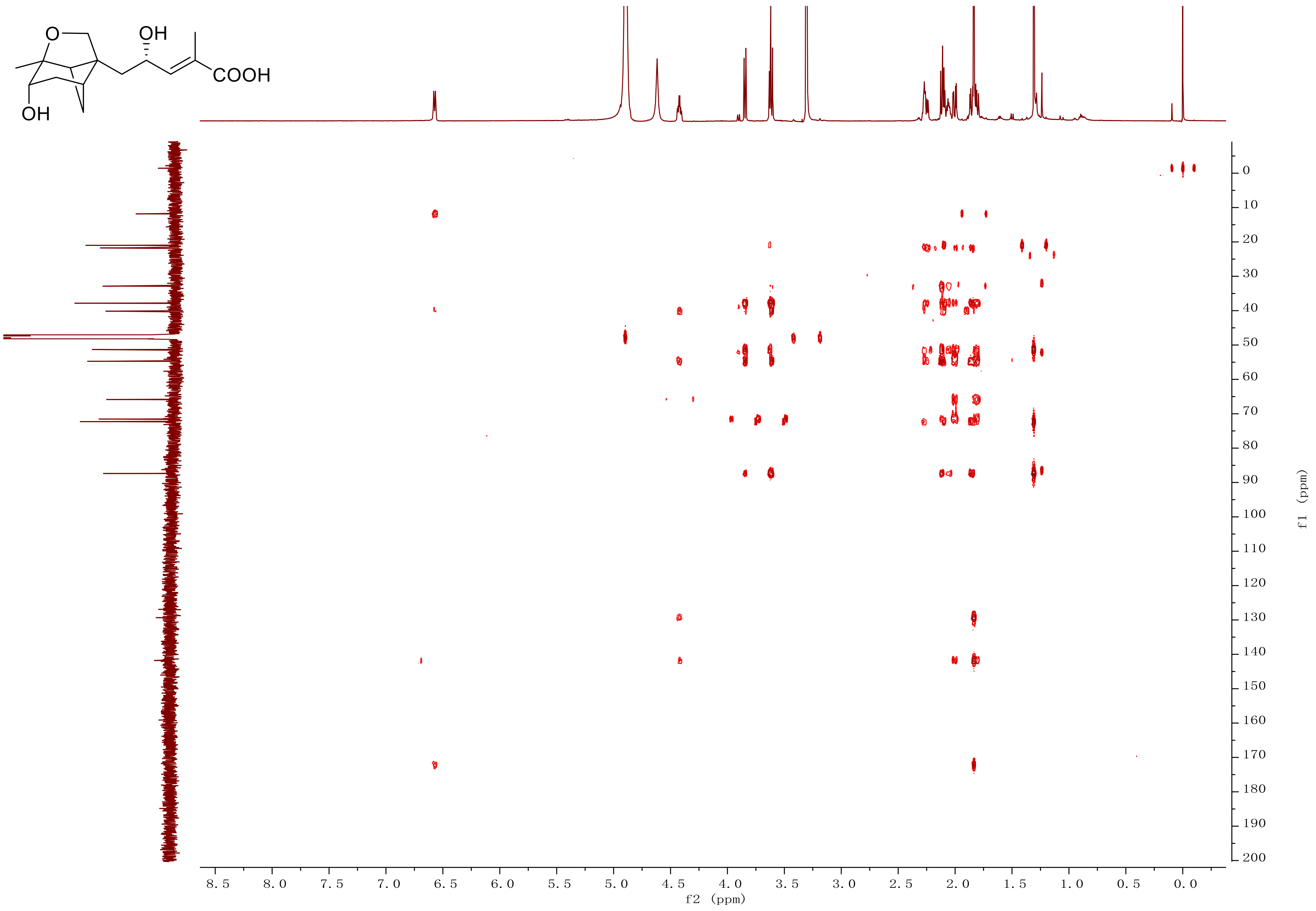

Figure S98. ^{1}H-^{1}H COSY of compound **11** in CD_3OD

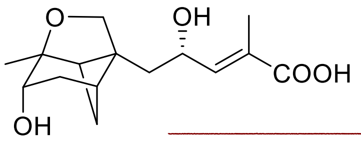

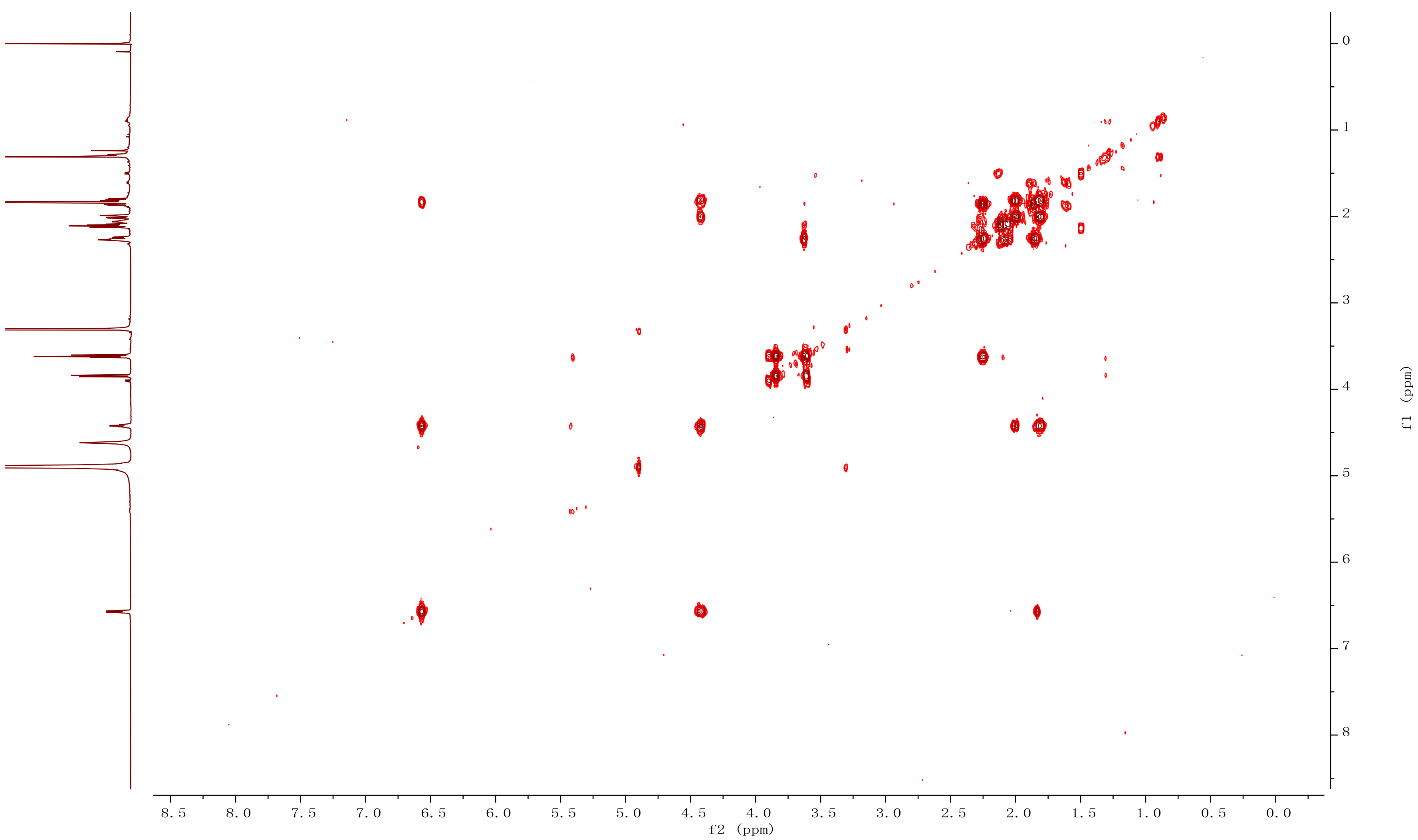

Figure S99. ROESY of compound **11** in CD_3OD

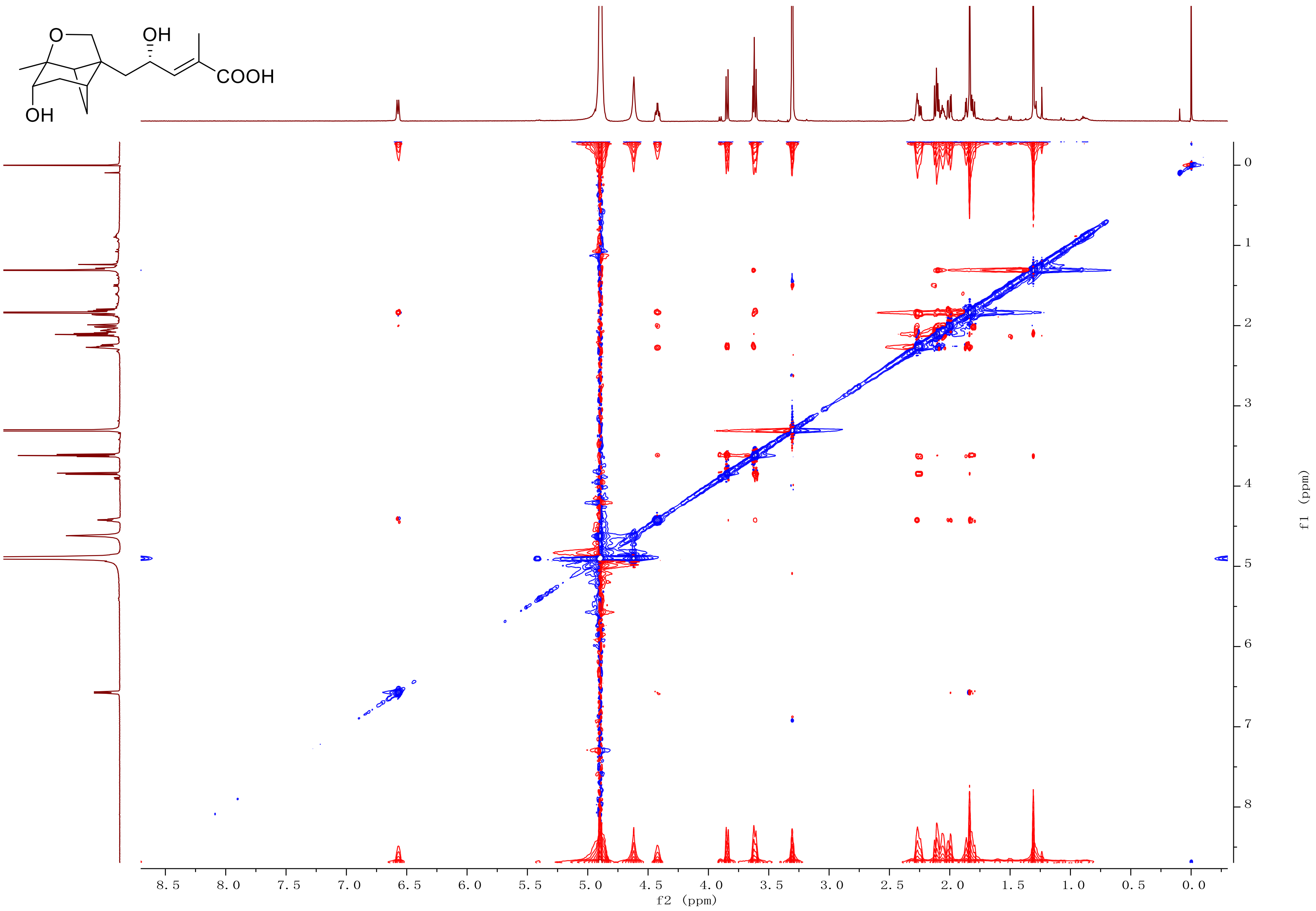

Figure S100. HR-ESIMS of compound **11**

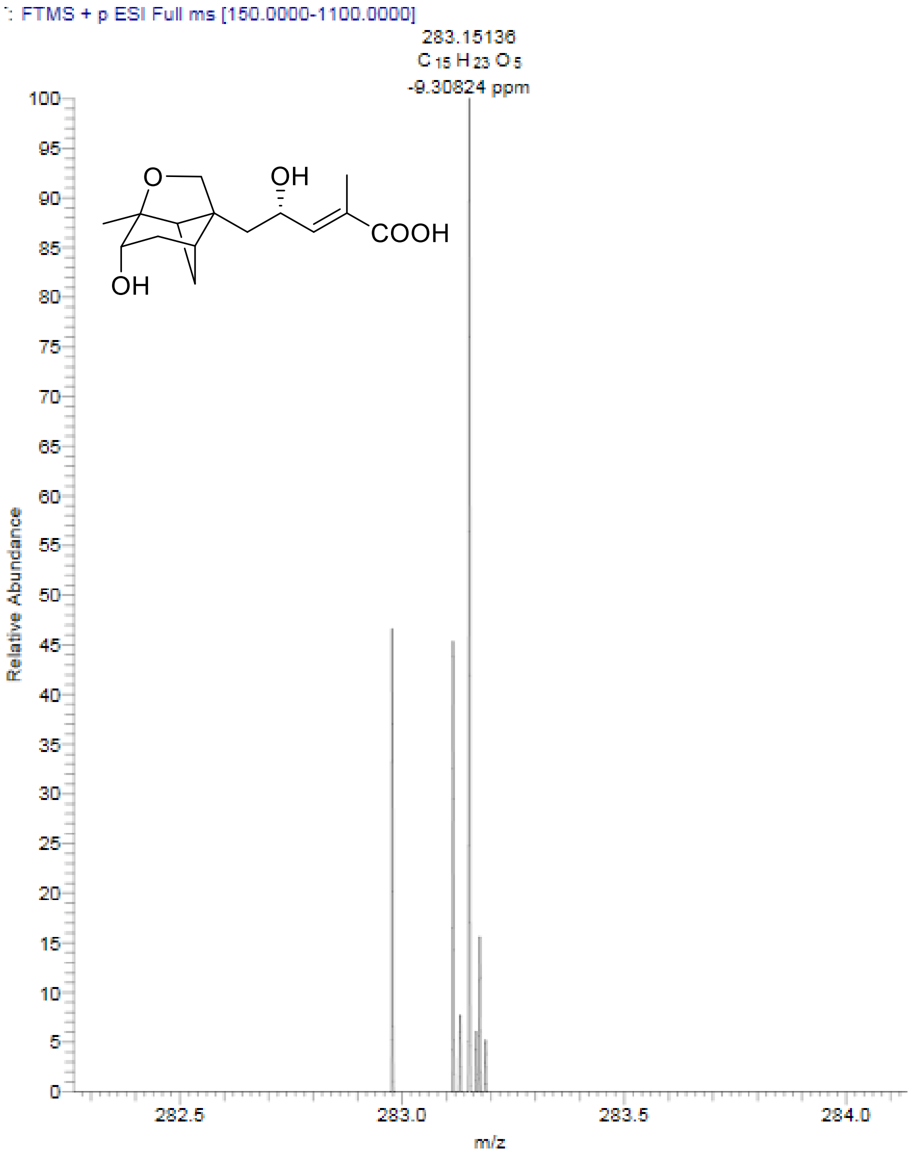

Section S19. NMR and MS spectra for 12

Figure S101. ^{1}H NMR of compound **12** in CD_3OD

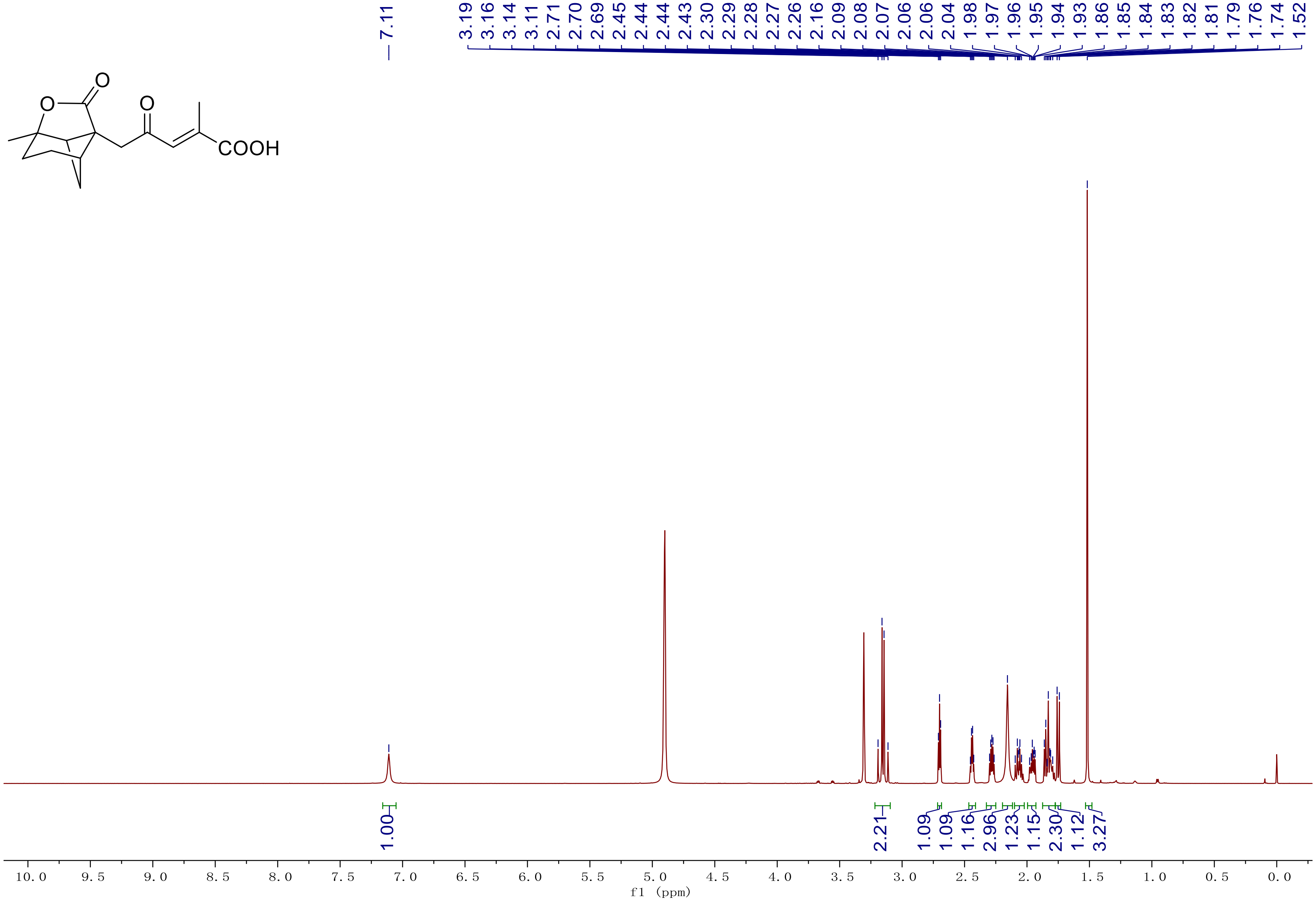

Figure S102. ^{13}C NMR and DEPT of compound **12** in CD_3OD

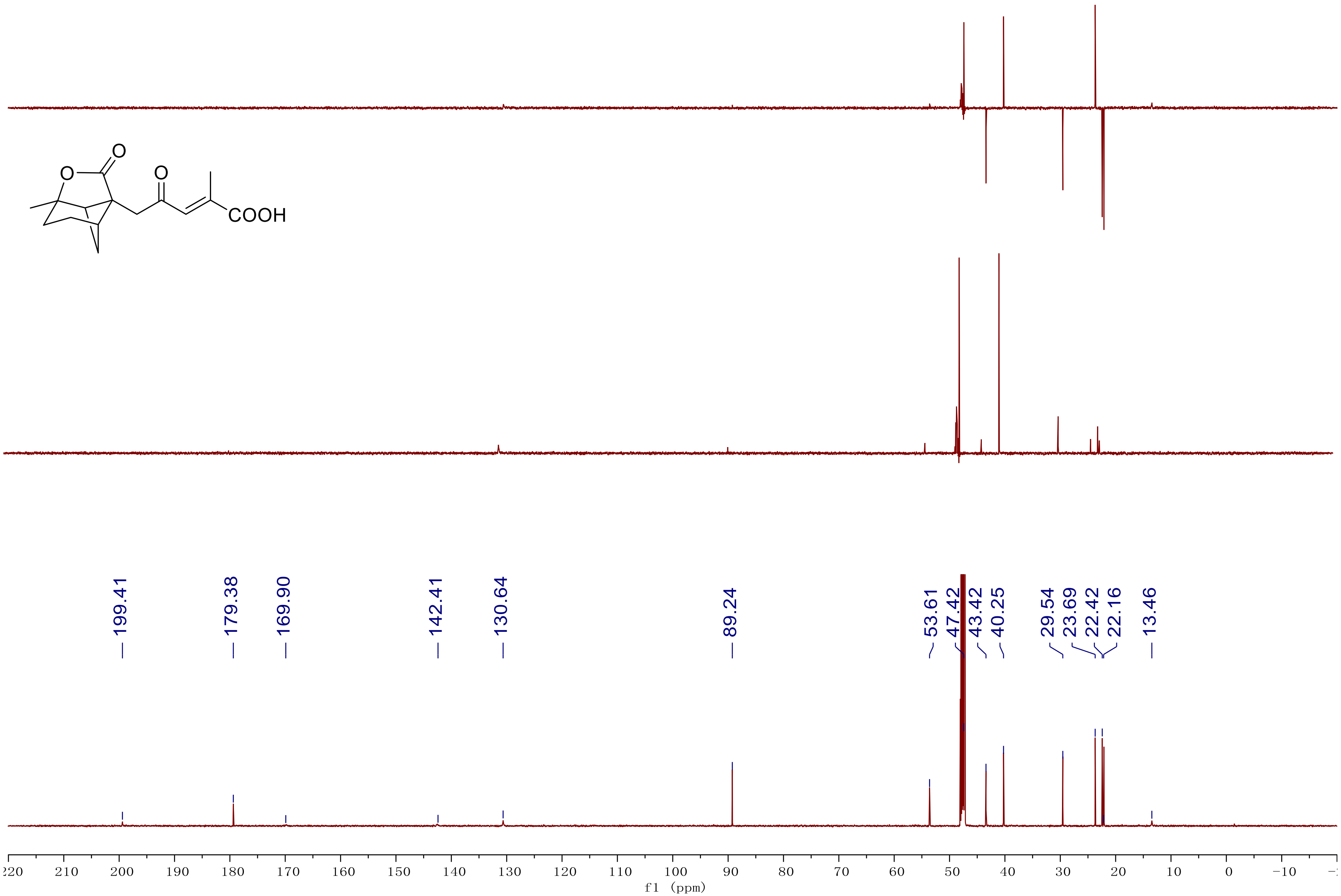

Figure S103. HSQC of compound **12** in CD_3OD

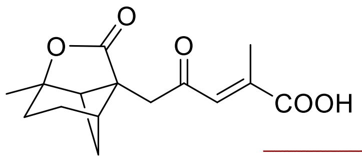

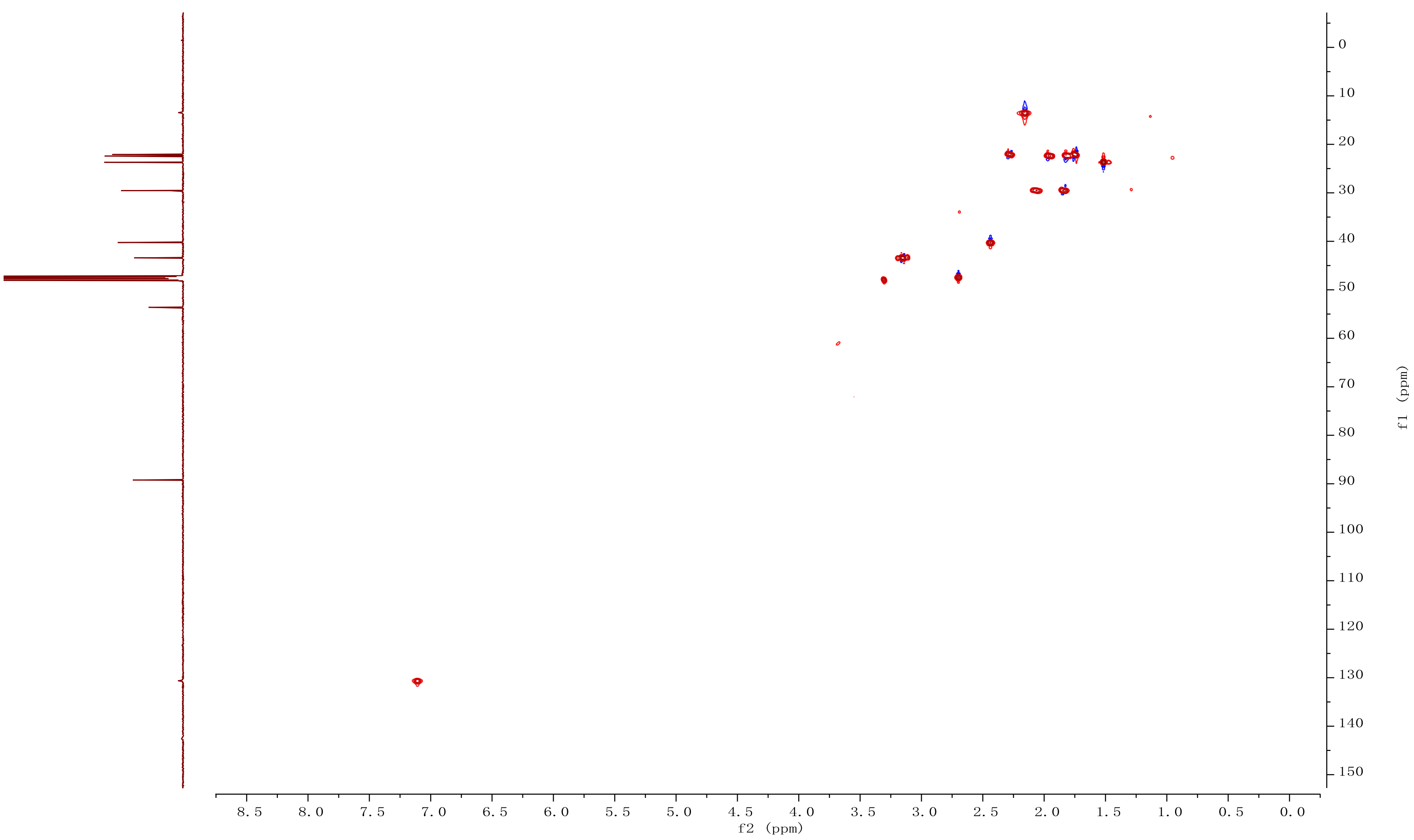

Figure S104. HMBC of compound **12** in CD_3OD

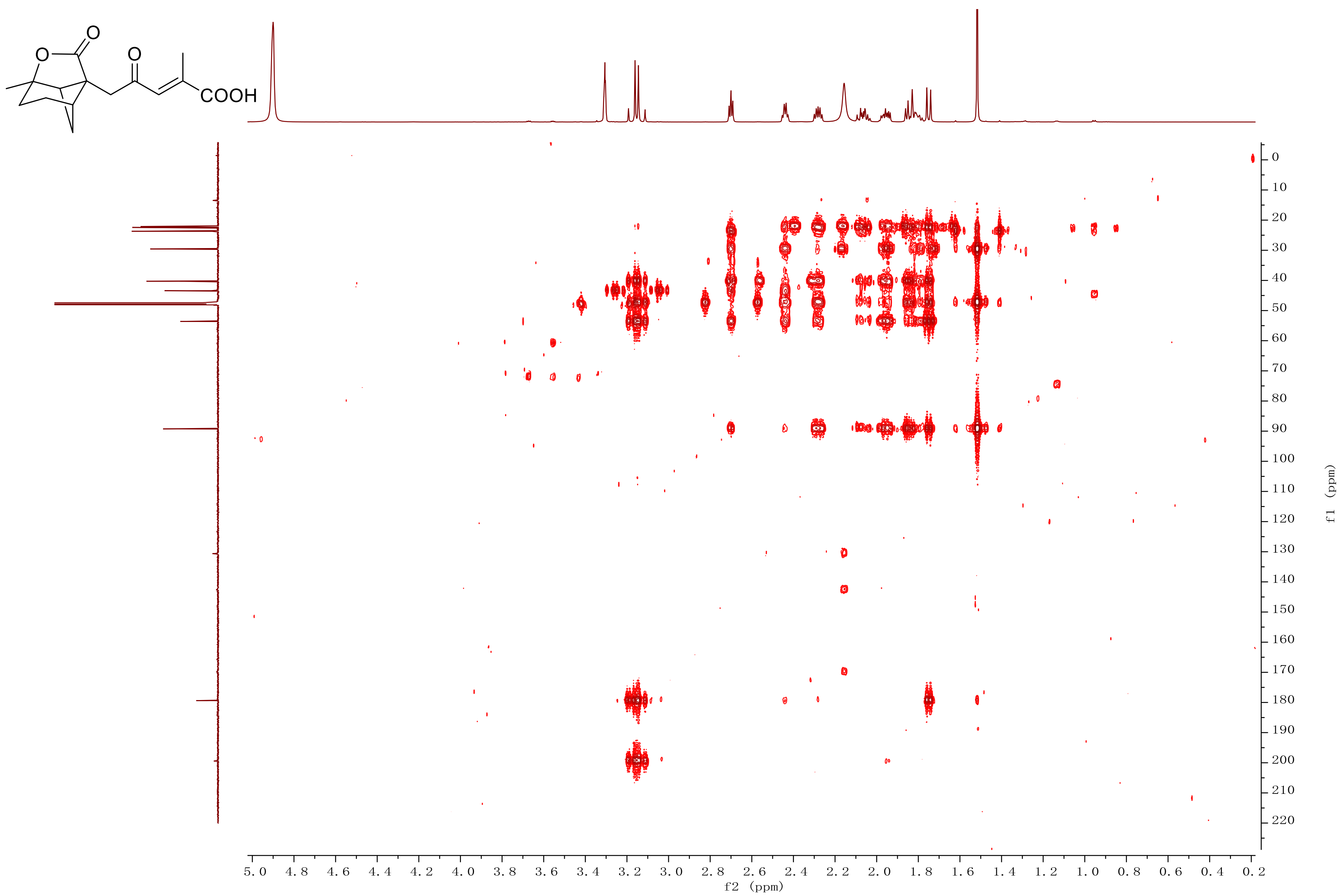

Figure S105. ^{1}H-^{1}H COSY of compound **12** in CD_3OD

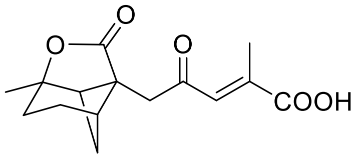

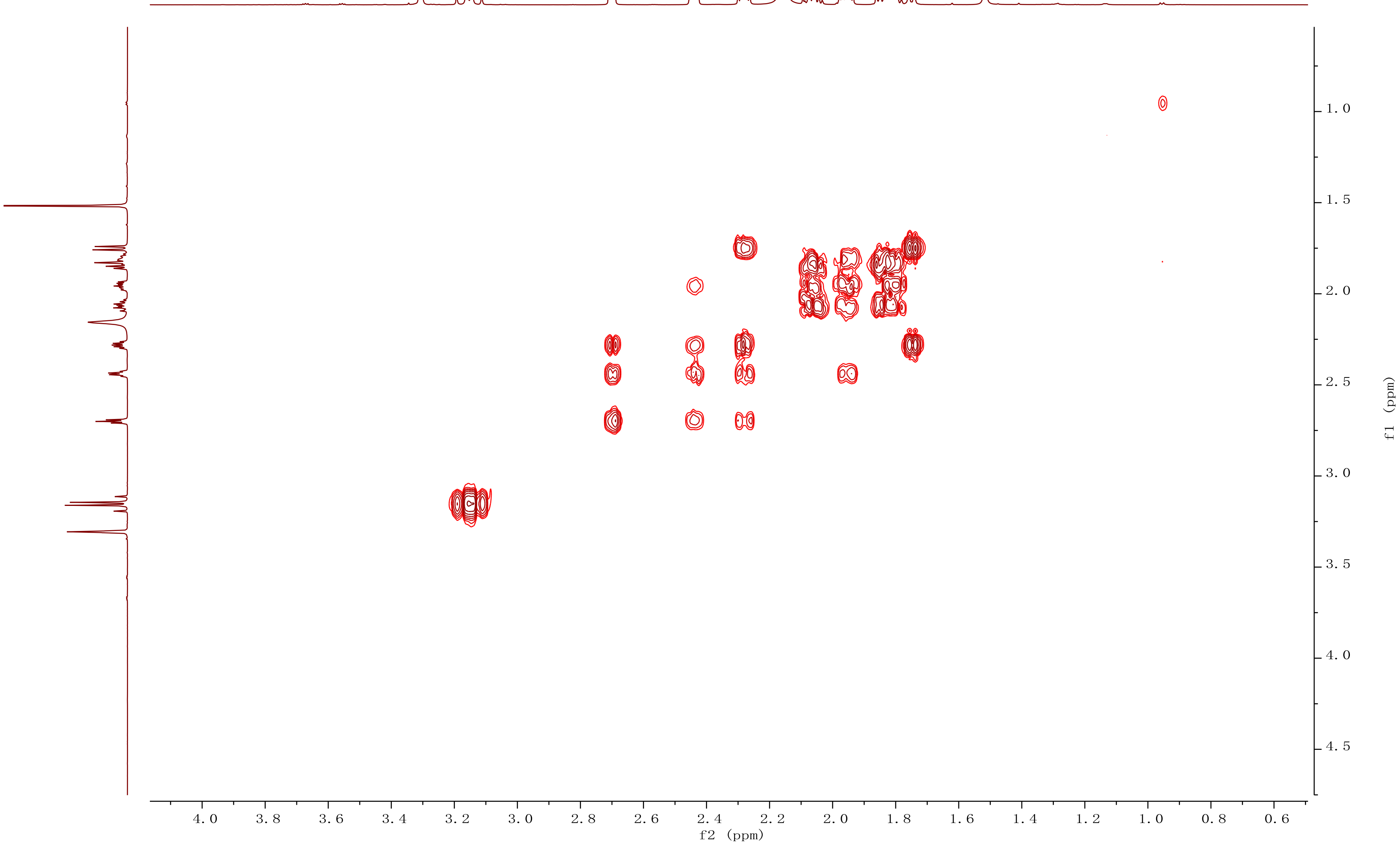

Figure S106. ROESY of compound **12** in CD_3OD

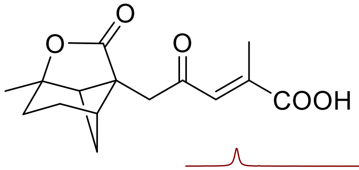

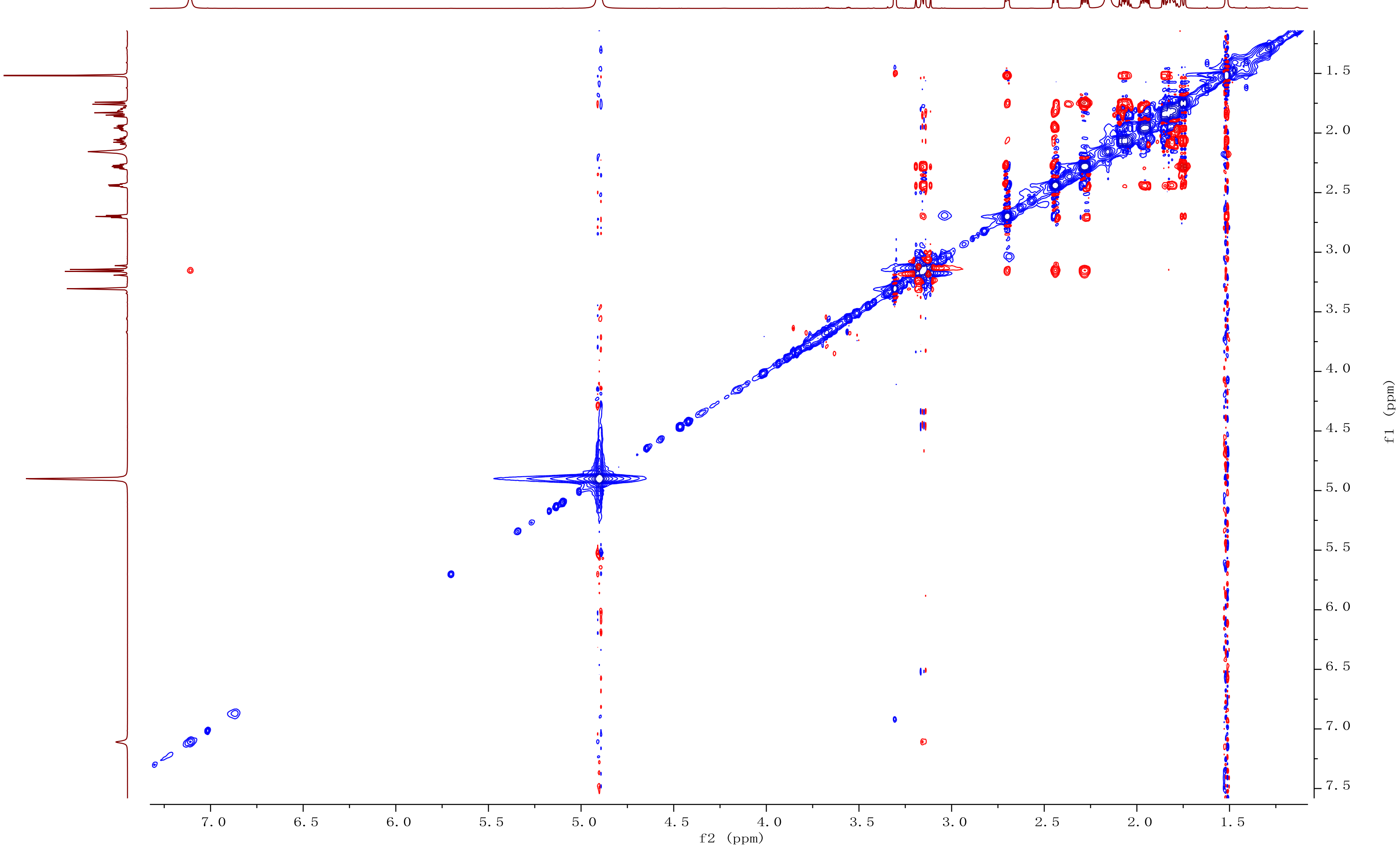

Figure S107. HR-ESIMS of compound **12**

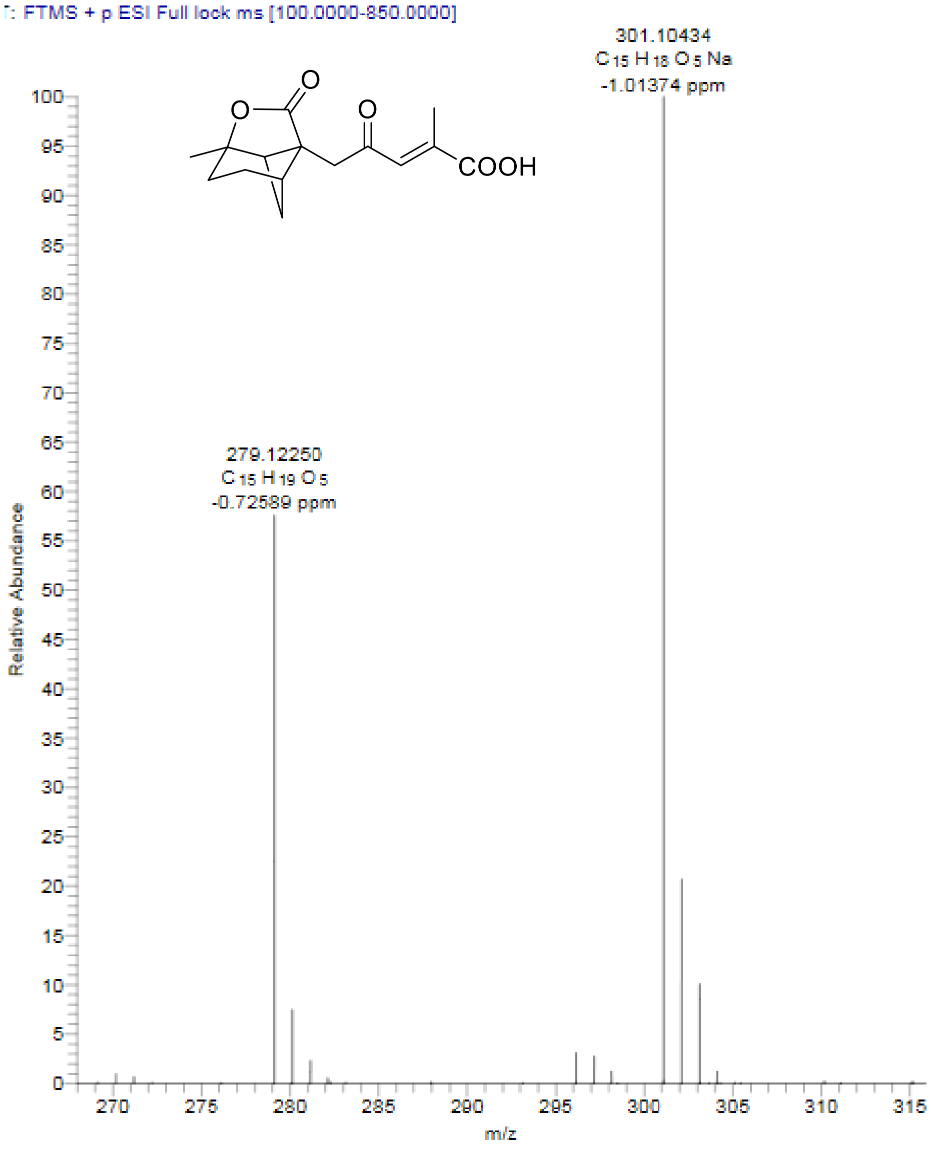

Section S20. NMR and MS spectra for 13

Figure S108. ^{1}H NMR of compound **13** in CD_3OD

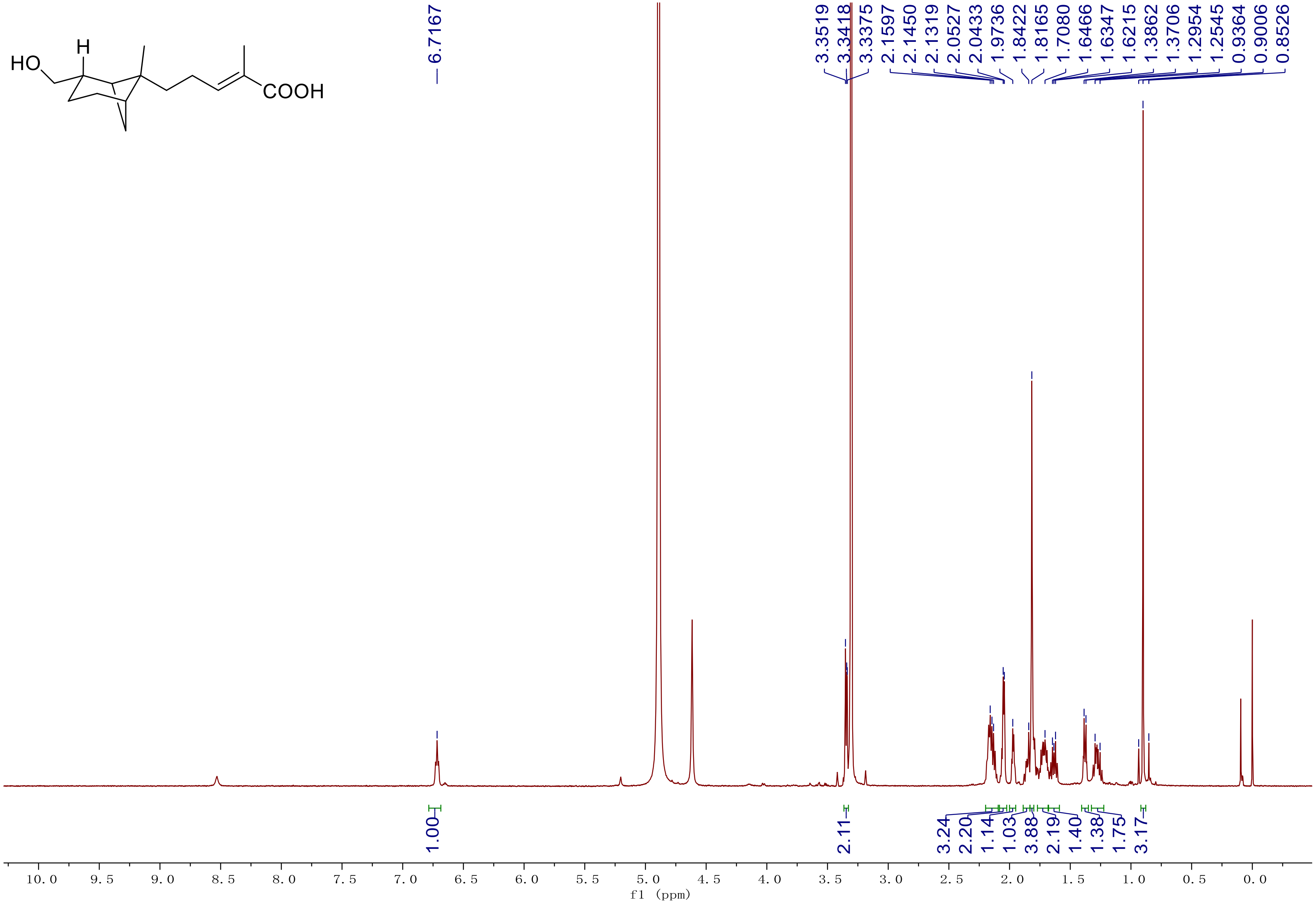

Figure S109. ^{13}C NMR and DEPT of compound **13** in CD_3OD

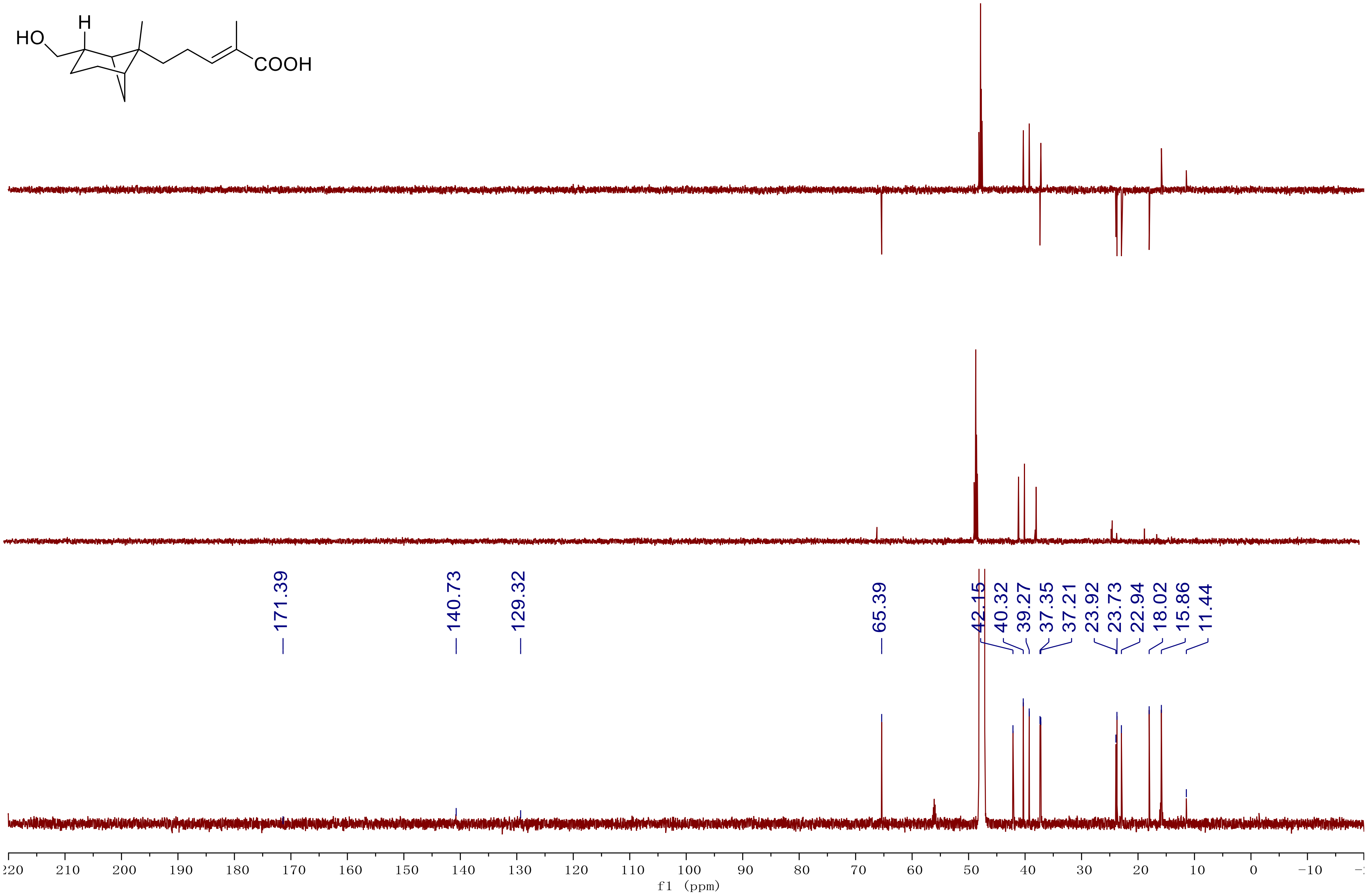

Figure S110. HSQC of compound **13** in CD_3OD

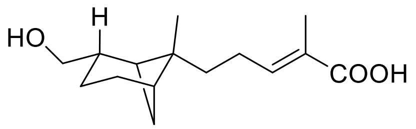

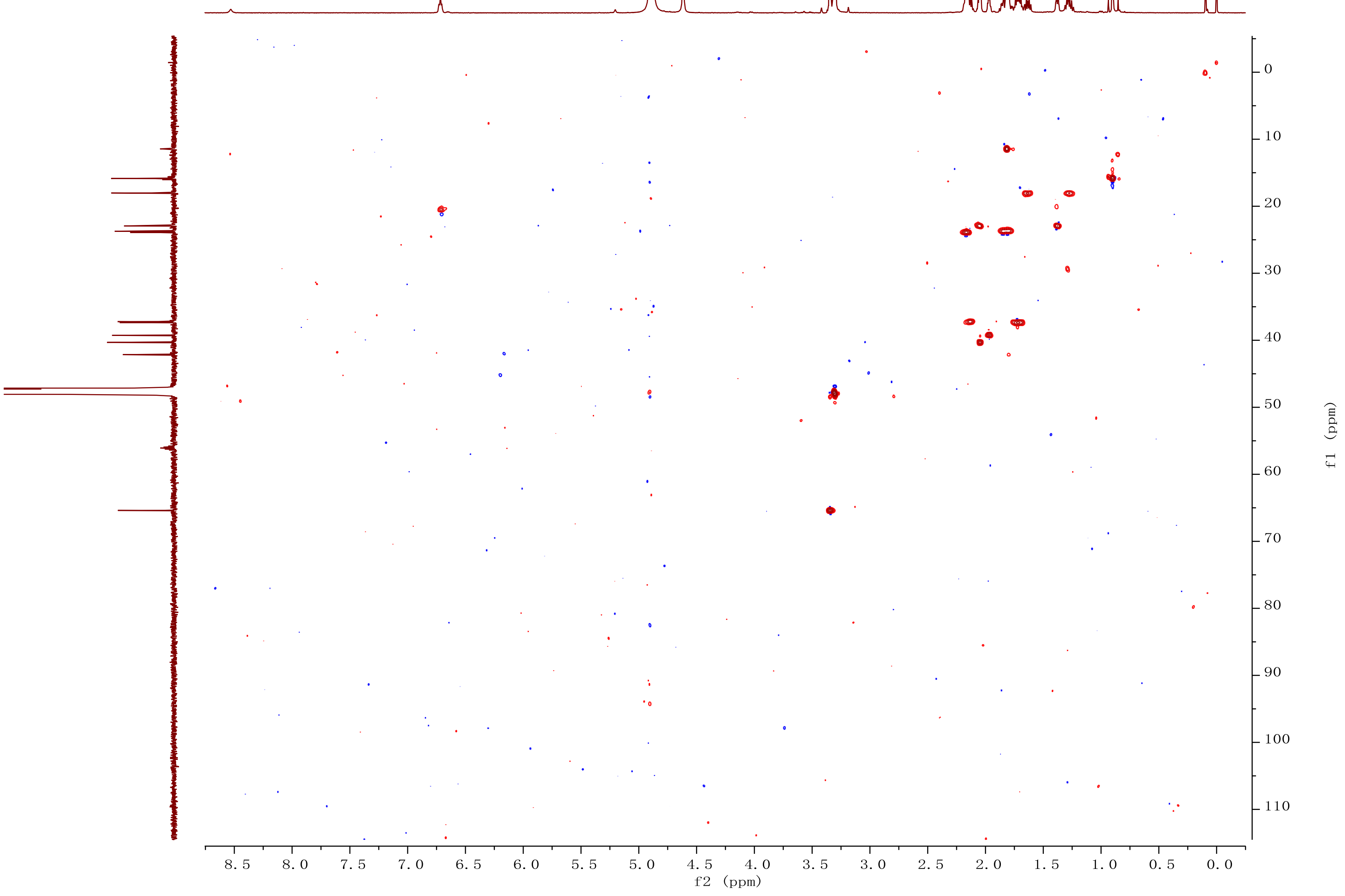

Figure S111. HMBC of compound **13** in CD_3OD

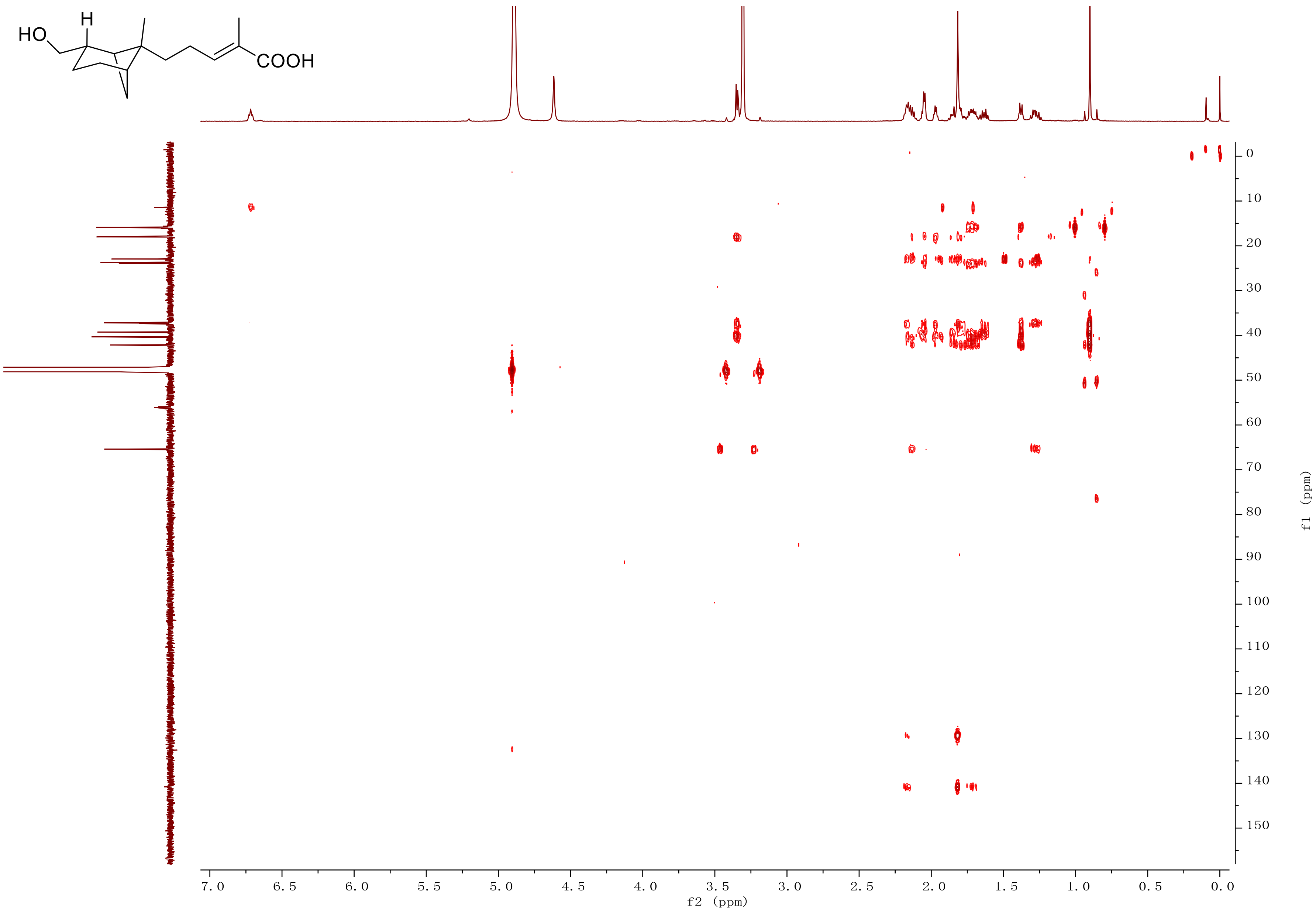

Figure S112. ^{1}H-^{1}H COSY of compound **13** in CD_3OD

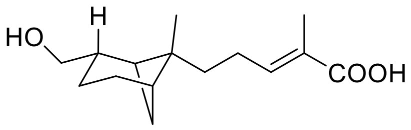

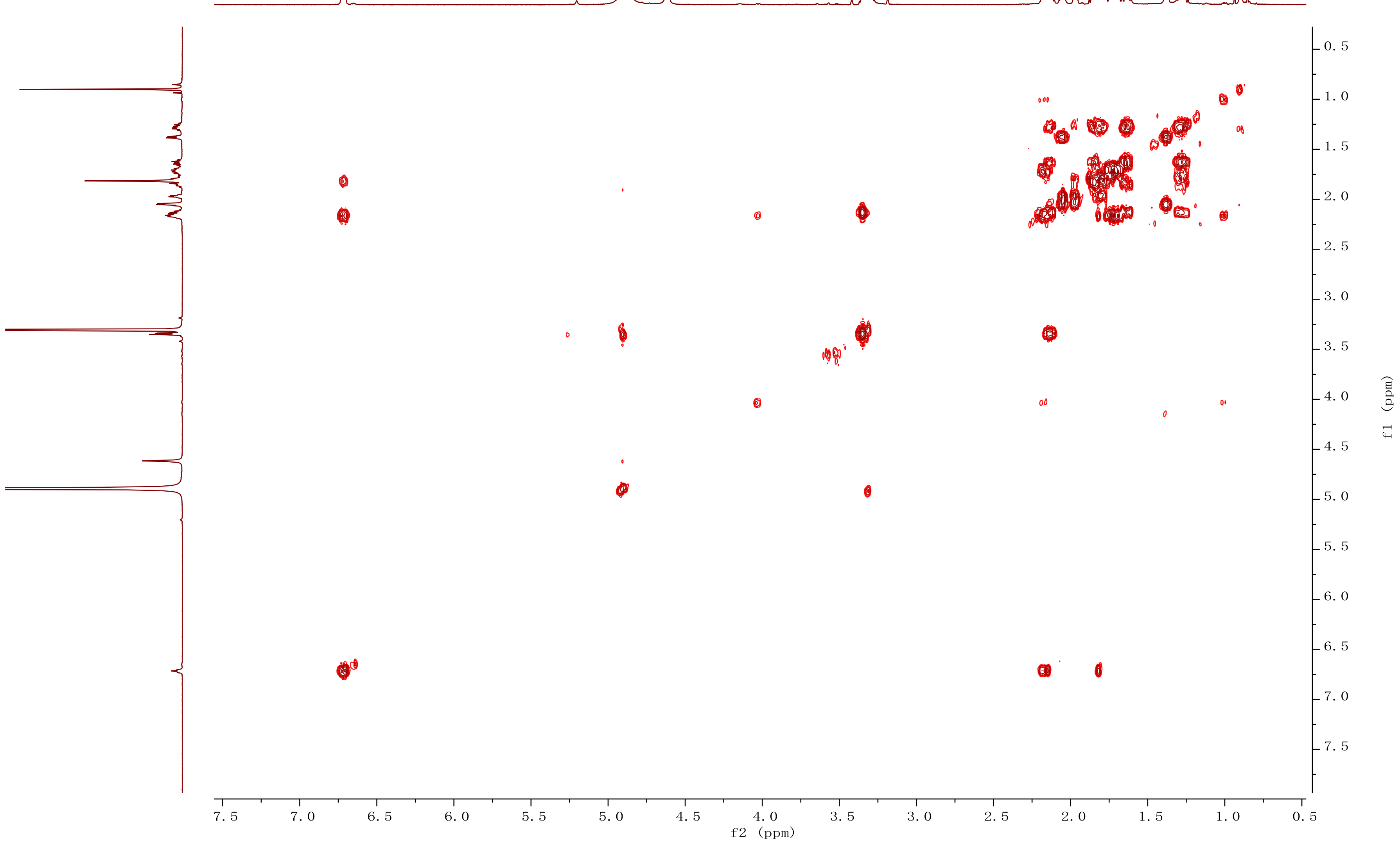

Figure S113. ROESY of compound **13** in CD_3OD

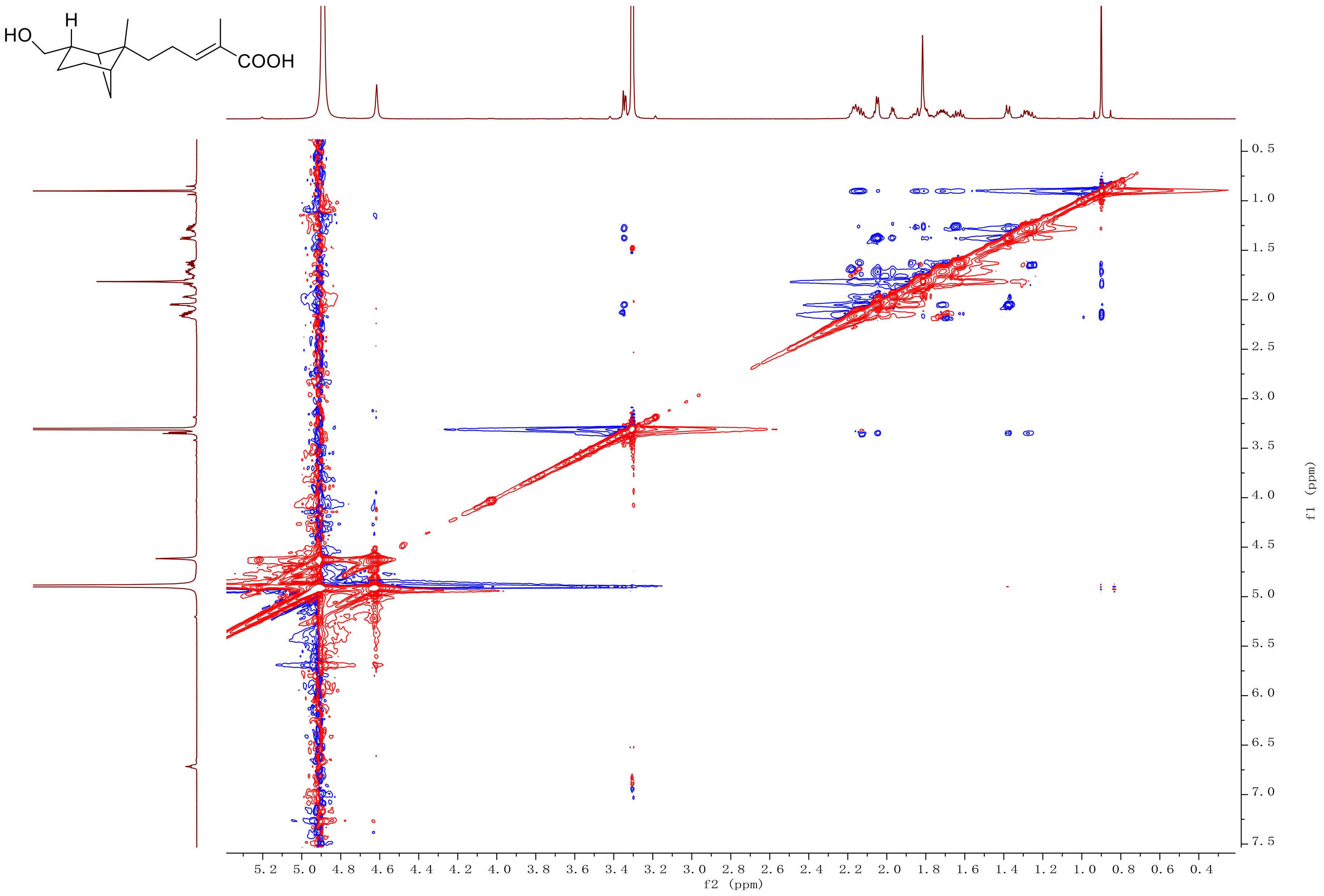

Figure S114. HR-ESIMS of compound **13**

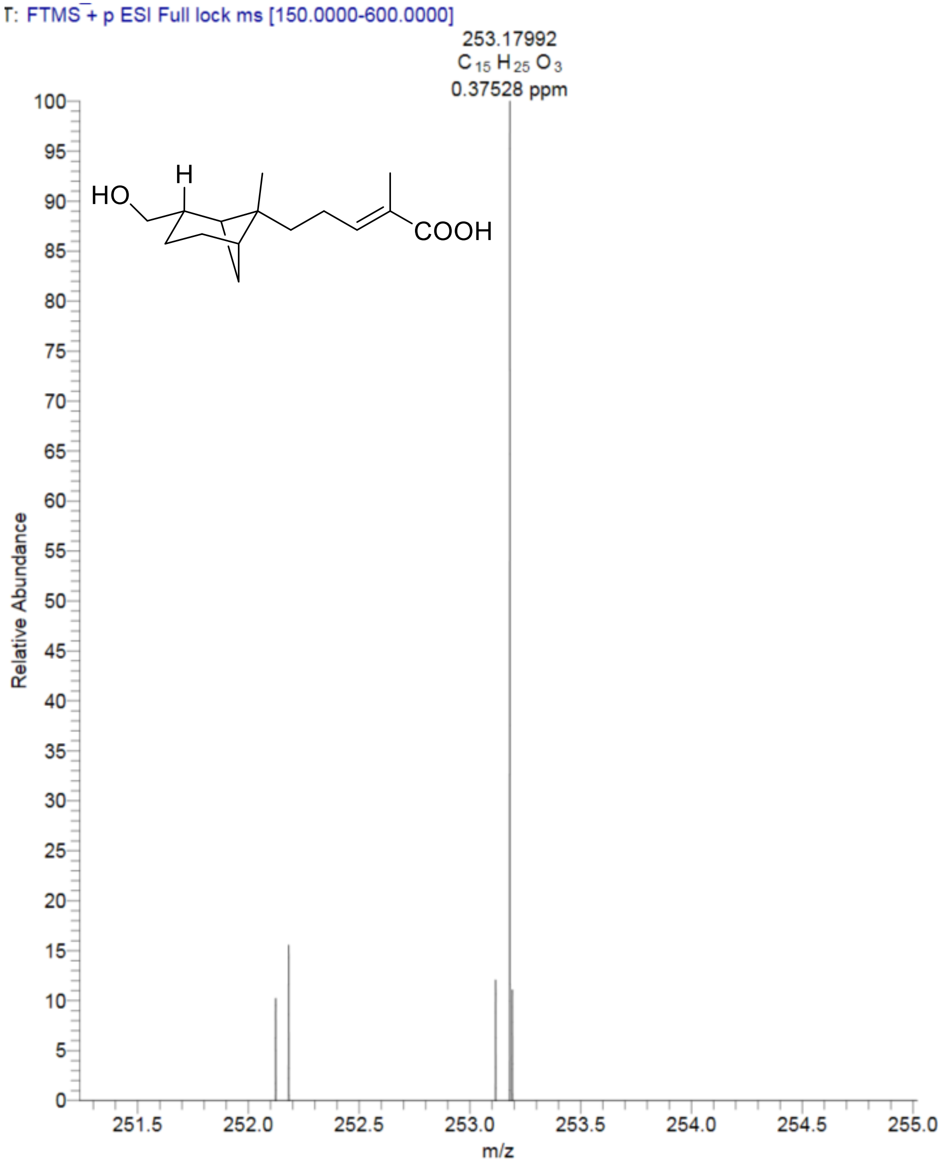

Section S21. NMR and MS spectra for 14

Figure S115. ^{1}H NMR of compound **14** in CD_3OD

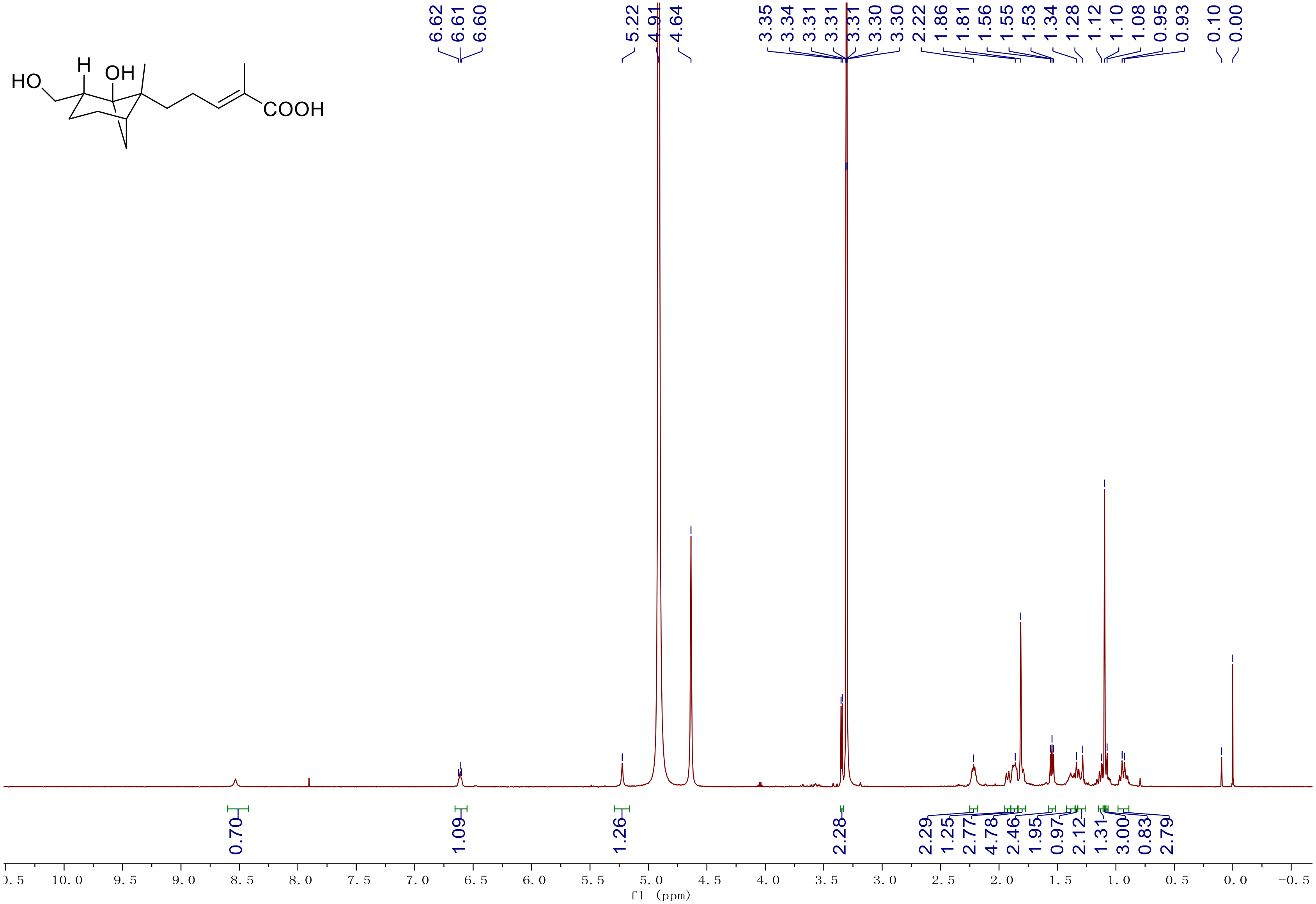

Figure S116. ^{13}C NMR and DEPT of compound **14** in CD_3OD

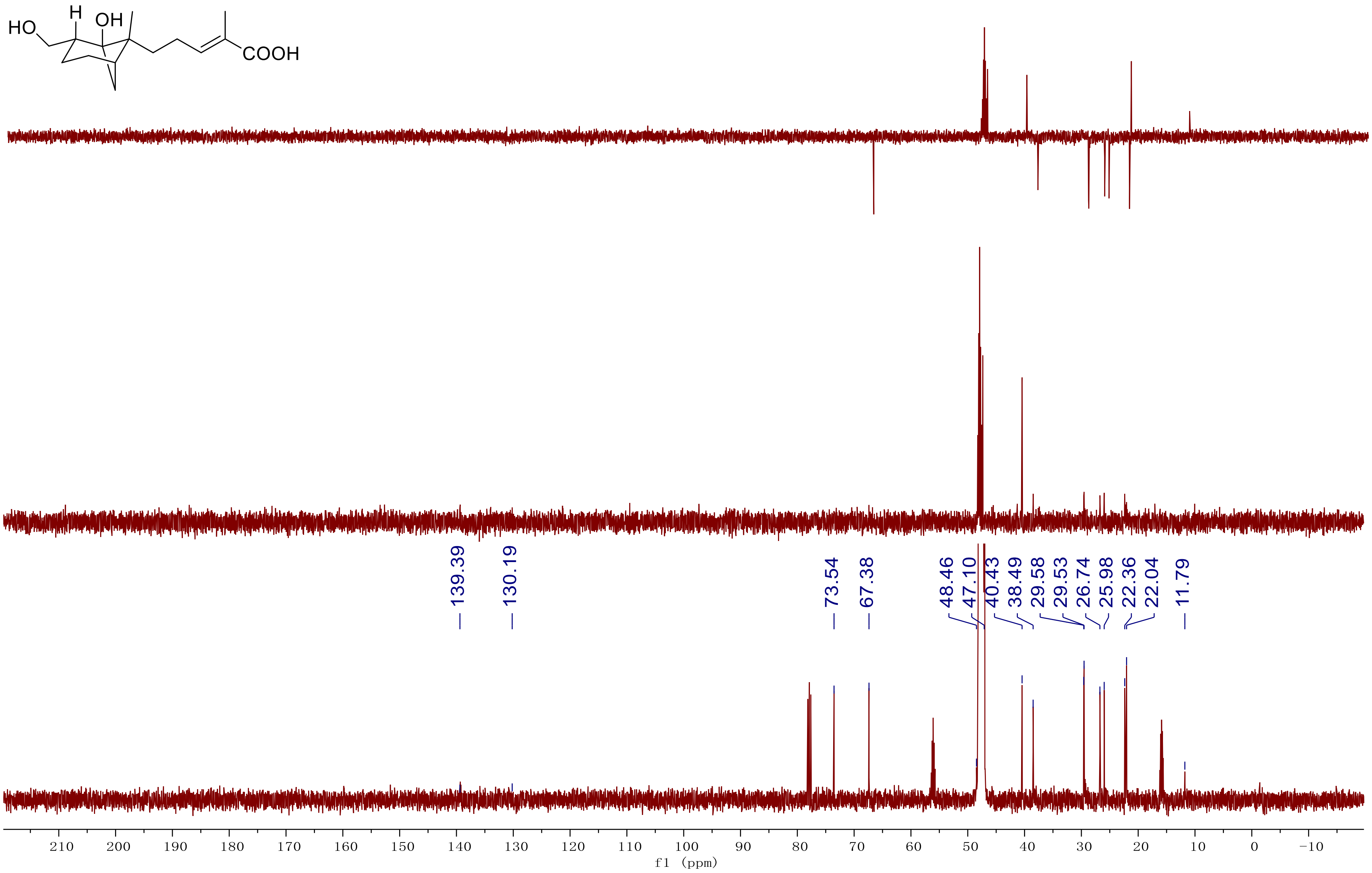

Figure S117. HSQC of compound **14** in CD_3OD

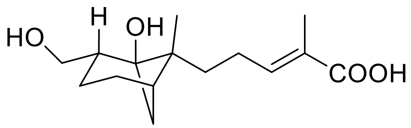

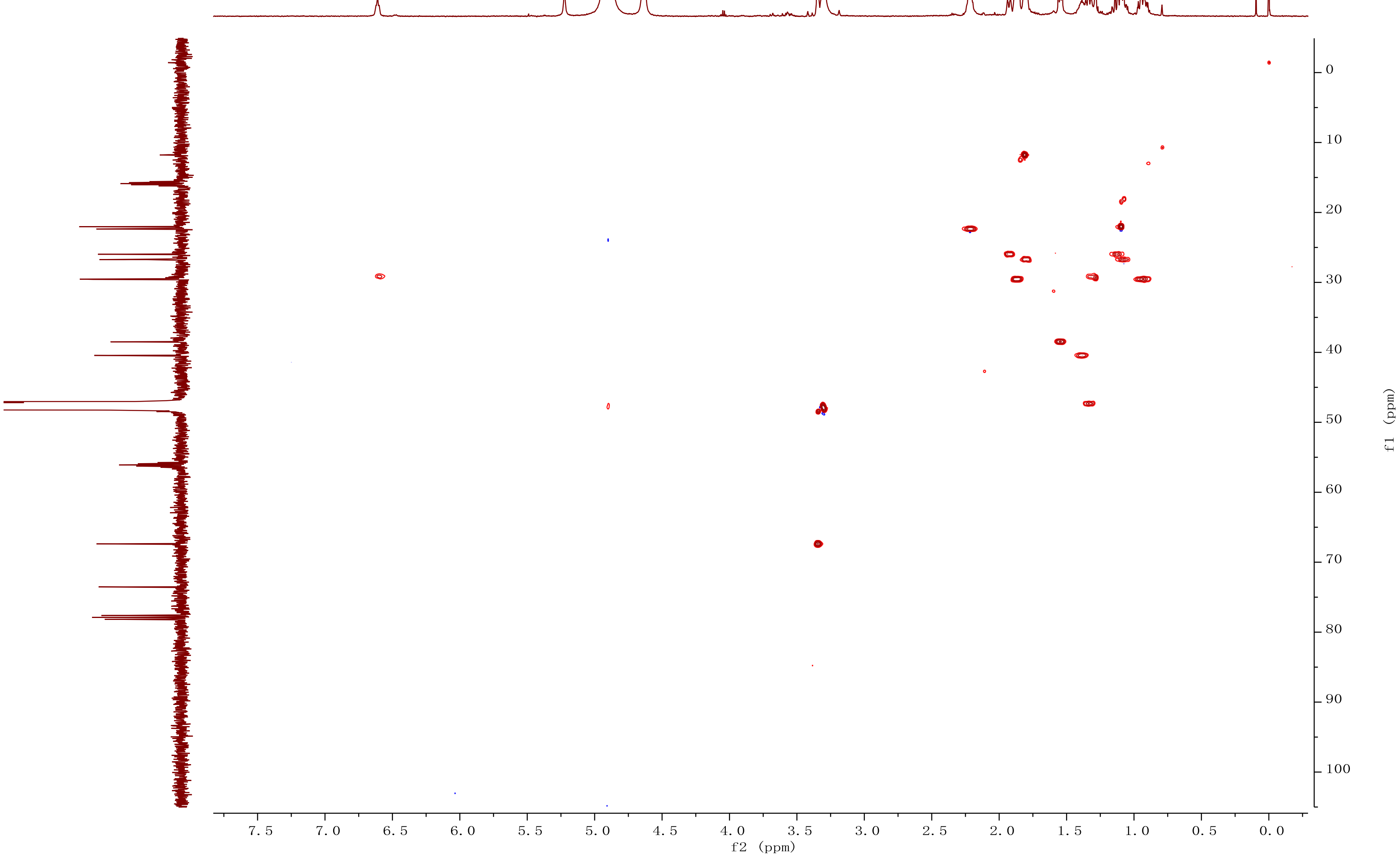

Figure S118. HMBC of compound **14** in CD_3OD

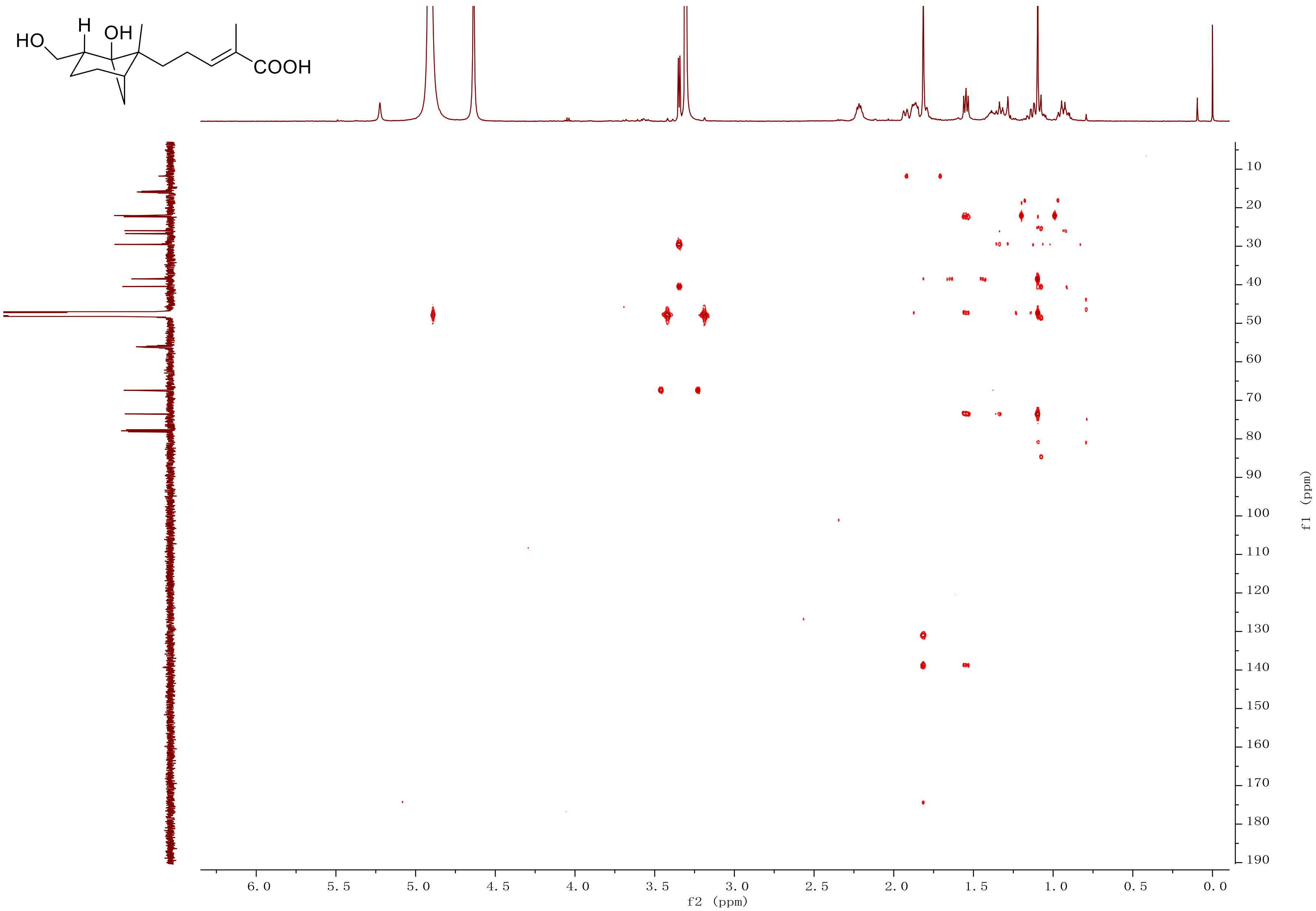

Figure S119. ^{1}H-^{1}H COSY of compound **14** in CD_3OD

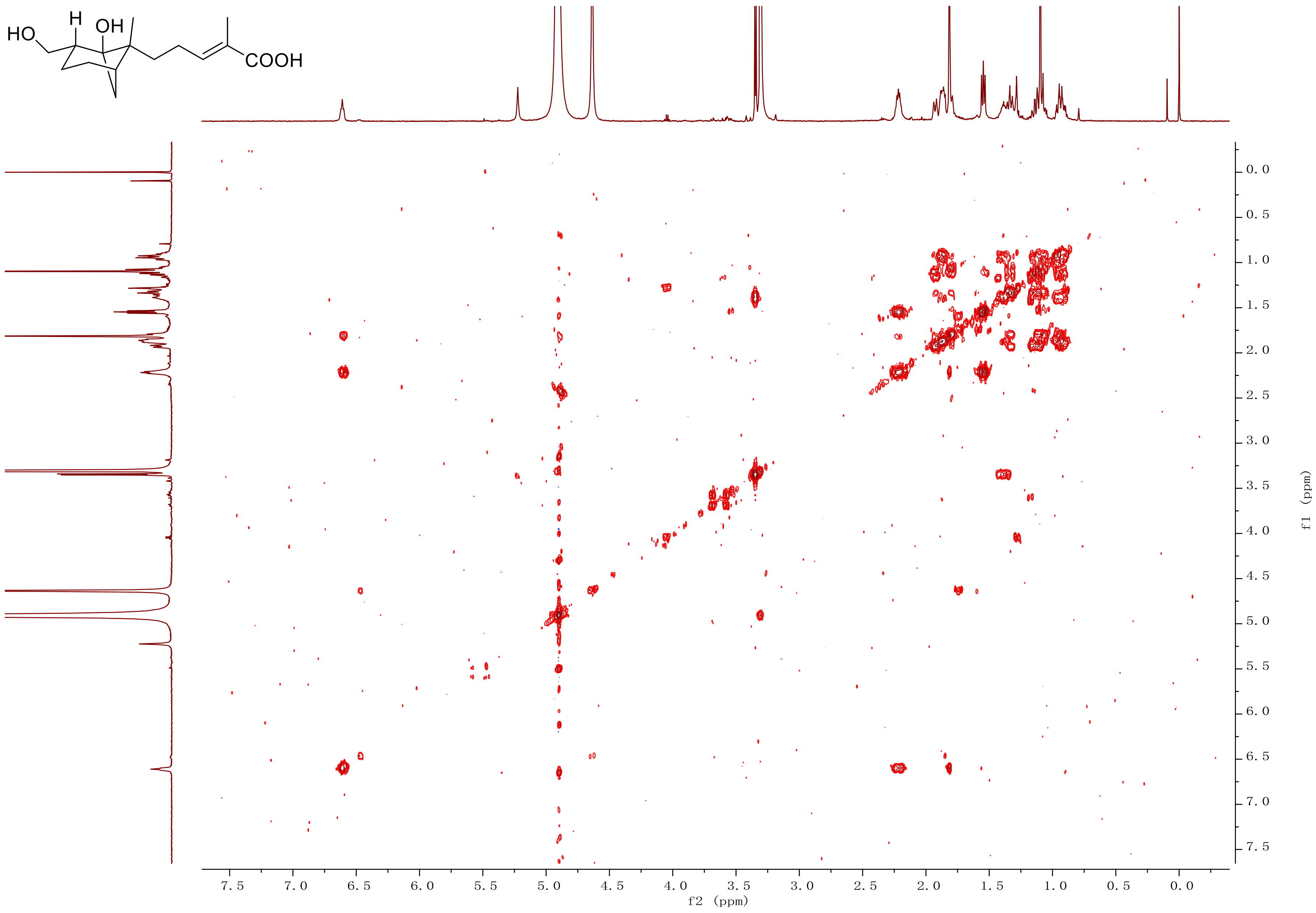

Figure S120. ROESY of compound **14** in CD_3OD

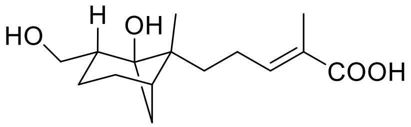

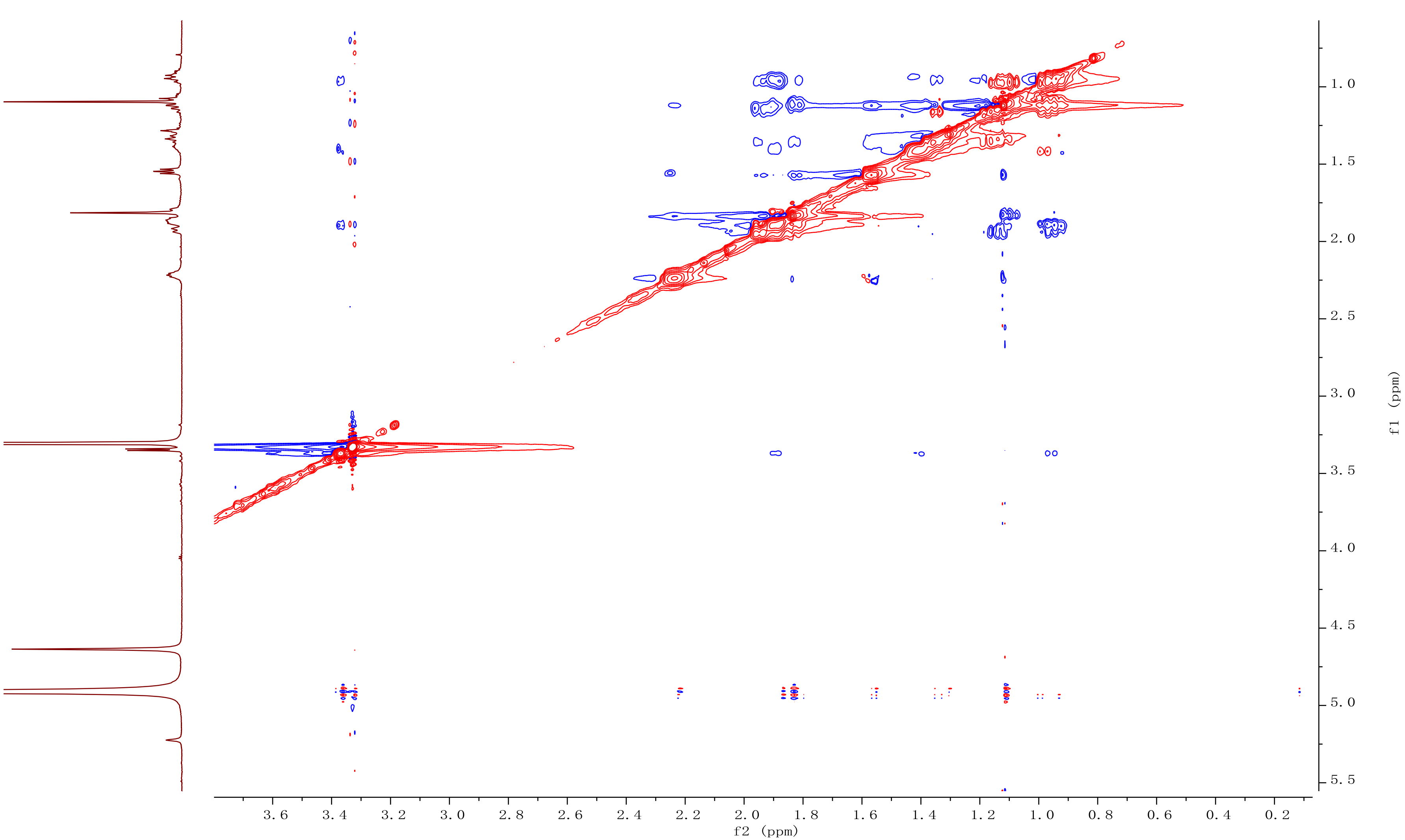

Figure S121. HR-ESIMS of compound **14**

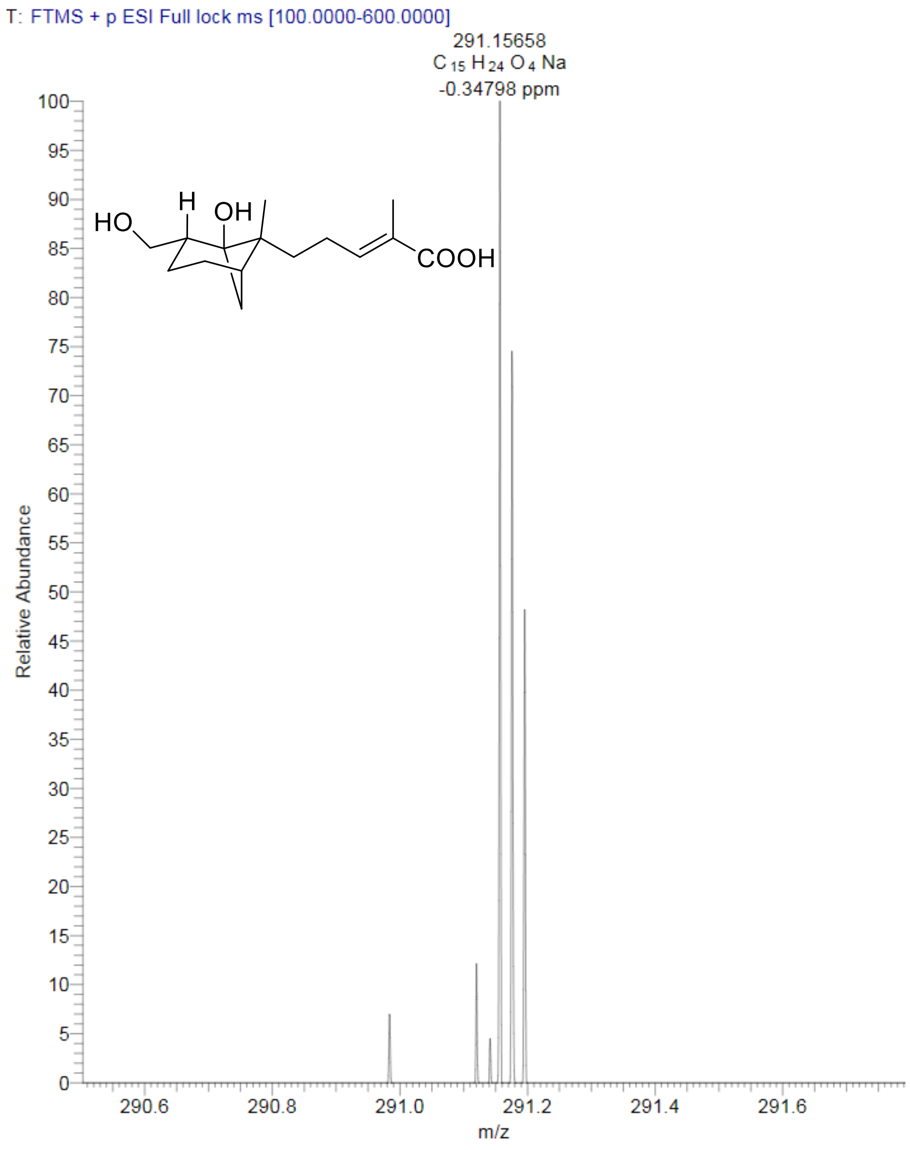

Section S22. NMR and MS spectra for 15

Figure S122. ^{1}H NMR of compound **15** in CD_3OD

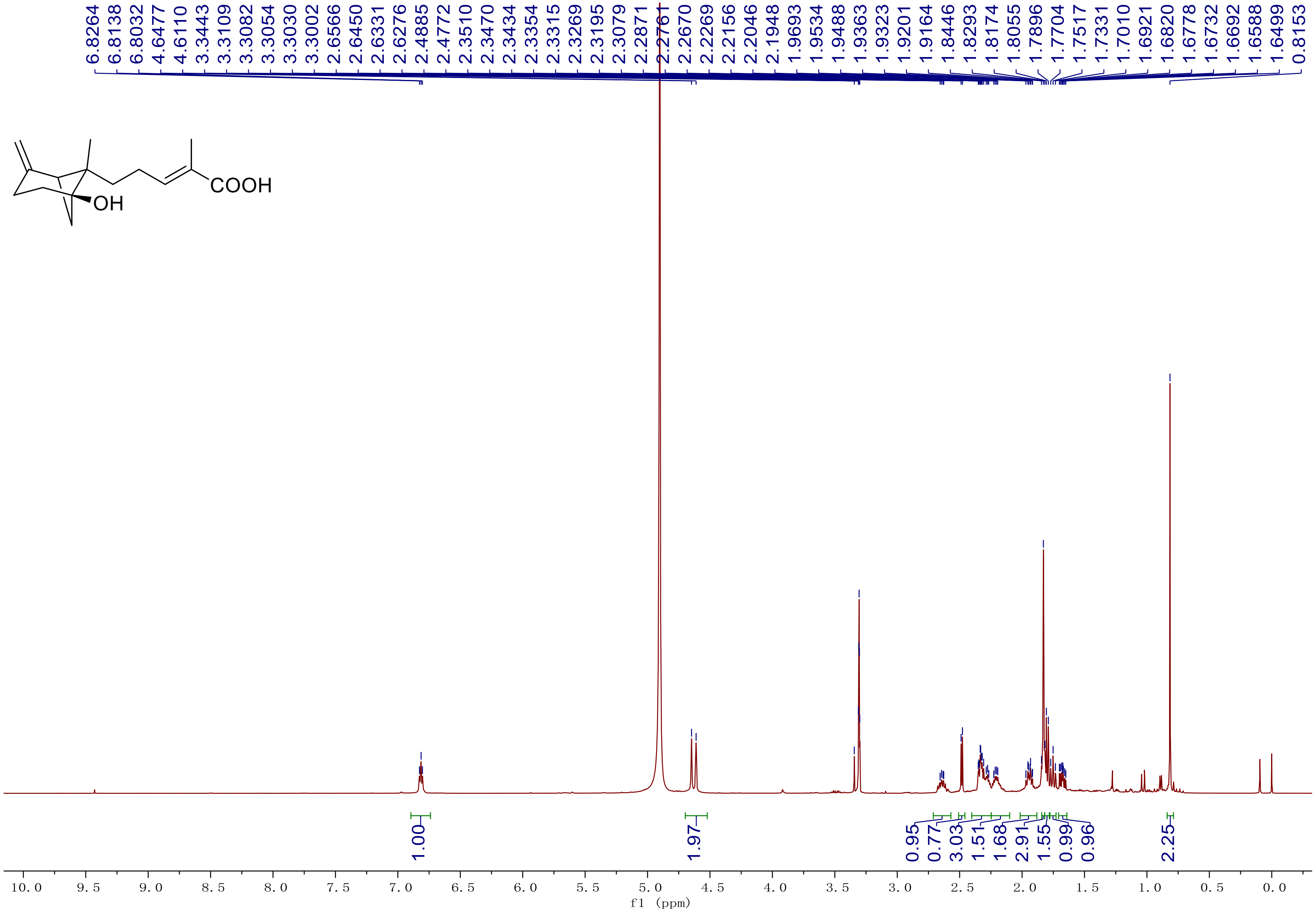

Figure S123. ^{13}C NMR and DEPT of compound **15** in CD_3OD

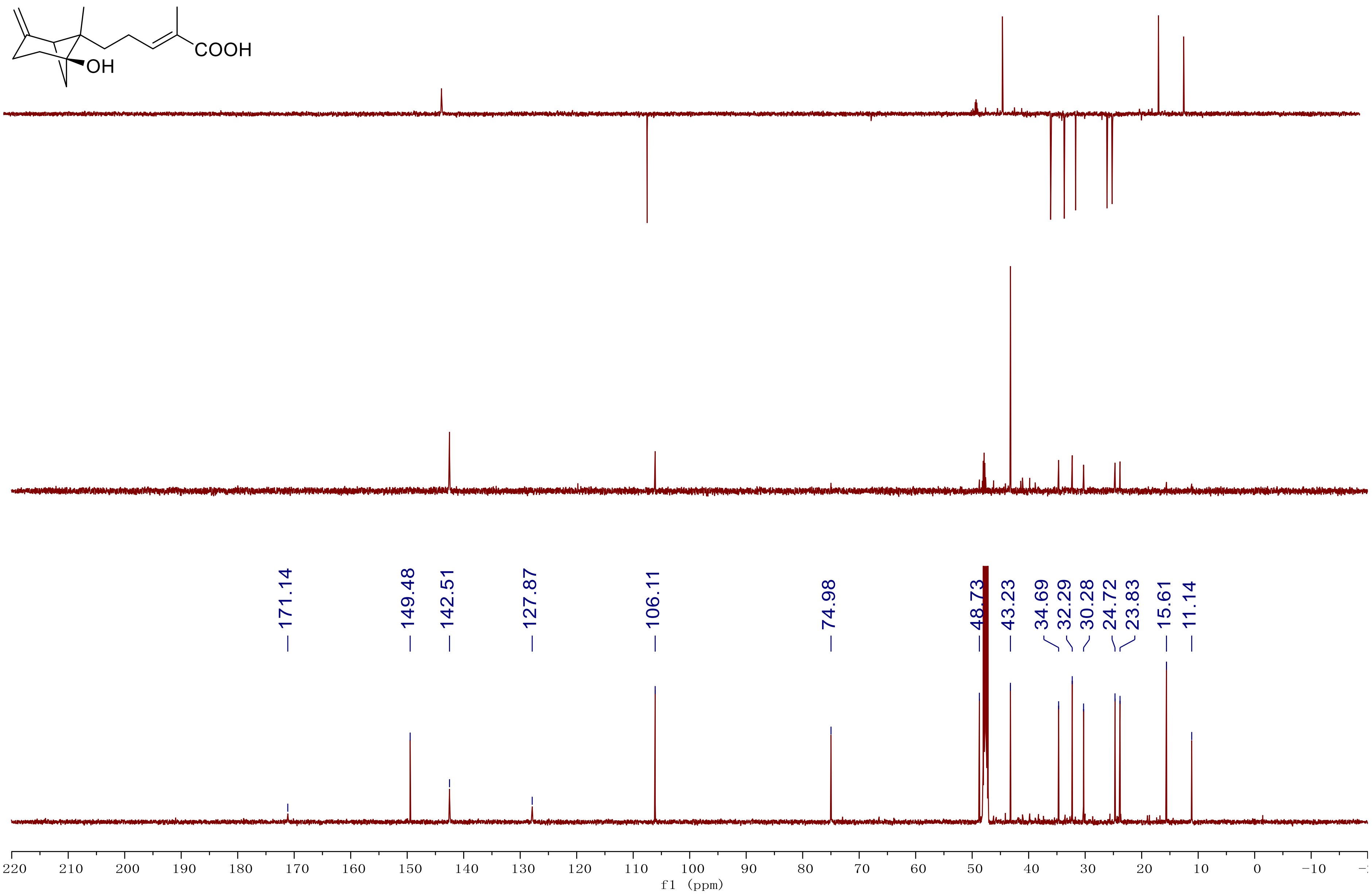

Figure S124. HSQC of compound **15** in CD_3OD

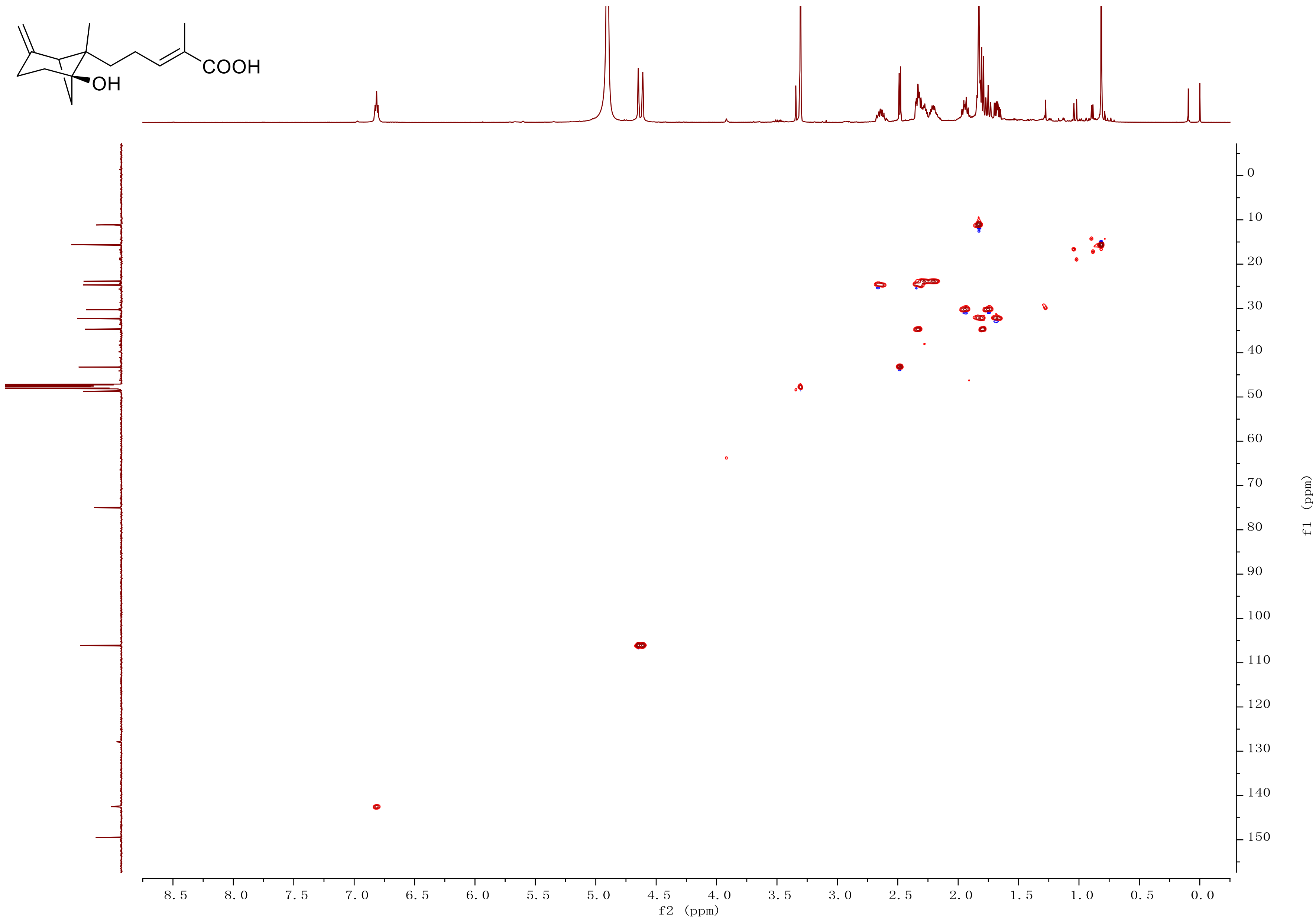

Figure S125. HMBC of compound **15** in CD_3OD

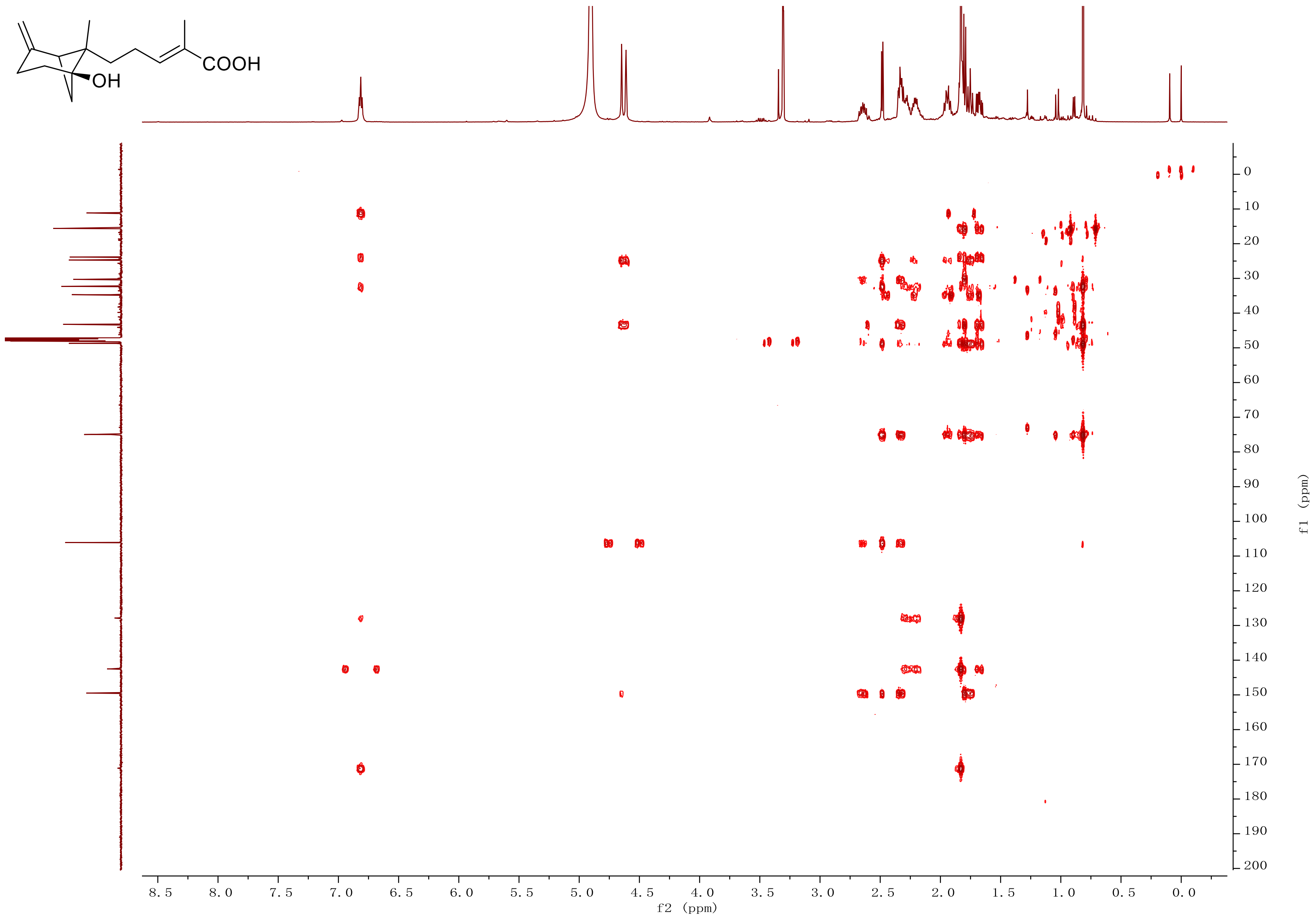

Figure S126. ${}^{1}H$-${}^{1}H$ COSY of compound **15** in CD_3OD

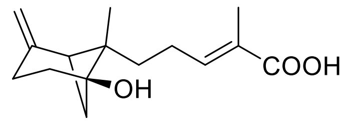

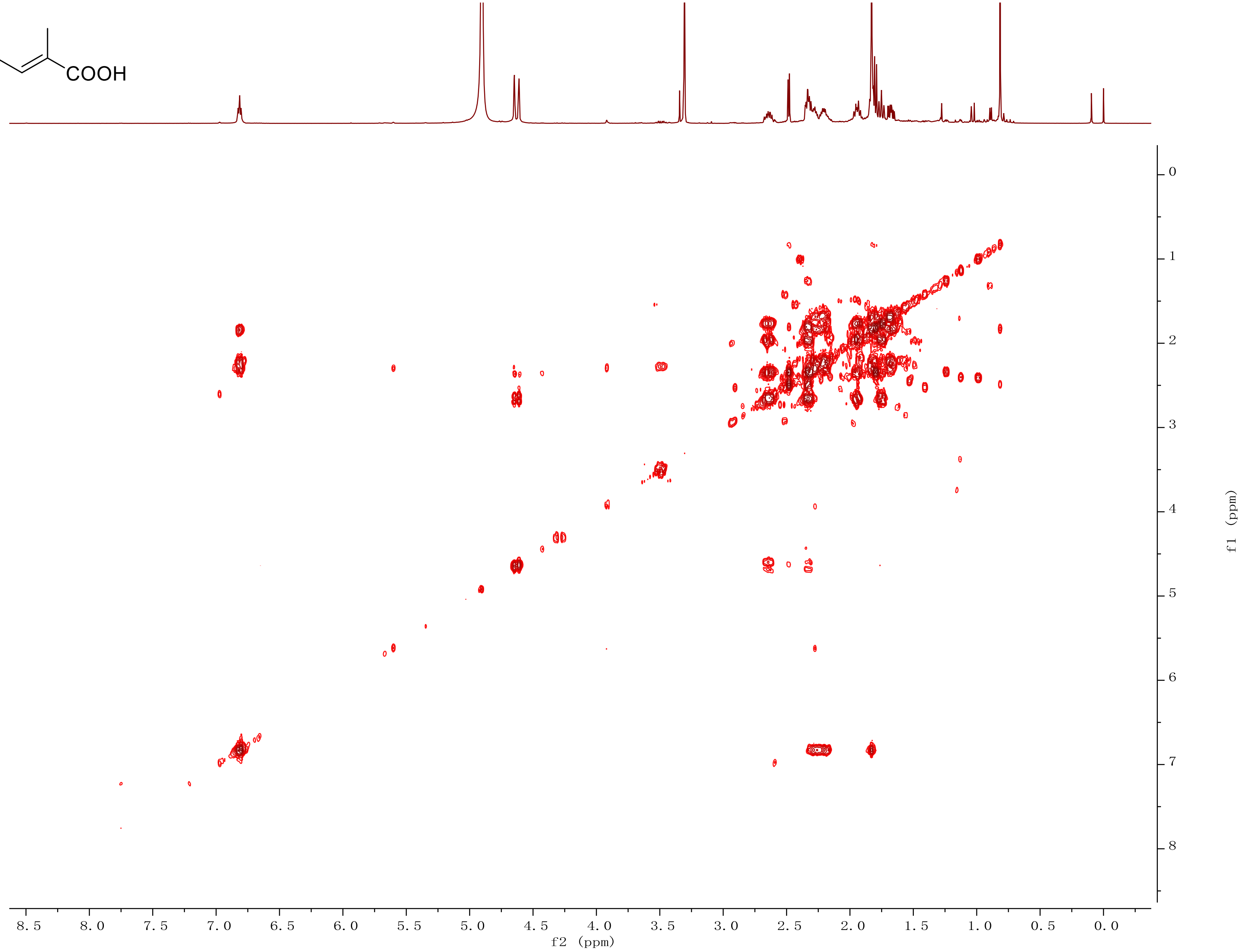

Figure S127. ROESY of compound **15** in CD_3OD

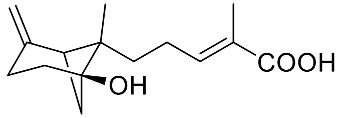

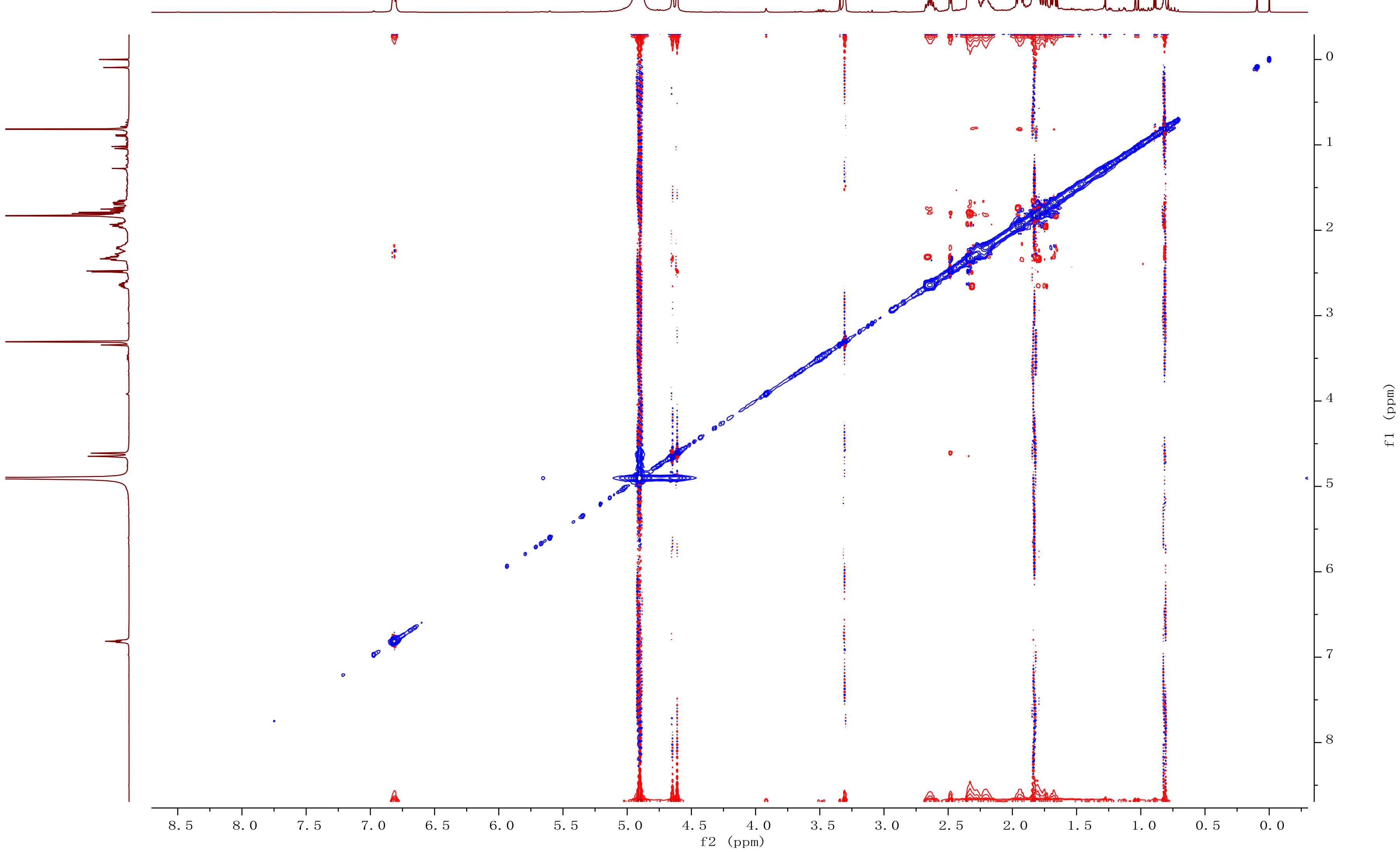

Figure S128. HR-ESIMS of compound **15**

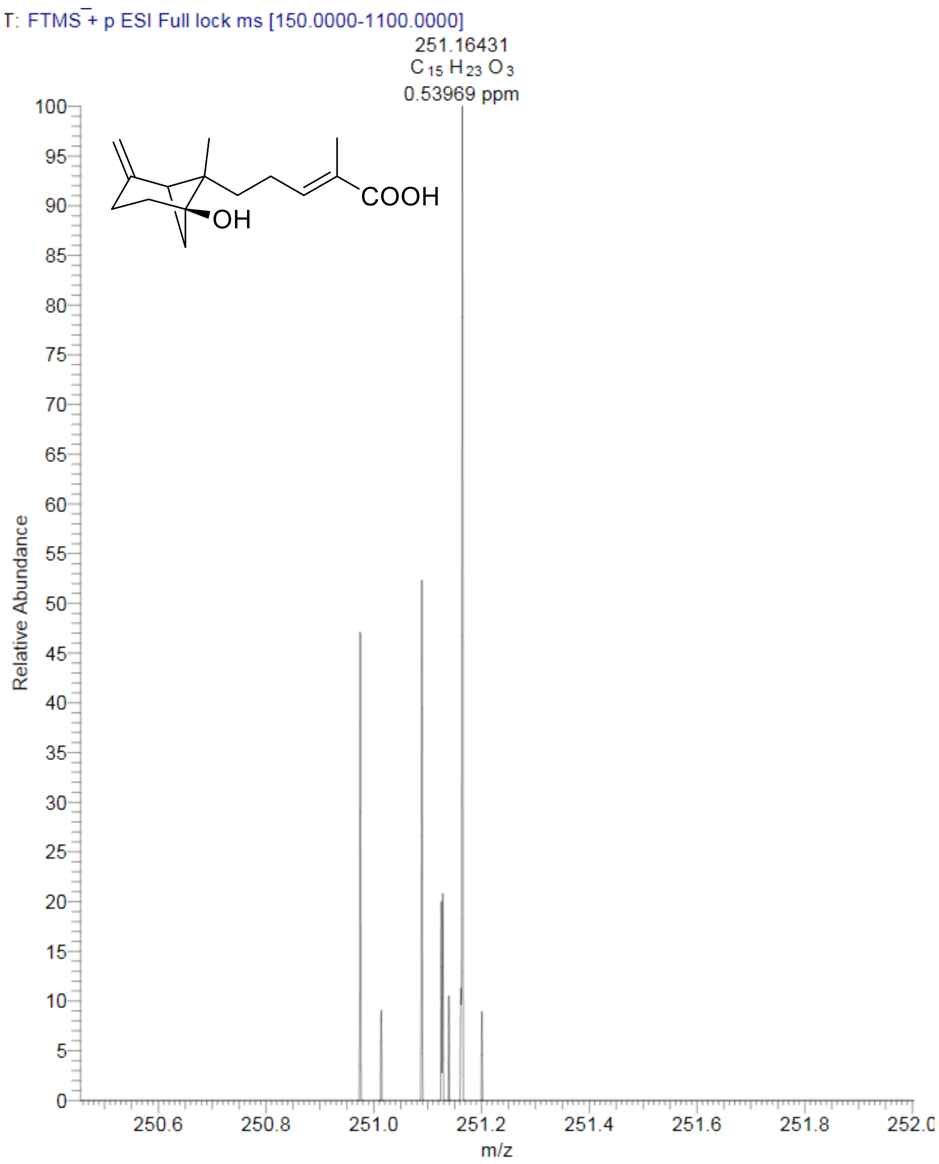

Section S23. NMR and MS spectra for 16

Figure S129. ^{1}H NMR of compound **16** in CD_3OD

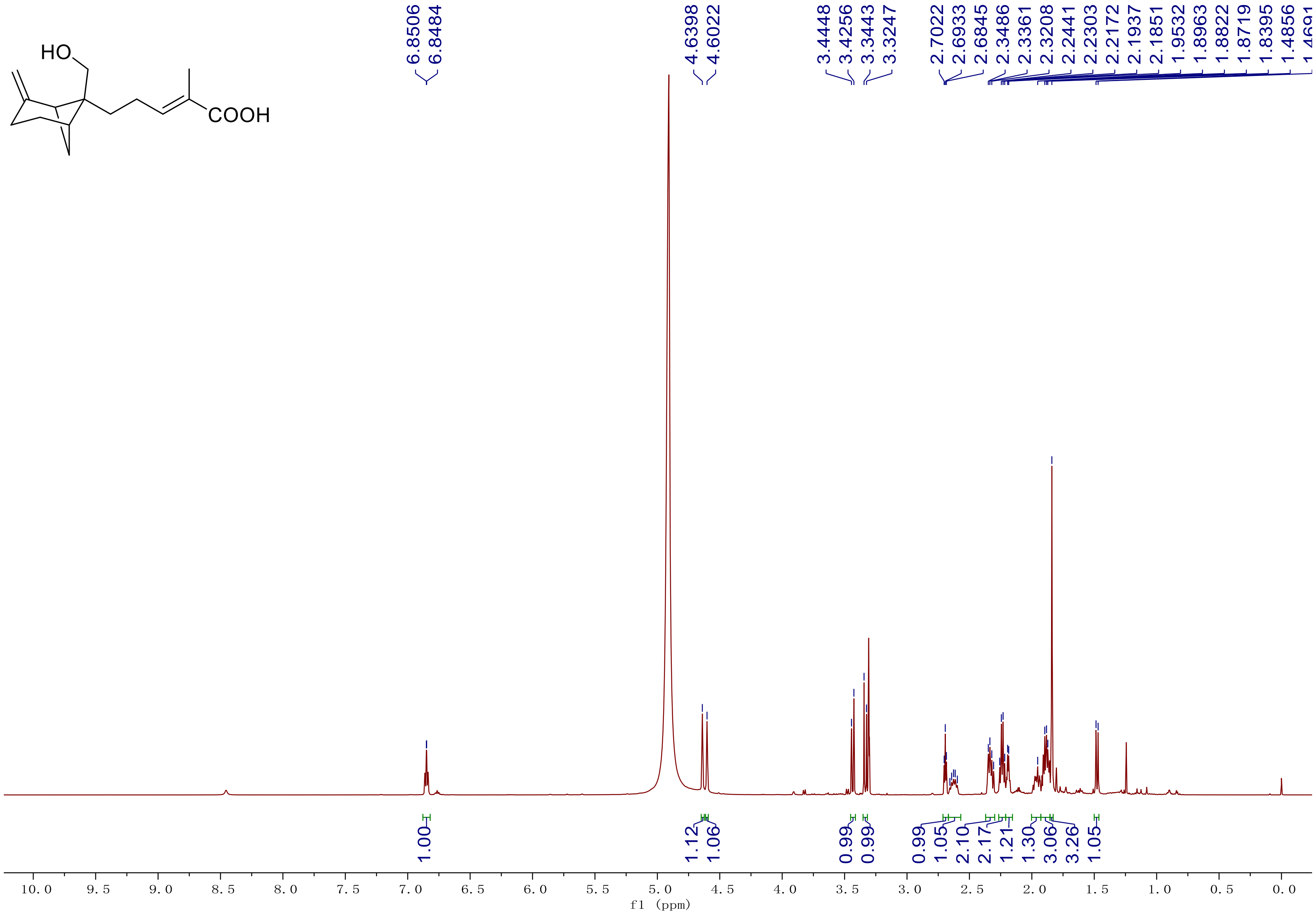

Figure S130. ^{13}C NMR and DEPT of compound **16** in CD_3OD

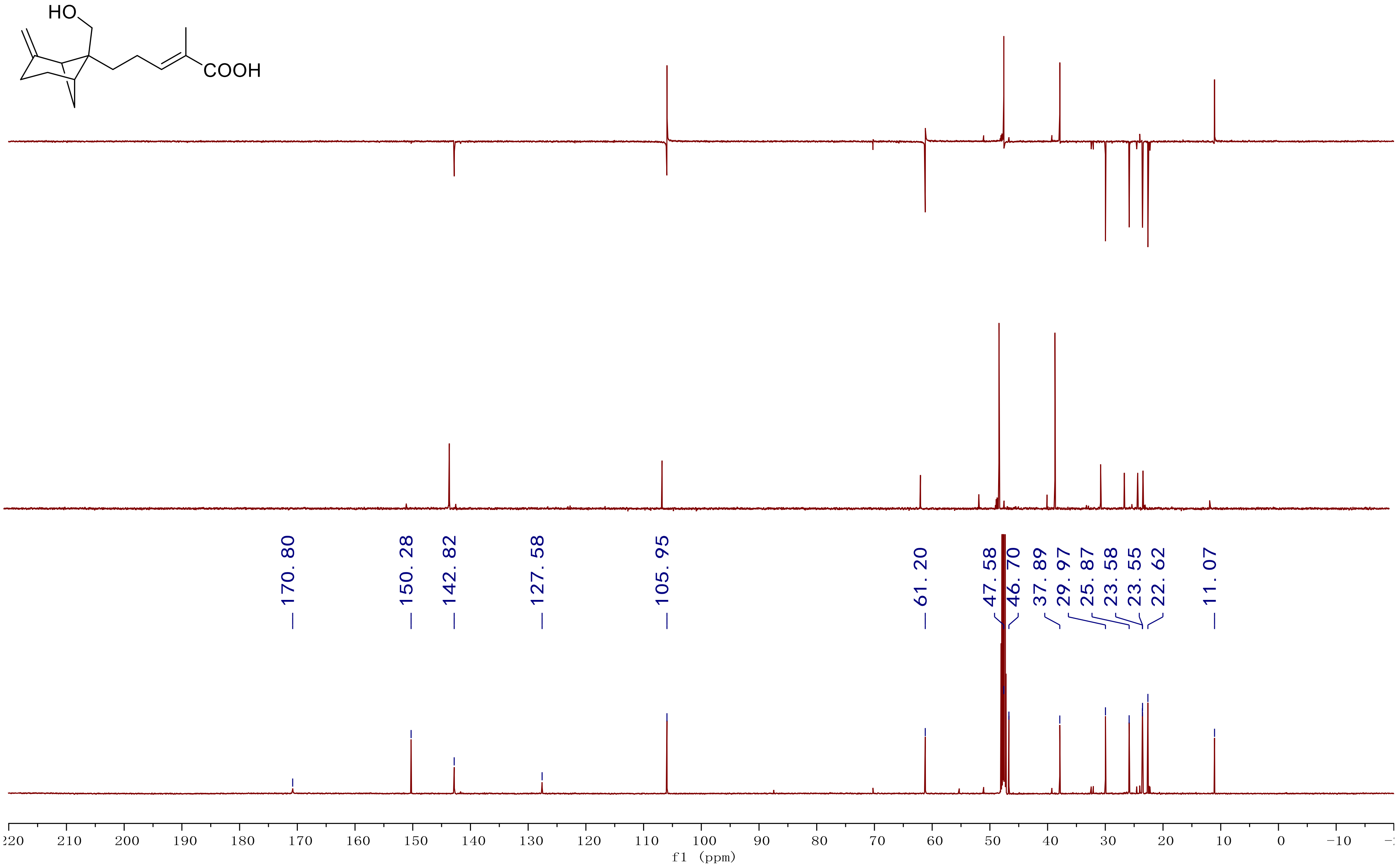

Figure S131. HSQC of compound **16** in CD_3OD

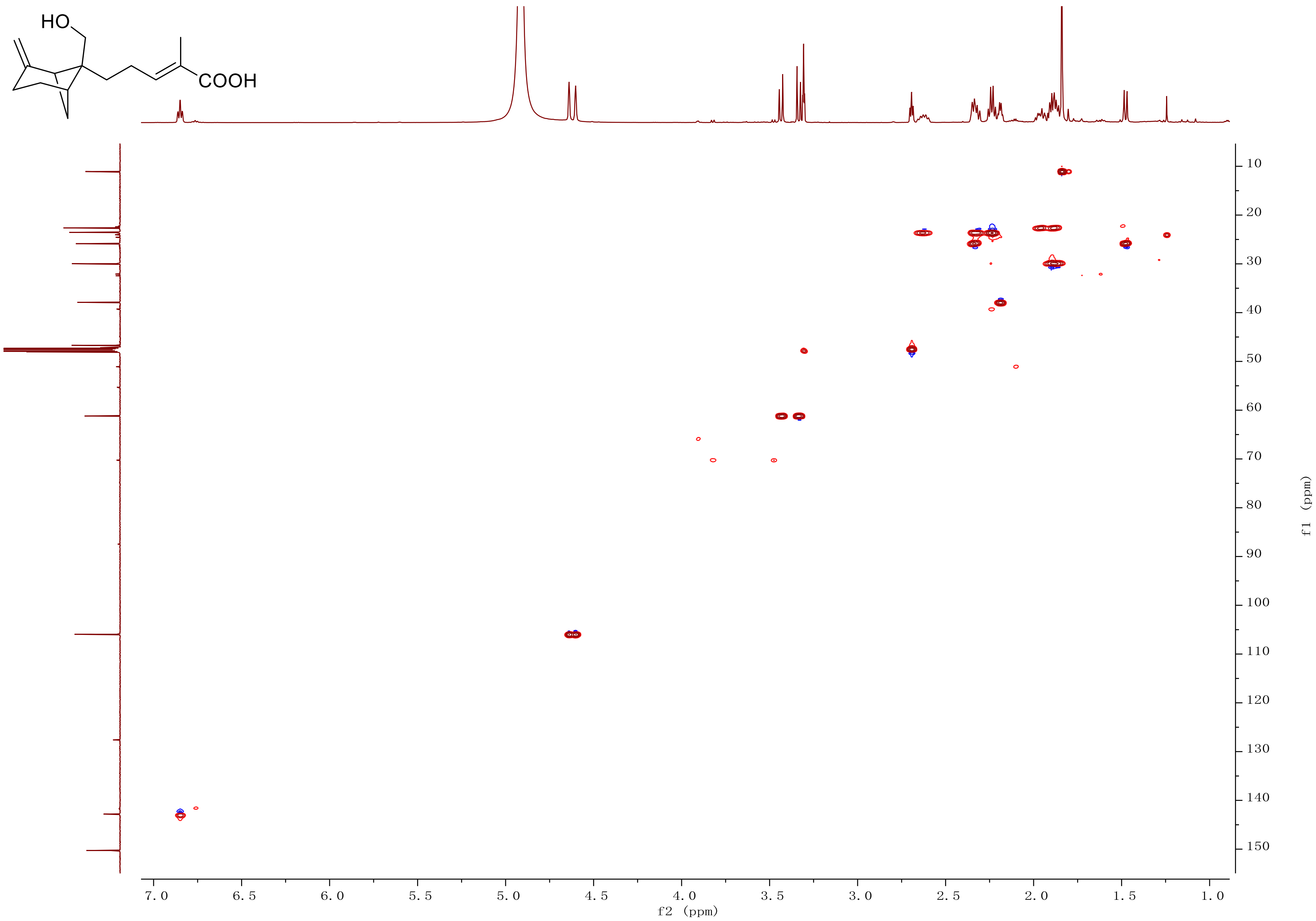

Figure S132. HMBC of compound **16** in CD_3OD

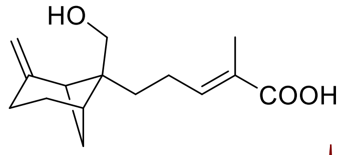

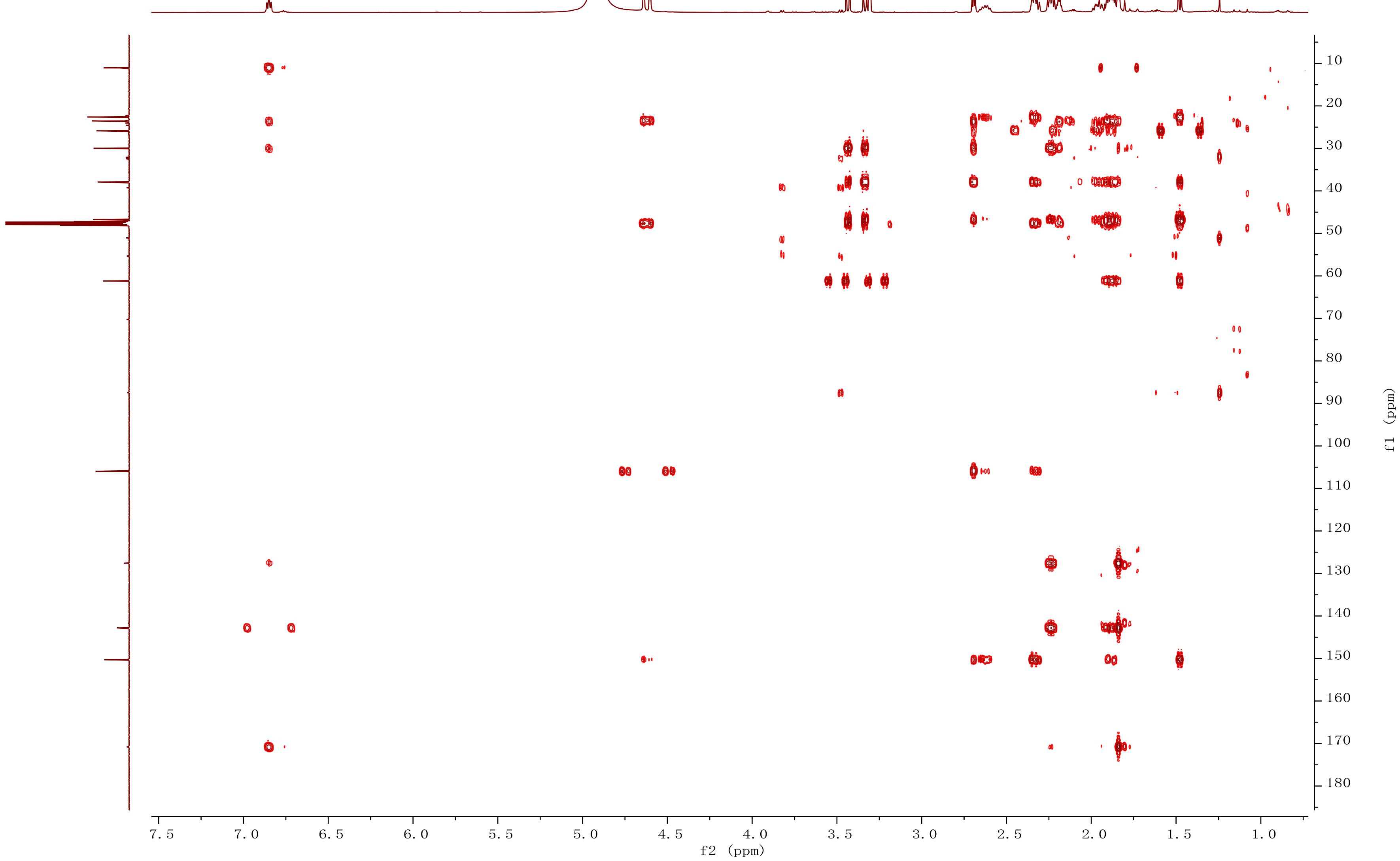

Figure S133. ^{1}H-^{1}H COSY of compound **16** in CD_3OD

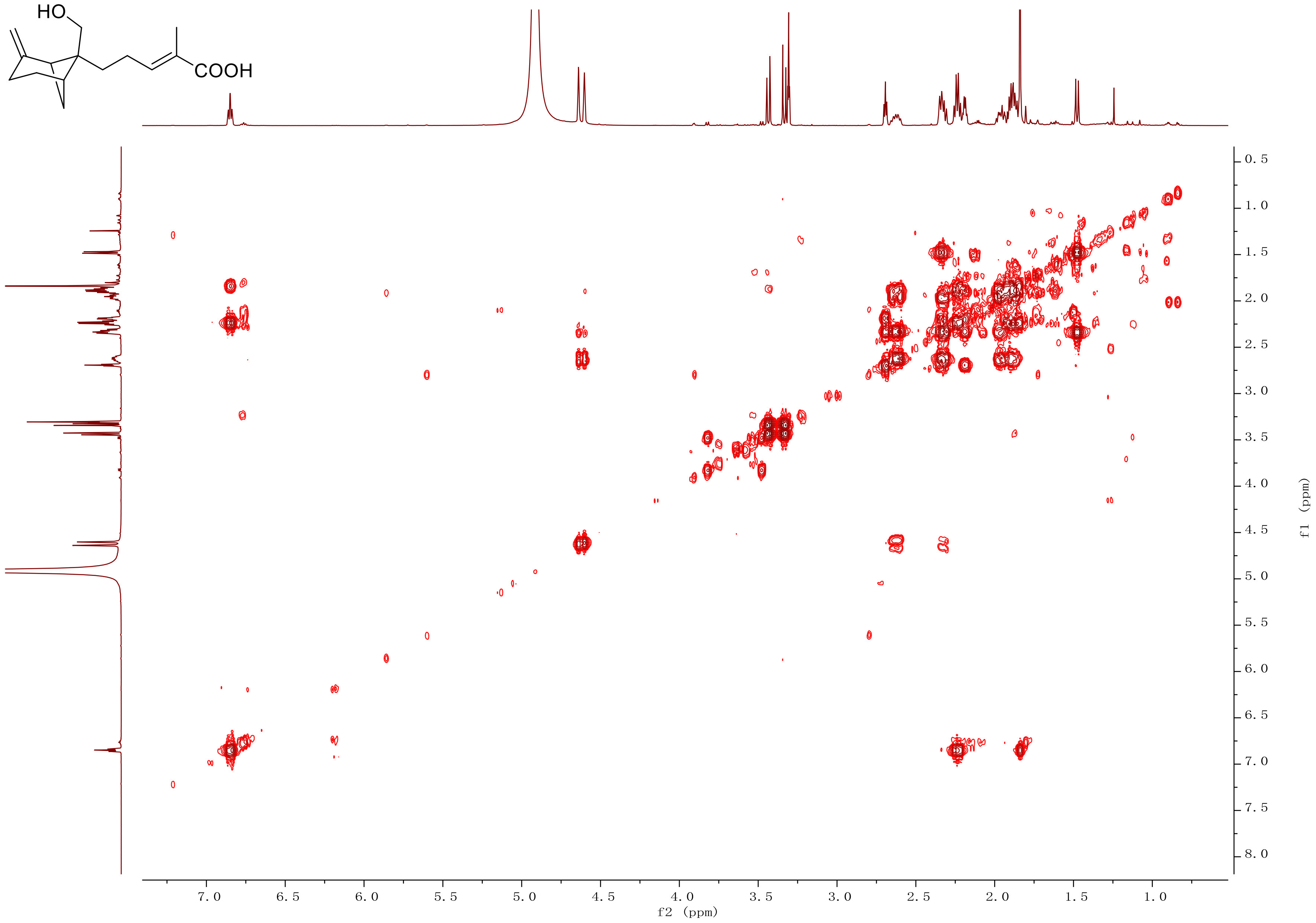

Figure S134. ROESY of compound **16** in CD_3OD

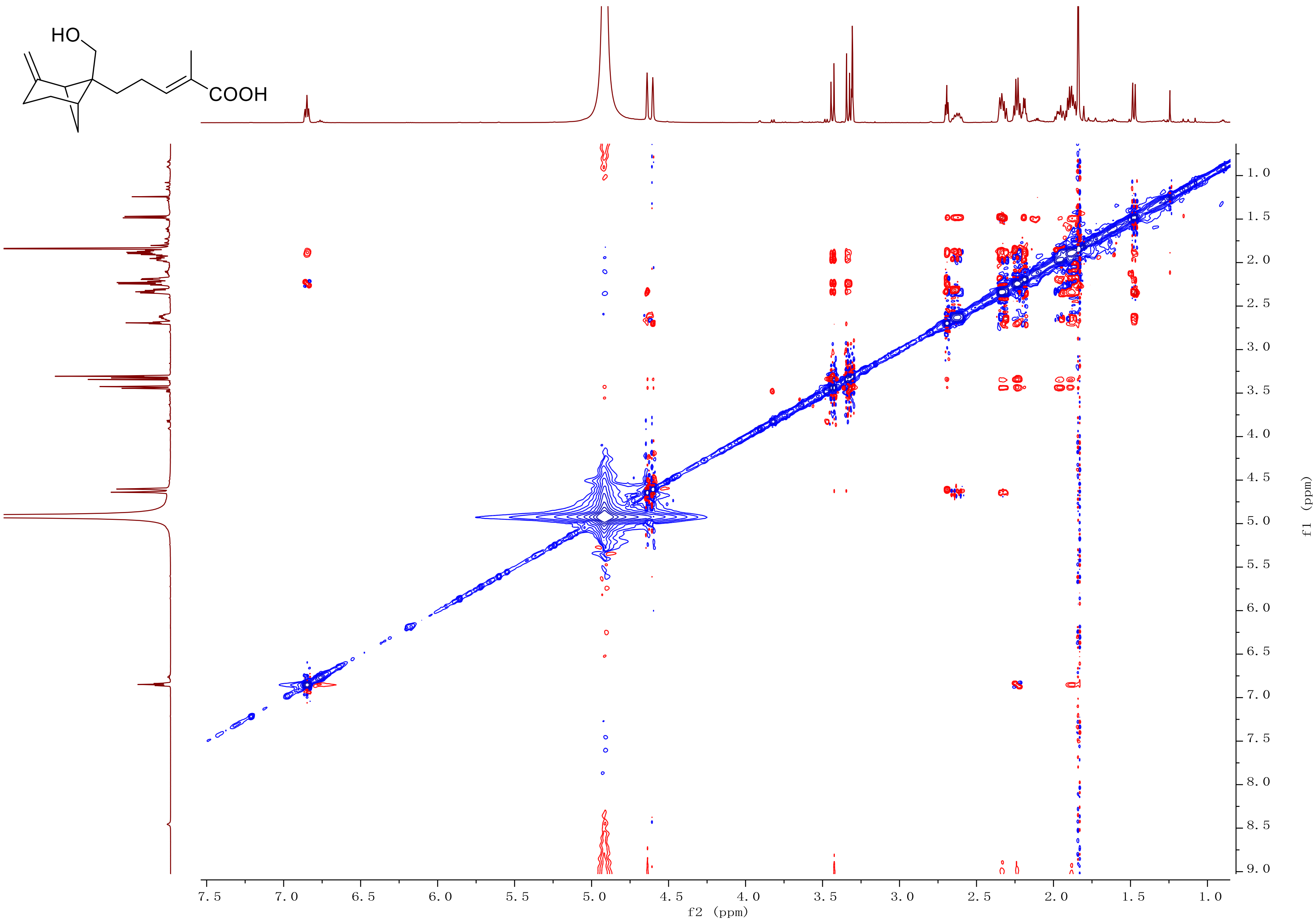

Figure S135. HR-ESIMS of compound **16**

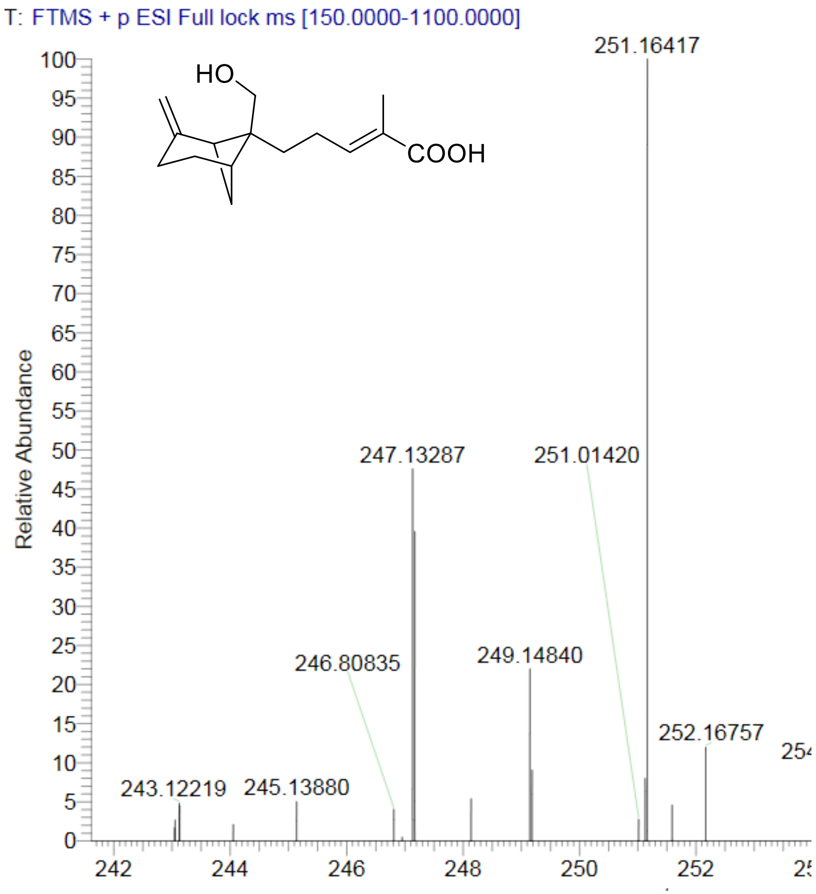

Section S24. NMR and MS spectra for 17

Figure S136. ^{1}H NMR of compound **17** in CD_3OD

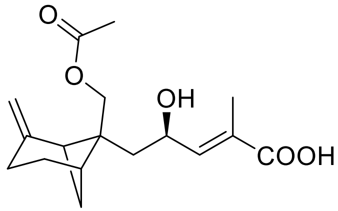

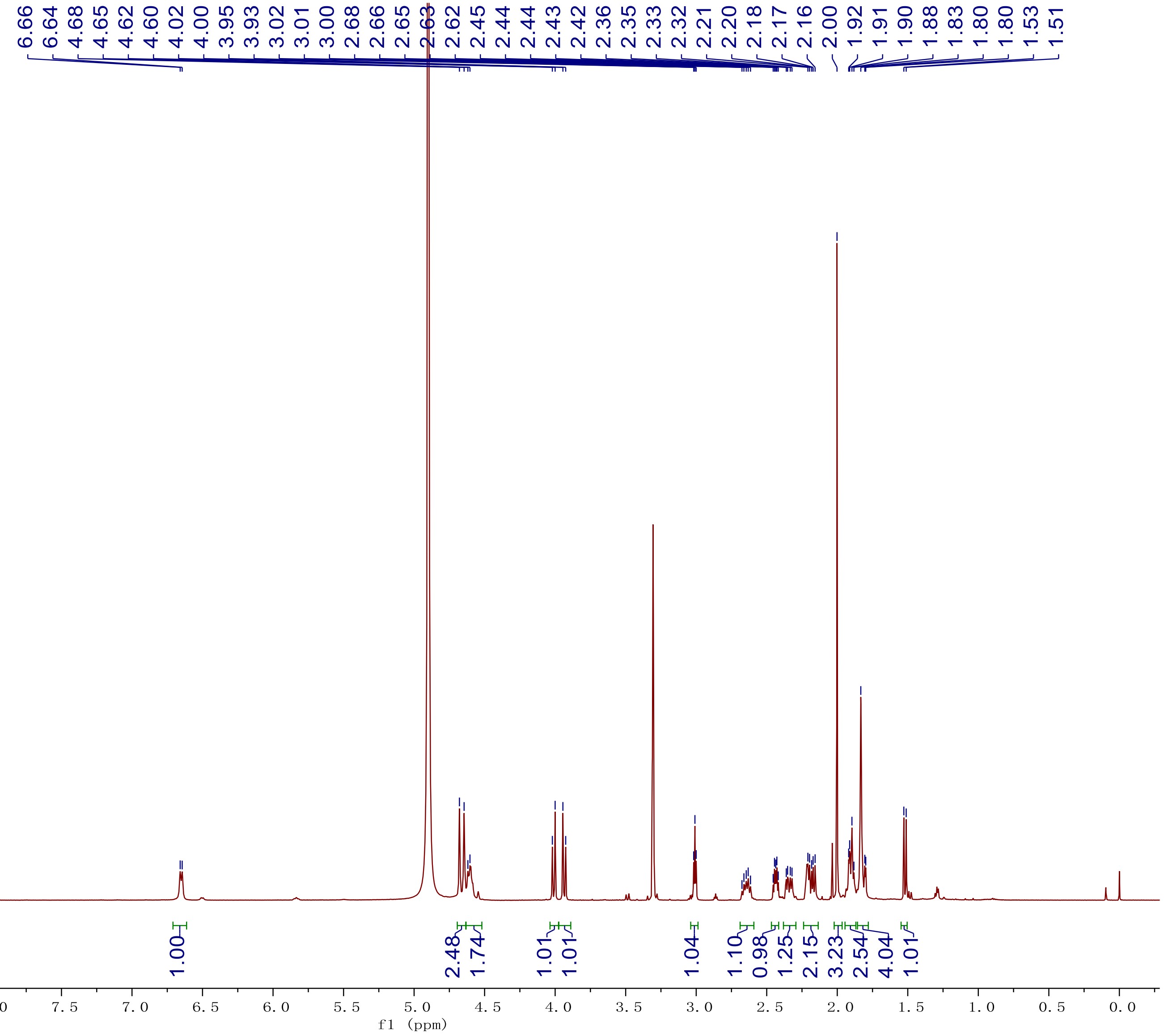

Figure S137. ^{13}C NMR and DEPT of compound **17** in CD_3OD

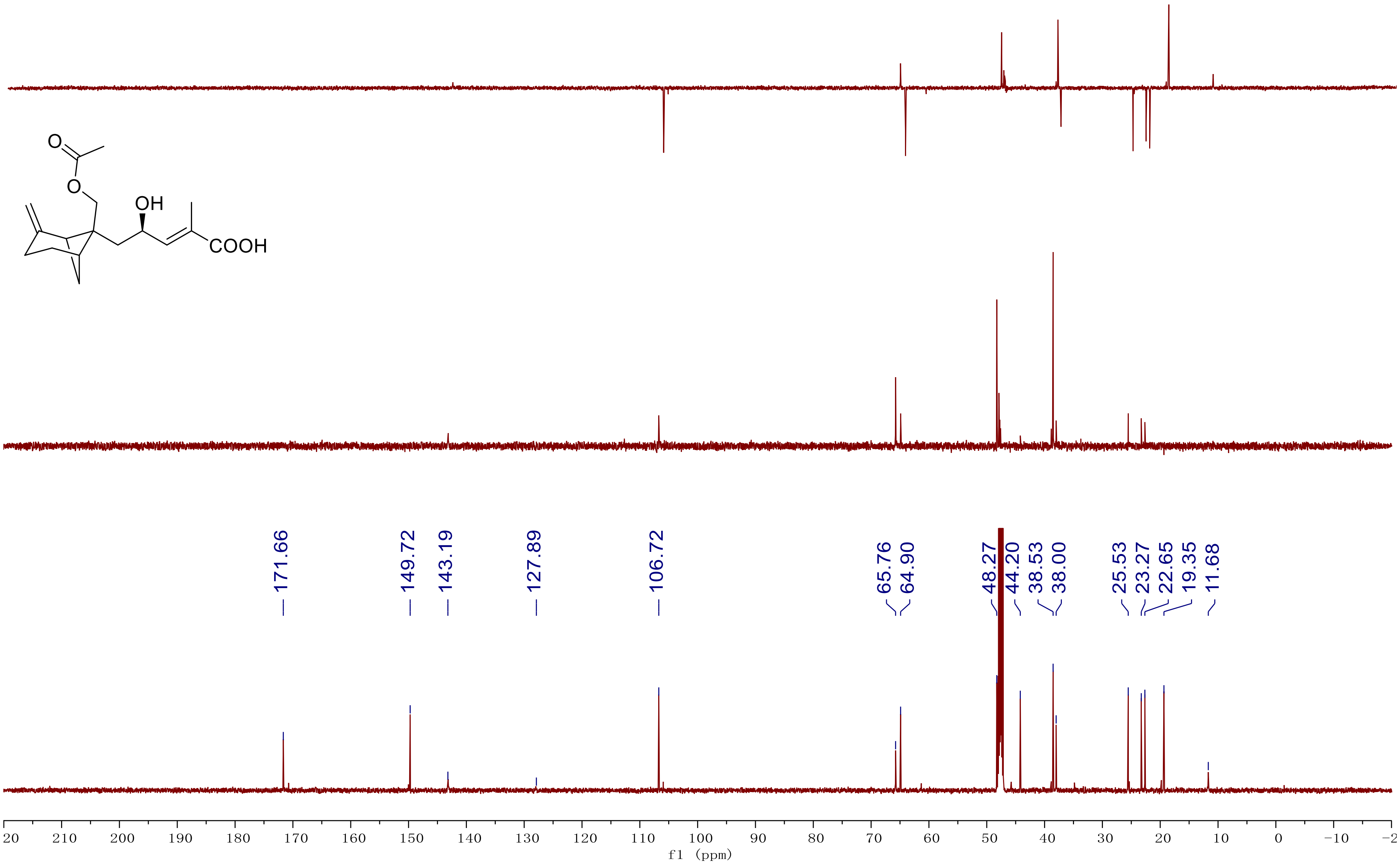

Figure S138. HSQC of compound **17** in CD_3OD

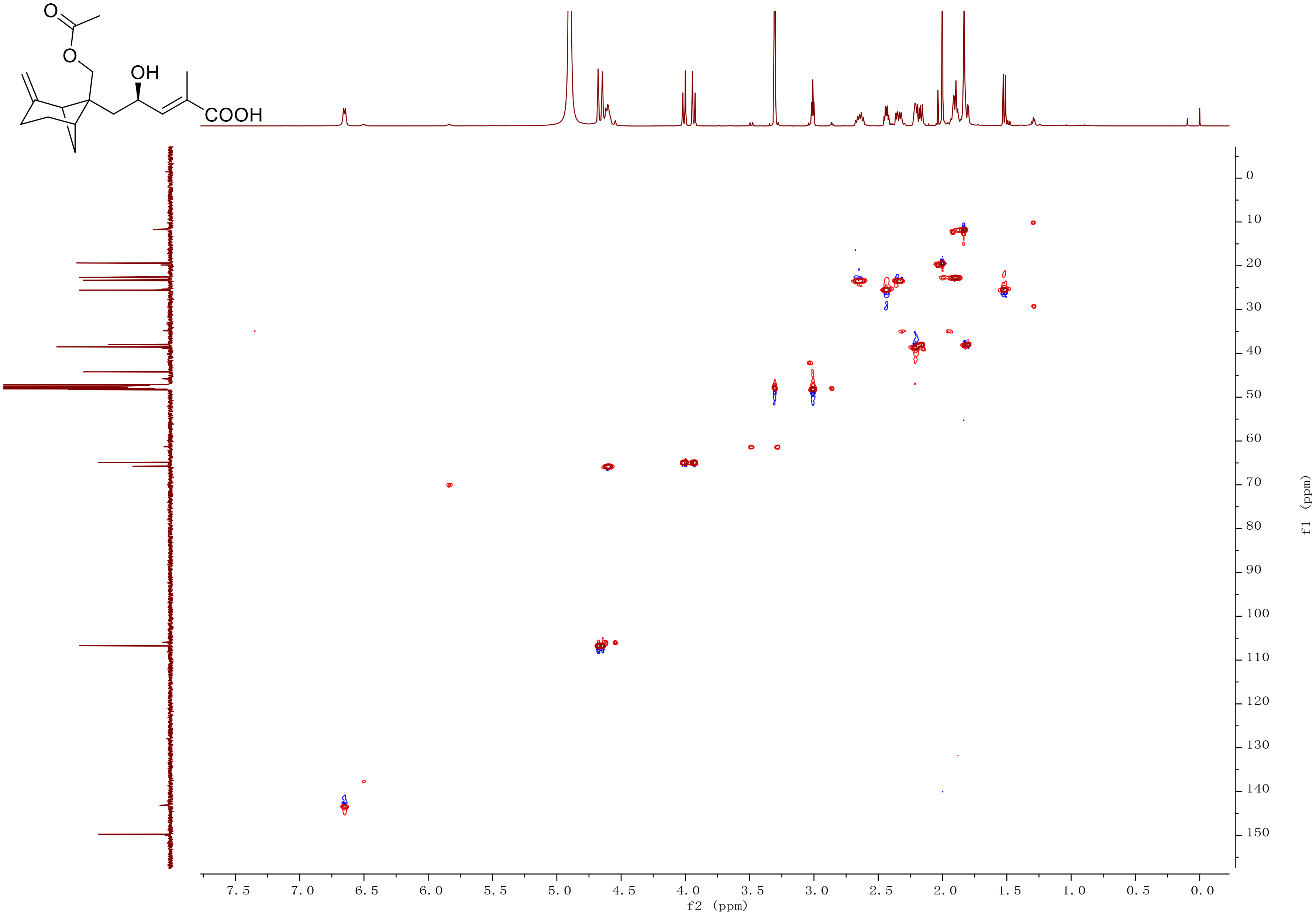

Figure S139. HMBC of compound **17** in CD_3OD

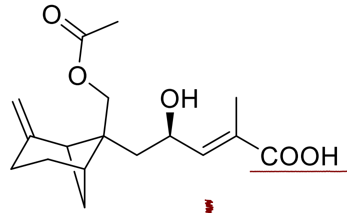

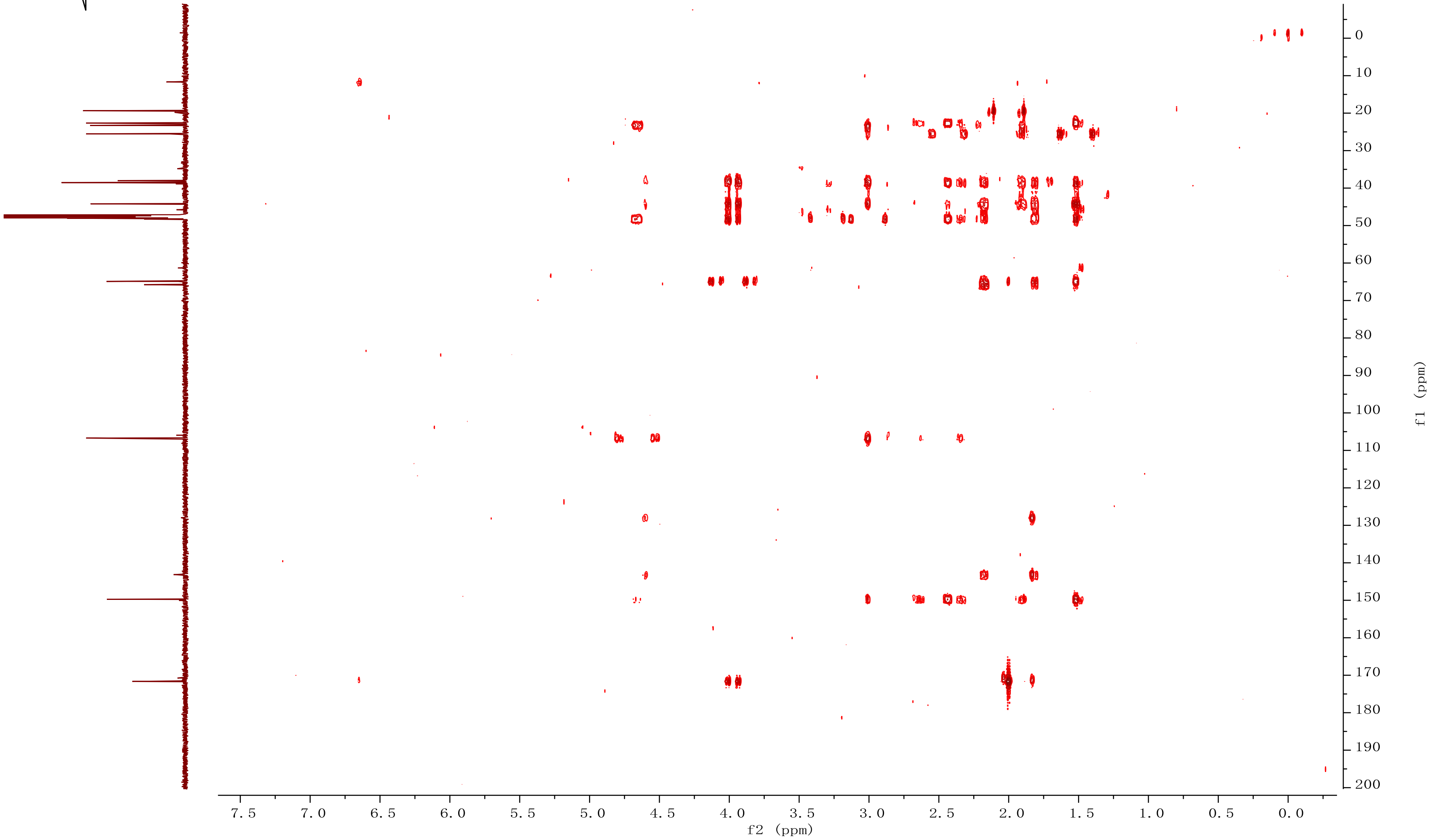

Figure S140. ^{1}H-^{1}H COSY of compound **17** in CD_3OD

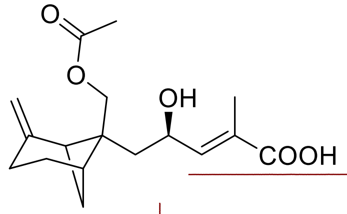

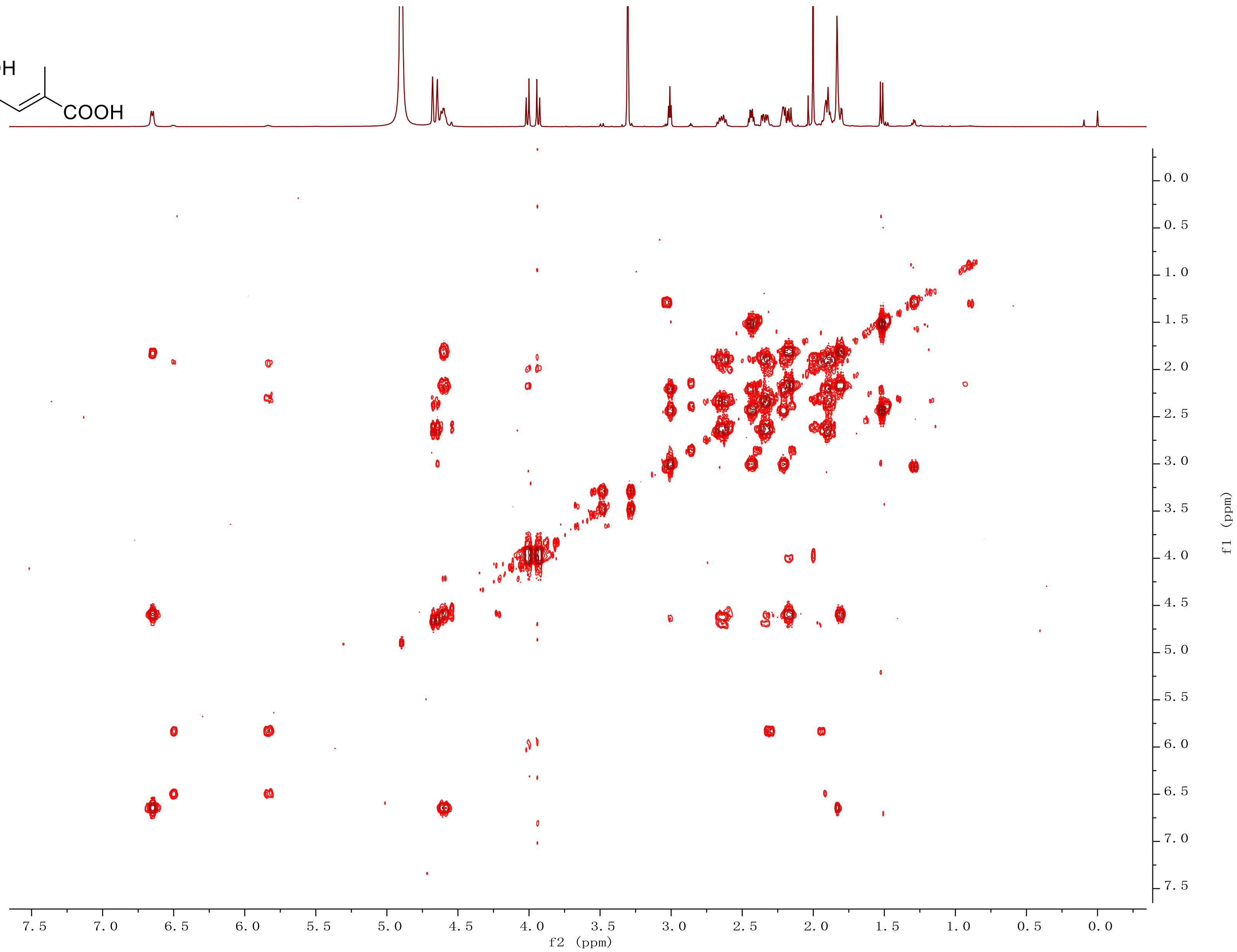

Figure S141. ROESY of compound **17** in CD_3OD

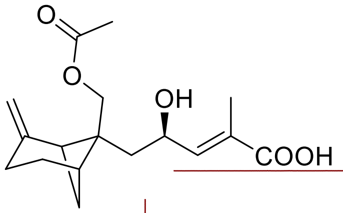

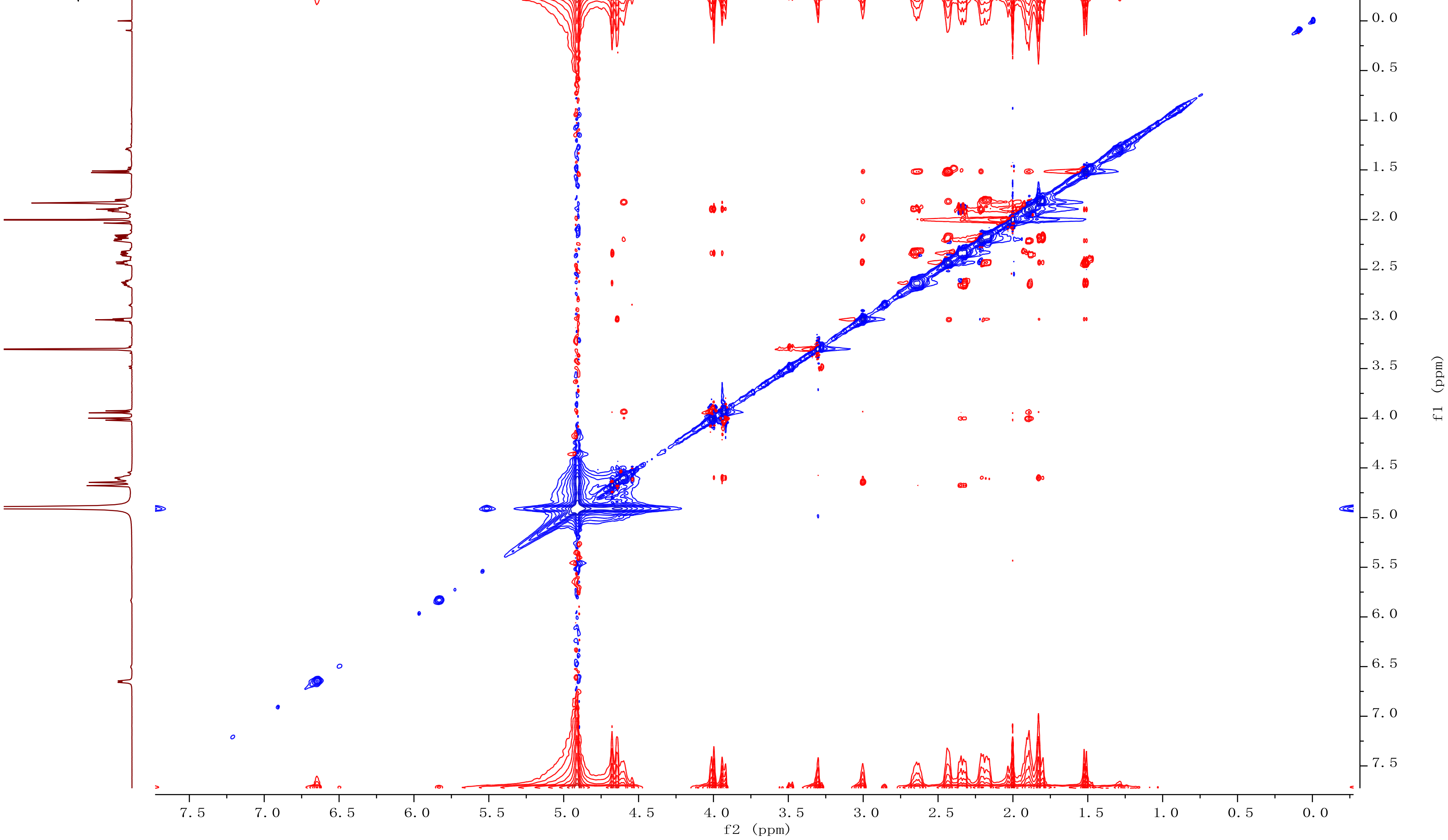

Figure S142. HR-ESIMS of compound **17**

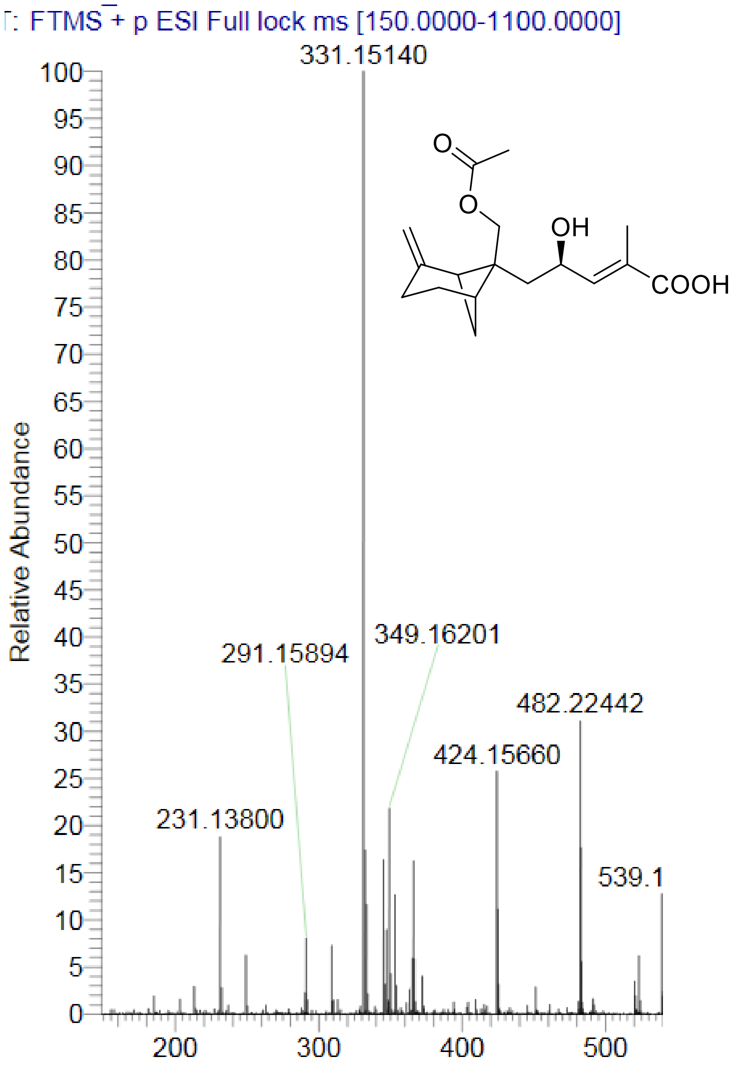

Section S25. NMR and MS spectra for 18

Figure S143. ^{1}H NMR of compound **18** in CD_3OD

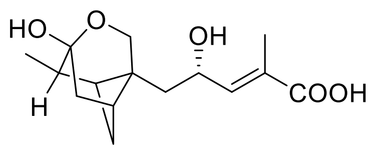

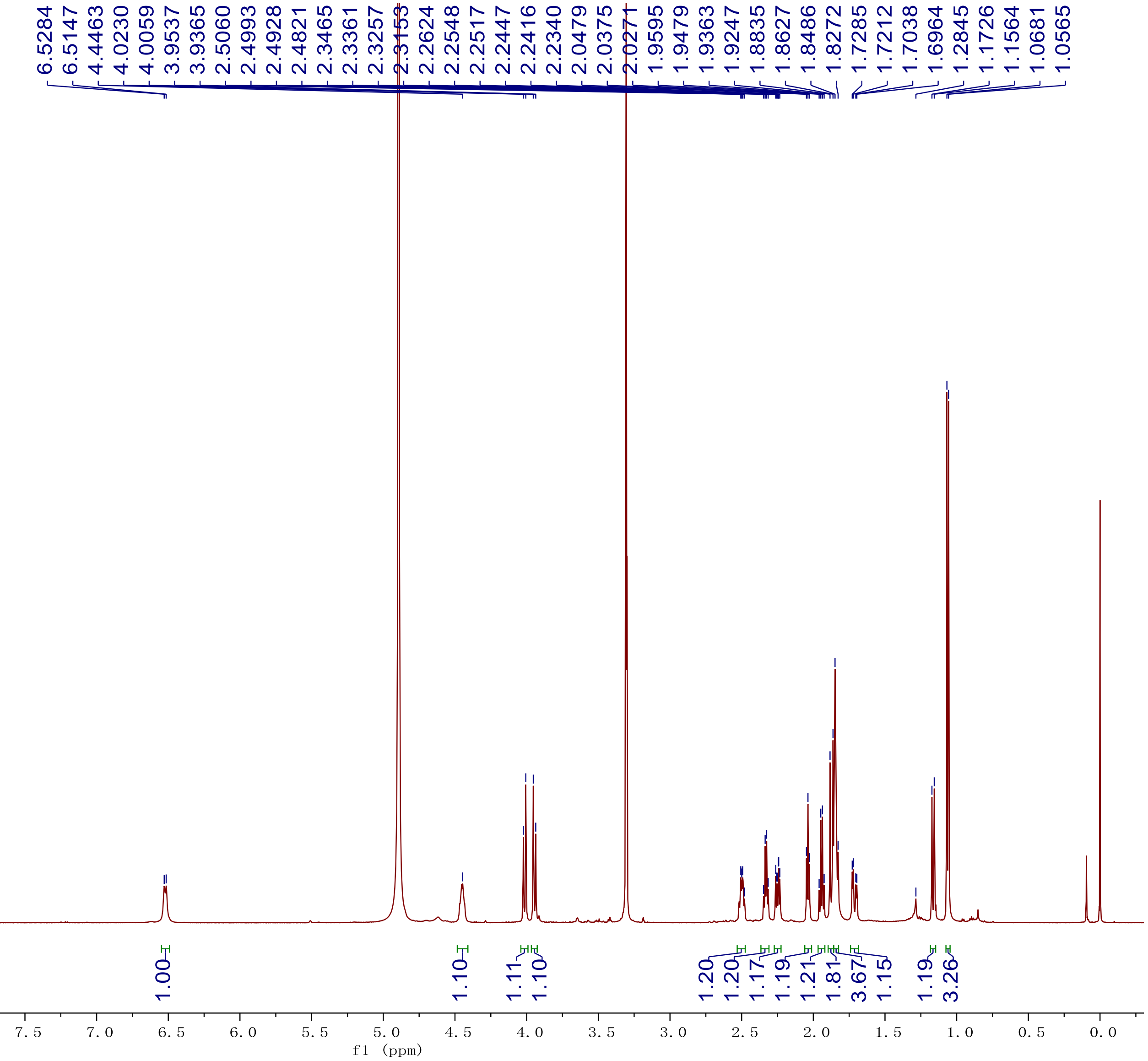

Figure S144. ^{13}C NMR and DEPT of compound **18** in CD_3OD

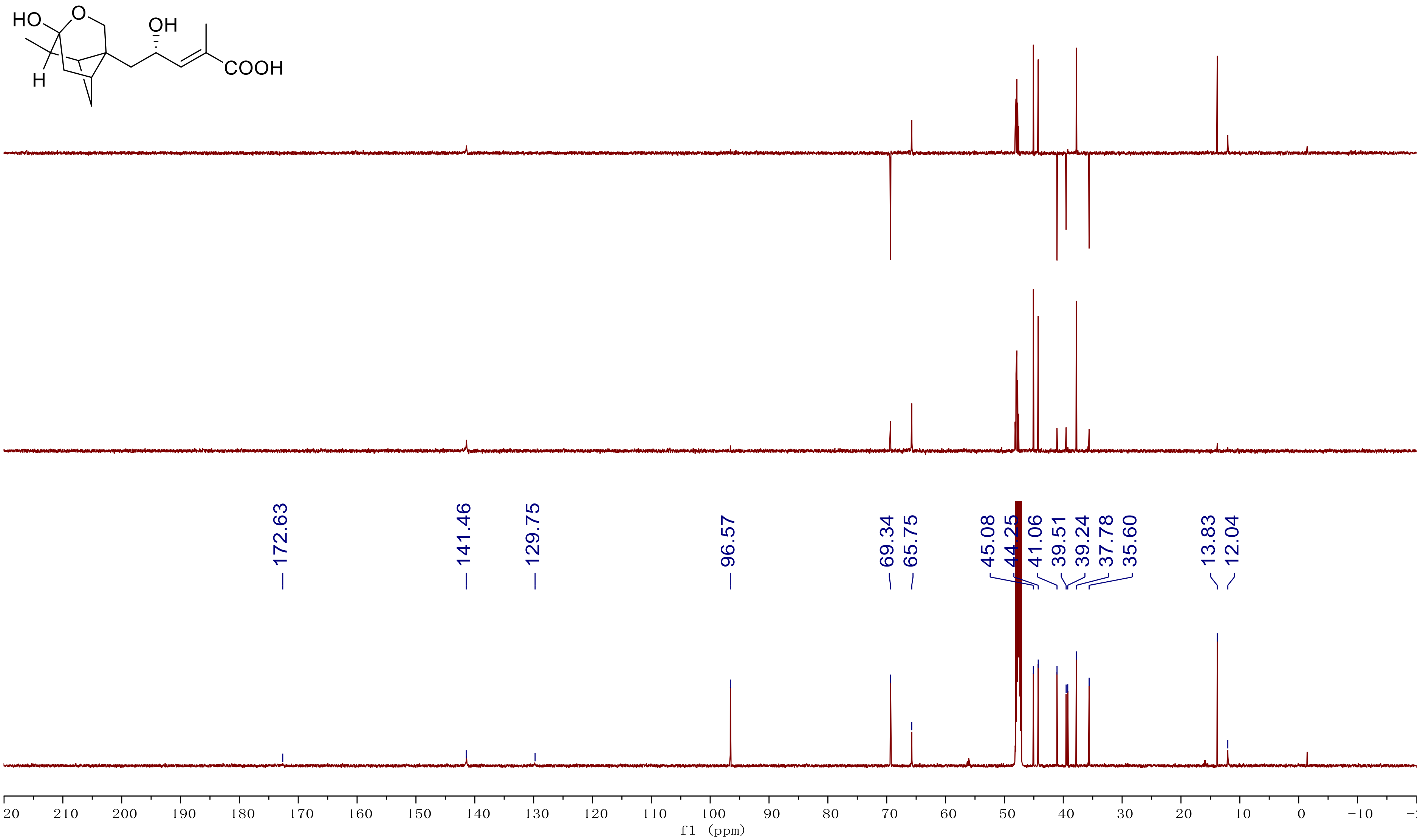

Figure S145. HSQC of compound **18** in CD_3OD

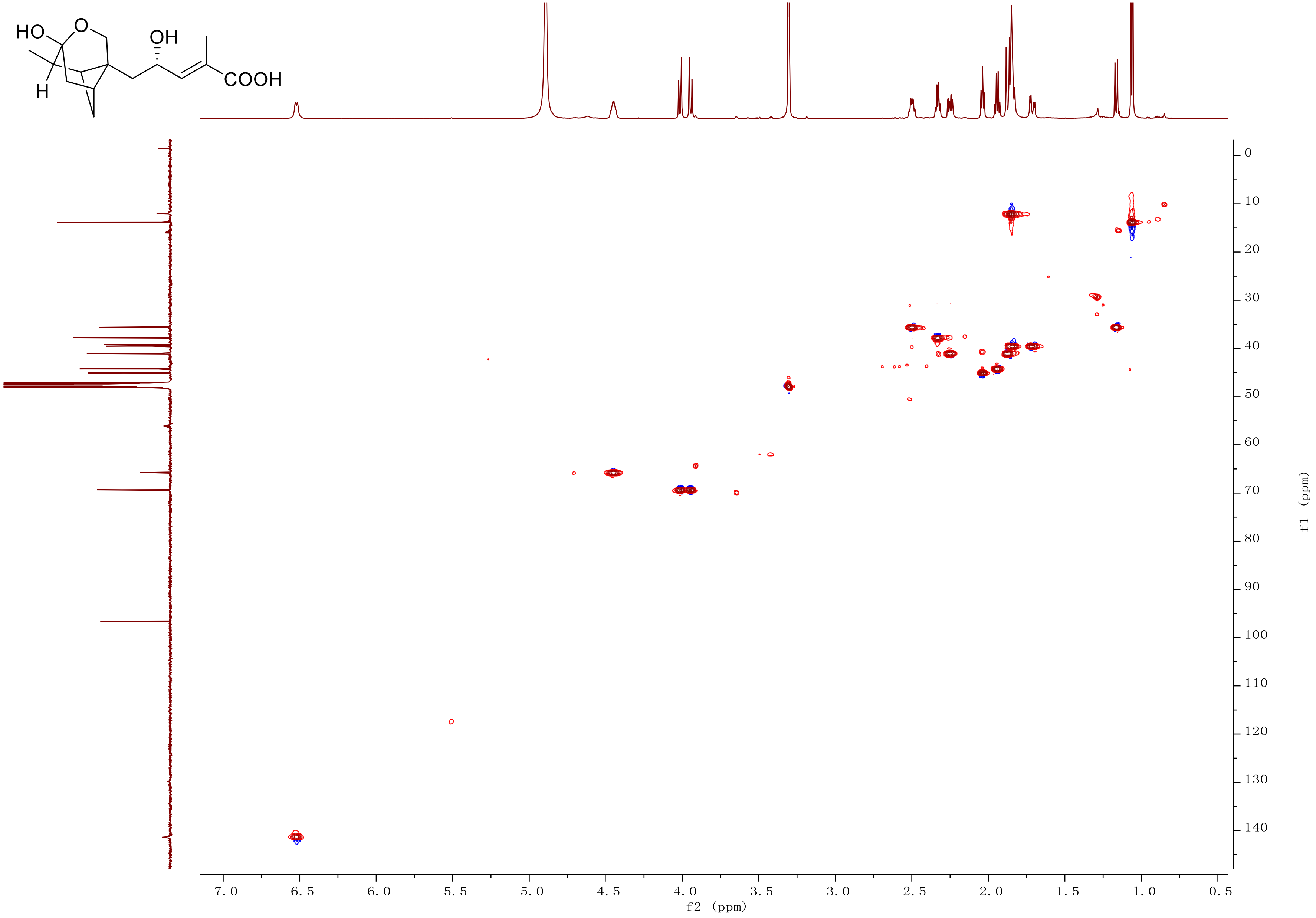

Figure S146. HMBC of compound **18** in CD_3OD

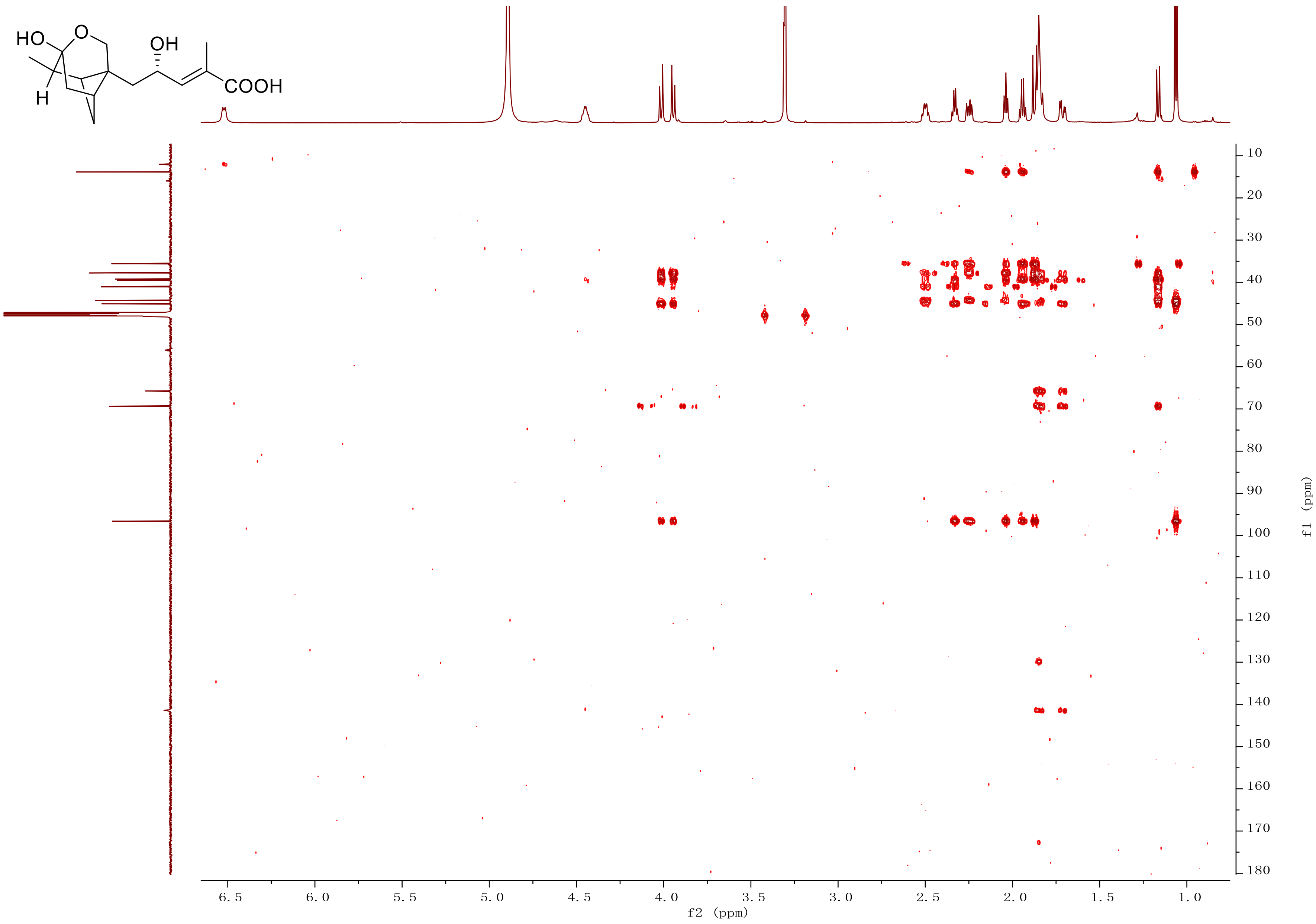

Figure S147. ^{1}H-^{1}H COSY of compound **18** in CD_3OD

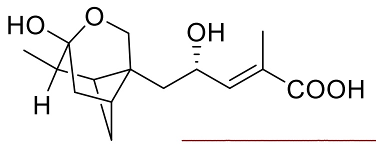

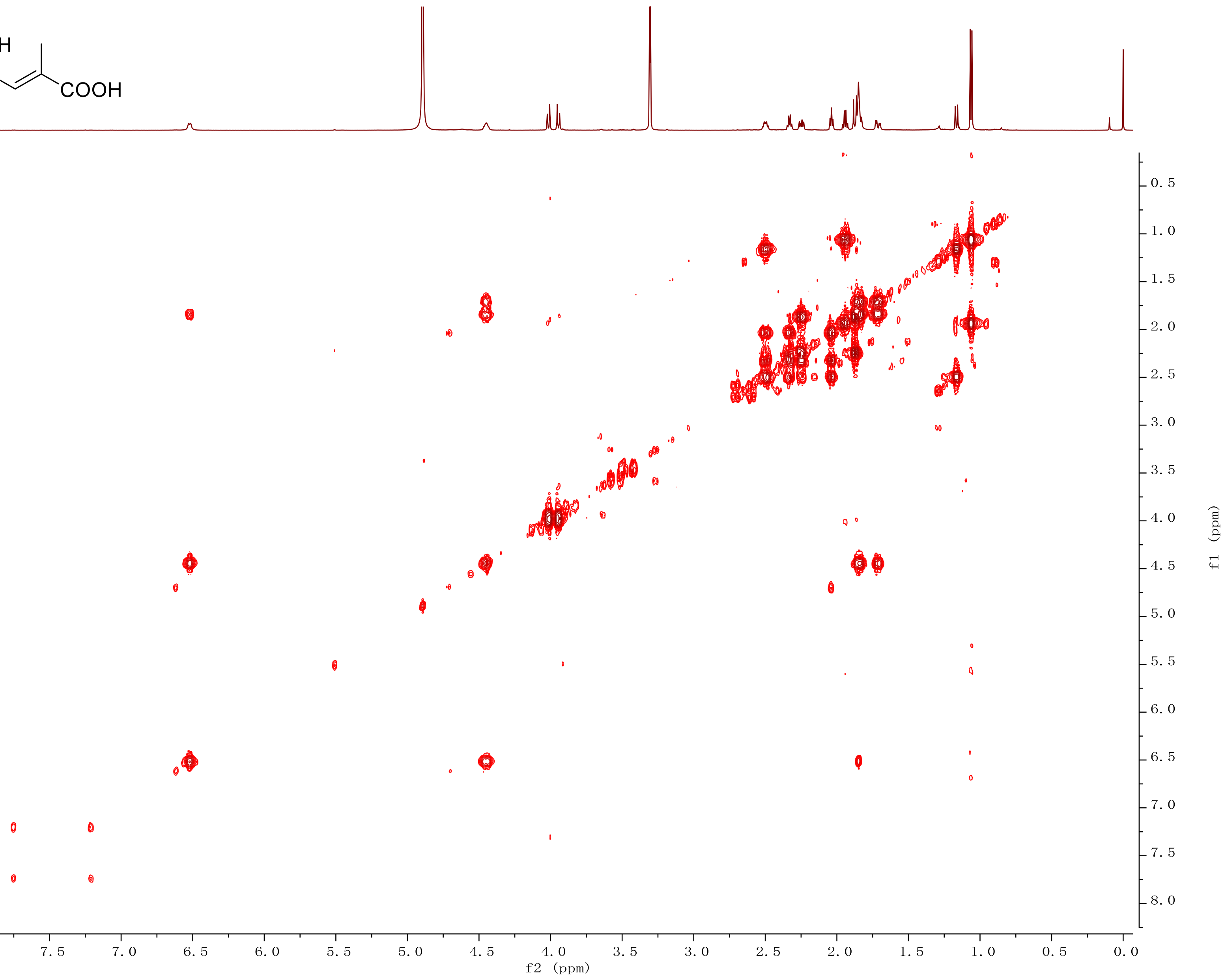

Figure S148. ROESY of compound **18** in CD_3OD

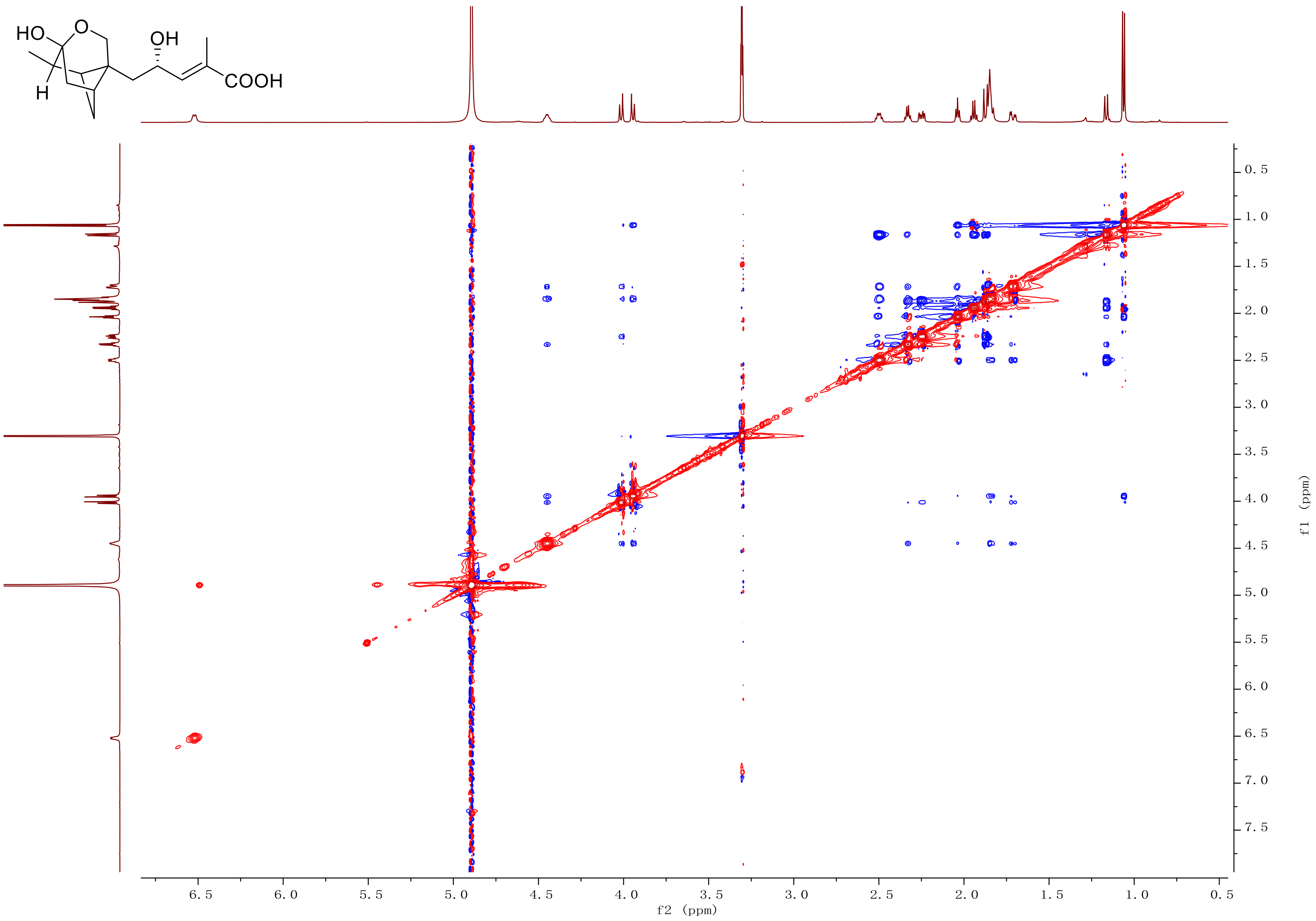

Figure S149. HR-ESIMS of compound **18**

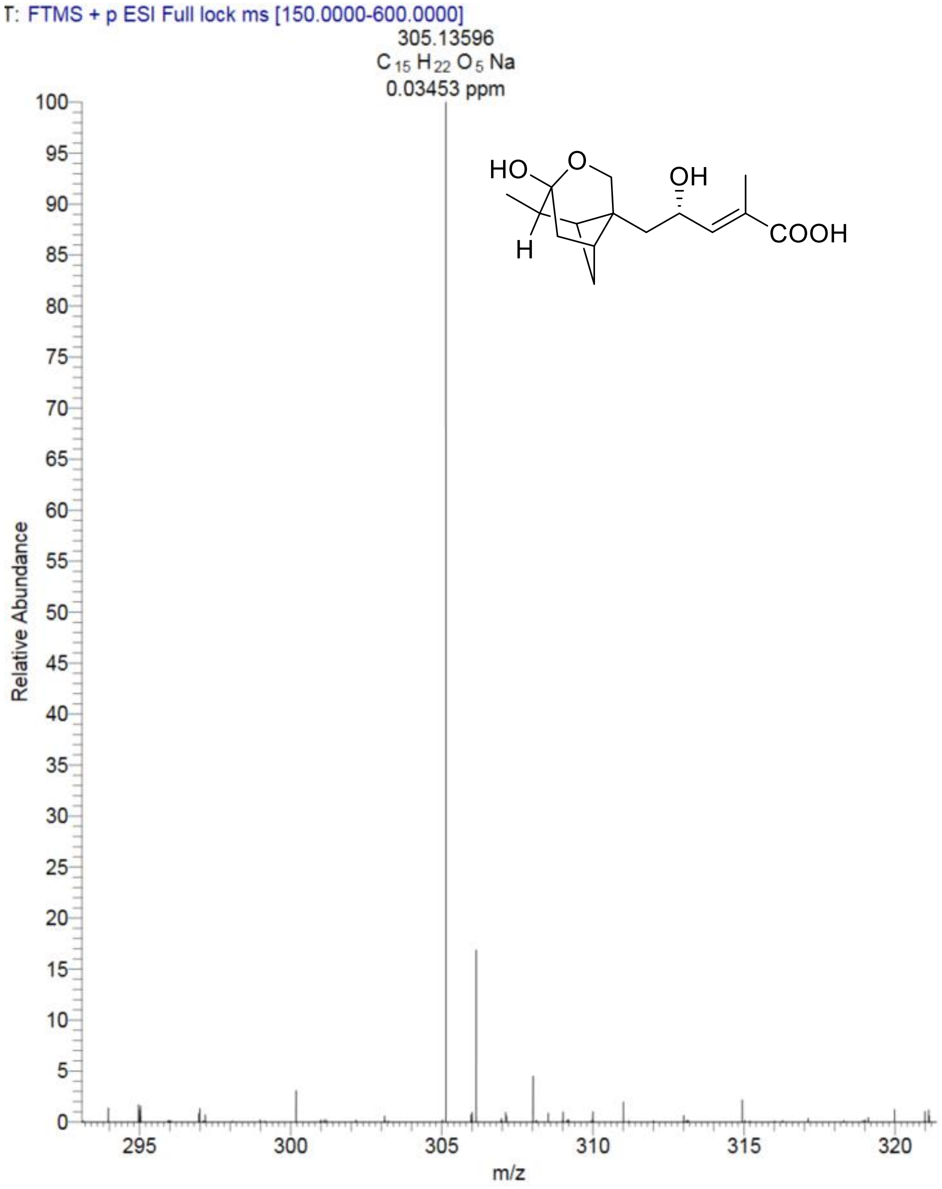

Section S26. NMR and MS spectra for 19

Figure S150. ^{1}H NMR of compound **19** in CD_3OD

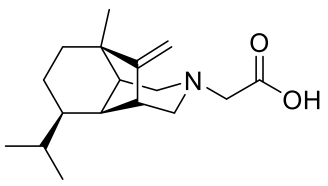

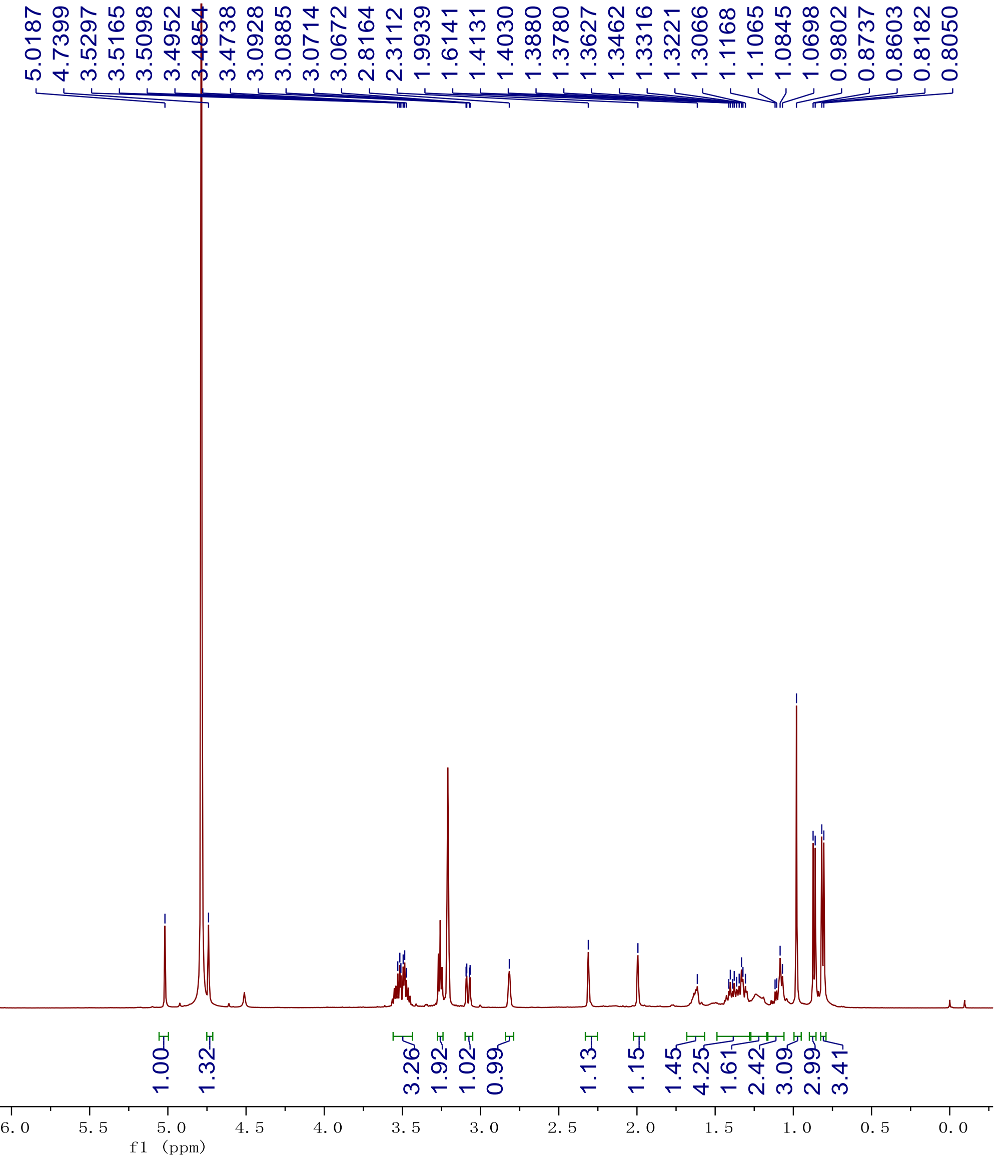

Figure S151. ^{13}C NMR and DEPT of compound **19** in CD_3OD

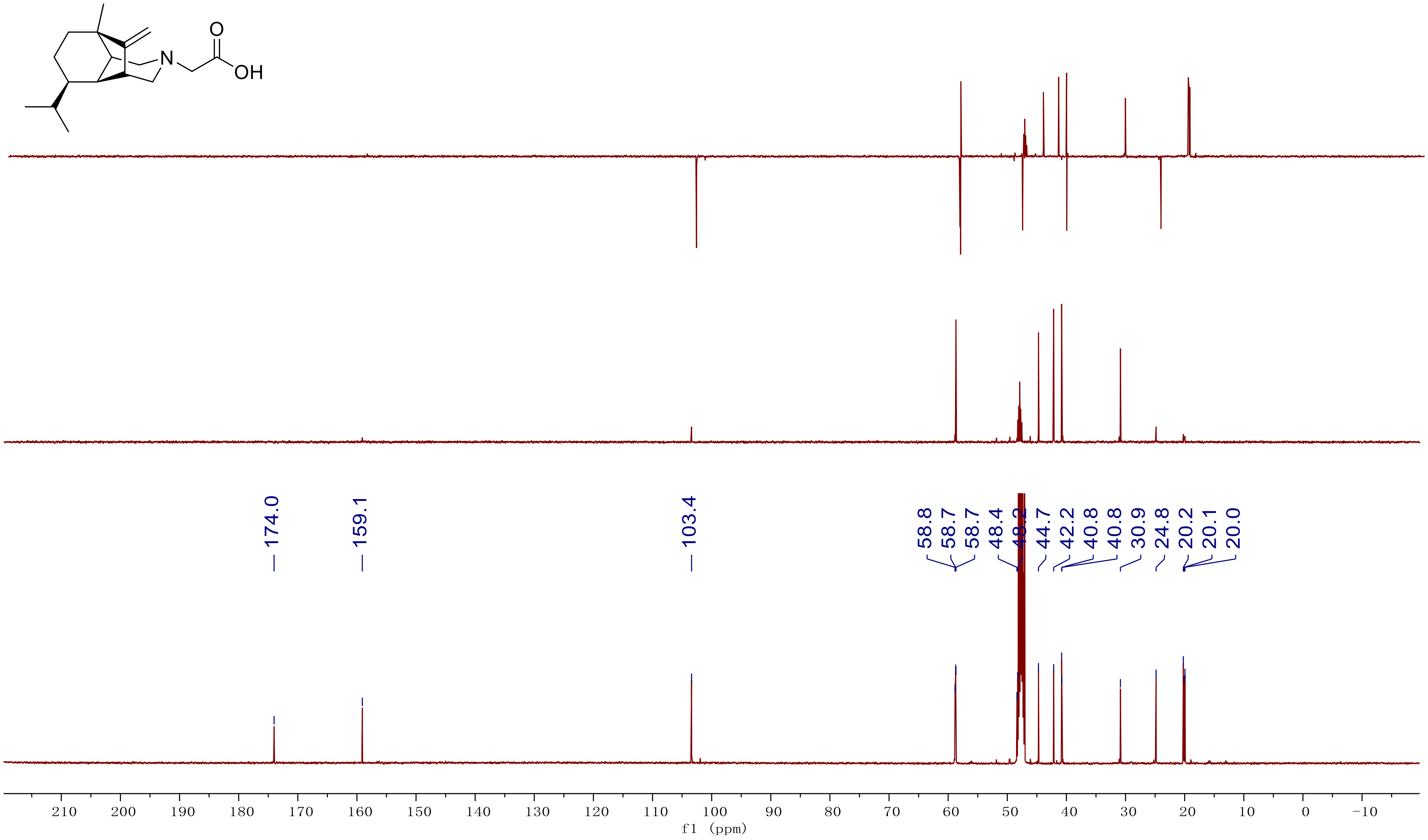

Figure S152. HSQC of compound **19** in CD_3OD

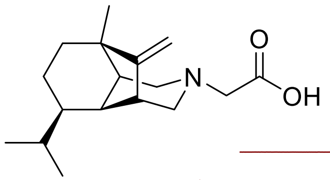

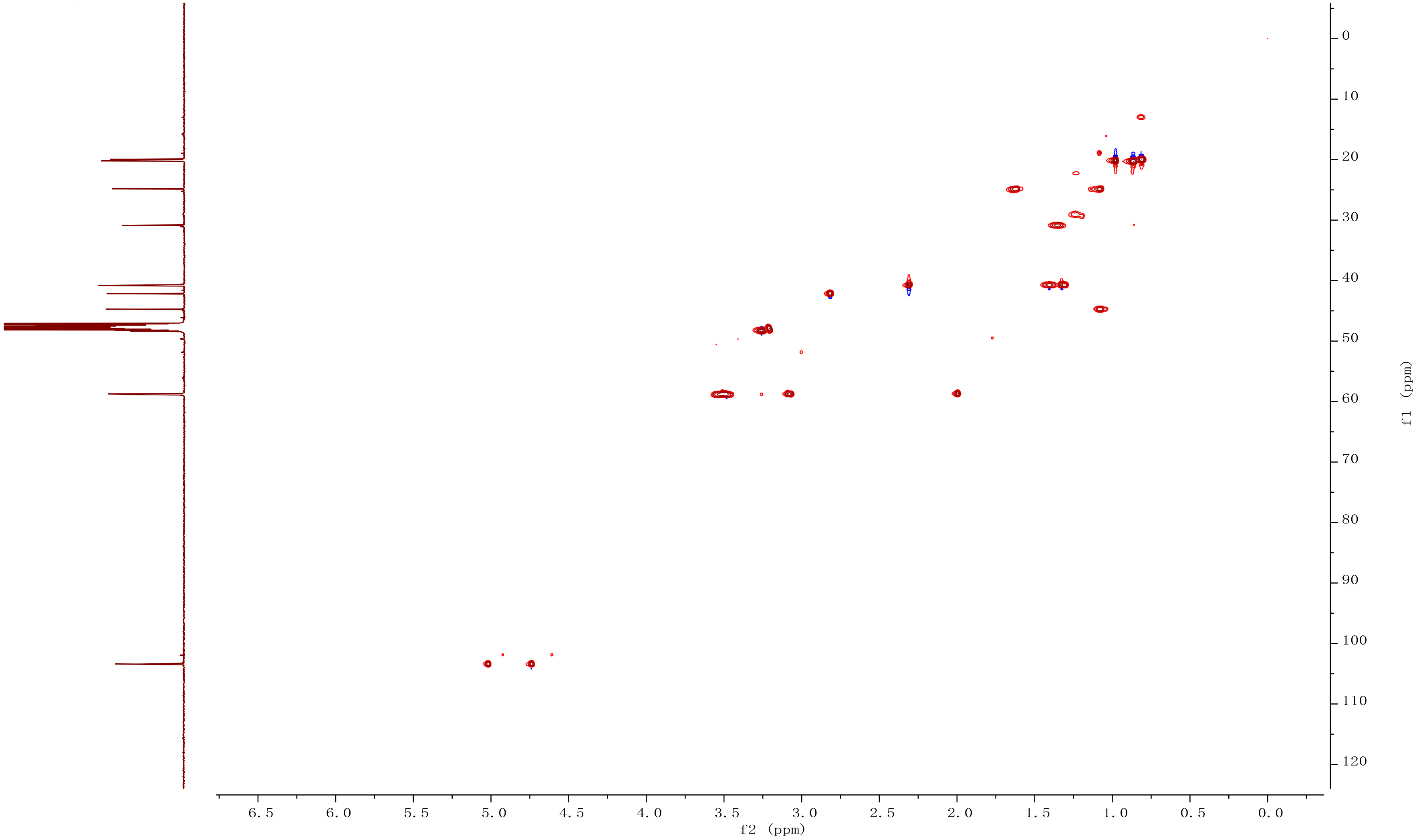

Figure S153. HMBC of compound **19** in CD_3OD

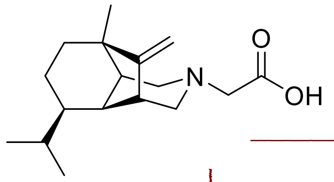

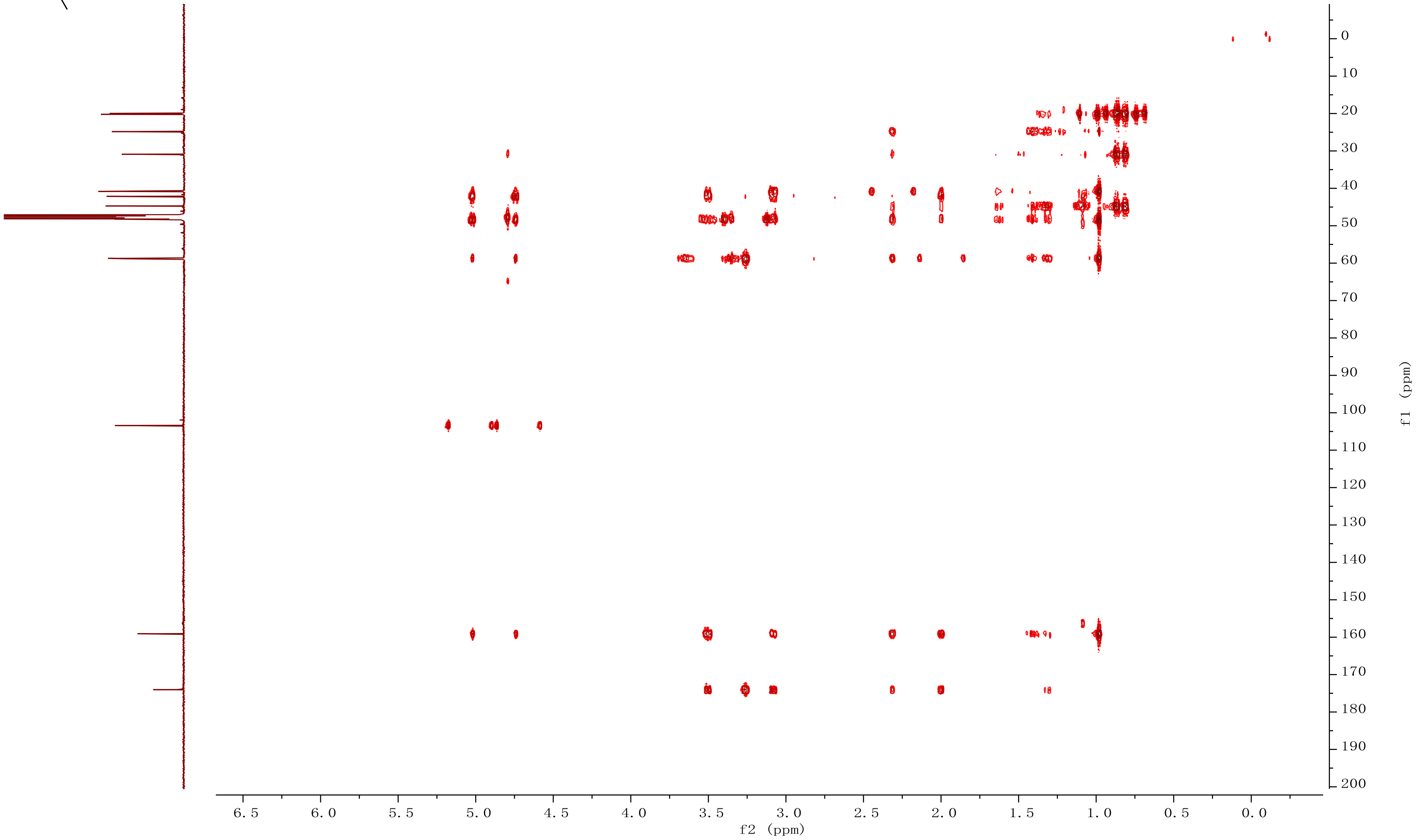

Figure S154. ^{1}H-^{1}H COSY of compound **19** in CD_3OD

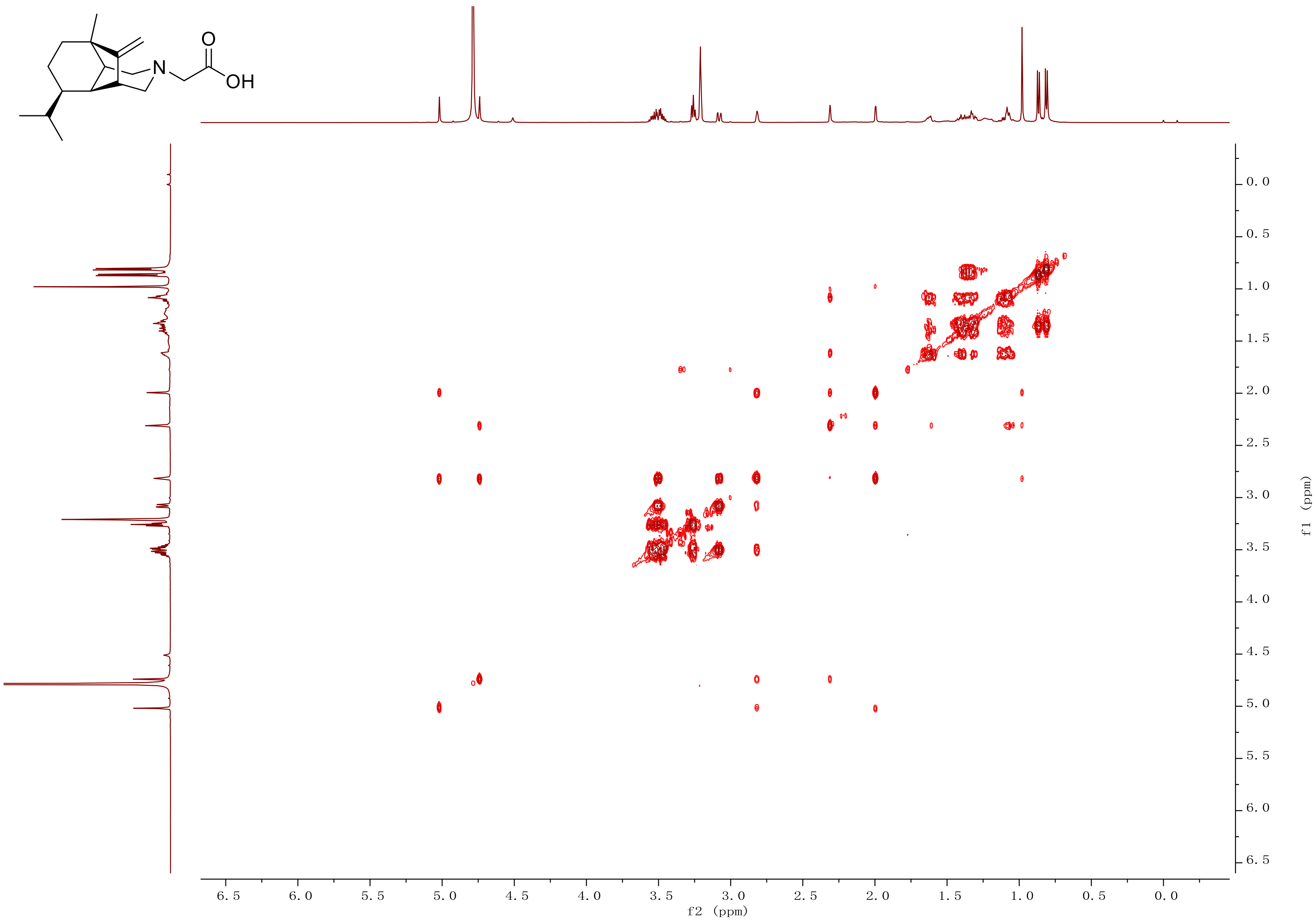

Figure S155. ROESY of compound **19** in CD_3OD

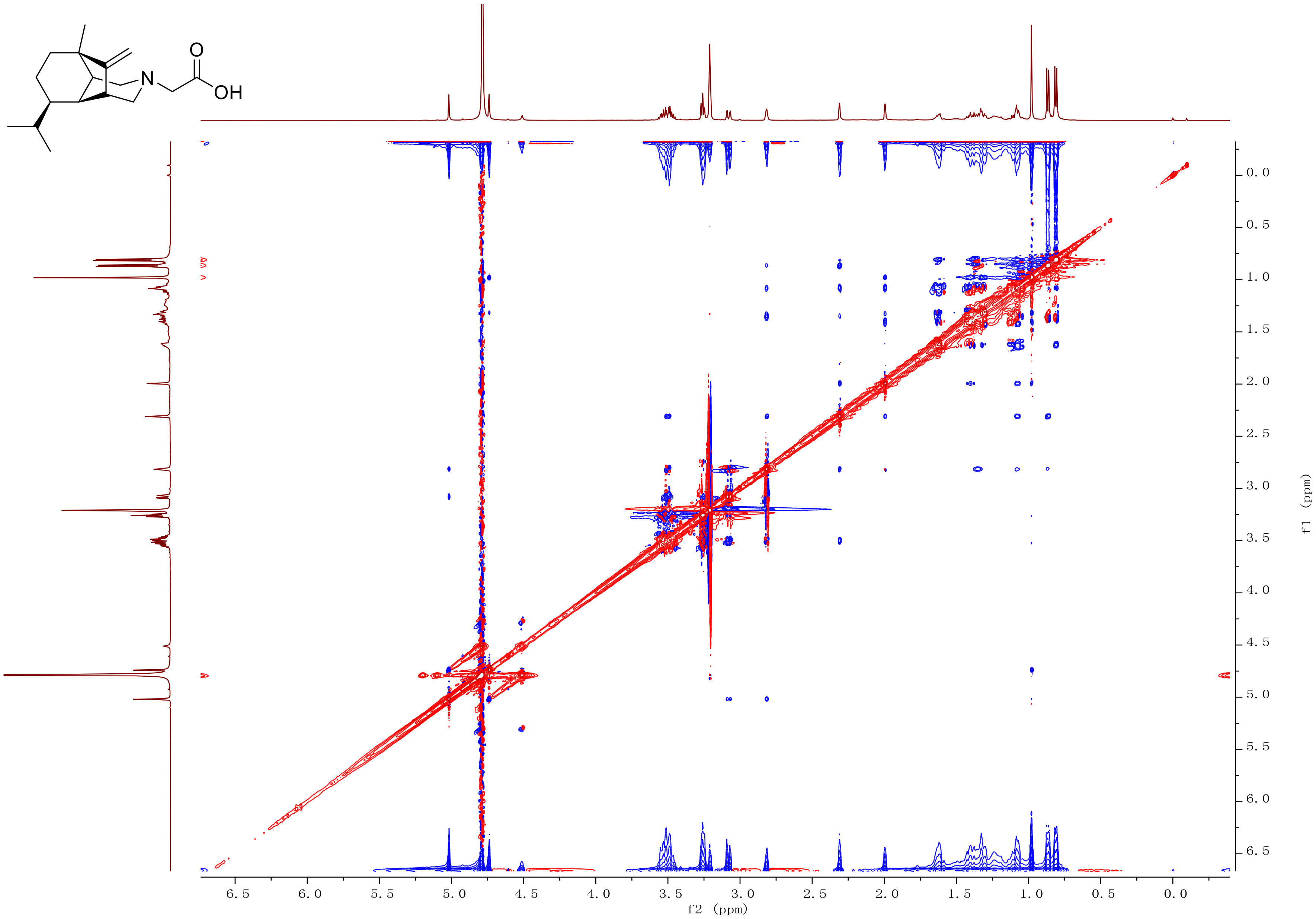

Figure S156. HR-ESIMS of compound **19**

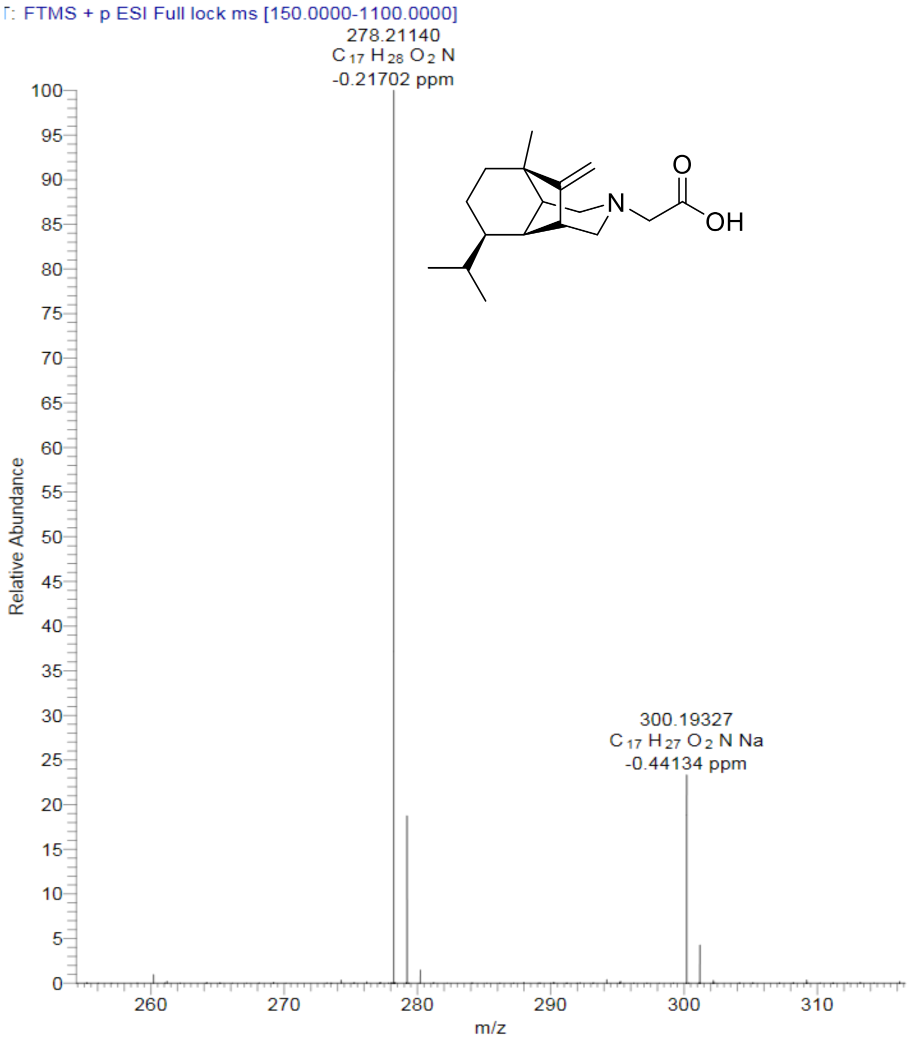

Supplement: Supplementary file 1 [file jof-07-01052-s001.zip › Supporting information.pdf]
